# Synthesis of Diversified Pyrazolo[3,4-*b*]pyridine Frameworks from 5-Aminopyrazoles and Alkynyl Aldehydes via Switchable C≡C Bond Activation Approaches

**DOI:** 10.3390/molecules27196381

**Published:** 2022-09-27

**Authors:** Xiao-Yu Miao, Yong-Ji Hu, Fu-Rao Liu, Yuan-Yuan Sun, Die Sun, An-Xin Wu, Yan-Ping Zhu

**Affiliations:** 1 Key Laboratory of Molecular Pharmacology and Drug Evaluation, Ministry of Education, Collaborative Innovation Center of Advanced Drug Delivery System and Biotech Drugs in Universities of Shandong, School of Pharmacy, Yantai University, Yantai 264005, China; 2 Key Laboratory of Pesticide & Chemical Biology, Ministry of Education, College of Chemistry, Central China Normal University, Wuhan 430079, China

**Keywords:** pyrazolo[3,4-*b*]pyridine, alkyne activation, regional selectivity, *6-endo-dig* cyclization

## Abstract

A cascade *6-endo-dig* cyclization reaction was developed for the switchable synthesis of halogen and non-halogen-functionalized pyrazolo[3,4-*b*]pyridines from 5-aminopyrazoles and alkynyl aldehydes via C≡C bond activation with silver, iodine, or NBS. In addition to its wide substrate scope, the reaction showed good functional group tolerance as well as excellent regional selectivity. This new protocol manipulated three natural products, and the arylation, alkynylation, alkenylation, and selenization of iodine-functionalized products. These reactions demonstrated the potential applications of this new method.

## 1. Introduction

**Figure 1:**
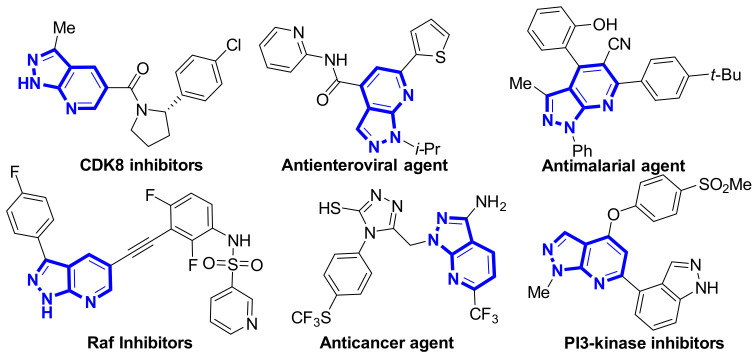
Some biologically active pyrazolo[3,4-*b*]pyridine derivatives.

A series of natural products and biologically active molecules contain pyrazolo[3,4-*b*]pyridine as a key structural motif [1,2]. Several of these compounds are effective antienterovirals, antimalarials, anticancer agents, and kinase inhibitors (Figure 1) [3,4,5]. This has inspired the development of efficient methods to construct these compounds and has become a hot topic in modern organic synthesis.

**Scheme 1:**
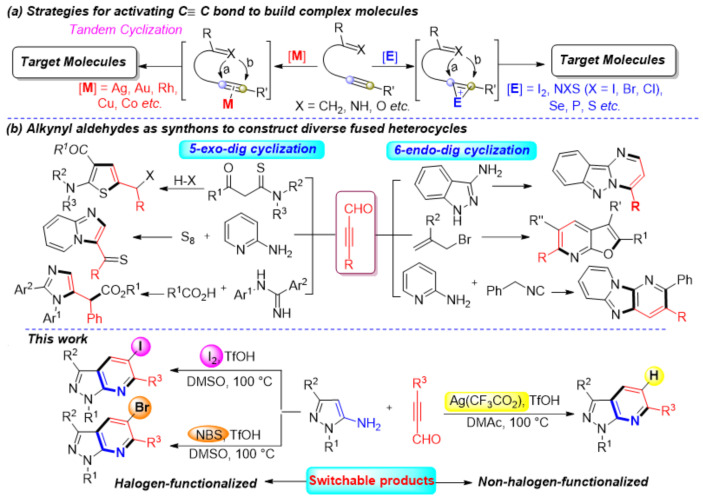
Strategies for the synthesis of diverse molecules via activating C≡C bond.

Recently, catalytic carbon-carbon bond activation has emerged as a useful tool to build complex molecules rapidly and efficiently [6,7,8,9]. There are versatile intermediates involved in these reactions, which could be trapped in situ by a second molecule that triggers subsequent tandem reactions [10,11,12,13,14,15]. The nucleophilic/electrophilic addition reactions of alkynes are well-known and provide a convenient way to synthesize functionalized molecules [16,17,18,19,20]. The high reactivity, good selectivity, excellent functional-group tolerance, and mild reaction conditions of these reactions have inspired significant research over the past few decades. Generally, this process forms highly active intermediates using transition metals, such as Ag, Au, Rh, Cu, and Co [21,22,23,24,25,26,27], or electrophiles like I_2_, NXS (X = I, Br, Cl), Se, S, and P (Scheme 1a) [28,29,30,31,32,33,34]. Each reagent type sees significant use in the development of synthetic methodologies and applications to prepare bioactive compounds or complex naturally occurring skeletons. However, to our knowledge, among those strategies, a direct and efficient protocol for the selective synthesis of polysubstituted and functionalized fused heterocycles, such as halogen-functionalized pyrazolo[3,4-*b*]pyridine frameworks, by C≡C bond activation has seldom been described. Thus, developing convenient and sustainable synthetic methods to build these high-value compounds merits attention.

As a kind of synthetic block with bifunctional groups (C≡C and carbonyl), alkynyl aldehydes are essential synthons with rich and unexpected chemical properties [35,36,37,38]. Tandem cyclization reactions using alkynyl aldehydes as synthons yields a variety of heterocycles. Generally, tandem cyclization occurs in one of two ways, *5-exo-dig* or *6-endo-dig* cyclizations. For example, some efficient synthesis strategies have been reported for the synthesis of multi-substituted thiazoles, imidazo[1,2-*a*]pyridines, and imidazoles by using alkynyl aldehydes as synthons via *5-exo-dig* cyclization [39,40,41]. The *6-endo-dig* cyclization of alkynyl aldehydes is an alternative method to construct complex fused ring systems (Scheme 1b) [42,43,44]. These protocols typically use simple starting materials, with good functional tolerance and high yields. Inspired by these achievements, we sought to selectively activate the C≡C bond by changing the reaction conditions to obtain a series of compounds with a pyrazolo[3,4-*b*]pyridine structure core.

## 2. Results and Discussion

To evaluate our idea, we chose 3-methyl-1-phenyl-1*H*-pyrazol-5-amine (**1a**) and 3-phenylpropiolaldehyde (**2a**) as the model substrates for the optimization of the conditions. Through optimization of the catalyst, additive, solvent and temperature, the optimal reaction conditions can be summarized as follows: **1a** (0.2 mmol) and **2a** (0.2 mmol) in DMAc (1.5 mL) with Ag(CF_3_CO_2_) (10 mol%), TfOH (30 mol%), at 100 °C for 2 h (details appear in Supplementary Materials Tables S3–S6).

**Scheme 2:**
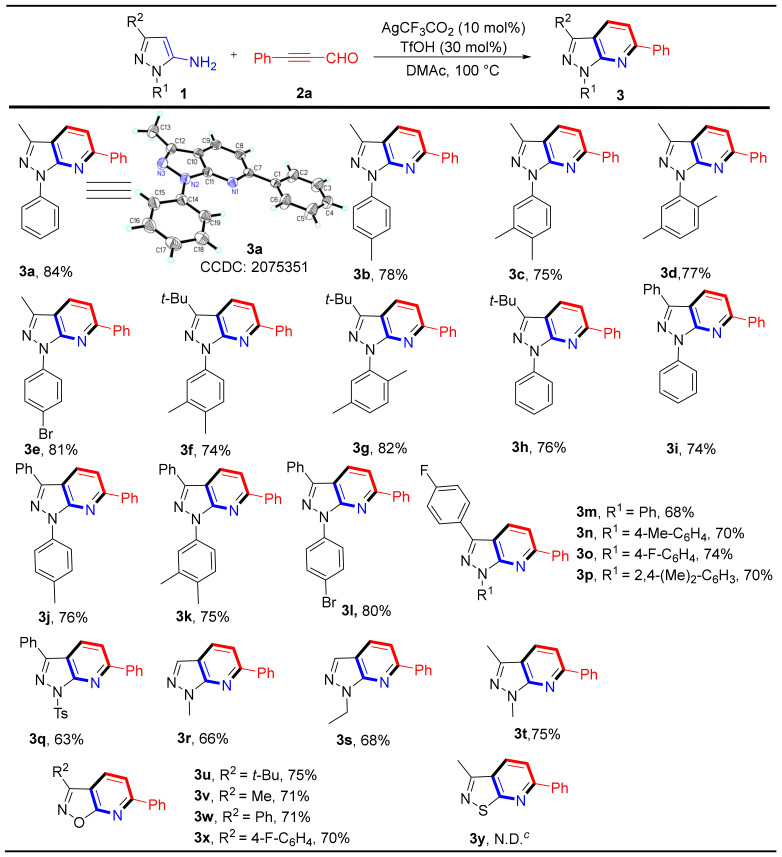
Substrate scope and Isolated yield of substituted 5-aminopyrazoles and derivatives. Reaction conditions: **1** (0.2 mmol), 2**a** (0.2 mmol), Ag(CF_3_CO_2_) (10 mol%), TfOH (30 mol%) in DMAc (1.5 mL) at 100 °C for 2 h. *^c^* N.D. = not detected.

Having optimized the reaction conditions, we determined the versatility of this reaction. We examined a series of 5-aminopyrazole derivatives to test the generality of this method and evaluate the electronic influence of aromatic ring substitutions. As shown in Scheme 2, pyrazole rings bearing electron-donating groups (e.g., 3-Me, 3-(t-Bu), 1-Ph, 3-Ph) led to good yields (74–84%) of the corresponding products (**3a**–**3d** and **3f**–**3k**). Notably, the structure of compound **3a** was confirmed by X-ray single-crystal diffraction (Scheme 2). The substrates of aromatic rings attached to halogen atoms (e.g., 4-F, 4-Br) also led to their corresponding products (**3e** and **3l**–**3p**) in yields between 68–81%. A strongly electron-deficient substrate was applied and afforded its product in 63% yield for the corresponding product **3q**. Pyrazole rings only bearing alkyl groups were used as starting materials and yielded the expected products (**3r**–**3t**) in moderate to good yields (66–75%). Additionally, 5-aminoisoxazoles also readily reacted with 3-phenylpropiolaldehyde, yielding the desired products (**3u**–**3x**) in good yields (70–75%). However, 3-methylisothiazol-5-amine did not yield the desired product **3y**.

**Scheme 3:**
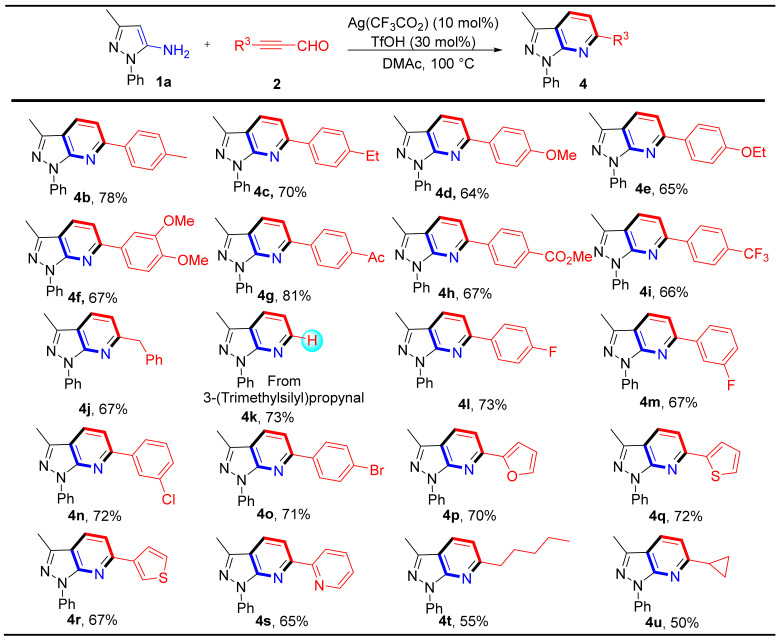
Substrate scope and Isolated yield of substituted alkynyl aldehydes and derivatives. Reaction conditions: **1a** (0.2 mmol), **2** (0.2 mmol), Ag(CF_3_CO_2_) (10 mol%), TfOH (30 mol%) in DMAc (1.5 mL) at 100 °C for 2 h.

We next investigated the scope of alkynyl aldehydes derivatives for this reaction (Scheme 3). First, we examined 3-phenylpropiolaldehydes with phenyl rings containing electron-rich substituents (e.g., 4-Me, 4-Et, 4-OMe, 4-OEt, 3,4-(OMe)_2_). Annulation reactions occurred smoothly to deliver products (**4b**–**4f**) in 64–78% yields. For 3-phenylpropiolaldehyde containing electron-withdrawing groups (e.g., 4-Ac, 4-CO_2_Me, 4-CF_3_, 4-F, 3-F, 3-Cl, 4-Br), the reaction proceeded smoothly and afforded products (**4g**–**4i** and **4l**–**4o**) in moderate to good yields (66–81%). In addition, when 4-phenylbut-2-ynal and 3-(trimethylsilyl)propiolaldehyde were used as starting materials, products **4j** and **4k** were obtained in good yields (67% and 73% respectively). It is worth noting that when using 3-(trimethylsilyl)propiolaldehyde, compound **4k** was the product of the trimethylsilyl group removal. Furthermore, different heterocyclic aldehydes were also investigated including furan, thiophene, and pyridine to generate products **4p**–**4s** in 65–72% yields. We were delighted to find that alkyl alkynyl aldehydes gave the corresponding products **4t**-**4u** in moderate yields as well.

The iodinated product was detected when 1.0 equivalent of iodine was added to the reaction system (control experiment, Scheme 7d). We chose **1a** and **2a** as model substrates to investigate the optimal conditions to synthesize iodinated products (more details appear in Supplementary Materials Tables S7 and S8).

**Scheme 4:**
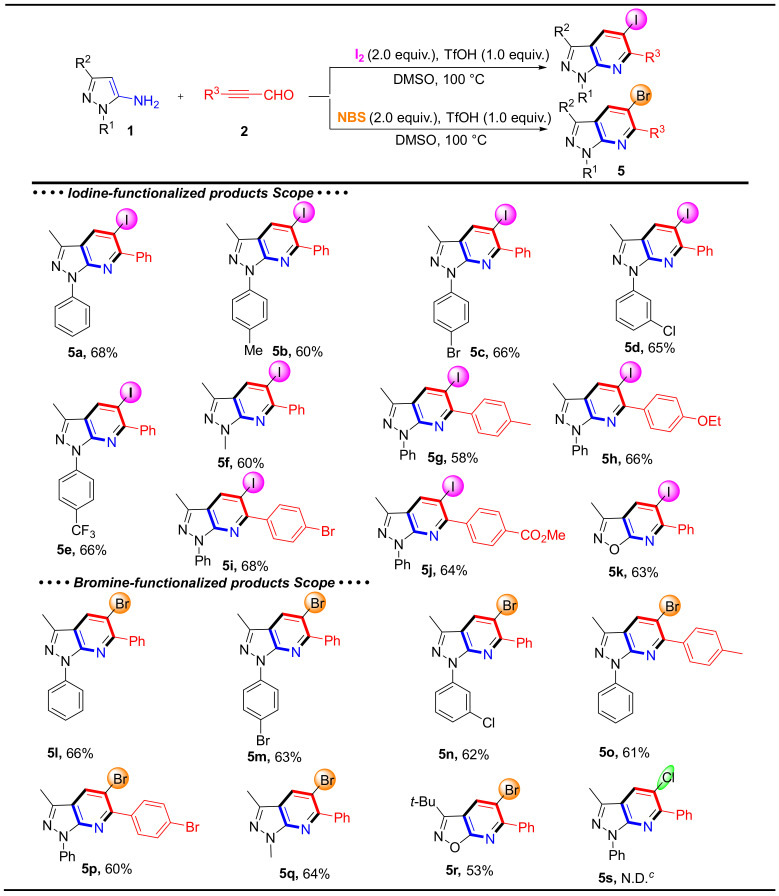
The substrate scope and Isolated yield of halogen-functionalized products. Reaction conditions: **1** (0.2 mmol), **2** (0.2 mmol), I_2_ or NBS (2.0 equiv.), TfOH (1.0 equiv.) in DMSO (2 mL) at 100 °C for 6 h. *^c^* N.D. = not detected.

Next, various 5-aminopyrazoles and alkynyl aldehydes were tested to determine the scope of iodine-functionalized products (Scheme 4). These reactions produced the corresponding products **5a**–**5j** 58–68% yields. Meanwhile, 3-methylisoxazol-5-amine tolerated the reaction conditions and reacted with 3-phenylpropiolaldehyde (**2a**) to generate **5k** in moderate yield. When iodine was replaced by NBS, the expected compounds **5l–5r** were obtained in moderated yields (53–66%). However, after many trials, the Cl-functionalized product **5s** was not obtained.

**Scheme 5:**
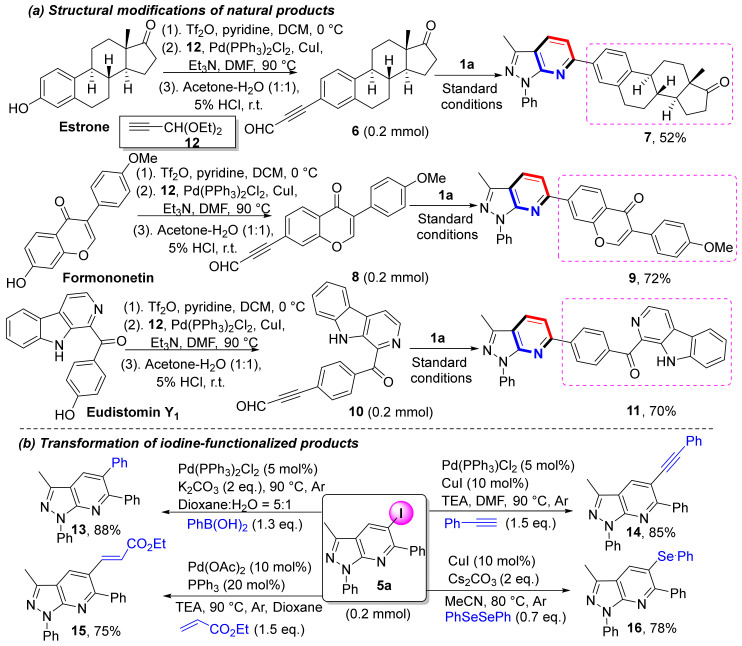
Strategies to synthesize diverse molecules via C≡C bond activation.

To demonstrate the applicability of this method, we modified natural products (Scheme 5a). For example, estrone, formononetin, and eudistomin Y_1_ are all biologically active natural products, and these compounds have phenolic hydroxyl groups that undergo conversion into trifluoromethane sulfonates. Those sulfonates undergo Sonogashira coupling and deketalization to afford alkynaldehyde intermediates (**6**, **8**, and **10**). By using these alkynaldehyde intermediates as substrates for this protocol, we successfully obtained three natural product functionalized pyrazolo[3,4-*b*]pyridines in moderate to good yields (**7**, **9**, and **11**). Because heteroaryl iodides are highly useful functional structures in synthetic organic chemistry, additional applications of iodine-functionalized products were conducted (Scheme 5b) [45]. A series of coupling reactions were examined to form iodine-functionalized products, including Suzuki, Sonogashira, and Heck couplings that yielded the expected products **13**–**15** in good yields. Furthermore, selenization of iodine-functionalized products afforded **16** in very good yield (78%).

**Scheme 6:**
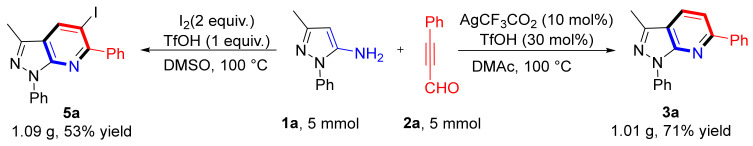
Scale-up reactions.

The reaction of **1a** and **2a** was scaled up to 5 mmol to illustrate the potential applications of this method; **3a** and **5a** formed 71% and 53% yields, respectively (Scheme 6). This promising result lays a good foundation for large-scale syntheses.

**Scheme 7:**
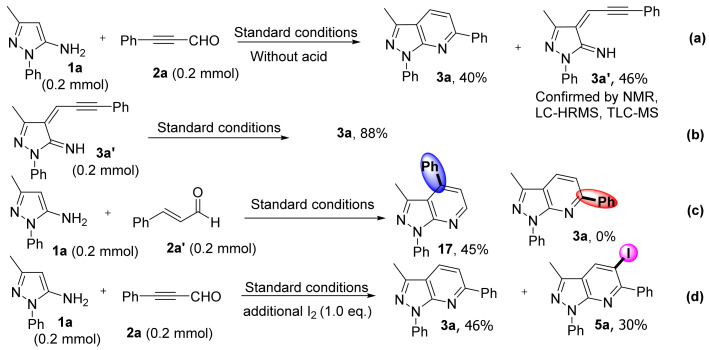
Control experiments. (**a**) Form intermediate **3a**′. (**b**) Intermediate **3a**′ transform to **3a** under standard conditions. (**c**) Validation of regioselectivity experiments. (**d**) Add one equivalent of iodine under standard conditions.

Relevant control experiments were conducted to probe the reaction mechanism for the formation of pyrazolo[3,4-*b*]pyridine frameworks. When the reaction of **1a** with **2a** was conducted without acid for 2 h, it afforded **3a** and **3a**′ in 40% and 46% yields, respectively, (Scheme 7a). Intermediate **3a**′ was confirmed by TLC-MS(APCI), LC-HRMS, and NMR (the details can be seen in Supplementary Materials). In addition, intermediate **3a**′ transform to **3a** in 88% yield under standard conditions (Scheme 7b). These results suggested that **3a**′ may serve as the intermediate in this reaction. To illustrate regioselectivity, we chose cinnamaldehyde (**2a**′) as a substrate to react with **1a** under standard conditions. Compared with a standard simple, 3-methyl-1,4-diphenyl-1*H*-pyrazolo[3,4-*b*]pyridine (**17**) was obtained in 45% yield, no product **3a** was observed (Scheme 7c). These results confirmed the regioselectivity of this method, as it only afforded the C6 substituted pyrazolo[3,4-*b*]pyridine for alkynyl aldehydes substrates. Furthermore, when 1 eq. of iodine was added, non-iodinated and iodized products **3a** and **5a** were detected in 46% and 30% yields, respectively (Scheme 7d).

**Scheme 8:**
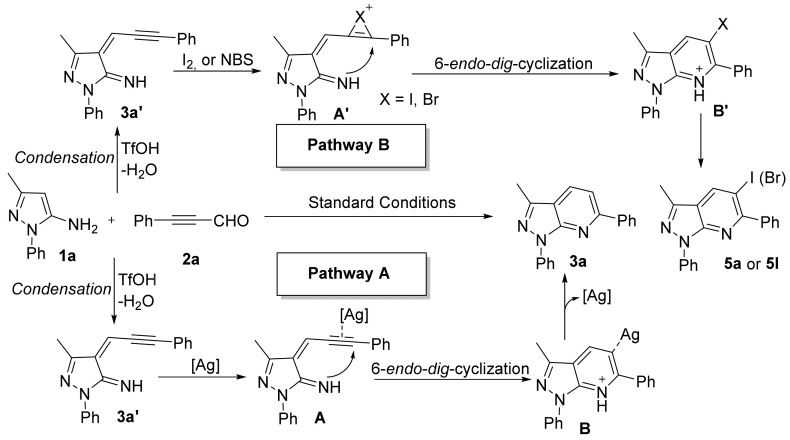
Plausible mechanistic pathway.

Considering the aforementioned control experiments and earlier works [46,47,48], a reaction mechanism is shown in Scheme 8 using **1a** with **2a** as a typical reaction. Initially, 3-methyl-1-phenyl-1*H*-pyrazol-5-amine (**1a**) undergoes condensation with 3-phenylpropiolaldehyde (**2a**) to form intermediate **3a**′. Next, the silver salt coordinates to the alkyne of **3a**′ to form intermediate **A**; this undergoes *6-endo-dig* cyclization to form **B**. Finally, **B** undergoes demetallation to afford product **3a** (Scheme 8, pathway A). Similarly, I_2_ or NBS adds to a triple bond that leads to intermediate **A’**, which undergoes *6-endo-dig* cyclization to form **B’**, followed by acid loss from **B’** to obtain **5a** or **5l** (Scheme 8, pathway B).

## 3. Materials and Methods

### 3.1. General Information

Aminopyrazoles and NBS were purchased from Shanghai Shaoyuan Co. Ltd. (Shanghai, China) 3-substituted propiolaldehyde and Ag(CF_3_CO_2_) were purchased from Leyan. Unless stated otherwise, all solvents and commercially available reagents were obtained from commercial suppliers and used without further purification. In addition, petroleum ether (b.p. 60–90 ^o^C) was distilled prior to use for Column chromatography. Non-commercial starting materials were prepared as described below or according to literature procedures. TLC analysis was performed using pre-coated glass plates. Column chromatography was performed using silica gel (200–300 mesh). Nuclear magnetic resonance (NMR) spectra were recorded on a Bruker Advance 400 MHz spectrometer at ambient temperature using the non or partly deuterated solvent as internal standard (^1^H: *δ* 7.26 ppm and ^13^C{^1^H}: *δ* 77.0 ppm for CDCl_3_; ^1^H: *δ* 2.50 ppm and ^13^C{^1^H}: *δ* 40.0 ppm for DMSO-*d*_6_). Chemical shifts (*δ*) are reported in ppm, relative to the internal standard of tetramethylsilane (TMS). The coupling constants (*J*) are quoted in hertz (Hz). Resonances are described as s (singlet), d (doublet), t (triplet), q (quartet), m (multiplet), br (broad) or combinations thereof. High resolution mass-spectrometric (HRMS) were obtained on an Apex-Ultra MS equipped with an electrospray source. Melting points were determined using SGW X-4 apparatus and not corrected. The X-ray diffraction data for the crystallized compound were collected on a Bruker Smart APEX CCD area detector diffractometer (graphite monochromator, Mo Kα radiation, λ = 0.71073 Å) at 296(2) K. All the heating procedures were conducted with an oil bath.

### 3.2. Synthetic Procedures

**Typical Procedure (TP 1) for the Synthesis of 3 and 4 Taking 3a as an Example.** A 25 mL pressure vial was charged with **1a** (34.6 mg, 0.20 mmol, 1.0 equiv.), **2a** (26 mg, 24.5 uL, 0.20 mmol, 1.0 equiv.), Ag(CF_3_CO_2_) (4.4 mg, 0.02 mmol, 10 mol%), TfOH (9 mg, 5.3 uL, 0.06 mmol, 30 mol%) and DMAc (1.5 mL).The vial was sealed and the reaction mixture was stirred at 100 °C for 2 h under air atmosphere (monitored by TLC). After the reaction was completed, and added 50 mL water to the mixture, then extracted with EtOAc 3 times (3 × 50 mL). The solution was dried over anhydrous Na_2_SO_4_, concentrated under reduced pressure, and dried under vacuum. The residue was purified by flash column chromatography by using ethyl acetate/petroleum ether mixture to obtain the corresponding product **3a**.

**Typical Procedure (TP 2) for the Synthesis of 5 Taking 5a as an Example.** A 25 mL pressure vial was charged with **1a** (34.6 mg, 0.20 mmol, 1.0 equiv.), **2a** (26 mg, 24.5 uL, 0.20 mmol, 1.0 equiv.), I_2_ (101.5 mg, 0.40 mmol, 2.0 equiv.), TfOH (30 mg, 17.6 uL, 0.20 mmol, 1.0 equiv.) and DMSO (2.0 mL). The vial was sealed, and the reaction mixture was stirred at 100 °C for 6 h under air atmosphere (monitored by TLC). After the reaction was completed, and added 50 mL water to the mixture, then extracted with EtOAc 3 times (3 × 50 mL). The extract was washed with 10% Na_2_S_2_O_3_ solution, dried over anhydrous Na_2_SO_4_ and concentrated under reduced pressure. The residue was purified by flash column chromatography by using ethyl acetate/petroleum ether mixture to obtain the corresponding product **5a**.

**General Procedure for Synthesis of 13.** K_2_CO_3_ (0.4 mmol, 2.0 equiv.), phenylboronic acid (0.26 mmol, 1.3 equiv.) and PdCl_2_(PPh_3_)_2_ (5 mol%) were added to a solution of **5a** (0.2 mmol, 1.0 equiv.) in a 5:1 solvent mixture of dioxane and water. The reaction mixture was heated to 90 °C and stirred at this temperature until complete consumption of **5a** was observed (monitored by TLC). After cooling to room temperature, the mixture was diluted with a mixture of EA and water and the aqueous layer was extracted with EtOAc (3 × 50 mL). dried over anhydrous Na_2_SO_4_ and concentrated under reduced pressure. The residue was purified by flash column chromatography by using ethyl acetate/petroleum ether mixture to obtain the desired product **13** in 88% yield.

**General Procedure for Synthesis of 14. 5a** (0.2 mmol, 1 equiv.), PdCl_2_(PPh_3_)_2_ (5 mol%), CuI (10 mol%) and phenylacetylene (0.3 mmol, 1.5 equiv.) were added to a 25 mL Schlenk flask with a stir bar under an Ar atmosphere. Then DMF (2 mL) and TEA (1 mL) were added sequentially. The reaction mixture was then stirred at 90 °C. Afterwards 15 mL of water were added, and the reaction mixture was extracted with EtOAc (3 × 50 mL). The combined organic fractions were washed with brine and dried over Na_2_SO_4_. After filtration, the solvent was removed under reduced pressure. The residue was purified by flash column chromatography by using ethyl acetate/petroleum ether mixture to obtain the desired product **14** in 85% yield.

**General Procedure for Synthesis of 15. 5a** (0.2 mmol, 1 equiv.), Pd(OAc)_2_ (10 mol%), PPh_3_ (20 mol%) and ethyl acrylate (0.3 mmol, 1.5 equiv.) were added to a 25 mL Schlenk flask with a stir bar under an Ar atmosphere. Then dioxane (2 mL) and TEA (1 mL) were added sequentially. The reaction mixture was then stirred at 90 °C. Afterwards 50 mL of water were added, and the reaction mixture was extracted with EtOAc (3 × 50 mL). The combined organic fractions were washed with brine and dried over Na_2_SO_4_. After filtration, the solvent was removed under reduced pressure. The residue was purified by flash column chromatography by using ethyl acetate/petroleum ether mixture to obtain the desired product **15** in 75% yield.

**General Procedure for Synthesis of 16.** Adapting a literature procedure [49], A 25 mL Schlenk flask with a stir bar was charged with **5a** (0.2 mmol, 1.0 equiv.), diphenyl diselenide (0.14 mmol, 0.7 equiv.) CuI (0.02 mmol, 10 mol%) and Cs_2_CO_3_ (0.4 mmol, 2.0 equiv.) in MeCN (2.0 mL). The vial was sealed and the resulting mixture was stirred at 80 °C for 24 h under an Ar atmosphere. After the reaction completed, and added 50 mL water to the mixture, then extracted with EtOAc 3 times (3 × 50 mL). The extract was washed with brine, dried over anhydrous Na_2_SO_4_ and concentrated under reduced pressure. The residue was purified by flash column chromatography by using ethyl acetate/petroleum ether mixture to obtain the desired product **16** in 78% yield.

## 4. Conclusions

In summary, a cascade *6-endo-dig* cyclization reaction was developed for the switchable synthesis of halogen and non-halogen-functionalized pyrazolo[3,4-*b*]pyridines from 5-aminopyrazoles and alkynyl aldehydes. This method afforded diversified pyrazolo[3,4-*b*]pyridine frameworks via C≡C bond activation with silver, iodine, or NBS. The protocol was characterized by a wide substrate scope, good functional group tolerance, and excellent regional selectivity. The structural modification of estrone, formononetin, and eudistomin Y_1_ provided new ideas for syntheses of drug molecules. Iodine functionalization allowed several additional transformations, including arylation, alkenylation, alkynylation, and selenization to fabricate useful molecules.

## 5. Patents

A patent (Yantai University, CN 112300157, and 2021 A) has been derived from this manuscript. The patent is entitled Novel pyrazolopyridine compound with antitumor activity and preparation method thereof.

## Data Availability

Not applicable.

## Supplementary Materials

The following supporting information can be downloaded at: https://www.mdpi.com/article/10.3390/molecules27196381/s1, Characterization data for product **3**, **4**, **5**, **6–11**, and **13–17**, include ^1^H- and ^13^C-NMR spectroscopies are available online. CCDC 2075351 contain the supplementary crystallographic data for this paper. These data can be obtained free of charge via www.ccdc.cam.ac.uk/data_request/cif, or by emailing data_request@ccdc.cam.ac.uk, or by contacting The Cambridge Crystallographic Data Centre, 12 Union Road, Cambridge CB2 1EZ, UK; Fax: +44 1223 336033. References [50,51,52,53,54,55,56] are cited in the Supplementary Materials.

## Sample Availability

Samples of the compounds **3**, **4**, and **5** are available from the authors.
