# Supplementary material for: Synthesis of Diversified Pyrazolo[3,4-b]pyridine Frameworks from 5-Aminopyrazoles and Alkynyl Aldehydes via Switchable C≡C Bond Activation Approaches
Source: Molecules. 2022 Sep 27;27(19):6381. doi: 10.3390/molecules27196381 (PMC9571537; doi:10.3390/molecules27196381)
Supplement: Supplementary file 1 [file molecules-27-06381-s001.zip › molecules-1925181-supplementary.pdf]

Supplementary Materials

Synthesis of Diversified Pyrazolo[3,4-*b*]pyridine Frameworks from 5-Aminopyrazoles and Alkynyl aldehydes via Switchable C≡C bond Activation Approaches

Xiao-Yu Miao,[1†] Yong-Ji Hu,[1†] Fu-Rao Liu,[1] Yuan-Yuan Sun,[1] Die Sun,[1] An-Xin Wu,[2*] Yan-Ping Zhu[1*]

[1] School of Pharmacy, Key Laboratory of Molecular Pharmacology and Drug Evaluation, Ministry of Education, Collaborative Innovation Center of Advanced Drug Delivery System and Biotech Drugs in Universities of Shandong, Yantai University, Shandong, Yantai, 264005, P. R. China

[2] Key Laboratory of Pesticide & Chemical Biology, Ministry of Education, College of Chemistry, Central China Normal University, Hubei, Wuhan, 430079, P. R. China

[†] These authors contributed equally to this work.

Table of Contents

1. **General information** **S1**
2. **Mechanism investigation** **S1**
3. **Experimental Procedures** **S8**
4. **Characterization of Products** **S15**
5. **Copy of ^{1}H and ^{13}C NMR Spectra of Products** **S38**

1. General information

Materials and General Experimental: Aminopyrazoles and NBS were purchased from Shanghai Shaoyuan Co. Ltd. 3-Phenylpropiolaldehyde and Ag(CF_3CO_2) were purchased from Leyan. Unless stated otherwise, all solvents and commercially available reagents were obtained from commercial suppliers and used without further purification. In addition, petroleumether (b.p. 60-90 ºC), which was used for column chromatography, was distilled prior to use. Non-commercial starting materials were prepared as described below or according to literature procedures. Analytical thin layer chromatography (TLC) was performed using pre-coated silica gel HF254 glass plates. Column chromatography was performed using silica gel (200-300 mesh).

Instrumentation: Nuclear magnetic resonance (NMR) spectra were recorded on a Bruker Advance 400 MHz spectrometer at ambient temperature using the non or partly deuterated solvent as internal standard (^{1}H: δ 7.26 ppm and ^{13}C{1H}: δ 77.0 ppm for $CDCl_3$,). Chemical shifts (δ) are reported in ppm, relative to the internal standard of tetramethylsilane (TMS). The coupling constants (*J*) are quoted in hertz (Hz). Resonances are described as s (singlet), d (doublet), t (triplet), q (quartet), m (multiplet), br (broad) or combinations thereof. High resolution mass spectra were obtained on Thermo Scientific Q-Exactive (ESI mode). Melting points were determined using SGW X-4 apparatus and not corrected.

2. Mechanism investigation

2.1 The control experimental results

When the reaction of **1a** with **2a** was conducted under the standard conditions without acid for 2 h, product **3a** was obtained in 40% yield and 5-methyl-2-phenyl-4-(3-phenylprop-2-yn-1-ylidene)-2,4-dihydro-3*H*-pyrazol-3-imine (**3a'**) could be detected by TLC-MS (APCI) and NMR (Figure S1-S4).

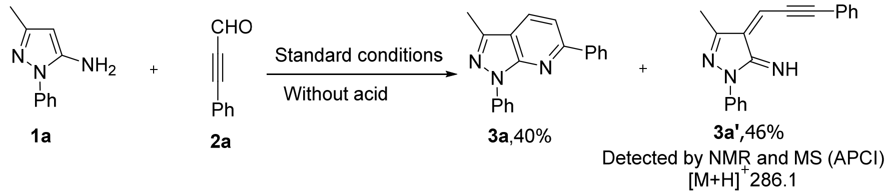

Scheme S1. Control experiment for the reaction of **1a** with **2a**.

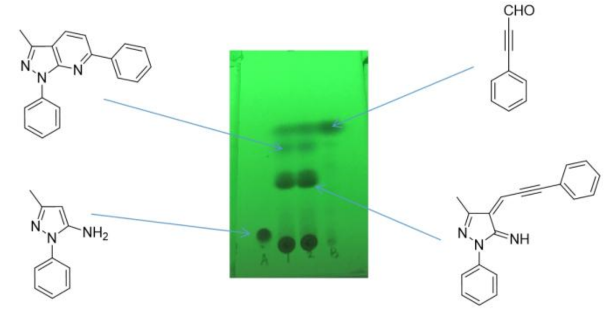

Figure S1. The TLC results for the reaction of **1a** with **2a**.

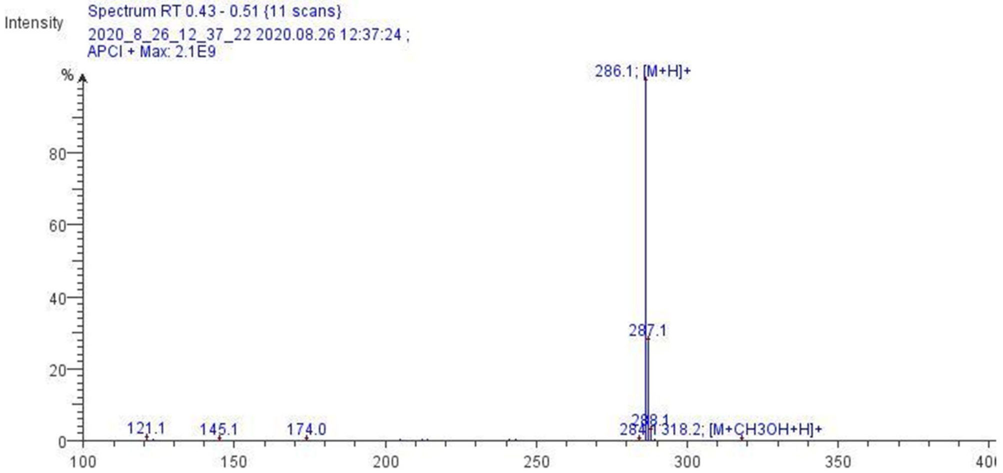

Figure S2. The MS (APCI) results for the **3a’**.

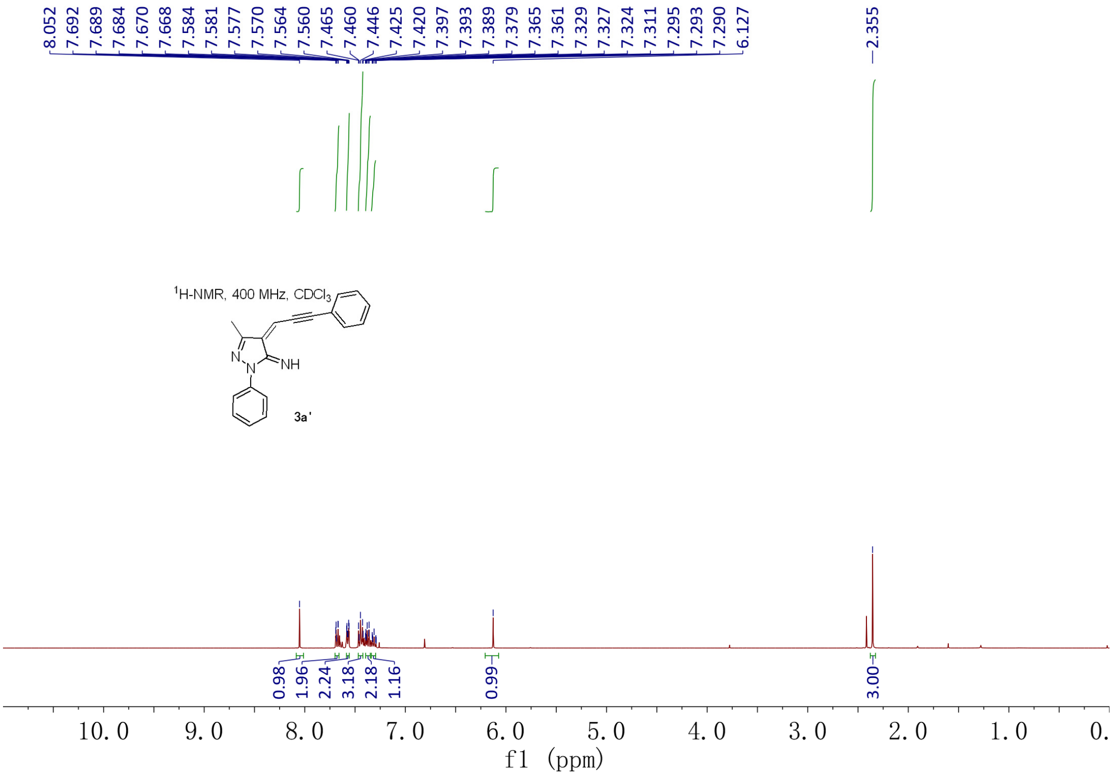

Figure S3. The copy of ^{1}H NMR spectra of product **3a’**.

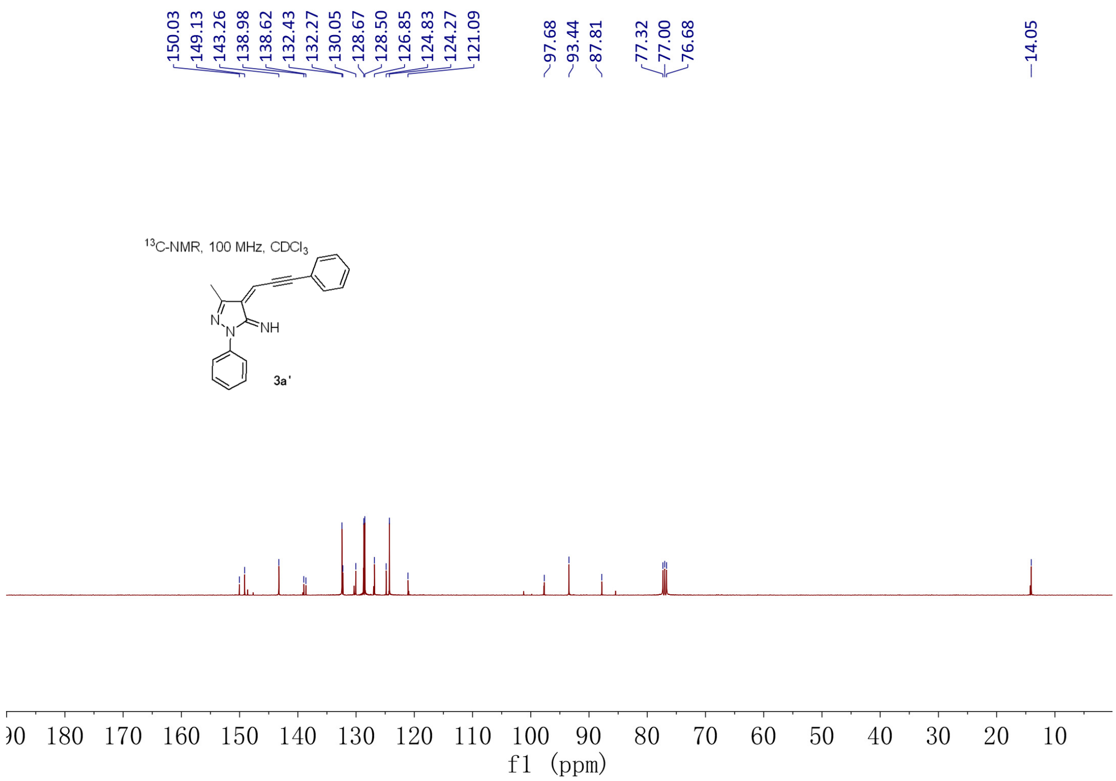

Figure S4. The copy of ^{13}C NMR spectra of product **3a'**.

In order to prove the regioselectivity of our method, we chosen cinnamaldehyde (**2a'**) as a substrate to react with **1a** under the standard conditions (Scheme S2). Compared with a standard simple, the 3-methyl-1,4-diphenyl-1*H*-pyrazolo[3,4-*b*]pyridine (**17)** was obtained in 45% yield (NMR see Figure S5-S6).

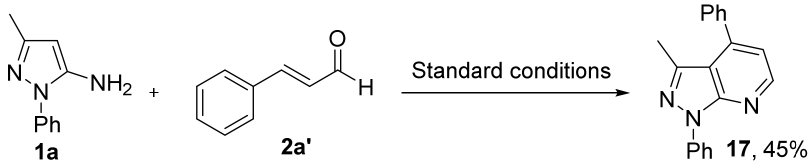

Scheme S2. Control experiment for the reaction of **1a** with **2a'**.

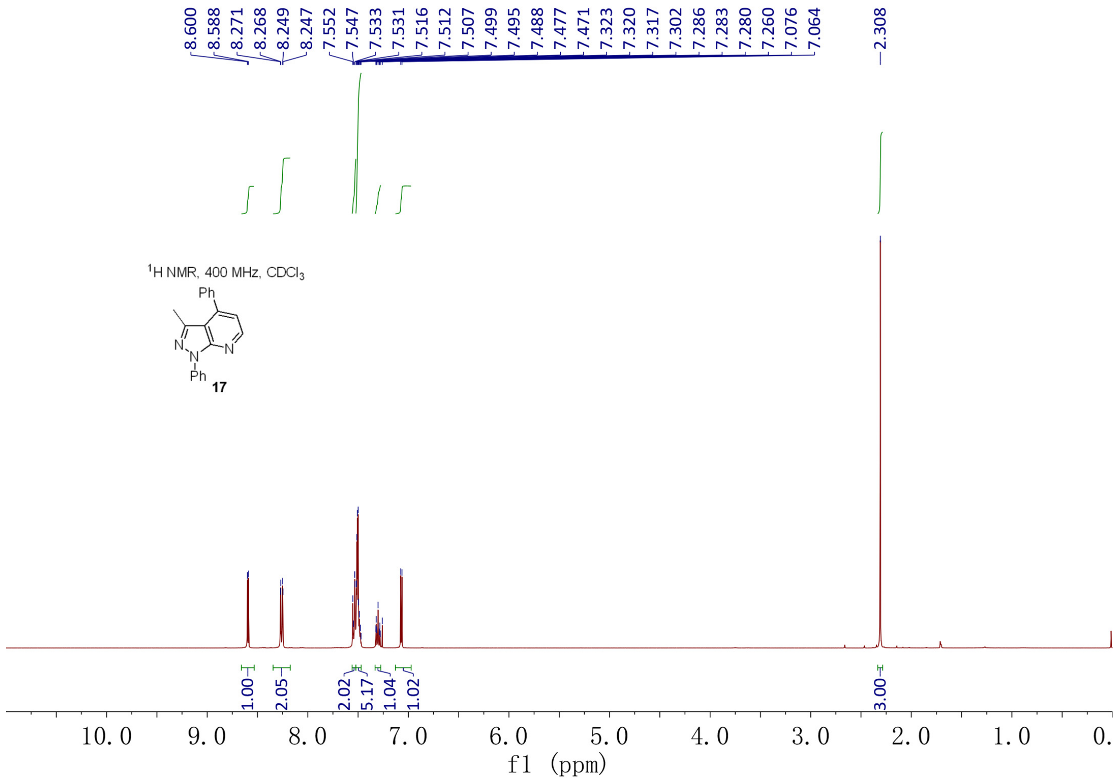

Figure S5. The copy of ^{1}H NMR spectra of product **17**.

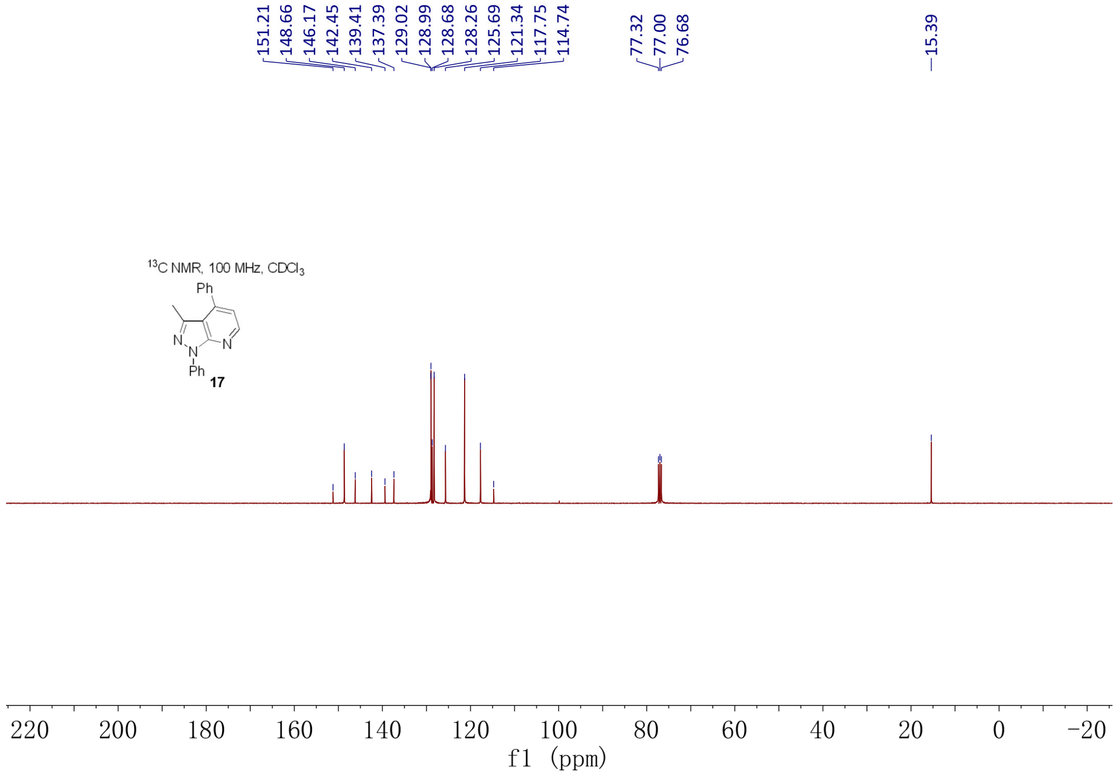

Figure S6. The copy of ^{13}C NMR spectra of product **17**.

2.2 The LC-HRMS results

In order to further confirm the potential intermediate 5-methyl-2-phenyl-4-(3-phenylprop-2-yn-1-ylidene)-2,4-dihydro-3*H*-pyrazol-3-imine

(3a') in the reaction. The reaction of **1a** with **2a** was conducted under the standard conditions without acid for 1 h, then analysis the reaction mixture by LC-HRMS. Through compared the standard sample, the intermediate **3a** could be observed and confirmed (See Figure S7-S11 and Table S1).

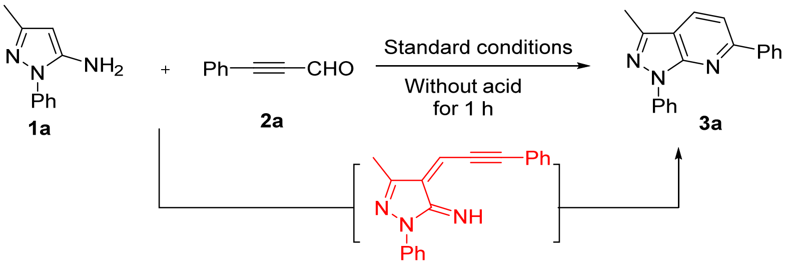

Scheme S3. Control experiment for the reaction of **1a** with **2a**.

HPLC conditions:
WATERS e2695
Chromatographic column: Agilent-TC-C18 (250 mm×4.6 mm, 5μm)
Flow rate: 1ml/min
Flow phase: MeOH: H_2O (with 0.1% HCO_2H),
0-3.5 min: 75:25; 3.5-4.0 min: 85:15; 4.0-20.0 min: 85:15
Wavelength: 254 nm

LC-MS:
LC conditions:
Thermo Scientific
Chromatographic column: Waters, ACQUITY UPLC BEH C18 column (2.1×50 mm, 1.7 μm)
Flow rate: 0.4 ml/min
Flow phase: MeOH: 0.1% HCO_2H (75: 25)
MS conditions: Thermo Scientific, Q-Exactive
Mass Spectrometry Scanning Mode: cations MS
Ion source: ESI

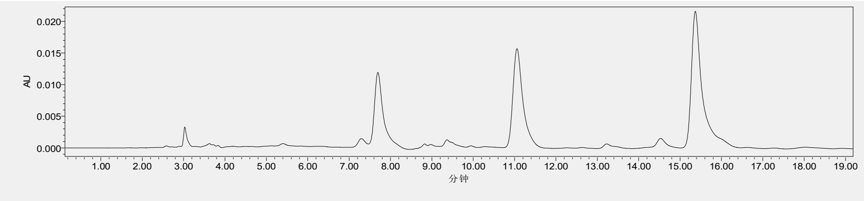

Figure S7. The chromatography results for the reaction mixture.

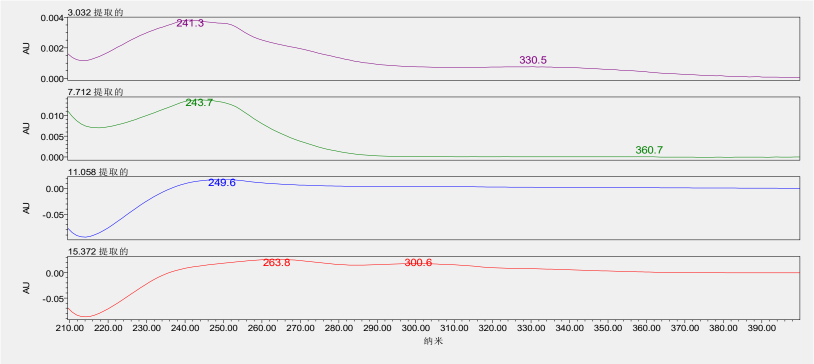

Figure S8. The spectrum for the reaction mixture.

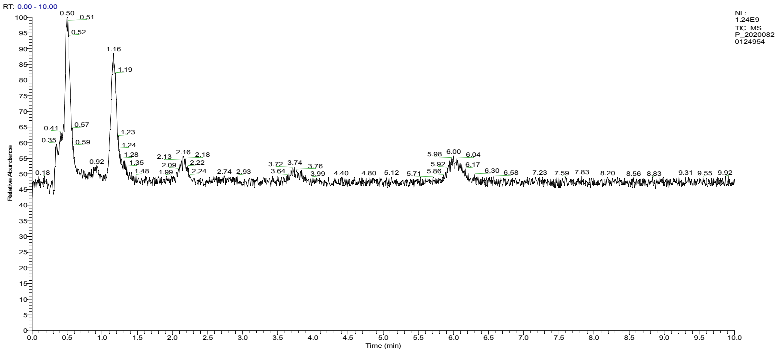

Figure S9. The information of LC-HRMS for the reaction mixture.

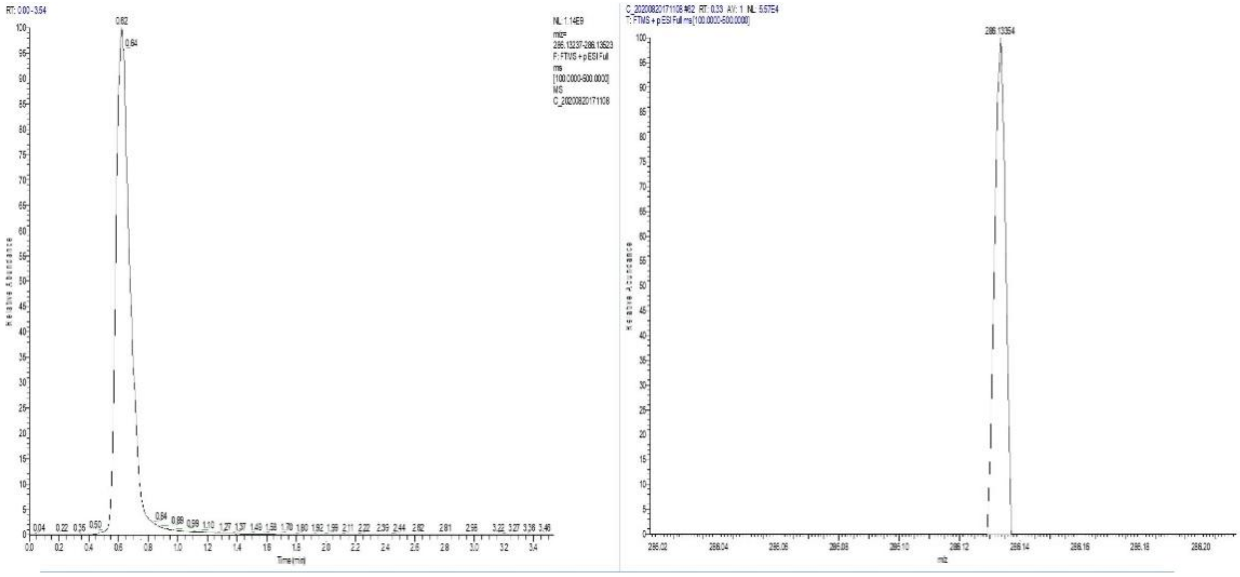

Figure S10. (a) Extracted ion chromatography for **3a'** (Standard Sample); (b) The HRMS result for standard sample **3a'**.

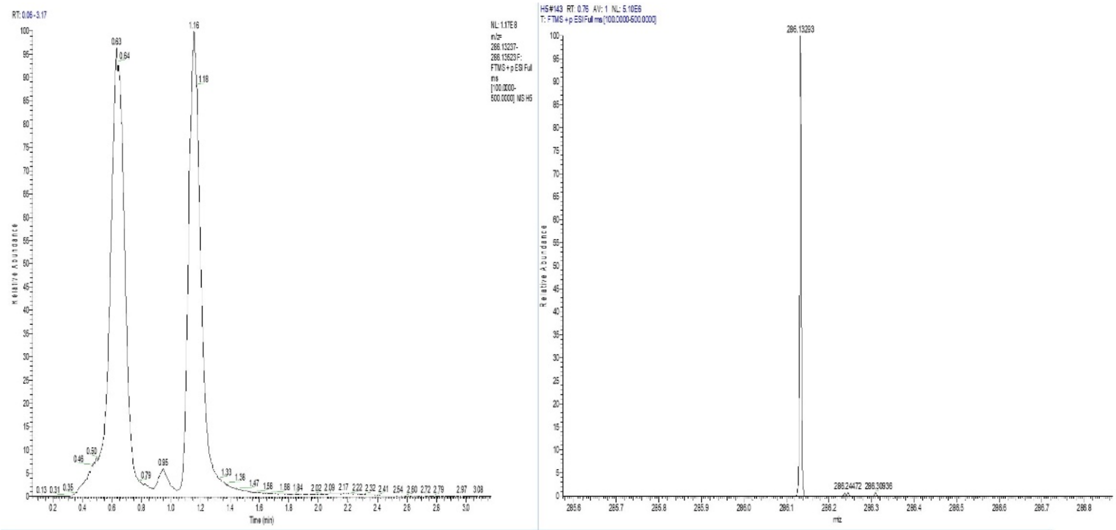

Figure S11. The information for intermediate **3a’** in the reaction mixture. (a) extracted ion chromatography for **3a’**（from the reaction mixture），0.63min; (b) The HRMS result for **3a’**.

Table S1. The summary of the LC-HRMS results.

Simple	Retention Time （LC）/min	wavelength /nm	Retention Time （MS）/min	Calcd for $(M+H)^+$	found	Measurement Error (ppm)
SS-**1a**	3.549	240.1	0.36	174.1026	174.1022	-2.320
RM-**1a**	3.032	241.3	0.5		174.1021	-3.009
SS-**2a**	4.47	237.8	0.43	131.0491	131.0490	-1.461
RM-**2a**	-	-	0.53		131.0489	-1.690
SS-**3a’**	6.997	336.5	0.62	286.1339	286.1335	-1.168
RM-**3a’**	-	-	0.63		286.1329	-3.299
SS-**3a**	11.924	234.2；263.8；300.6	1.16	286.1339	286.1335	-1.482
RM-**3a**	15.372	263.8；300.6	1.16		286.1330	-3.055

SS: Standard Sample，RM: Reaction Mixture

2.3 Molecular structure and crystallographic data

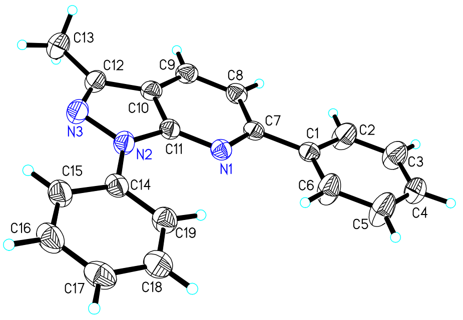

Figure S12. X-ray crystal structure of compound **3a**

Table S2. Crystal data and structure refinement for compound 3a.

Identification code	CCDC: 2075351
Empirical formula	$C_{19}H_{15}N_3$
Formula weight	285.34
Temperature/K	296(2)
Crystal system	orthorhombic
Space group	Fdd2
a/Å	24.743(8)
b/Å	45.272(14)
c/Å	5.2644(16)
α/°	90
β/°	90
γ/°	90
Volume/Å^3	5897(3)
Z	16
ρ_{calc}g/cm^3	1.286
μ/mm^{-1}	0.078
F(000)	2400.0
Crystal size/mm^3	0.12 × 0.1 × 0.1
Radiation	MoKα (λ = 0.71073)
2Θ range for data collection/	3.598 to 49.312
Index ranges	-28 ≤ h ≤ 26, -52 ≤ k ≤ 52, -6 ≤ l ≤ 6
Reflections collected	9715
Independent reflections	2477 [Rint = 0.0300, Rsigma = 0.0292]
Data/restraints/parameters	2477/1/200
Goodness-of-fit on F^2	1.046
Final R indexes [I>=2σ (I)]	R1 = 0.0376, wR = 0.0961
Final R indexes [all data]	R1 = 0.0456, wR2 = 0.1024
Largest diff. peak/hole / e Å^{-3}	0.12/-0.15
Flack parameter	-2.1(10)

3. Experimental Procedures

3.1 Reaction Optimization

We optimized the reaction conditions using 3-methyl-1-phenyl-1*H*-pyrazol-5-amine **(1a)** and 3-phenylpropiolaldehyde **(2a)** as model substrates. Initially, the reaction was carried out with various metal catalysts (10 mol%), such as $Cu(OAc)_2$, CuCl, CuBr, $Cu(OTf)_2$, CuO, $FeCl_3$, $Pd(OAc)_2$, $PdCl_2$, and AgOAc (Table S3, entries 1-9).When the reaction was performed with AgOAc as the catalyst (Table S3, entry 9), the reaction worked well to afford the desired product **3a** in 43% yield. Next, a series of silver catalysts were screened under the selected conditions (Table S3, entries 9-13). When $AgCF_3CO_2$ was employed as the catalyst, the yield was increased to 56% (Table S3, entry 13). Thus, $AgCF_3CO_2$ was chosen as the catalyst for further studies.

Table S3. Optimization for the metal catalysts of reaction. [a]

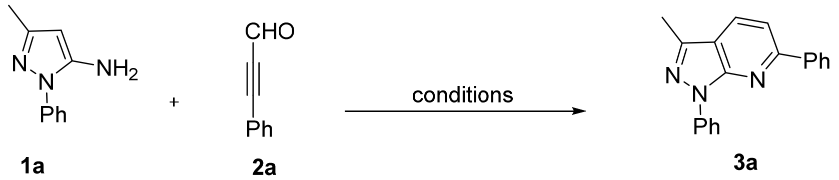

Entry	Cat.	Solvent	T(°C)	Yield(%)[b]
1	$Cu(OAc)_2$	DMAc	100	23
2	CuCl	DMAc	100	30
3	$CuBr_2$	DMAc	100	33
4	$Cu(OTf)_2$	DMAc	100	35
5	CuO	DMAc	100	N.D.[c]
6	$FeCl_3$	DMAc	100	30
7	$Pd(OAc)_2$	DMAc	100	N.D.[c]
8	$PdCl_2$	DMAc	100	12
9	AgOAc	DMAc	100	43
10	Ag_2SO_4	DMAc	100	20
11	$AgBF_4$	DMAc	100	35
12	Ag_2CO_3	DMAc	100	40
**13**	**$AgCF_3CO_2$**	**DMAc**	**100**	**56**

[a] Reaction conditions: **1a** (0.2 mmol), **2a** (0.2 mmol), cat. (10 mol%), solvent (1.5 mL), at 100 °C for 2 h under air atmosphere. [b] Isolated yield. [c] N.D.=not detected

After a systematic screening of silver salts, a serial of acids, such as TFA, TfOH, HCl, TsOH, HOAc, and TfOH were then examined (Table S4, entries 2-6). The results showed that TfOH was the most suitable choice, delivering **3a** in 84% yield (Table S4, entry 3).

Table S4. Optimization for the acid of reaction. [a]

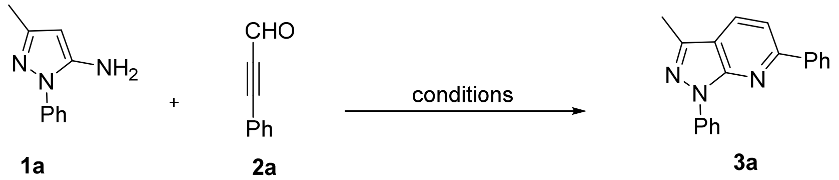

Entry	Cat.	Acid	Solvent	T(°C)	Yield(%)[b]
1	$AgCF_3CO_2$		DMAc	100	56
2	$AgCF_3CO_2$	TFA	DMAc	100	70
**3**	**$AgCF_3CO_2$**	**TfOH**	**DMAc**	**100**	**84**
4	$AgCF_3CO_2$	HCl	DMAc	100	67
5	$AgCF_3CO_2$	TsOH	DMAc	100	53
6	$AgCF_3CO_2$	HOAc	DMAc	100	49

[a] Reaction conditions: **1a** (0.2 mmol), **2a** (0.2 mmol), cat. (10 mol%), acid (30 mol%), solvent (1.5 mL), at 100 °C for 2 h under air atmosphere. [b] Isolated yield.

The reaction solvent was also examined, such as DMSO, MeCN, DMF, toluene, PhCl, THF, dioxane, and DCE (Table S5, entries 1-9). The desired product **3a** was obtained in 45% to 66% yields. The results showed DMAc was the best solvent (Table S5,

entry 1).

Table S5. Optimization for the solvent of reaction. [a]

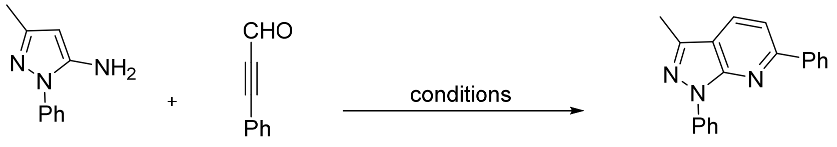

Entry	Cat.	Acid	Solvent	T(°C)	Yield(%)[b]
**1**	**$AgCF_3CO_2$**	**TfOH**	**DMAc**	**100**	**84**
2	$AgCF_3CO_2$	TfOH	DMSO	100	58
3	$AgCF_3CO_2$	TfOH	MeCN	100	53
4	$AgCF_3CO_2$	TfOH	DMF	100	60
5	$AgCF_3CO_2$	TfOH	Toluene	100	66
6	$AgCF_3CO_2$	TfOH	PhCl	100	34
7	$AgCF_3CO_2$	TfOH	THF	100	56
8	$AgCF_3CO_2$	TfOH	Dioxane	100	60
9	$AgCF_3CO_2$	TfOH	DCE	100	45

[a] Reaction conditions: 1a (0.2 mmol), 2a (0.2 mmol), cat. (10 mol%), acid (30 mol%), solvent (1.5 mL), at 100 °C for 2 h under air atmosphere. [b] Isolated yield.

Besides, the reaction temperature was also screened and 100 °C was found to be the best temperature (Table S6, entries 1-5).

Table S6. Optimization for the temperature of reaction. [a]

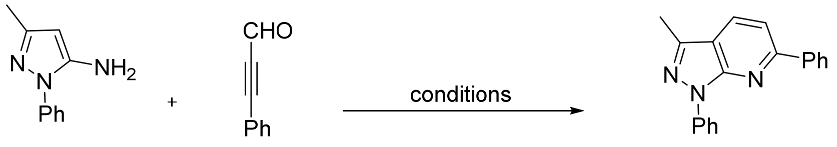

Entry	Cat.	Acid	Solvent	T(°C)	Yield(%)[b]
1	$AgCF_3CO_2$	TfOH	DMAc	60	60
2	$AgCF_3CO_2$	TfOH	DMAc	80	69
**3**	**$AgCF_3CO_2$**	**TfOH**	**DMAc**	**100**	**84**
4	$AgCF_3CO_2$	TfOH	DMAc	110	76
5	$AgCF_3CO_2$	TfOH	DMAc	120	70

[a] Reaction conditions: **1a** (0.2 mmol), **2a** (0.2 mmol), cat. (10 mol%), acid (30 mol%), solvent (1.5 mL), at different temperature for 2 h under air atmosphere. [b] Isolated yield.

Table S7. Optimization of the iodine-functionalized product. [a]

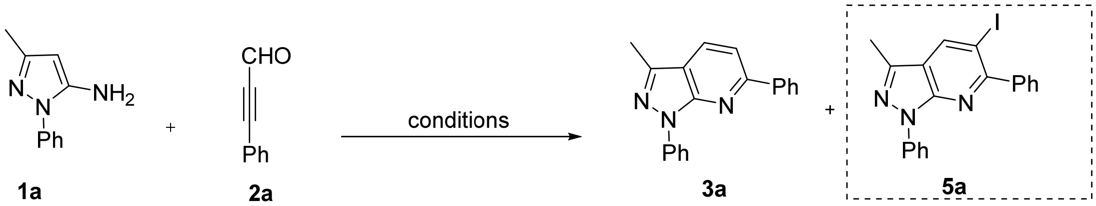

Entry	I source (eq.)	Acid (eq.)	Solvent	T (°C)	Yield (%) [c]	
					3a	5a

1 [b]	I_2 (1.0)	TfOH (0.3)	DMAc	100	46	30
2	I_2 (1.0)	TfOH (0.3)	DMAc	100	20	46
3	TBAI (1.0)	TfOH (0.3)	DMAc	100	22	34
4	NIS (1.0)	TfOH (0.3)	DMAc	100	30	40
5	NaI (1.0)	TfOH (0.3)	DMAc	100	26	43
6	KI (1.0)	TfOH (0.3)	DMAc	100	24	42
7	NH_4I (1.0)	TfOH (0.3)	DMAc	100	30	33
8	I_2 (1.5)	TfOH (0.3)	DMAc	100	20	50
9	I_2 (2.0)	TfOH (0.3)	DMAc	100	15	55
10	I_2 (2.0)	TfOH (0.5)	DMAc	100	15	58
11	I_2 (2.0)	TfOH (1.0)	DMAc	100	10	60
**12**	**I_2 (2.0)**	**TfOH (1.0)**	**DMSO**	**100**	**<5**	**68**
13	I_2 (2.0)	TfOH (1.0)	DMF	100	23	50
14	I_2 (2.0)	TfOH (1.0)	DCE	100	20	40
15	I_2 (2.0)	TfOH (1.0)	MeCN	100	30	44
16	I_2 (2.0)	TfOH (1.0)	Dioxane	100	33	43
17	I_2 (2.0)	TfOH (1.0)	Toluene	100	37	42
18	I_2 (2.0)	TfOH (1.0)	NMP	100	33	40
19	I_2 (2.0)	TfOH (1.0)	DMSO	90	25	50
20	I_2 (2.0)	TfOH (1.0)	DMSO	80	30	45
21	I_2 (2.0)	TfOH (1.0)	DMSO	70	35	22
22	I_2 (2.0)	TfOH (1.0)	DMSO	60	30	N.D.[d]

[a] Reaction conditions: **1a** (0.2 mmol), **2a** (0.2 mmol), I source, acid , solvent (2.0 mL), at shown temperature for 6 h under air atmosphere. [b] 10 mol% $AgCF_3CO_2$ was added [c] Isolated yield. [d] N.D. = not detected

We chose 3-methyl-1-phenyl-1*H*-pyrazol-5-amine (**1a**) and 3-phenylpropiolaldehyde (**2a**) as the model substrates for the optimization of the condition. Firstly, a series of iodine sources were screened under the selected conditions, including I_2, TBAI, NIS, NaI, KI and NH_4I (Table S7 entries 1-7). When I_2 was employed iodine source, the yield of **5a** was increased to 46% (Table S7, entry 2). Next, the amount of I_2 was examined and the results showed that 2.0 equivalent of I_2 provided **5a** in 60% yield (Table S7, entries 8-9). After screening the iodine sources, we turned our attention to selecting the amount of TfOH, the results showed that 1.0 equivalent of TfOH was the best choice, providing **5a** in 60% yield (Table S7, entries 9-12).Then, the reaction solvent was examined, such as DMSO, DMF, DCE, MeCN, dioxane, toluene and NMP (Table S7, entries 12-18). The results showed DMSO was the best solvent (Table 1, entry 12). Finally, the effect of reaction temperature was also screened. The experiment results showed that 100 °C was the best reaction temperature, giving the desired product **5a** in 68% yield (Table S7, entries 19-22).

Table S8. Optimization of the bromine-functionalized product. [a,b]

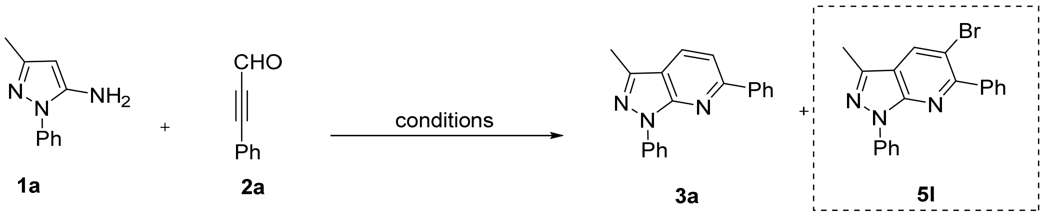

Entry	Br source (eq.)	Acid (eq.)	Solvent	T (°C)	Yield (%) [b]	
					3a	5l
1	NBS (2.0)	TfOH (1.0)	DMSO	100	trace	66
2	NBA (2.0)	TfOH (1.0)	DMSO	100	22	42
3	NBP (2.0)	TfOH (1.0)	DMSO	100	25	38
4	DBDMH (2.0)	TfOH (1.0)	DMSO	100	15	43

[a] Reaction conditions: **1a** (0.2 mmol), **2a** (0.2 mmol), Br source, acid , solvent (1.5 mL), at shown temperature for 6 h under air atmosphere. [b] Isolated yield. [c] NBA = *N*-Bromoacetamide. [d] NBP = *N*-(Bromomethyl)phthalimide. [e] DBDMH = 1,3-Dibromo-5,5-dimethylhydantoin.

The bromine sources also were screened, when NBS was used as external bromine source, giving the expected product **5l** in 66% yield (Table S8, entries 1-4).

3.2 Synthesis of 6, 8, 10 (adapted from literature) [50]

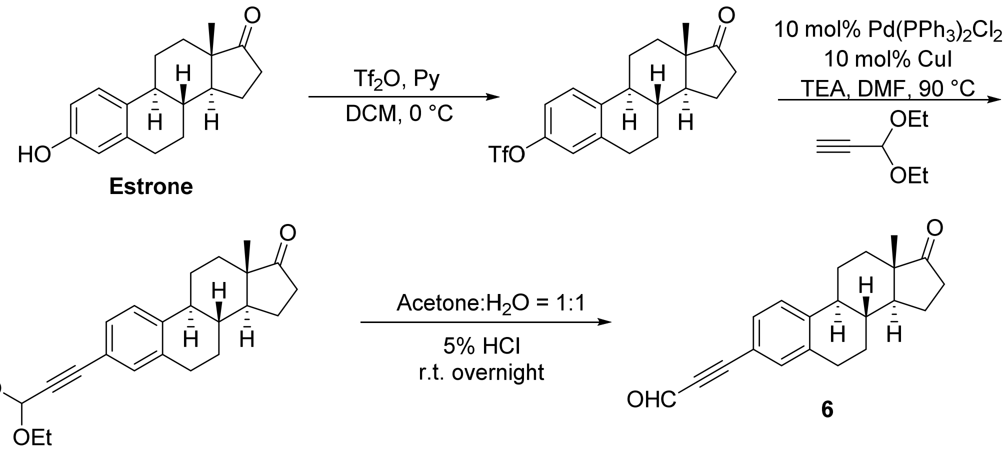

Scheme S4. Synthesis of **6** via Sonogashira coupling reaction and acetal hydrolysis reaction

Step 1: Estrone (1.0 equiv., 5 mmol) and pyridine (2.0 equiv., 10.0 mmol) was sequentially dissolved in 20 mL of dry DCM, following by dropwise addition of Tf_2O (1.2 equiv., 6.0 mmol) in 10 mL of dry DCM at 0 °C. After that, the mixture was warmed to rt, and stirred overnight. When the reaction completed, 10% HCl was added to the solution to quench the reaction, and then the mixture was extracted with DCM. The organic layer was washed with saturated $NaHCO_3$ and saturated brine, dried over anhydrous Na_2SO_4, filtered and concentrated in vacuo. Purification of the residue by column chromatography on silica gel gave the corresponding trifluoromethanesulfonate as a white solid.

Step 2: A mixture of trifluoromethanesulfonate (1.0 equiv., 4.5 mmol), 3,3-diethoxyprop-1-yne (1.25 equiv., 5.6 mmol), triethylamine (3.0 mL), and $Pd(PPh_3)_2Cl_2$ (10 mol%, 0.45 mmol), CuI (10 mol%, 0.45 mmol) was dissolved in 15

mL of DMF and stirred at 90 °C for 4 h under Ar. The reaction mixture was diluted with water (150 mL) and extracted with EtOAc (3 × 50 mL), the combined organic layers were washed with brine for three times (3 × 30 mL), dried by Na_2SO_4, and filtrated. The filtrate was evaporated. Chromatography of the residue on silica gel provided the corresponding product acetal.

Step 3: Acetal (1.0 equiv., 4.0 mmol) was dissolved in mixed solvents (20 mL) of acetone and H_2O (*v:v* = 1:1), then 5 equivalents of 5% HCl (12 mL, 20 mmol) was added to the solution, and the mixture was allowed to react overnight at room temperature. Upon completion of the reaction, acetone was removed under reduced pressure, after that the aqueous layer was extracted with DCM (3 × 20 mL), the combined organic layers were dried over anhydrous Na_2SO_4, and concentrated in vacuo. Purification of the residue by column chromatography gave **6** in overall 54% yield from Estrone. The experimental data of **6** match with those reported in the literature.[1]

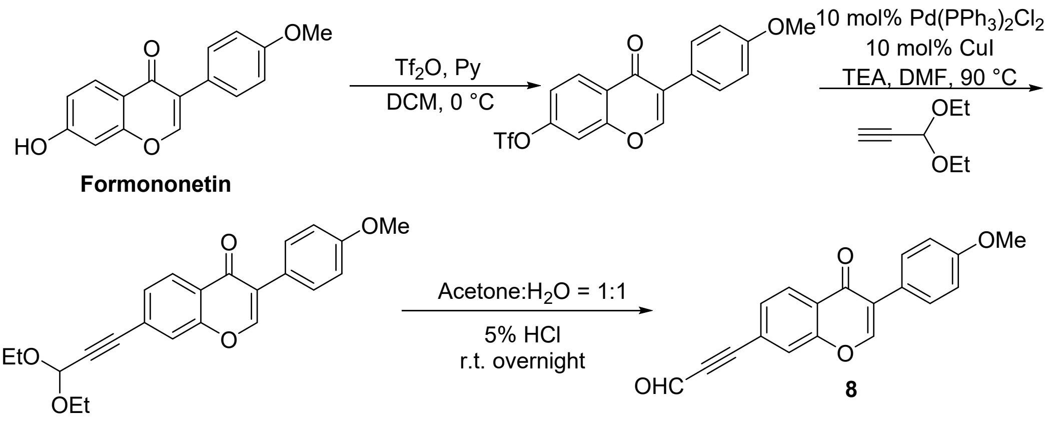

Scheme S5. Synthesis of **8** via Sonogashira coupling reaction and acetal hydrolysis reaction

Step 1: Formononetin (1.0 equiv., 5 mmol) and pyridine (2.0 equiv., 10.0 mmol) was sequentially dissolved in 20 mL of dry DCM, following by dropwise addition of Tf_2O (1.2 equiv., 6.0 mmol) in 10 mL of dry DCM at 0 °C. After that, the mixture was warmed to rt, and stirred overnight. When the reaction completed, 10% HCl was added to the solution to quench the reaction, and then the mixture was extracted with DCM. The organic layer was washed with saturated $NaHCO_3$ and saturated brine, dried over anhydrous Na_2SO_4, filtered and concentrated in vacuo. Purification of the residue by column chromatography on silica gel gave the corresponding trifluoromethanesulfonate as a yellow solid.

Step 2: A mixture of trifluoromethanesulfonate (1.0 equiv., 4.5 mmol), 3,3-diethoxyprop-1-yne (1.25 equiv., 5.6 mmol), triethylamine (3.0 mL), and $Pd(PPh_3)_2Cl_2$ (10 mol%, 0.45 mmol), CuI (10 mol%, 0.45 mmol) was dissolved in 15 mL of DMF and stirred at 90 °C for 4 h under Ar. The reaction mixture was diluted with water (150 mL) and extracted with EtOAc (3 × 50 mL), the combined organic layers were washed with brine for three times (3 × 30 mL), dried by Na_2SO_4, and

filtrated. The filtrate was evaporated. Chromatography of the residue on silica gel provided the corresponding product acetal.

Step 3: Acetal (1.0 equiv., 4.04 mmol) was dissolved in mixed solvents (20 mL) of acetone and H_2O (*v:v* = 1:1), then 5 equivalents of 5% HCl (12 mL, 20 mmol) was added to the solution, and the mixture was allowed to react overnight at room temperature. Upon completion of the reaction, acetone was removed under reduced pressure, after that the aqueous layer was extracted with DCM (3 × 20 mL), the combined organic layers were dried over anhydrous Na_2SO_4, and concentrated in vacuo. Purification of the residue by column chromatography gave **8** in overall 56% yield from formononetin.

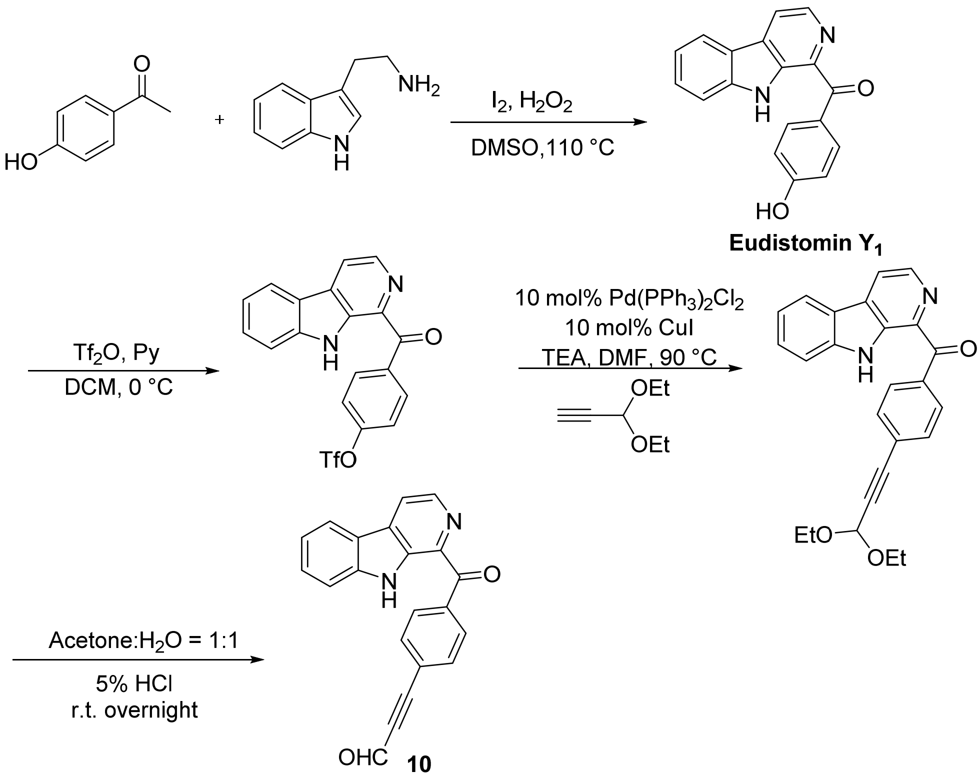

Scheme S6. Synthesis of **10** via tandem cyclization protocol, Sonogashira coupling reaction and acetal hydrolysis reaction

Step 1: Adapting a literature procedure [51], hydrogen peroxide (30% aqueous solution, 1.5 equiv.) was added to a mixture of acetophenone (1.0 equiv., 1 mmol), tryptamine (1.0 equiv., 1 mmol), and I_2 (0.8 equiv., 0.8 mmol) in DMSO (5 mL) at room temperature. The resulting mixture was stirred at 110 °C for 5 h during which it was monitored by TLC analysis. The solution was then cooled to RT, diluted with water (50 mL), and extracted with EtOAc three times (3 × 50 mL). The extract was washed with 10% $Na_2S_2O_3$ solution, dried over a hydrous Na_2SO_4, and concentrated under reduced pressure. The residue was purified by column chromatography on silica gel to afford the desired product eudistomin Y_1 as a yellow solid.

Step 2: Eudistomin Y_1 (1.0 equiv., 5 mmol) and pyridine (2.0 equiv., 10.0 mmol) was sequentially dissolved in 20 mL of dry DCM, following by dropwise addition of Tf_2O (1.2 equiv., 6.0 mmol) in 10 mL of dry DCM at 0 °C. After that, the mixture was warmed to rt, and stirred overnight. When the reaction completed, 10% HCl was

added to the solution to quench the reaction, and then the mixture was extracted with DCM. The organic layer was washed with saturated $NaHCO_3$ and saturated brine, dried over anhydrous Na_2SO_4, filtered and concentrated in vacuo. Purification of the residue by column chromatography on silica gel gave the corresponding trifluoromethanesulfonate as a yellow solid.

Step 3: A mixture of trifluoromethanesulfonate (1.0 equiv., 4.5 mmol), 3,3-diethoxyprop-1-yne (1.25 equiv., 5.6 mmol), triethylamine (3.0 mL), and $Pd(PPh_3)_2Cl_2$ (10 mol%, 0.45 mmol), CuI (10 mol%, 0.45 mmol) was dissolved in 15 mL of DMF and stirred at 90 °C for 4 h under Ar. The reaction mixture was diluted with water (150 mL) and extracted with EtOAc (3 × 50 mL), the combined organic layers were washed with brine for three times (3 × 30 mL), dried by Na_2SO_4, and filtrated. The filtrate was evaporated. Chromatography of the residue on silica gel provided the corresponding product acetal.

Step 4: Acetal (1.0 equiv., 4.0 mmol) was dissolved in mixed solvents (20 mL) of acetone and H_2O (*v:v* = 1:1), then 5 equivalents of 5% HCl (12 mL, 20 mmol) was added to the solution, and the mixture was allowed to react overnight at room temperature. Upon completion of the reaction, acetone was removed under reduced pressure, after that the aqueous layer was extracted with DCM (3 × 20 mL), the combined organic layers were dried over anhydrous Na_2SO_4, and concentrated in vacuo. Purification of the residue by column chromatography gave **10** in overall 55% yield from Eudistomin Y_1.

4. Characterization of Products

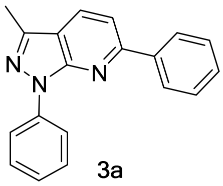

3a

3-Methyl-1,6-diphenyl-1*H*-pyrazolo[3,4-*b*]pyridine (3a), 48 mg, 84%, white solid, m.p. 126–127 °C. ^1H NMR (400 MHz, $CDCl_3$): δ (ppm) 8.46 – 8.40 (m, 2H), 8.20 – 8.15 (m, 2H), 8.08 (d, *J* = 8.0 Hz, 1H), 7.67 (d, *J* = 8.0 Hz, 1H), 7.54 (m, 4H), 7.49 – 7.44 (m, 1H), 7.32 – 7.25 (m, 1H), 2.67 (s, 3H). ^{13}C NMR (100 MHz, $CDCl_3$): δ (ppm) 156.7, 151.0, 142.5, 139.8, 139.2, 129.9, 129.4, 128.9, 128.7, 127.5, 125.1, 120.5, 115.8, 114.2, 12.5. HR-MS (ESI): calcd for $[M+H]^+$ $C_{19}H_{16}N_3$: 286.1339; found: 286.1339. The experimental data of **3a** match with those reported in the literature[52].

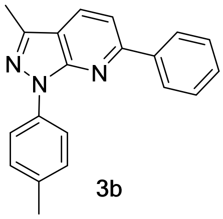

3b

3-Methyl-6-phenyl-1-(*p*-tolyl)-1*H*-pyrazolo[3,4-*b*]pyridine (3b), 47 mg, 78%, white solid, m.p. 132–133 °C. ^1H NMR (400 MHz, $CDCl_3$): δ (ppm) 8.29 – 8.24 (m, 2H), 8.20 – 8.15 (m, 2H), 8.07 (d, *J* = 8.3 Hz, 1H), 7.66 (d, *J* = 8.4 Hz, 1H), 7.55 – 7.49 (m,

2H), 7.49 – 7.42 (m, 1H), 7.37 – 7.31 (m, 2H), 2.66 (s, 3H), 2.42 (s, 3H). ^{13}C NMR (100 MHz, $CDCl_3$): δ (ppm) 156.5, 150.8, 142.1, 139.2, 137.3, 134.8, 129.8, 129.4, 129.3, 128.7, 127.5, 120.6, 115.5, 114.0, 21.0, 12.4. HR-MS (ESI): calcd for $[M+H]^+$ $C_{20}H_{18}N_3$: 300.1495; found: 300.1496.

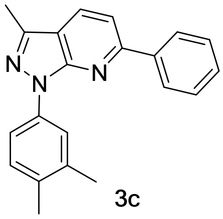

3c

1-(3,4-Dimethylphenyl)-3-methyl-6-phenyl-1*H*-pyrazolo[3,4-*b*]pyridine (3c), 47 mg, 75%, white solid, m.p. 152–153 °C. ^{1}H NMR (400 MHz, $CDCl_3$): δ (ppm) 8.20 – 8.13 (m, 3H), 8.12 – 8.05 (m, 2H), 7.66 (d, J = 8.4 Hz, 1H), 7.54 – 7.49 (m, 2H), 7.48 – 7.43 (m, 1H), 7.29 (d, J = 8.0 Hz, 1H), 2.66 (s, 3H), 2.39 (s, 3H), 2.33 (s, 3H). ^{13}C NMR (100 MHz, $CDCl_3$): δ (ppm) 156.5, 150.8, 142.0, 139.3, 137.5, 137.2, 133.6, 129.9, 129.8, 129.3, 128.7, 127.5, 121.9, 118.2, 115.5, 114.0, 20.0, 19.3, 12.4. HR-MS (ESI): calcd for $[M+H]^+$ $C_{21}H_{20}N_3$: 314.1651; found: 314.1653.

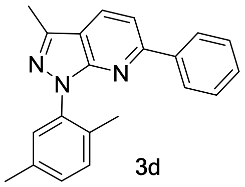

3d

1-(2,5-Dimethylphenyl)-3-methyl-6-phenyl-1*H*-pyrazolo[3,4-*b*]pyridine (3d), 48 mg, 77%, white solid, m.p. 154–155 °C. ^{1}H NMR (400 MHz, $CDCl_3$): δ (ppm) 8.10 (d, J = 8.0 Hz, 1H), 8.08 – 8.05 (m, 2H), 7.64 (d, J = 8.0 Hz, 1H), 7.47 – 7.37 (m, 3H), 7.31 – 7.26 (m, 2H), 7.19 (s, 1H), 2.67 (s, 3H), 2.38 (s, 3H), 2.23 (s, 3H). ^{13}C NMR (100 MHz, $CDCl_3$): δ (ppm) 156.8, 151.6, 142.0, 139.2, 137.3, 136.1, 132.1, 130.7, 129.8, 129.2, 129.1, 128.6, 128.2, 127.5, 114.1, 113.8, 20.8, 18.2, 12.5. HR-MS (ESI): calcd for $[M+H]^+$ $C_{21}H_{20}N_3$: 314.1651; found: 314.1545.

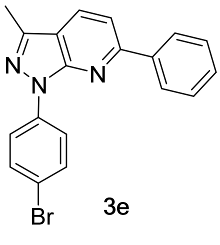

3e

1-**(4-Bromophenyl)-3-methyl-6-phenyl-1*H*-pyrazolo[3,4-*b*]pyridine (3e)**, 59 mg, 81%, white solid, m.p. 164–165 °C. ^{1}H NMR (400 MHz, $CDCl_3$): δ (ppm) 8.39 – 8.35 (m, 2H), 8.16 – 8.11 (m, 2H), 8.06 (d, J = 8.0 Hz, 1H), 7.66 (d, J = 8.0 Hz, 1H), 7.65 – 7.61 (m, 2H), 7.56 – 7.50 (m, 2H), 7.50 – 7.45 (m, 1H), 2.64 (s, 3H). ^{13}C NMR (100 MHz, $CDCl_3$): δ (ppm) 156.8, 150.9, 142.9, 138.9, 138.8, 131.9, 130.0, 129.5, 128.8, 127.4, 121.6, 118.0, 115.9, 114.4, 12.5. HR-MS (ESI): calcd for $[M+H]^+$ $C_{19}H_{15}BrN_3$: 364.0443; found: 364.0436.

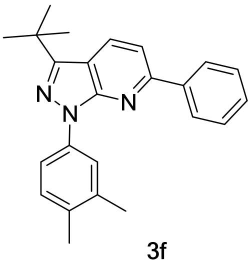

3f

3-(*tert*-Butyl)-1-(3,4-dimethylphenyl)-6-phenyl-1*H*-pyrazolo[3,4-*b*]pyridine (3f), 52 mg, 74%, white solid, m.p. 122–123 °C. ^{1}H NMR (400 MHz, $CDCl_3$): δ (ppm) 8.27 (d, J = 8.4 Hz, 1H), 8.20 – 8.17 (m, 2H), 8.17 – 8.14 (m, 2H), 7.63 (d, J = 8.5 Hz, 1H), 7.55 – 7.50 (m, 2H), 7.49 – 7.44 (m, 1H), 7.30 (d, J = 8.1 Hz, 1H), 2.41 (s, 3H), 2.33 (s, 3H), 1.60 (s, 9H). ^{13}C NMR (100 MHz, $CDCl_3$): δ (ppm) 155.7, 153.0, 151.3, 139.3, 137.7, 137.0, 133.4, 131.4, 129.8, 129.2, 122.1, 118.4, 113.5, 113.4, 34.2, 29.9, 20.1, 19.3. HR-MS (ESI): calcd for $[M+H]^+$ $C_{24}H_{26}N_3$: 356.2121; found: 356.2124.

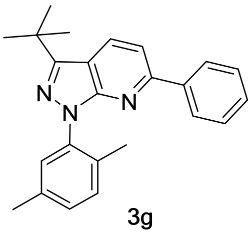

3g

3-(*tert*-Butyl)-1-(2,5-dimethylphenyl)-6-phenyl-1*H*-pyrazolo[3,4-*b*]pyridine (3g), 58 mg, 82 %, white solid, m.p. 145–147 °C. ^{1}H NMR (400 MHz, $CDCl_3$): δ (ppm) 8.29 (d, J = 8.5 Hz, 1H), 8.09 – 8.03 (m, 2H), 7.61 (d, J = 8.5 Hz, 1H), 7.48 – 7.35 (m, 4H), 7.28 (s, 1H), 7.16 (dd, J = 7.8, 1.4 Hz, 1H), 2.40 (s, 3H), 2.24 (s, 3H), 1.59 (s, 9H). ^{13}C NMR (100 MHz, $CDCl_3$): δ (ppm) 156.0, 153.2, 152.1, 139.3, 137.4, 135.9, 132.1, 131.4, 130.9, 129.1, 128.8, 128.6, 128.2, 127.4, 113.4, 112.0, 34.2, 30.1, 20.8, 18.4. HR-MS (ESI): calcd for $[M+H]^+$ $C_{24}H_{26}N_3$: 356.2121; found: 356.2123.

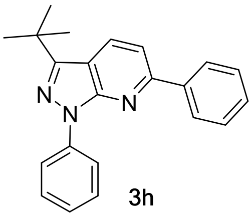

3h

3-(*tert*-Butyl)-1,6-diphenyl-1*H*-pyrazolo[3,4-*b*]pyridine (3h), 50 mg, 76%, white solid, m.p. 104–106 °C. ^{1}H NMR (400 MHz, $CDCl_3$): δ (ppm) 8.49 (dd, J = 8.7, 1.1 Hz, 2H), 8.28 (d, J = 8.4 Hz, 1H), 8.21 – 8.14 (m, 2H), 7.64 (d, J = 8.5 Hz, 1H), 7.54 (ddd, J = 12.7, 4.9, 2.3 Hz, 4H), 7.50 – 7.44 (m, 1H), 7.28 (dt, J = 7.6, 1.0 Hz, 1H), 1.60 (s, 9H). ^{13}C NMR (100 MHz, $CDCl_3$): δ (ppm) 155.9, 153.5, 151.6, 140.0, 139.3, 131.5, 129.3, 128.7, 127.5, 125.0, 120.6, 113.8, 34.3, 29.9. HR-MS (ESI): calcd for $[M+H]^+$ $C_{22}H_{22}N_3$: 328.1808; found: 328.1810.

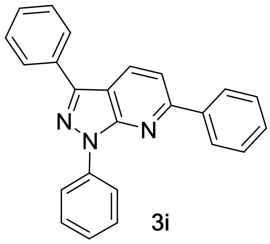

3i

1,3,6-Triphenyl-1*H*-pyrazolo[3,4-*b*]pyridine (3i), 51 mg, 74%, white solid, m.p. 160–161 °C. ^{1}H NMR (400 MHz, $CDCl_3$): *δ* (ppm) 8.55 (dd, *J* = 8.7, 1.0 Hz, 2H), 8.43 (d, *J* = 8.5 Hz, 1H), 8.23 – 8.17 (m, 2H), 8.08 (dd, *J* = 8.1, 1.1 Hz, 2H), 7.75 (d, *J* = 8.5 Hz, 1H), 7.59 (d, *J* = 7.5 Hz, 2H), 7.56 (d, *J* = 1.0 Hz, 2H), 7.55 – 7.52 (m, 2H), 7.51 – 7.46 (m, 2H), 7.37 – 7.31 (m, 1H). ^{13}C NMR (100 MHz, $CDCl_3$): *δ* (ppm) 156.7, 151.6, 144.1, 139.7, 139.0, 132.8, 131.0, 129.5, 128.9, 128.9, 128.8, 128.6, 127.5, 127.3, 125.6, 121.1, 115.2, 114.1. HR-MS (ESI): calcd for $[M+H]^+$ $C_{24}H_{18}N_3$: 348.1495; found: 348.1495.

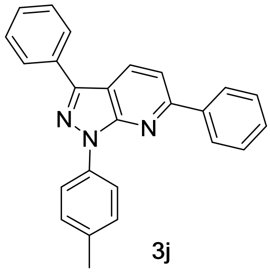

3j

3,6-Diphenyl-1-(*p*-tolyl)-1*H*-pyrazolo[3,4-*b*]pyridine (3j), 55 mg, 76%, white solid, m.p. 179–181 °C. ^{1}H NMR (400 MHz, $CDCl_3$): *δ* (ppm) 8.42 (dd, *J* = 8.5, 1.4 Hz, 1H), 8.40 – 8.36 (m, 2H), 8.19 (dd, *J* = 8.1, 1.1 Hz, 2H), 8.08 (dd, *J* = 8.0, 1.0 Hz, 2H), 7.74 (dd, *J* = 8.5, 1.3 Hz, 1H), 7.55 (q, *J* = 8.2, 7.8 Hz, 4H), 7.51 – 7.45 (m, 2H), 7.38 (d, *J* = 8.2 Hz, 2H), 2.45 (s, 3H). ^{13}C NMR (100 MHz, $CDCl_3$): *δ* (ppm) 156.5, 151.4, 143.7, 139.0, 137.3, 135.4, 132.9, 130.9, 129.5, 128.9, 128.7, 128.5, 127.5, 127.2, 121.1, 115.1, 113.9, 21.0. HR-MS (ESI): calcd for $[M+H]^+$ $C_{25}H_{20}N_3$: 362.1651; found: 362.1651.

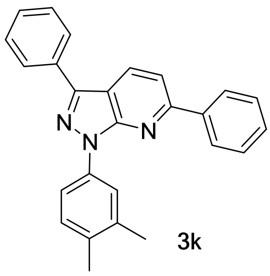

3k

1-(3,4-Dimethylphenyl)-3,6-diphenyl-1*H*-pyrazolo[3,4-*b*]pyridine (3k), 56 mg, 75%, white solid, m.p. 205–206 °C. ^{1}H NMR (400 MHz, $CDCl_3$): *δ* (ppm) 8.42 (d, *J* = 8.5 Hz, 1H), 8.25 (dd, *J* = 10.9, 2.7 Hz, 2H), 8.22 – 8.17 (m, 2H), 8.08 (d, *J* = 7.1 Hz, 2H), 7.74 (d, *J* = 8.5 Hz, 1H), 7.59 – 7.54 (m, 2H), 7.53 (d, *J* = 7.8 Hz, 2H), 7.50 – 7.44 (m, 2H), 7.33 (d, *J* = 8.1 Hz, 1H), 2.42 (s, 3H), 2.36 (s, 3H). ^{13}C NMR (100 MHz, $CDCl_3$): *δ* (ppm) 156.4, 151.4, 143.6, 139.0, 137.5, 137.2, 134.1, 133.0, 130.8, 129.9,

129.4, 128.9, 128.7, 128.5, 127.5, 127.2, 122.4, 118.7, 115.0, 113.8, 20.1, 19.3. HR-MS (ESI): calcd for $[M+H]^+$ $C_{26}H_{22}N_3$: 376.1808; found: 376.1808.

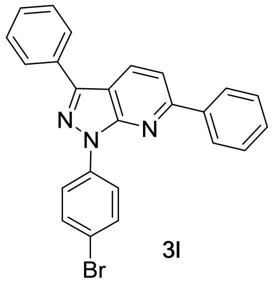

3l

1-(4-Bromophenyl)-3,6-diphenyl-1*H*-pyrazolo[3,4-*b*]pyridine (3l), 68 mg, 80%, white solid, m.p. 172–173 °C. ^{1}H NMR (400 MHz, $CDCl_3$): δ (ppm) 8.52 – 8.47 (m, 2H), 8.44 (d, J = 8.5 Hz, 1H), 8.20 – 8.15 (m, 2H), 8.09 – 8.04 (m, 2H), 7.77 (d, J = 8.5 Hz, 1H), 7.70 – 7.65 (m, 2H), 7.59 – 7.52 (m, 4H), 7.52 – 7.46 (m, 2H). ^{13}C NMR (100 MHz, $CDCl_3$): δ (ppm) 156.8, 151.6, 144.5, 138.8, 132.6, 131.9, 131.1, 129.7, 129.1, 128.9, 127.5, 127.3, 122.2, 118.7, 115.5, 114.3. HR-MS (ESI): calcd for $[M+H]^+$ $C_{24}H_{17}BrN_3$: 426.0600; found: 426.0602.

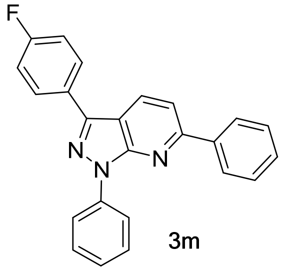

3m

3-(4-Fluorophenyl)-1,6-diphenyl-1*H*-pyrazolo[3,4-*b*]pyridine (3m), 50 mg, 68%, white solid, m.p. 164–165 °C. ^{1}H NMR (400 MHz, $CDCl_3$): δ (ppm) 8.51 (dd, J = 8.6, 1.0 Hz, 2H), 8.35 (d, J = 8.5 Hz, 1H), 8.18 (dd, J = 8.2, 1.2 Hz, 2H), 8.08 – 8.00 (m, 2H), 7.73 (d, J = 8.5 Hz, 1H), 7.60 – 7.46 (m, 5H), 7.34 (t, J = 7.4 Hz, 1H), 7.23 (d, J = 8.7 Hz, 2H). ^{13}C NMR (100 MHz, $CDCl_3$): δ (ppm) 164.30, 161.83, 156.73, 151.51, 143.12, 139.68, 138.87, 130.73, 129.61, 128.9 (d, J = 2.0 Hz), 128.8 (d, J = 8.0 Hz), 127.55, 125.72, 121.02, 115.9 (d, J = 22.0 Hz), 115.29, 113.86. HR-MS (ESI): calcd for $[M+H]^+$ $C_{24}H_{17}FN_3$: 366.1401; found: 366.1405.

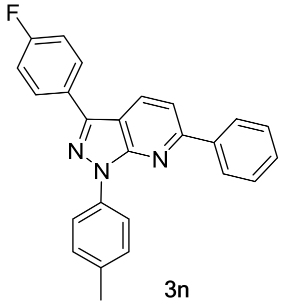

3n

3-(4-Fluorophenyl)-6-phenyl-1-(*p*-tolyl)-1*H*-pyrazolo[3,4-*b*]pyridine (3n), 53 mg, 70%, white solid, m.p. 188–189 °C. ^{1}H NMR (400 MHz, $CDCl_3$): δ (ppm) 8.41 – 8.33 (m, 3H), 8.21 – 8.17 (m, 2H), 8.08 – 8.02 (m, 2H), 7.75 (d, J = 8.5 Hz, 1H), 7.57 – 7.51 (m, 2H), 7.51 – 7.46 (m, 1H), 7.40 – 7.35 (m, 2H), 7.26 – 7.20 (m, 2H), 2.45 (s, 3H). ^{13}C NMR (100 MHz, $CDCl_3$): δ (ppm) 164.2, 161.7, 156.6, 151.3, 142.8, 138.93,

137.1, 135.5, 130.7, 129.5 (d, *J* = 3.0 Hz), 128.9 (d, *J* = 8.0 Hz), 128.8, 127.5, 121.1, 115.9 (d, *J* = 21.0 Hz), 115.2, 113.6, 21.0. HR-MS (ESI): calcd for $[M+H]^+$ $C_{25}H_{19}FN_3$: 380.1557; found: 380.1559.

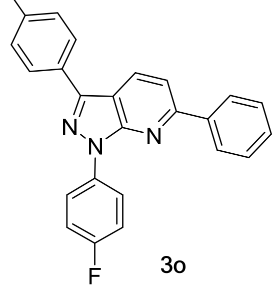

3o

1,3-bis(4-Fluorophenyl)-6-phenyl-1*H*-pyrazolo[3,4-*b*]pyridine (3o), 57 mg, 74%, white solid, m.p. 207–208 °C. ^{1}H NMR (400 MHz, $CDCl_3$): δ (ppm) 8.47 (dd, *J* = 8.7, 4.9 Hz, 2H), 8.38 (d, *J* = 8.6 Hz, 1H), 8.17 (d, *J* = 7.2 Hz, 2H), 8.08 – 7.98 (m, 2H), 7.76 (d, *J* = 8.5 Hz, 1H), 7.53 (dt, *J* = 13.9, 7.1 Hz, 3H), 7.26 (q, *J* = 5.7, 4.0 Hz, 4H). ^{13}C NMR (100 MHz, $CDCl_3$): δ (ppm) 164.3, 161.8 (d, *J* = 8.0 Hz), 159.3, 156.9, 151.3, 143.2, 138.8, 135.8 (d, *J* = 3.0 Hz), 130.9, 129.2, 129.0, 128.9 (d, *J* = 4.0 Hz), 127.5, 122.6 (d, *J* = 8.0 Hz), 116.1, 115.9 (d, *J* = 9.0 Hz), 115.5 (d, *J* = 18.0 Hz), 113.7, 99.9. HR-MS (ESI): calcd for $[M+H]^+$ $C_{24}H_{16}F_2N_3$: 384.1306; found: 384.1310.

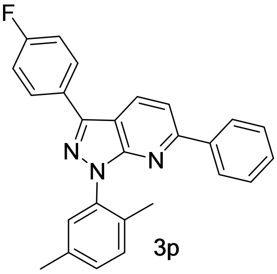

3p

1-(2,5-Dimethylphenyl)-3-(4-fluorophenyl)-6-phenyl-1*H*-pyrazolo[3,4-*b*]pyridine (3p), 55 mg, 70%, white solid, m.p. 163–164 °C. ^{1}H NMR (400 MHz, $CDCl_3$): δ (ppm) 8.41 (d, *J* = 8.5 Hz, 1H), 8.08 (dd, *J* = 8.1, 1.4 Hz, 2H), 8.06 – 8.01 (m, 2H), 7.74 (d, *J* = 8.5 Hz, 1H), 7.48 – 7.40 (m, 3H), 7.38 (s, 1H), 7.31 (d, *J* = 7.8 Hz, 1H), 7.21 (d, *J* = 8.6 Hz, 3H), 2.40 (s, 3H), 2.26 (s, 3H). ^{13}C NMR (100 MHz, $CDCl_3$): δ (ppm) 164.1, 161.6, 156.9, 152.1, 143.0, 138.8, 137.1, 136.2, 132.1, 130.8 (d, *J* = 20.0 Hz), 129.4 (d, *J* = 4.0 Hz), 128.8 (d, *J* = 8.0 Hz), 128.7, 128.3, 127.5, 115.9 (d, *J* = 22.0 Hz), 115.0, 112.2, 20.8, 18.3. HR-MS (ESI): calcd for $[M+H]^+$ $C_{26}H_{21}FN_3$: 394.1714; found: 394.1714.

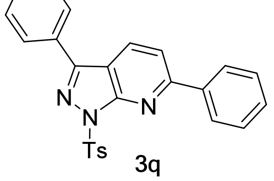

3q

3,6-Diphenyl-1-tosyl-1*H*-pyrazolo[3,4-*b*]pyridine (3q), 53 mg, 63%, white solid, m.p. 198–199 °C. ^{1}H NMR (400 MHz, $CDCl_3$): δ (ppm) 8.29 (d, *J* = 8.5 Hz, 1H), 8.27 – 8.21 (m, 2H), 8.19 (d, *J* = 8.4 Hz, 2H), 7.97 (dd, *J* = 8.0, 1.4 Hz, 2H), 7.81 (d, *J* =

8.5 Hz, 1H), 7.59 – 7.44 (m, 6H), 7.27 (d, *J* = 8.1 Hz, 2H), 2.35 (s, 3H). ^{13}C NMR (100 MHz, $CDCl_3$): δ (ppm) 157.7, 152.8, 148.4, 145.3, 138.0, 135.1, 131.3, 130.0, 129.7, 129.7, 128.9, 128.3, 127.7, 127.6, 116.7, 114.8, 21.6. HR-MS (ESI): calcd for $[M+H]^+$ $C_{25}H_{20}N_3O_2S$: 426.1270; found: 426.1273.

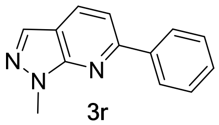
3r

Methyl-6-phenyl-1*H*-pyrazolo[3,4-*b*]pyridine (3r), 28 mg, 66%, white solid, m.p. 51–53 °C. ^{1}H NMR (400 MHz, $CDCl_3$): δ (ppm) 8.16 – 8.11 (m, 2H), 8.08 (d, *J* = 8.3 Hz, 1H), 8.00 (s, 1H), 7.59 (d, *J* = 8.3 Hz, 1H), 7.51 (t, *J* = 7.3 Hz, 2H), 7.45 (t, *J* = 7.2 Hz, 1H), 4.22 (s, 3H). ^{13}C NMR (100 MHz, $CDCl_3$): δ (ppm) 156.2, 150.4, 139.2, 131.4, 130.1, 129.1, 128.6, 127.3, 114.1, 113.9, 33.6. HR-MS (ESI): calcd for $[M+H]^+$ $C_{13}H_{12}N_3$: 210.1025; found: 210.1028.

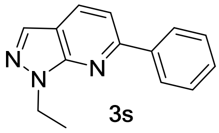
3s

Ethyl-6-phenyl-1*H*-pyrazolo[3,4-*b*]pyridine (3s), 30 mg, 68%, white solid, m.p. 67–69 °C. ^{1}H NMR (400 MHz, $CDCl_3$): δ (ppm) 8.16 – 8.12 (m, 2H), 8.08 (d, *J* = 8.4 Hz, 1H), 8.00 (s, 1H), 7.59 (d, *J* = 8.4 Hz, 1H), 7.54 – 7.48 (m, 2H), 7.48 – 7.42 (m, 1H), 4.66 (q, *J* = 7.3 Hz, 2H), 1.60 (t, *J* = 7.3 Hz, 3H). ^{13}C NMR (100 MHz, $CDCl_3$): δ (ppm) 156.2, 150.1, 139.4, 131.5, 130.2, 129.2, 128.7, 127.4, 114.3, 41.8, 14.9. HR-MS (ESI): calcd for $[M+H]^+$ $C_{14}H_{14}N_3$: 224.1182; found: 224.1180. The experimental data of **3s** match with those reported in the literature [53].

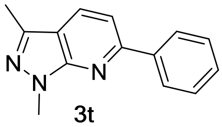
3t

1,3-Dimethyl-6-phenyl-1*H*-pyrazolo[3,4-*b*]pyridine (3t), 33 mg, 75%, white solid, m.p. 57–59 °C. ^{1}H NMR (400 MHz, $CDCl_3$): δ (ppm) 8.16 – 8.11 (m, 2H), 8.01 (d, *J* = 8.0 Hz, 1H), 7.55 (d, *J* = 8.0 Hz, 1H), 7.53 – 7.48 (m, 2H), 7.47 – 7.42 (m, 1H), 4.14 (s, 3H), 2.58 (s, 3H). ^{13}C NMR (100 MHz, $CDCl_3$): δ (ppm) 156.4, 151.4, 140.2, 139.5, 129.7, 129.2, 128.7, 127.4, 113.6, 113.4, 33.3, 12.4. HR-MS (ESI): calcd for $[M+H]^+$ $C_{14}H_{14}N_3$: 224.1182; found: 224.1184.

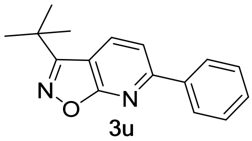
3u

3-(*tert*-Butyl)-6-phenylisoxazolo[5,4-*b*]pyridine (3u), 38 mg, 75%, white solid, m.p. 99–100 °C. ^{1}H NMR (400 MHz, $CDCl_3$): δ (ppm) 8.20 (d, *J* = 8.3 Hz, 1H), 8.14 – 8.10 (m, 2H), 7.75 (d, *J* = 8.3 Hz, 1H), 7.53 – 7.46 (m, 3H), 1.55 (s, 9H). ^{13}C NMR (100 MHz, $CDCl_3$): δ (ppm) 170.4, 165.7, 158.2, 137.8, 133.0, 130.0, 128.8, 127.6, 116.1, 110.1, 34.0, 28.9. HR-MS (ESI): calcd for $[M+H]^+$ $C_{16}H_{17}N_2O$: 253.1335;

found: 253.1338.

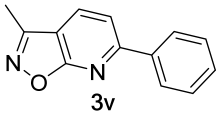

3v

3-Methyl-6-phenylisoxazolo[5,4-*b*]pyridine (3v), 30 mg, 71%, yellow solid, m.p. 172–173 °C. ^{1}H NMR (400 MHz, $CDCl_3$): δ (ppm) 8.13 (dt, J = 2.1, 1.3 Hz, 1H), 8.12 (dd, J = 2.2, 1.4 Hz, 1H), 8.05 (d, J = 8.2 Hz, 1H), 7.77 (d, J = 8.2 Hz, 1H), 7.53 – 7.50 (m, 1H), 7.50 – 7.45 (m, 2H), 2.60 (s, 3H). ^{13}C NMR (100 MHz, $CDCl_3$): δ (ppm) 170.0, 158.8, 155.3, 137.9, 131.6, 130.1, 128.9, 127.6, 116.4, 111.9, 10.8. HR-MS (ESI): calcd for $[M+H]^+$ $C_{13}H_{11}N_2O$: 211.0865; found: 211.0867. The experimental data of **3v** match with those reported in the literature [54].

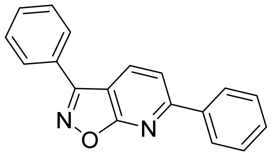

3w

3,6-Diphenylisoxazolo[5,4-*b*]pyridine (3w), 39 mg, 71%, white solid, m.p. 155–156 °C. ^{1}H NMR (400 MHz, $CDCl_3$): δ (ppm) 8.35 (dd, J = 8.3, 0.5 Hz, 1H), 8.21 – 8.13 (m, 2H), 8.02 – 7.96 (m, 2H), 7.88 – 7.83 (m, 1H), 7.61 – 7.56 (m, 3H), 7.56 – 7.48 (m, 3H). ^{13}C NMR (100 MHz, $CDCl_3$): δ (ppm) 170.7, 158.8, 157.2, 137.7, 132.6, 130.6, 130.2, 129.2, 128.9, 128.7, 127.7, 127.6, 117.1, 110.3. HR-MS (ESI): calcd for $[M+H]^+$ $C_{18}H_{13}N_2O$: 273.1022; found: 273.1023.

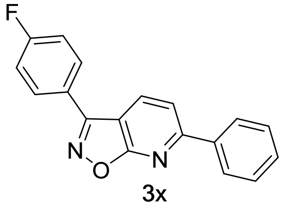

3x

3-(4-Fluorophenyl)-6-phenylisoxazolo[5,4-*b*]pyridine (3x), 41 mg, 70%, white solid, m.p. 204–205 °C. ^{1}H NMR (400 MHz, $CDCl_3$): δ (ppm) 8.32 (d, J = 8.3 Hz, 1H), 8.19 – 8.14 (m, 2H), 8.02 – 7.97 (m, 2H), 7.87 (d, J = 8.3 Hz, 1H), 7.57 – 7.48 (m, 3H), 7.31 – 7.26 (m, 2H). ^{13}C NMR (100 MHz, $CDCl_3$): δ (ppm) 170.7, 165.4, 162.9, 158.9, 156.3, 137.6, 132.4, 130.3, 129.6 (d, J = 8.0 Hz), 128.9, 127.7, 124.9 (d, J = 3.0 Hz), 117.2, 116.5 (d, J = 22.0 Hz), 110.1. HR-MS (ESI): calcd for $[M+H]^+$ $C_{18}H_{12}FN_2O$: 291.0928; found: 291.0929.

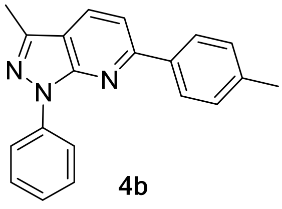

4b

3-Methyl-1-phenyl-6-(*p*-tolyl)-1*H*-pyrazolo[3,4-*b*]pyridine (4b), 47 mg, 78%, white solid, m.p. 100–101 °C. ^{1}H NMR (400 MHz, $CDCl_3$): δ (ppm) 8.48 – 8.42 (m, 2H), 8.09 – 8.05 (m, 2H), 8.02 (d, J = 8.3 Hz, 1H), 7.62 (d, J = 8.4 Hz, 1H), 7.59 – 7.50 (m, 2H), 7.33 (d, J = 8.4 Hz, 2H), 7.29 (m, 1H), 2.65 (s, 3H), 2.44 (s, 3H). ^{13}C NMR (100

MHz, $CDCl_3$): δ (ppm) 156.7, 151.0, 142.4, 139.8, 139.4, 136.4, 129.7, 129.4, 128.9, 127.4, 125.0, 120.4, 115.5, 114.0, 21.2, 12.4. HR-MS (ESI): calcd for $[M+H]^+$ $C_{20}H_{18}N_3$: 300.1495; found: 300.1497. The experimental data of **4b** match with those reported in the literature [52].

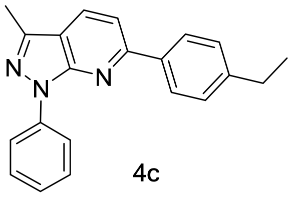

4c

6-(4-Ethylphenyl)-3-methyl-1-phenyl-1*H*-pyrazolo[3,4-*b*]pyridine (4c), 44 mg, 70%, white solid, m.p. 101–102 °C. ^1H NMR (400 MHz, $CDCl_3$): δ (ppm) 8.47 – 8.43 (m, 2H), 8.12 – 8.08 (m, 2H), 8.05 (d, *J* = 8.4 Hz, 1H), 7.64 (d, *J* = 8.4 Hz, 1H), 7.57 – 7.50 (m, 2H), 7.36 (d, *J* = 8.5 Hz, 2H), 7.29 (dt, *J* = 7.1, 1.0 Hz, 1H), 2.74 (q, *J* = 7.6 Hz, 2H), 2.66 (s, 3H), 1.31 (t, *J* = 7.6 Hz, 3H). ^{13}C NMR (100 MHz, $CDCl_3$): δ (ppm) 156.8, 151.1, 145.8, 142.5, 139.8, 136.7, 129.7, 128.9, 128.3, 127.5, 125.0, 120.4, 115.6, 114.1, 28.7, 15.4, 12.4. HR-MS (ESI): calcd for $[M+H]^+$ $C_{21}H_{20}N_3$: 314.1651; found: 314.1651.

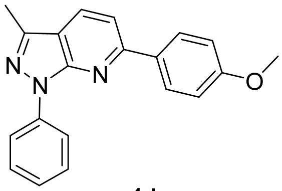

4d

6-(4-Methoxyphenyl)-3-methyl-1-phenyl-1*H*-pyrazolo[3,4-*b*]pyridine (4d), 40 mg, 64%, white solid, m.p. 117–118 °C. ^1H NMR (400 MHz, $CDCl_3$): δ (ppm) 8.46 – 8.40 (m, 2H), 8.16 – 8.10 (m, 2H), 8.01 (d, *J* = 8.4 Hz, 1H), 7.59 (d, *J* = 8.4 Hz, 1H), 7.57 – 7.49 (m, 2H), 7.31 – 7.26 (m, 1H), 7.07 – 7.00 (m, 2H), 3.89 (s, 3H), 2.64 (s, 3H). ^{13}C NMR (100 MHz, $CDCl_3$): δ (ppm) 160.8, 156.3, 151.1, 142.5, 139.8, 131.8, 129.7, 128.9, 128.8, 125.0, 120.5, 115.3, 114.1, 113.6, 55.3, 12.4. HR-MS (ESI): calcd for $[M+H]^+$ $C_{20}H_{18}N_3O$: 316.1444; found: 316.1445. The experimental data of **4d** match with those reported in the literature[52].

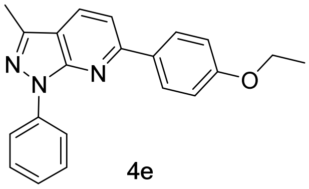

4e

6-(4-Ethoxyphenyl)-3-methyl-1-phenyl-1*H*-pyrazolo[3,4-*b*]pyridine (4e), 43 mg, 65%, white solid, m.p. 132–133 °C. ^1H NMR (400 MHz, $CDCl_3$): δ (ppm) 8.45 – 8.39 (m, 2H), 8.14 – 8.09 (m, 2H), 8.02 (d, *J* = 8.4 Hz, 1H), 7.60 (d, *J* = 8.4 Hz, 1H), 7.57 – 7.50 (m, 2H), 7.31 – 7.26 (m, 1H), 7.05 – 6.99 (m, 2H), 4.12 (q, *J* = 7.0 Hz, 2H), 2.64 (s, 3H), 1.46 (t, *J* = 7.0 Hz, 3H). ^{13}C NMR (100 MHz, $CDCl_3$): δ (ppm) 160.2, 156.4, 151.1, 142.5, 139.8, 131.6, 129.7, 128.9, 128.8, 125.0, 120.5, 115.2, 114.7, 113.6, 63.5, 14.8, 12.4. HR-MS (ESI): calcd for $[M+H]^+$ $C_{21}H_{20}N_3O$: 330.1600; found: 336.1602.

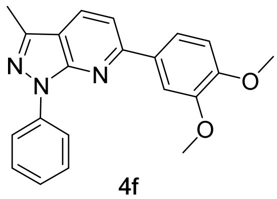

4f

6-(3,4-Dimethoxyphenyl)-3-methyl-1-phenyl-1*H*-pyrazolo[3,4-*b*]pyridine (4f), 46 mg, 67%, white solid, m.p. 119–120 °C. ^{1}H NMR (400 MHz, $CDCl_3$): δ (ppm) 8.43 (dd, J = 8.7, 1.1 Hz, 2H), 8.01 (d, J = 8.4 Hz, 1H), 7.84 (d, J = 2.0 Hz, 1H), 7.69 (dd, J = 8.4, 2.1 Hz, 1H), 7.60 (d, J = 8.4 Hz, 1H), 7.55 – 7.48 (m, 2H), 7.31 – 7.25 (m, 1H), 6.98 (d, J = 8.4 Hz, 1H), 4.02 (s, 3H), 3.96 (s, 3H), 2.64 (s, 3H). ^{13}C NMR (100 MHz, $CDCl_3$): δ (ppm) 156.1, 150.9, 150.4, 149.1, 142.5, 139.8, 132.0, 129.7, 128.8, 125.0, 120.4, 120.2, 115.3, 113.6, 111.0, 110.4, 55.9, 55.8, 12.4. HR-MS (ESI): calcd for $[M+H]^+$ $C_{21}H_{20}N_3O_2$: 346.1550; found: 346.1552.

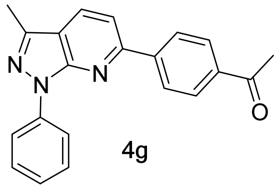

4g

(4-(3-Methyl-1-phenyl-1*H*-pyrazolo[3,4-*b*]pyridin-6-yl)phenyl)ethan-1-one (4g), 53 mg, 81%, white solid,m.p. 149–151 °C. ^{1}H NMR (400 MHz, $CDCl_3$): δ (ppm) 8.42 – 8.38 (m, 2H), 8.25 – 8.21 (m, 2H), 8.08 (dt, J = 8.5, 1.6 Hz, 3H), 7.67 (d, J = 8.3 Hz, 1H), 7.57 – 7.51 (m, 2H), 7.29 (m, 1H), 2.66 (s, 6H). ^{13}C NMR (100 MHz, $CDCl_3$): δ (ppm) 197.6, 155.0, 150.8, 143.3, 142.5, 139.6, 137.3, 130.1, 128.9, 128.7, 127.5, 125.3, 120.4, 116.2, 114.4, 26.7, 12.4. HR-MS (ESI): calcd for $[M+H]^+$ $C_{21}H_{18}N_3O$: 328.1444; found: 328.1447.

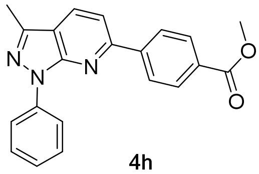

4h

Methyl 4-(3-methyl-1-phenyl-1*H*-pyrazolo[3,4-*b*]pyridin-6-yl)benzoate (4h), 46 mg, 67%, white solid, m.p. 139–140 °C. ^{1}H NMR (400 MHz, $CDCl_3$): δ (ppm) 8.44 – 8.35 (m, 2H), 8.22 – 8.18 (m, 2H), 8.17 – 8.13 (m, 2H), 8.05 (d, J = 8.3 Hz, 1H), 7.65 (d, J = 8.3 Hz, 1H), 7.58 – 7.50 (m, 2H), 7.33 – 7.26 (m, 1H), 3.96 (s, 3H), 2.65 (s, 3H). ^{13}C NMR (100 MHz, $CDCl_3$): δ (ppm) 166.7, 155.1, 150.8, 143.2, 142.5, 139.6, 130.6, 130.0, 129.9, 128.9, 127.3, 125.3, 120.4, 116.1, 114.4, 52.1, 12.4. HR-MS (ESI): calcd for $[M+H]^+$ $C_{21}H_{18}N_3O_2$: 344.1393; found: 344.1393.

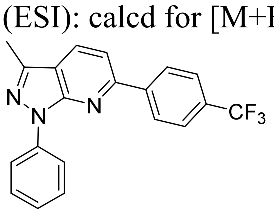

4i

Methyl-1-phenyl-6-(4-(trifluoromethyl)phenyl)-1*H*-pyrazolo[3,4-*b*]pyridine (4i), 47 mg, 66%, white solid,m.p. 147–148 °C. ^{1}H NMR (400 MHz, $CDCl_3$): δ (ppm) 8.41

– 8.37 (m, 2H), 8.27 – 8.22 (m, 2H), 8.09 (d, *J* = 8.3 Hz, 1H), 7.76 (d, *J* = 8.2 Hz, 2H), 7.65 (d, *J* = 8.3 Hz, 1H), 7.57 – 7.51 (m, 2H), 7.30 (m, 1H), 2.67 (s, 3H). ^{13}C NMR (100 MHz, $CDCl_3$): δ (ppm) 154.9, 150.8, 142.5, 139.6, 130.2, 128.9, 127.7, 125.6 (q, *J* = 4.0 MHz), 125.3, 120.5, 116.3, 114.3, 12.4. HR-MS (ESI): calcd for $[M+H]^+$ $C_{20}H_{15}F_3N_3$: 354.1212; found: 354.1212. The experimental data of **4i** match with those reported in the literature [55].

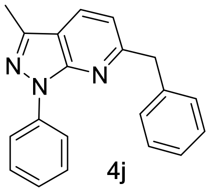

4j

6-Benzyl-3-methyl-1-phenyl-1*H*-pyrazolo[3,4-*b*]pyridine (4j), 40 mg, 67%, white solid, m.p. 71–72 °C. ^{1}H NMR (400 MHz, $CDCl_3$): δ (ppm) 8.43 – 8.29 (m, 2H), 7.90 (d, *J* = 8.2 Hz, 1H), 7.54 – 7.45 (m, 2H), 7.38 – 7.30 (m, 4H), 7.29 – 7.23 (m, 2H), 7.01 (d, *J* = 8.2 Hz, 1H), 4.30 (s, 2H), 2.61 (s, 3H). ^{13}C NMR (100 MHz, $CDCl_3$): δ (ppm) 160.9, 150.6, 142.4, 139.7, 139.3, 129.5, 129.1, 128.8, 128.4, 126.3, 125.1, 120.4, 116.8, 115.1, 45.0, 12.3. HR-MS (ESI): calcd for $[M+H]^+$ $C_{19}H_{15}BrN_3$: 300.1495; found: 300.1495.

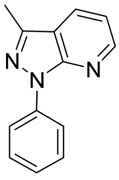

4k

3-Methyl-1-phenyl-1*H*-pyrazolo[3,4-*b*]pyridine (4k), 31 mg, 73%, yellow solid, m.p. 53–54 °C. ^{1}H NMR (400 MHz, $CDCl_3$): δ (ppm) 8.60 (dd, *J* = 4.6, 1.6 Hz, 1H), 8.29 – 8.21 (m, 2H), 8.03 (dd, *J* = 8.0, 1.6 Hz, 1H), 7.55 – 7.42 (m, 2H), 7.31 – 7.25 (m, 1H), 7.16 (dd, *J* = 8.0, 4.6 Hz, 1H), 2.65 (s, 3H). ^{13}C NMR (100 MHz, $CDCl_3$): δ (ppm) 150.5, 148.8, 142.4, 139.4, 129.4, 128.9, 125.4, 120.8, 116.9, 116.7, 12.3. HR-MS (ESI): calcd for $[M+H]^+$ $C_{13}H_{12}N_3$: 210.1025; found: 210.1025.

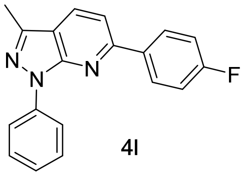

4l

(4-Fluorophenyl)-3-methyl-1-phenyl-1*H*-pyrazolo[3,4-*b*]pyridine (4l), 44 mg, 73%, white solid, m.p. 128–129 °C. ^{1}H NMR (400 MHz, $CDCl_3$): δ (ppm) 8.44 – 8.35 (m, 2H), 8.13 (m, 2H), 8.04 (d, *J* = 8.3 Hz, 1H), 7.58 (d, *J* = 8.4 Hz, 1H), 7.57 – 7.50 (m, 2H), 7.32 – 7.26 (m, 1H), 7.23 – 7.15 (m, 2H), 2.65 (s, 3H). ^{13}C NMR (100 MHz, $CDCl_3$): δ (ppm) 165.0, 162.5, 155.5, 150.9, 142.5, 139.7, 135.3 (d, *J* = 3.0 MHz), 130.0, 129.3 (d, *J* = 9.0 MHz), 128.9, 125.2, 120.5, 115.7 (d, *J* = 21.0 MHz), 113.8, 12.4. HR-MS (ESI): calcd for $[M+H]^+$ $C_{19}H_{15}FN_3$: 304.1244; found: 304.1244.

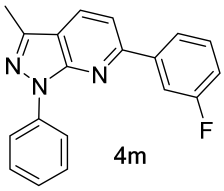

4m

(3-**Fluorophenyl)-3-methyl-1-phenyl-1*H*-pyrazolo[3,4-*b*]pyridine (4m)**, 41 mg, 67%, white solid, m.p. 117–118 °C. ^{1}H NMR (400 MHz, $CDCl_3$): δ (ppm) 8.43 – 8.36 (m, 2H), 8.05 (d, J = 8.3 Hz, 1H), 7.93 – 7.85 (m, 2H), 7.60 (d, J = 8.3 Hz, 1H), 7.58 – 7.51 (m, 2H), 7.46 (td, J = 8.0, 6.1 Hz, 1H), 7.30 (tt, J = 7.7, 1.1 Hz, 1H), 7.15 (m, 1H), 2.65 (s, 3H). ^{13}C NMR (100 MHz, $CDCl_3$): δ (ppm) 164.4, 162.0, 155.1 (d, J = 3.0 MHz), 150.8, 142.5, 141.5 (d, J = 8.0 MHz), 139.6, 130.2 (d, J = 8.0 MHz), 130.0, 128.9, 125.3, 123.0 (d, J = 3.0 MHz), 120.5, 116.2, 116.1 (d, J = 4.0 MHz), 114.3 (d, J = 23.0 MHz), 114.1, 12.4. HR-MS (ESI): calcd for $[M+H]^+$ $C_{19}H_{15}FN_3$: 304.1244; found: 304.1244.

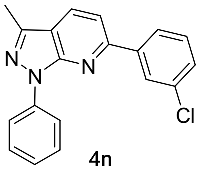

4n

6-(3-Chlorophenyl)-3-methyl-1-phenyl-1*H*-pyrazolo[3,4-*b*]pyridine (4n), 46 mg, 72%, white solid, m.p. 124–125 °C. ^{1}H NMR (400 MHz, $CDCl_3$): δ (ppm) 8.38 (d, J = 7.7 Hz, 2H), 8.18 – 7.97 (m, 3H), 7.59 (d, J = 8.3 Hz, 1H), 7.54 (t, J = 7.9 Hz, 2H), 7.47 – 7.39 (m, 2H), 7.30 (t, J = 7.3 Hz, 1H), 2.65 (s, 3H). ^{13}C NMR (100 MHz, $CDCl_3$): δ (ppm) 155.0, 150.8, 142.5, 141.0, 139.6, 134.8, 130.0, 129.9, 129.2, 128.9, 127.6, 125.6, 125.3, 120.5, 116.1, 114.1, 12.4. HR-MS (ESI): calcd for $[M+H]^+$ $C_{19}H_{15}ClN_3$: 320.0949; found: 320.0951.

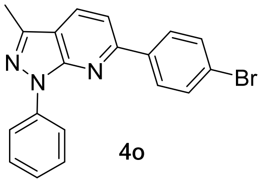

4o

(4-Bromophenyl)-3-methyl-1-phenyl-1*H*-pyrazolo[3,4-*b*]pyridine (4o), 52 mg, 71%, yellow solid, m.p. 127–128 °C. ^{1}H NMR (400 MHz, $CDCl_3$): δ (ppm) 8.41 – 8.36 (m, 2H), 8.04 – 7.98 (m, 3H), 7.65 – 7.60 (m, 2H), 7.57 (d, J = 8.4 Hz, 1H), 7.55 – 7.50 (m, 2H), 7.32 – 7.26 (m, 1H), 2.64 (s, 3H). ^{13}C NMR (100 MHz, $CDCl_3$): δ (ppm) 155.3, 150.8, 142.5, 139.6, 138.0, 131.8, 130.0, 128.9, 128.9, 125.2, 123.9, 120.4, 115.9, 113.8, 12.4. HR-MS (ESI): calcd for $[M+H]^+$ $C_{19}H_{15}BrN_3$: 364.0443; found: 364.0436.

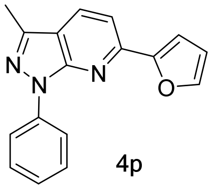

4p

6-(Furan-2-yl)-3-methyl-1-phenyl-1*H*-pyrazolo[3,4-*b*]pyridine (4p), 39 mg, 70%, yellow solid, m.p. 108–109 °C.. ^{1}H NMR (400 MHz, $CDCl_3$): *δ* (ppm) 8.43 – 8.34 (m, 2H), 8.02 (dd, *J* = 8.3, 0.7 Hz, 1H), 7.62 (dd, *J* = 8.3, 0.6 Hz, 1H), 7.58 (dd, *J* = 1.7, 0.8 Hz, 1H), 7.55 – 7.49 (m, 2H), 7.30 – 7.26 (m, 1H), 7.23 (dd, *J* = 3.4, 0.8 Hz, 1H), 6.58 (dd, *J* = 3.4, 1.8 Hz, 1H), 2.63 (s, 3H). ^{13}C NMR (100 MHz, $CDCl_3$): *δ* (ppm) 153.9, 150.6, 148.3, 143.7, 142.6, 139.7, 129.8, 128.9, 125.1, 120.4, 115.7, 112.5, 112.2, 109.9, 12.4. HR-MS (ESI): calcd for $[M+H]^+$ $C_{17}H_{14}N_3O$: 276.1131; found: 276.1131.

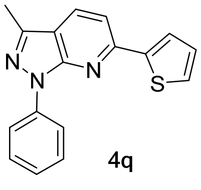

4q

3-Methyl-1-phenyl-6-(thiophen-2-yl)-1*H*-pyrazolo[3,4-*b*]pyridine (4q), 42 mg, 72%, yellow solid, m.p. 91–92 °C. ^{1}H NMR (400 MHz, $CDCl_3$): *δ* (ppm) 8.44 – 8.40 (m, 2H), 7.96 (d, *J* = 8.3 Hz, 1H), 7.70 (dd, *J* = 3.7, 1.1 Hz, 1H), 7.56 – 7.50 (m, 3H), 7.44 (dd, *J* = 5.1, 1.1 Hz, 1H), 7.30 – 7.25 (m, 1H), 7.13 (dd, *J* = 5.0, 3.7 Hz, 1H), 2.62 (s, 3H). ^{13}C NMR (100 MHz, $CDCl_3$): *δ* (ppm) 151.6, 150.4, 145.1, 142.6, 139.7, 129.8, 128.8, 128.3, 128.0, 125.7, 125.0, 120.2, 115.6, 112.8, 12.4. HR-MS (ESI): calcd for $[M+H]^+$ $C_{17}H_{14}N_3S$: 292.0902; found: 292.0904.

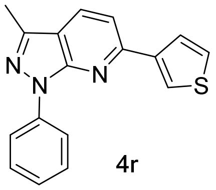

4r

3-Methyl-1-phenyl-6-(thiophen-3-yl)-1*H*-pyrazolo[3,4-*b*]pyridine (4r), 39 mg, 67%, yellow solid, m.p. 121–122 °C. ^{1}H NMR (400 MHz, $CDCl_3$): *δ* (ppm) 8.43 – 8.38 (m, 2H), 8.02 (dd, *J* = 3.0, 1.3 Hz, 1H), 7.99 (d, *J* = 8.3 Hz, 1H), 7.82 (dd, *J* = 5.1, 1.3 Hz, 1H), 7.56 – 7.50 (m, 3H), 7.42 (dd, *J* = 5.0, 3.0 Hz, 1H), 7.31 – 7.26 (m, 1H), 2.63 (s, 3H). ^{13}C NMR (100 MHz, $CDCl_3$): *δ* (ppm) 152.5, 150.8, 142.5, 142.2, 139.7, 129.8, 128.8, 126.7, 126.2, 125.1, 124.6, 120.4, 115.4, 114.2, 12.4. HR-MS (ESI): calcd for $[M+H]^+$ $C_{17}H_{14}N_3S$: 292.0902; found: 292.0902.

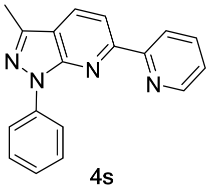

4s

3-Methyl-1-phenyl-6-(pyridin-2-yl)-1*H*-pyrazolo[3,4-*b*]pyridine (4s), 37 mg, 65%, white solid, m.p. 103–104 °C. ^{1}H NMR (400 MHz, $CDCl_3$): *δ* (ppm) 8.75 – 8.70 (m, 1H), 8.58 (d, *J* = 8.0 Hz, 1H), 8.47 – 8.35 (m, 3H), 8.14 (d, *J* = 8.4 Hz, 1H), 7.88 (td, *J* = 7.9, 1.8 Hz, 1H), 7.55 (td, *J* = 7.5, 1.9 Hz, 2H), 7.36 (ddd, *J* = 7.5, 4.8, 1.1 Hz, 1H), 7.34 – 7.27 (m, 1H), 2.67 (s, 3H). ^{13}C NMR (100 MHz, $CDCl_3$): *δ* (ppm) 155.8, 154.9, 150.6, 148.8, 142.6, 139.7, 137.2, 130.0, 128.9, 125.2, 124.0, 121.8, 120.5,

117.1, 114.7, 12.4. HR-MS (ESI): calcd for $[M+H]^+$ $C_{18}H_{15}N_4$: 287.1291; found: 287.1293.

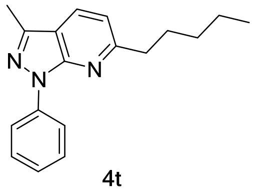

4t

3-Methyl-6-pentyl-1-phenyl-1*H*-pyrazolo[3,4-*b*]pyridine (4t), 31 mg, 55%, yellow liquid. ^{1}H NMR (400 MHz, $CDCl_3$): δ (ppm) 8.40 – 8.36 (m, 2H), 7.92 – 7.87 (m, 1H), 7.53 – 7.47 (m, 2H), 7.28 – 7.23 (m, 1H), 7.02 (d, J = 8.1 Hz, 1H), 2.99 – 2.90 (m, 2H), 2.62 (s, 3H), 1.85 (p, J = 7.4 Hz, 2H), 1.40 (dq, J = 7.1, 3.7 Hz, 4H), 0.97 – 0.91 (m, 3H). ^{13}C NMR (100 MHz, $CDCl_3$): δ (ppm) 162.8, 150.8, 142.3, 139.8, 129.1, 128.8, 124.9, 120.4, 116.7, 114.9, 38.5, 31.5, 29.3, 22.5, 14.0, 12.4. HR-MS (ESI): calcd for $[M+H]^+$ $C_{18}H_{22}N_3$: 280.1808; found: 280.1810.

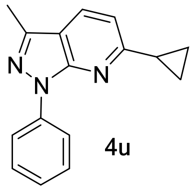

4u

6-Cyclopropyl-3-methyl-1-phenyl-1*H*-pyrazolo[3,4-*b*]pyridine (4u), 25 mg, 50%, white liquid. ^{1}H NMR (400 MHz, $CDCl_3$): δ (ppm) 8.37 – 8.28 (m, 2H), 7.84 (d, J = 8.2 Hz, 1H), 7.51 – 7.44 (m, 2H), 7.24 (dt, J = 7.1, 1.0 Hz, 1H), 7.05 (d, J = 8.2 Hz, 1H), 2.60 (s, 3H), 2.19 (tt, J = 8.1, 4.7 Hz, 1H), 1.20 (dt, J = 4.7, 3.1 Hz, 2H), 1.10 – 1.06 (m, 2H). ^{13}C NMR (100 MHz, $CDCl_3$): δ (ppm) 163.6, 151.0, 142.4, 139.9, 128.8, 124.8, 120.1, 115.8, 114.9, 17.7, 12.4, 11.1. HR-MS (ESI): calcd for $[M+H]^+$ $C_{16}H_{16}N_3$: 250.1339; found: 250.1338.

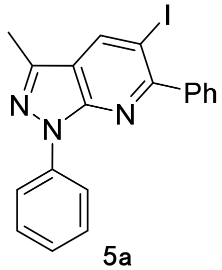

5a

5-Iodo-3-methyl-1,6-diphenyl-1*H*-pyrazolo[3,4-*b*]pyridine (5a), 55 mg, 68%, white solid, m.p. 117–118 °C. ^{1}H NMR (400 MHz, $CDCl_3$): δ (ppm) 8.61 (s, 1H), 8.34 – 8.25 (m, 2H), 7.72 – 7.65 (m, 2H), 7.49 (dp, J = 7.7, 1.9 Hz, 3H), 7.47 – 7.43 (m, 2H), 7.26 – 7.20 (m, 1H), 2.65 (s, 3H). ^{13}C NMR (125 MHz, $CDCl_3$): δ (ppm) 159.6, 149.9, 142.2, 141.4, 140.5, 139.4, 129.7, 129.0, 128.8, 127.8, 125.5, 120.4, 118.2, 84.1, 12.5. HR-MS (ESI): calcd for $[M+H]^+$ $C_{19}H_{15}IN_3$: 412.0305; found: 412.0305.

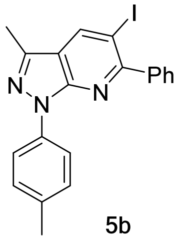

5b

5-Iodo-3-methyl-6-phenyl-1-(*p*-tolyl)-1*H*-pyrazolo[3,4-*b*]pyridine (5b), 51 mg, 60%, white solid, m.p. 137–138 °C. ^{1}H NMR (400 MHz, $CDCl_3$): δ (ppm) 8.60 (s, 1H), 8.18 – 8.07 (m, 2H), 7.72 – 7.63 (m, 2H), 7.51 – 7.45 (m, 3H), 7.26 (d, J = 0.9 Hz, 2H), 2.64 (s, 3H), 2.37 (s, 3H). ^{13}C NMR (125 MHz, $CDCl_3$): δ (ppm) 159.5, 149.7, 142.3, 141.0, 140.5, 136.9, 135.2, 129.7, 129.5, 128.7, 127.7, 120.5, 118.0, 83.9, 20.9, 12.4. HR-MS (ESI): calcd for $[M+H]^+$ $C_{20}H_{17}IN_3$: 426.0462; found: 425.0462.

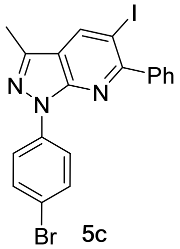

5c

1-(4-Bromophenyl)-5-iodo-3-methyl-6-phenyl-1*H*-pyrazolo[3,4-*b*]pyridine (5c), 64 mg, 66%, white solid, m.p. 204–205 °C. ^{1}H NMR (400 MHz, $CDCl_3$): δ (ppm) 8.61 (s, 1H), 8.31 – 8.16 (m, 2H), 7.70 – 7.63 (m, 2H), 7.58 – 7.54 (m, 2H), 7.52 – 7.47 (m, 3H), 2.64 (s, 3H). ^{13}C NMR (125 MHz, $CDCl_3$): δ (ppm) 159.8, 149.9, 142.1, 141.8, 140.7, 138.5, 132.0, 129.7, 128.9, 127.8, 121.6, 118.4, 118.4, 84.4, 12.5. HR-MS (ESI): calcd for $[M+H]^+$ $C_{19}H_{14}BrIN_3$: 489.9410; found: 489.9411.

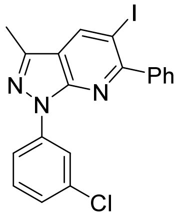

5d

1-(3-Chlorophenyl)-5-iodo-3-methyl-6-phenyl-1*H*-pyrazolo[3,4-*b*]pyridine (5d), 58 mg, 65%, white solid, m.p. 187–188 °C. ^{1}H NMR (400 MHz, $CDCl_3$): δ (ppm) 8.61 (s, 1H), 8.40 – 8.29 (m, 2H), 7.73 – 7.63 (m, 2H), 7.50 (qd, J = 4.9, 1.8 Hz, 3H), 7.40 – 7.33 (m, 1H), 7.23 – 7.17 (m, 1H), 2.64 (s, 3H). ^{13}C NMR (125 MHz, $CDCl_3$): δ (ppm) 159.8, 150.0, 142.0, 140.7, 140.4, 134.7, 130.0, 129.7, 128.9, 127.8, 125.3, 120.1, 118.4, 118.0, 84.5, 12.5. HR-MS (ESI): calcd for $[M+H]^+$ $C_{19}H_{14}ClIN_3$: 445.9915; found: 445.9915.

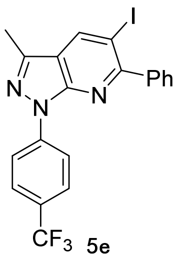

5e

5-Iodo-3-methyl-6-phenyl-1-(4-(trifluoromethyl)phenyl)-1*H*-pyrazolo[3,4-*b*]pyridine (5e), 63 mg, 66%, white solid, m.p. 169–170 °C. ^{1}H NMR (400 MHz, $CDCl_3$): δ (ppm) 8.63 (s, 1H), 8.57 – 8.48 (m, 2H), 7.72 – 7.69 (m, 2H), 7.68 (dd, J = 1.7, 0.7 Hz, 1H), 7.67 (q, J = 1.3 Hz, 1H), 7.54 – 7.48 (m, 3H), 2.66 (s, 3H). ^{13}C NMR (125 MHz, $CDCl_3$): δ (ppm) 160.0, 150.3, 142.5, 142.0, 140.7, 129.6, 128.9, 127.9, 126.2, 126.2 (q, J = 3.0 MHz), 118.6, 84.8, 12.5. HR-MS (ESI): calcd for $[M+H]^+$ $C_{20}H_{14}F_3IN_3$: 480.0179; found: 480.0179.

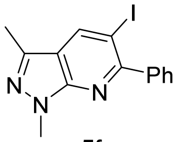

5f

5-Iodo-1,3-dimethyl-6-phenyl-1*H*-pyrazolo[3,4-*b*]pyridine (5f), 42 mg, 60%, white solid, m.p. 128–129 °C. ^{1}H NMR (400 MHz, $CDCl_3$): δ (ppm) 8.54 (s, 1H), 7.65 – 7.56 (m, 2H), 7.52 – 7.42 (m, 3H), 4.05 (s, 3H), 2.56 (s, 3H). ^{13}C NMR (125 MHz, $CDCl_3$): δ (ppm) 159.4, 150.2, 142.4, 140.2, 139.2, 129.4, 128.7, 127.9, 116.3, 83.1, 12.3. HR-MS (ESI): calcd for $[M+H]^+$ $C_{14}H_{13}IN_3$: 350.0149; found: 350.0149.

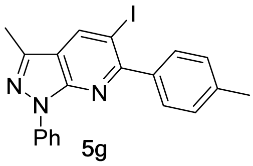

5g

5-Iodo-3-methyl-1-phenyl-6-(*p*-tolyl)-1*H*-pyrazolo[3,4-*b*]pyridine (5g), 49 mg, 58%, white solid, m.p. 139–140 °C. ^{1}H NMR (400 MHz, $CDCl_3$): δ (ppm) 8.60 (s, 1H), 8.34 – 8.22 (m, 2H), 7.65 – 7.55 (m, 2H), 7.50 – 7.42 (m, 2H), 7.30 (dd, J = 8.4, 0.6 Hz, 2H), 7.25 – 7.17 (m, 1H), 2.64 (s, 3H), 2.46 (s, 3H). ^{13}C NMR (125 MHz, $CDCl_3$): δ (ppm) 159.6, 149.9, 141.3, 140.5, 139.4, 138.7, 129.7, 128.9, 128.5, 125.3, 120.3, 118.1, 84.2, 21.4, 12.4. HR-MS (ESI): calcd for $[M+H]^+$ $C_{20}H_{17}IN_3$: 426.0462; found: 426.0464.

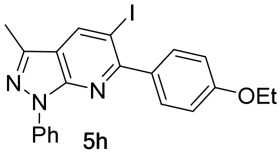

5h

6-(4-Ethoxyphenyl)-5-iodo-3-methyl-1-phenyl-1*H*-pyrazolo[3,4-*b*]pyridine (5h), 60 mg, 66%, white solid, m.p. 188–189 °C. ^{1}H NMR (400 MHz, $CDCl_3$): δ (ppm) 8.59 (s, 1H), 8.41 – 8.17 (m, 2H), 7.74 – 7.59 (m, 2H), 7.49 – 7.42 (m, 2H), 7.23 (tt, J = 7.8, 1.1 Hz, 2H), 7.08 – 6.91 (m, 2H), 4.13 (q, J = 7.0 Hz, 2H), 2.64 (s, 3H), 1.47 (t,

J = 7.0 Hz, 3H). ^{13}C NMR (100 MHz, CDCl$_3$): δ (ppm) 159.5, 159.2, 150.0, 141.4, 140.6, 139.4, 134.5, 131.3, 128.9, 125.4, 120.4, 117.9, 113.6, 84.2, 63.5, 14.8, 12.5. HR-MS (ESI): calcd for $[M+H]^+$ $C_{21}H_{19}IN_3O$: 456.0567; found: 456.0567.

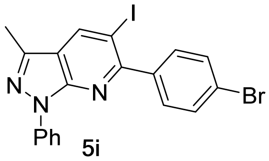

5i

6-(4-Bromophenyl)-5-iodo-3-methyl-1-phenyl-1*H*-pyrazolo[3,4-*b*]pyridine (5i), 66 mg, 68%, white solid, m.p. 167–168 °C. ^{1}H NMR (400 MHz, CDCl$_3$): δ (ppm) 8.60 (s, 1H), 8.35 – 8.09 (m, 2H), 7.65 – 7.52 (m, 4H), 7.46 (ddd, J = 10.6, 5.7, 1.9 Hz, 2H), 7.24 (dt, J = 7.8, 1.3 Hz, 1H), 2.65 (s, 3H). ^{13}C NMR (125 MHz, CDCl$_3$): δ (ppm) 158.3, 149.8, 141.4, 141.0, 140.7, 139.3, 131.5, 131.0, 129.0, 125.6, 123.3, 120.4, 118.4, 83.6, 12.5. HR-MS (ESI): calcd for $[M+H]^+$ $C_{19}H_{14}BrIN_3$: 489.9410; found: 489.9411.

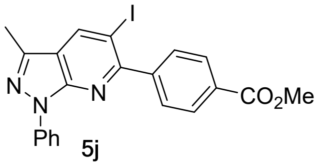

5j

Methyl 4-(5-iodo-3-methyl-1-phenyl-1*H*-pyrazolo[3,4-*b*]pyridin-6-yl)benzoate (5j), 60 mg, 64%, white solid, m.p. 182–183 °C. ^{1}H NMR (400 MHz, CDCl$_3$): δ (ppm) 8.60 (s, 1H), 8.32 – 8.22 (m, 2H), 8.22 – 8.10 (m, 2H), 7.85 – 7.62 (m, 2H), 7.55 – 7.39 (m, 2H), 7.26 – 7.21 (m, 1H), 3.97 (s, 3H), 2.65 (s, 3H). ^{13}C NMR (125 MHz, CDCl$_3$): δ (ppm) 166.7, 158.5, 149.7, 146.4, 141.5, 140.7, 139.3, 130.3, 129.9, 129.1, 129.0, 125.6, 120.4, 118.5, 83.4, 52.2, 12.5. HR-MS (ESI): calcd for $[M+H]^+$ $C_{21}H_{17}IN_3O_2$: 470.0360; found: 470.0362.

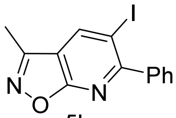

5k

5-Iodo-3-methyl-6-phenylisoxazolo[5,4-*b*]pyridine (5k), 42 mg, 63%, white solid, m.p. 119–120 °C. ^{1}H NMR (400 MHz, CDCl$_3$): δ (ppm) 8.57 (s, 1H), 7.65 – 7.63 (m, 1H), 7.62 – 7.60 (m, 1H), 7.52 – 7.45 (m, 3H), 2.60 (s, 3H). ^{13}C NMR (125 MHz, CDCl$_3$): δ (ppm) 169.1, 161.8, 154.5, 142.1, 141.0, 129.4, 129.3, 127.9, 114.7, 86.5, 10.8. HR-MS (ESI): calcd for $[M+H]^+$ $C_{13}H_9IN_2O$: 336.9832; found: 336.9833.

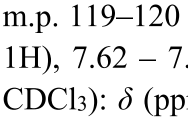

5l

5-Bromo-3-methyl-1,6-diphenyl-1*H*-pyrazolo[3,4-*b*]pyridine (5l), 44 mg, 66%, white solid, m.p. 151–152 °C. ^{1}H NMR (400 MHz, CDCl$_3$): δ (ppm) 8.34 (s, 1H), 8.32 – 8.28 (m, 2H), 7.80 – 7.75 (m, 2H), 7.53 – 7.48 (m, 3H), 7.48 – 7.43 (m, 2H), 7.24 (d, J = 7.5 Hz, 1H), 2.66 (s, 3H). ^{13}C NMR (125 MHz, CDCl$_3$): δ (ppm) 156.8,

149.3, 141.6, 139.9, 139.4, 133.9, 129.8, 129.0, 128.8, 127.8, 125.5, 120.3, 117.5, 111.8, 12.50. HR-MS (ESI): calcd for $[M+H]^+$ $C_{19}H_{15}BrN_3$: 364.0444; found: 364.0445.

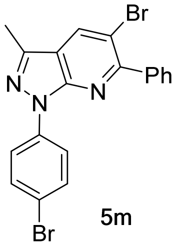

5m

5-Bromo-1-(4-bromophenyl)-3-methyl-6-phenyl-1*H*-pyrazolo[3,4-*b*]pyridine (5m), 55 mg, 63%, white solid, m.p. 178–179 °C. ^{1}H NMR (400 MHz, $CDCl_3$): δ (ppm) 8.34 (s, 1H), 8.29 – 8.21 (m, 2H), 7.79 – 7.72 (m, 2H), 7.59 – 7.54 (m, 2H), 7.50 (dd, J = 5.0, 2.1 Hz, 3H), 2.64 (s, 3H). ^{13}C NMR (125 MHz, $CDCl_3$): δ (ppm) 157.0, 149.3, 142.1, 139.8, 138.5, 134.0, 132.0, 129.8, 128.9, 127.9, 121.6, 118.5, 117.7, 112.1, 12.5. HR-MS (ESI): calcd for $[M+H]^+$ $C_{19}H_{14}Br_2N_3$: 441.9549; found: 441.9551.

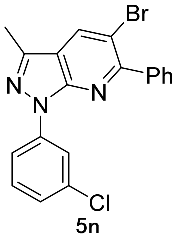

5n

5-Bromo-1-(3-chlorophenyl)-3-methyl-6-phenyl-1*H*-pyrazolo[3,4-*b*]pyridine (5n), 49 mg, 62%, white solid, m.p. 143–144 °C. ^{1}H NMR (400 MHz, $CDCl_3$): δ (ppm) 8.39 – 8.33 (m, 3H), 7.81 – 7.74 (m, 2H), 7.54 – 7.48 (m, 3H), 7.38 (td, J = 8.0, 0.5 Hz, 1H), 7.21 (ddd, J = 8.0, 2.0, 1.0 Hz, 1H), 2.65 (s, 3H). ^{13}C NMR (125 MHz, $CDCl_3$): δ (ppm) 157.0, 149.4, 142.3, 140.4, 139.7, 134.7, 134.1, 130.1, 129.8, 129.02, 127.9, 125.3, 120.1, 117.9, 117.7, 112.2, 99.9, 12.5. HR-MS (ESI): calcd for $[M+H]^+$ $C_{19}H_{14}BrClN_3$: 397.0054; found: 397.0056.

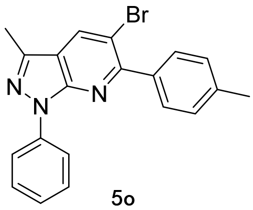

5o

5-Bromo-3-methyl-1-phenyl-6-(*p*-tolyl)-1*H*-pyrazolo[3,4-*b*]pyridine (5o), 46 mg, 61%, white solid, m.p. 124–125 °C. ^{1}H NMR (400 MHz, $CDCl_3$): δ (ppm) 8.32 (s, 1H), 8.32 – 8.29 (m, 2H), 7.72 – 7.66 (m, 2H), 7.49 – 7.43 (m, 2H), 7.35 – 7.29 (m, 2H), 7.25 – 7.19 (m, 1H), 2.65 (s, 3H), 2.45 (s, 3H). ^{13}C NMR (125 MHz, $CDCl_3$): δ (ppm) 156.8, 141.6, 139.5, 138.9, 137.1, 135.6, 129.8, 129.0, 128.6, 126.6, 125.4, 120.3, 117.4, 111.8, 21.4, 12.5. HR-MS (ESI): calcd for $[M+H]^+$ $C_{20}H_{17}BrN_3$: 378.0600; found: 378.0601.

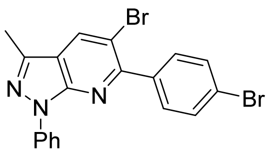

5p

5-Bromo-6-(4-bromophenyl)-3-methyl-1-phenyl-1*H*-pyrazolo[3,4-*b*]pyridine (5p), 53 mg, 60%, white solid, m.p. 181–182 °C. ^{1}H NMR (400 MHz, $CDCl_3$): δ (ppm) 8.34 (s, 1H), 8.30 – 8.21 (m, 2H), 7.70 – 7.60 (m, 4H), 7.51 – 7.41 (m, 2H), 7.26 – 7.23 (m, 1H), 2.66 (s, 3H). ^{13}C NMR (125 MHz, $CDCl_3$): δ (ppm) 155.6, 149.2, 141.7, 139.3, 138.7, 134.1, 131.5, 131.1, 129.0, 125.6, 123.4, 120.4, 117.6, 111.4, 12.5. HR-MS (ESI): calcd for $[M+H]^+$ $C_{19}H_{14}Br_2N_3$: 441.9549; found: 441.9549.

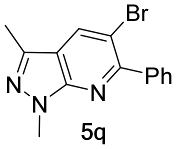

5q

5-Bromo-1,3-dimethyl-6-phenyl-1*H*-pyrazolo[3,4-*b*]pyridine (5q), 39 mg, 64%, white solid, m.p. 118–119 °C. ^{1}H NMR (400 MHz, $CDCl_3$): δ (ppm) 8.27 (s, 1H), 7.70 (dd, J = 2.4, 1.6 Hz, 1H), 7.69 (dd, J = 2.7, 1.2 Hz, 1H), 7.51 – 7.48 (m, 1H), 7.48 – 7.43 (m, 2H), 4.07 (s, 3H), 2.57 (s, 3H). ^{13}C NMR (125 MHz, $CDCl_3$): δ (ppm) 156.6, 149.7, 140.0, 139.5, 133.6, 129.5, 128.8, 127.9, 115.4, 110.8, 33.7, 12.4. HR-MS (ESI): calcd for $[M+H]^+$ $C_{14}H_{13}BrN_3$: 302.0287; found: 302.0288.

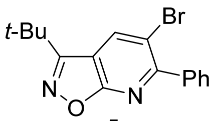

5r

5-Bromo-3-(*tert*-butyl)-6-phenylisoxazolo[5,4-*b*]pyridine (5r), 35 mg, 53%, white solid, m.p. 126–127 °C. ^{1}H NMR (400 MHz, $CDCl_3$): δ (ppm) 8.44 (s, 1H), 7.77 – 7.69 (m, 2H), 7.52 – 7.47 (m, 3H), 1.56 (s, 9H). ^{13}C NMR (125 MHz, $CDCl_3$): δ (ppm) 168.7, 165.3, 158.3, 138.6, 137.0, 129.6, 129.5, 128.0, 113.8, 112.3, 34.2, 28.9. HR-MS (ESI): calcd for $[M+H]^+$ $C_{16}H_{16}BrN_2O$: 331.0441; found: 331.0442.

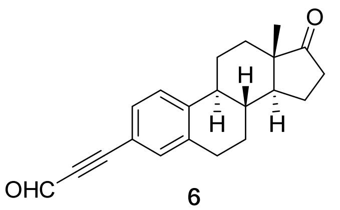

6

3-((8R,9S,13S,14S)-13-Methyl-17-oxo-7,8,9,11,12,13,14,15,16,17-decahydro-6*H*-cyclopenta[*a*]phenanthren-3-yl)propiolaldehyde (6), 826 mg, 54%, white solid, m.p.190-192 °C. ^{1}H NMR (400 MHz, $CDCl_3$): δ (ppm) 9.40 (s, 1H), 7.39 (dd, J = 8.1, 1.4 Hz, 1H), 7.36 – 7.30 (m, 2H), 2.91 (dd, J = 9.7, 5.1 Hz, 2H), 2.51 (dd, J = 18.7, 8.7 Hz, 1H), 2.45 – 2.38 (m, 1H), 2.32 (td, J = 11.7, 10.5, 4.3 Hz, 1H), 2.21 – 2.12 (m, 1H), 2.11 – 1.95 (m, 3H), 1.63 (m, 3H), 1.56 – 1.47 (m, 3H), 0.91 (s, 3H). ^{13}C NMR (100 MHz, $CDCl_3$): δ (ppm) 220.4, 176.7, 143.9, 137.2, 133.8, 130.6, 125.8, 116.6, 95.8, 88.3, 50.4, 47.8, 44.6, 37.7, 35.7, 31.4, 28.9, 26.1, 25.4, 21.5, 13.7. HR-MS

(ESI): calcd for $[M+H]^+$ $C_{21}H_{23}O_2$: 307.1693; found: 307.1694. The experimental data of **6** match with those reported in the literature [50].

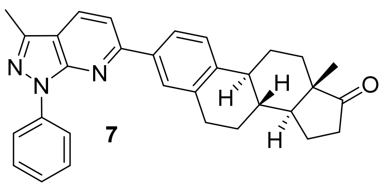

7

(8R,9S,13S,14S)-13-Methyl-3-(3-methyl-1-phenyl-1*H*-pyrazolo[3,4-*b*]pyridin-6-yl)-6,7,8,9,11,12,13,14,15,16-decahydro-17*H*-cyclopenta[*a*]phenanthren-17-one (7), 48 mg, 52%, white solid, m.p. 199-201 °C. ^{1}H NMR (400 MHz, $CDCl_3$): δ (ppm) 8.43 (d, J = 7.6 Hz, 2H), 8.05 (d, J = 8.3 Hz, 1H), 7.94 (dd, J = 8.2, 1.9 Hz, 1H), 7.87 (d, J = 1.7 Hz, 1H), 7.63 (d, J = 8.4 Hz, 1H), 7.57 – 7.50 (m, 2H), 7.44 (d, J = 8.1 Hz, 1H), 7.31 – 7.26 (m, 1H), 3.04 (dd, J = 10.6, 4.6 Hz, 2H), 2.65 (s, 3H), 2.59 – 2.50 (m, 1H), 2.48 (d, J = 9.3 Hz, 1H), 2.42 – 2.33 (m, 1H), 2.22 – 2.12 (m, 1H), 2.08 (m, 2H), 2.04 – 1.98 (m, 1H), 1.69 – 1.50 (m, 6H), 0.94 (s, 3H). ^{13}C NMR (100 MHz, $CDCl_3$): δ (ppm) 220.7, 156.7, 151.0, 142.5, 141.3, 139.8, 136.8, 136.8, 129.7, 128.9, 128.0, 125.8, 125.0, 124.9, 120.4, 115.6, 114.2, 50.5, 47.9, 44.4, 38.0, 35.8, 31.5, 29.5, 26.4, 25.6, 21.5, 13.8, 12.5. HR-MS (ESI): calcd for $[M+H]^+$ $C_{31}H_{32}N_3O$: 462.2540; found: 462.2542.

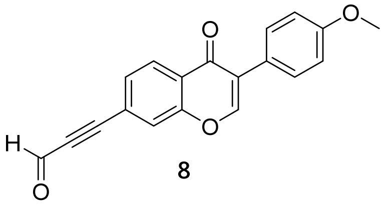

8

3-(3-(4-Methoxyphenyl)-4-oxo-4*H*-chromen-7-yl)propiolaldehyde (8), 852 mg, 56%, yellow solid, m.p. 164-166 °C. ^{1}H NMR (400 MHz, $CDCl_3$): δ (ppm) 9.47 (s, 1H), 8.33 (dd, J = 8.2, 0.5 Hz, 1H), 8.02 (s, 1H), 7.73 (dd, J = 1.4, 0.5 Hz, 1H), 7.58 (dd, J = 8.2, 1.5 Hz, 1H), 7.51 – 7.48 (m, 2H), 7.00 – 6.97 (m, 2H), 3.84 (s, 3H). ^{13}C NMR (100 MHz, CDCl3): δ (ppm) 176.2, 175.5, 159.8, 155.4, 152.7, 130.0, 128.7, 127.0, 125.8, 125.6, 124.3, 123.3, 122.9, 114.0, 91.6, 89.7, 55.3. HR-MS (ESI): calcd for $[M+H]^+$ $C_{19}H_{13}O_4$: 305.0808; found: 305.0810.

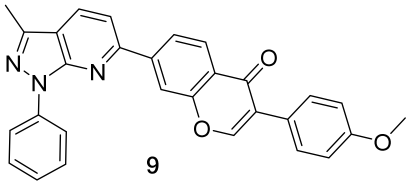

9

3-(4-Methoxyphenyl)-7-(3-methyl-1-phenyl-1*H*-pyrazolo[3,4-*b*]pyridin-6-yl)-4*H*-chromen-4-one (9), 66 mg, 72%, yellow solid, m.p. 213-215 °C. ^{1}H NMR (400 MHz, $CDCl_3$): δ (ppm) 8.49 – 8.35 (m, 3H), 8.28 (d, J = 1.6 Hz, 1H), 8.19 (dd, J = 8.4, 1.6 Hz, 1H), 8.15 (d, J = 8.3 Hz, 1H), 8.06 (s, 1H), 7.76 (d, J = 8.3 Hz, 1H), 7.59 – 7.52 (m, 4H), 7.35 – 7.28 (m, 1H), 7.04 – 6.95 (m, 2H), 3.86 (s, 3H), 2.69 (s, 3H). ^{13}C NMR (100 MHz, $CDCl_3$): δ (ppm) 176.2, 159.6, 156.4, 154.2, 152.8, 150.8, 144.3, 142.6, 139.5, 130.3, 130.0, 129.0, 126.8, 125.5, 125.2, 124.6, 120.6, 116.9, 116.5,

114.5, 114.0, 55.3, 12.5. HR-MS (ESI): calcd for $[M+H]^+$ $C_{29}H_{22}N_3O_3$: 460.1656; found: 460.1657.

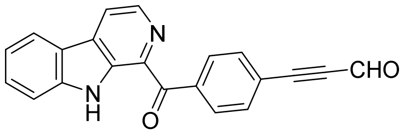

10

3-(4-(9*H*-Pyrido[3,4-*b*]indole-1-carbonyl)phenyl)propiolaldehyde (10), 892 mg, 55%, yellow solid, m.p. 185-187 °C. ^{1}H NMR (400 MHz, $CDCl_3$): δ (ppm) 10.45 (s, 1H), 9.48 (s, 1H), 8.60 (d, J = 4.9 Hz, 1H), 8.37 (d, J = 8.4 Hz, 2H), 8.22 – 8.16 (m, 2H), 7.76 (d, J = 8.4 Hz, 2H), 7.64 – 7.61 (m, 2H), 7.37 (m, 1H). ^{13}C NMR (100 MHz, $CDCl_3$): δ (ppm) 194.2, 176.6, 141.0, 139.5, 138.1, 137.3, 135.6, 132.6, 131.9, 131.3, 129.4, 122.9, 121.9, 121.0, 120.7, 118.9, 112.0, 93.8, 89.6. HR-MS (ESI): calcd for $[M+H]^+$ $C_{21}H_{13}N_2O_2$: 325.0972; found: 325.0974.

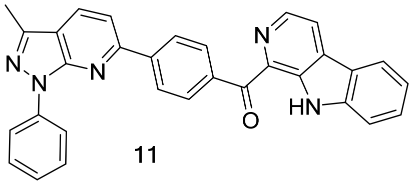

11

(4-(3-Methyl-1-phenyl-1*H*-pyrazolo[3,4-*b*]pyridin-6-yl)phenyl)(9H-pyrido[3,4-*b*]indol-1-yl)methanone (11), 67 mg, 70%, yellow solid, m.p. 239-241 °C. ^{1}H NMR (400 MHz, DMSO-d_6): δ (ppm) 11.99 (s, 1H), 8.43 (d, J = 4.9 Hz, 1H), 8.35 – 8.29 (m, 2H), 8.29 – 8.25 (m, 5H), 8.24 – 8.17 (m, 2H), 7.89 (d, J = 8.4 Hz, 1H), 7.74 – 7.68 (m, 1H), 7.53 – 7.43 (m, 3H), 7.20 (m, 2H), 3.21 (s, 3H). ^{13}C NMR (100 MHz, DMSO-d_6): δ (ppm) 193.7, 155.4, 150.8, 143.4, 142.2, 142.0, 139.7, 138.6, 137.7, 136.7, 136.3, 132.1, 131.9, 131.6, 129.7, 129.5, 127.3, 125.7, 122.3, 120.7, 120.5, 120.3, 119.5, 115.5, 113.5, 79.6, 12.7. HR-MS (ESI): calcd for $[M+H]^+$ $C_{31}H_{22}N_5O$: 480.1819; found: 480.1820.

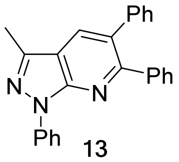

13

3-Methyl-1,5,6-triphenyl-1*H*-pyrazolo[3,4-*b*]pyridine (13), 63 mg, 88%, yellow solid, m.p.185-186 °C. ^{1}H NMR (400 MHz, $CDCl_3$): δ (ppm) 8.48 – 8.40 (m, 2H), 8.05 (s, 1H), 7.56 – 7.45 (m, 4H), 7.34 – 7.18 (m, 10H), 2.69 (s, 3H). ^{13}C NMR (125 MHz, $CDCl_3$): δ (ppm) 157.2, 150.1, 142.6, 140.6, 140.3, 139.8, 131.5, 130.5, 130.4, 130.4, 129.9, 128.9, 128.3, 128.0, 127.7, 126.9, 125.1, 120.3, 115.9, 12.6. HR-MS (ESI): calcd for $[M+H]^+$ $C_{25}H_{20}N_3$: 362.1652; found: 362.1653.

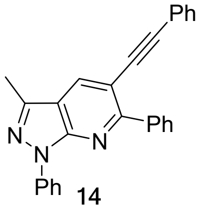

14

3-Methyl-1,6-diphenyl-5-(phenylethynyl)-1*H*-pyrazolo[3,4-*b*]pyridine (14), 65 mg, 85%, yellow solid, m.p.147-148 °C. ^{1}H NMR (400 MHz, $CDCl_3$): δ (ppm) 8.42 – 8.35 (m, 2H), 8.32 (s, 1H), 8.19 – 8.11 (m, 2H), 7.56 – 7.51 (m, 3H), 7.51 – 7.48 (m, 2H), 7.44 – 7.39 (m, 2H), 7.36 – 7.31 (m, 3H), 7.29 – 7.25 (m, 1H), 2.68 (s, 3H). ^{13}C NMR (125 MHz, $CDCl_3$): δ (ppm) 159.2, 149.4, 142.6, 139.5, 134.3, 131.1, 129.9, 129.1, 128.9, 128.4, 128.3, 127.8, 125.4, 123.2, 120.4, 115.4, 111.6, 92.9, 88.3, 12.5. HR-MS (ESI): calcd for $[M+H]^+$ $C_{27}H_{20}N_3$: 386.1652; found: 386.1655.

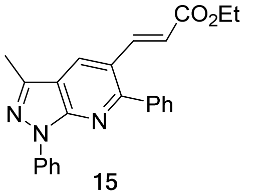

15

Ethyl (*E*)-3-(3-methyl-1,6-diphenyl-1*H*-pyrazolo[3,4-*b*]pyridin-5-yl)acrylate (15), 57 mg, 75%, yellow solid, m.p.165-166 °C. ^{1}H NMR (400 MHz, $CDCl_3$): δ (ppm) 8.39 – 8.34 (m, 2H), 8.32 (s, 1H), 7.90 (dd, J = 15.9, 0.5 Hz, 1H), 7.69 – 7.65 (m, 2H), 7.56 – 7.45 (m, 5H), 7.28 – 7.23 (m, 1H), 6.48 (d, J = 15.8 Hz, 1H), 4.26 (q, J = 7.1 Hz, 2H), 2.69 (s, 3H), 1.32 (t, J = 7.1 Hz, 3H). ^{13}C NMR (125 MHz, $CDCl_3$): δ (ppm) 166.5, 158.9, 150.5, 143.2, 143.2, 139.5, 139.2, 130.3, 129.1, 129.0, 128.6, 128.3, 125.5, 122.9, 120.4, 118.7, 116.2, 60.5, 14.3, 12.5. HR-MS (ESI): calcd for $[M+H]^+$ $C_{24}H_{22}N_3O_2$: 384.1707; found: 384.1708.

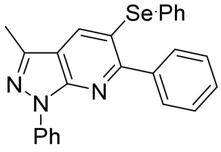

16

3-Methyl-1,6-diphenyl-5-(phenylselanyl)-1*H*-pyrazolo[3,4-*b*]pyridine (16), 68 mg, 78%, yellow solid, m.p.124-125 °C. ^{1}H NMR (400 MHz, $CDCl_3$): δ (ppm) 8.36 – 8.29 (m, 2H), 8.02 (s, 1H), 7.73 – 7.66 (m, 2H), 7.46 (tdd, J = 7.2, 3.8, 2.0 Hz, 5H), 7.43 – 7.38 (m, 2H), 7.32 – 7.26 (m, 3H), 7.25 – 7.20 (m, 1H), 2.55 (s, 3H). ^{13}C NMR (125 MHz, $CDCl_3$): δ (ppm) 159.5, 149.8, 141.9, 140.8, 139.6, 134.6, 133.6, 131.1, 129.56, 129.34, 128.9, 128.8, 127.9, 127.9, 125.3, 117.2, 12.4. HR-MS (ESI): calcd for $[M+H]^+$ $C_{25}H_{20}N_3Se$: 442.0817; found: 442.0820.

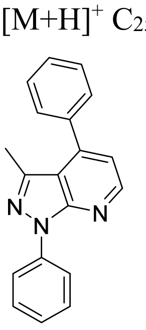

17

3-Methyl-1,4-diphenyl-1*H*-pyrazolo[3,4-*b*]pyridine (17), 26 mg, 45%, white solid, m.p. 126-128 °C. ^{1}H NMR (400 MHz, $CDCl_3$): δ (ppm) 8.59 (d, J = 4.7 Hz, 1H), 8.26 (dd, J = 8.7, 1.1 Hz, 2H), 7.54 (dd, J = 7.0, 1.5 Hz, 2H), 7.50 (m, 5H), 7.33 – 7.27 (m, 1H), 7.07 (d, J = 4.7 Hz, 1H), 2.31 (s, 3H). ^{13}C NMR (100 MHz, CDCl3): δ (ppm)

151.2, 148.6, 146.1, 142.4, 139.4, 137.4, 128.9, 128.6, 128.2, 125.6, 121.3, 117.7, 114.7, 15.4. HR-MS (ESI): calcd for $[M+H]^+$ $C_{19}H_{16}N_3$: 286.1339; found: 286.1340. The experimental data of **17** match with those reported in the literature [56].

5. Copy of ^1H and ^{13}C NMR Spectra of Products

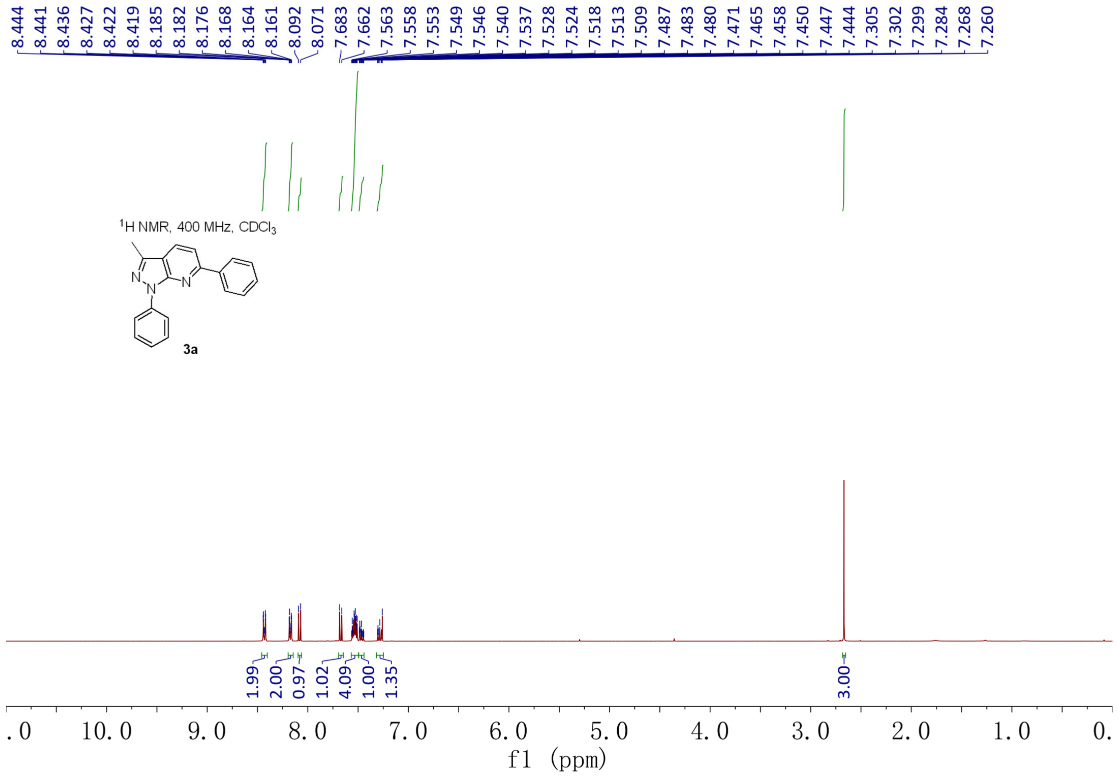

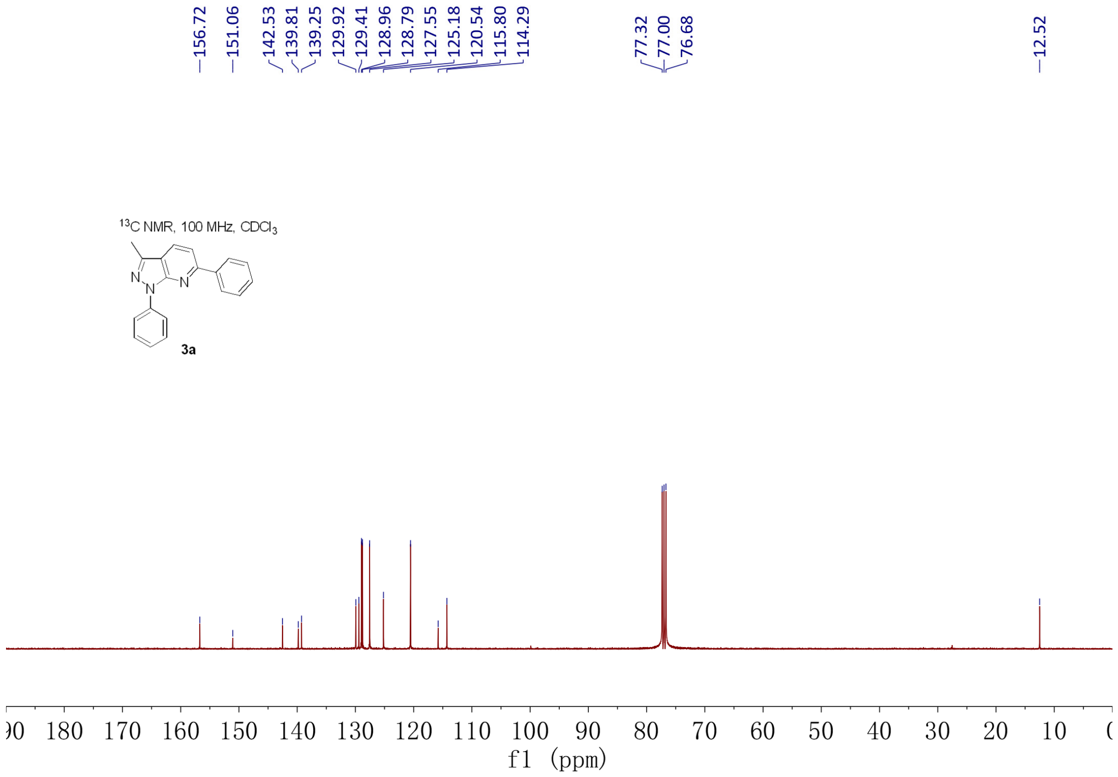

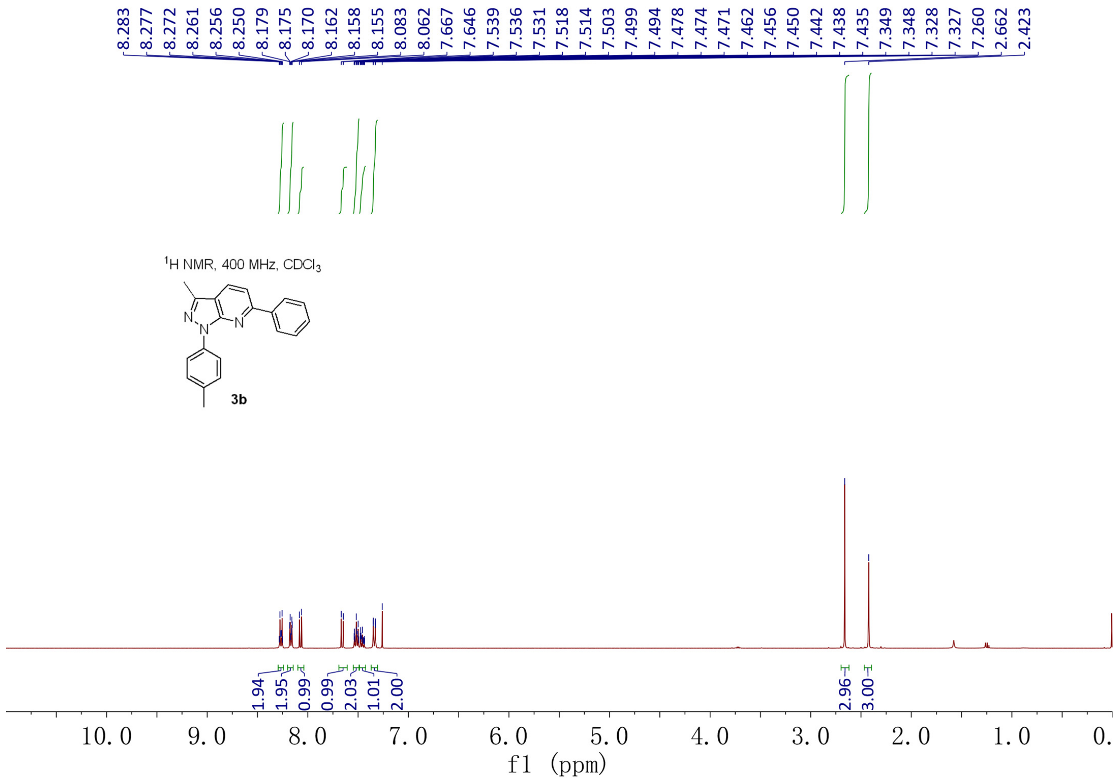
8.283
8.277
8.272
8.261
8.256
8.250
8.179
8.175
8.170
8.162
8.158
8.155
8.083
8.062
7.667
7.646
7.539
7.536
7.531
7.518
7.514
7.503
7.499
7.494
7.478
7.474
7.471
7.462
7.456
7.450
7.442
7.438
7.435
7.349
7.348
7.328
7.327
7.260
2.662
2.423
¹H NMR, 400 MHz, CDCl₃
N
N
N
3b
1.94
1.95
0.99
0.99
2.03
1.01
2.00
2.96
3.00
10.0
9.0
8.0
7.0
6.0
5.0
4.0
3.0
2.0
1.0
0.
f1 (ppm)

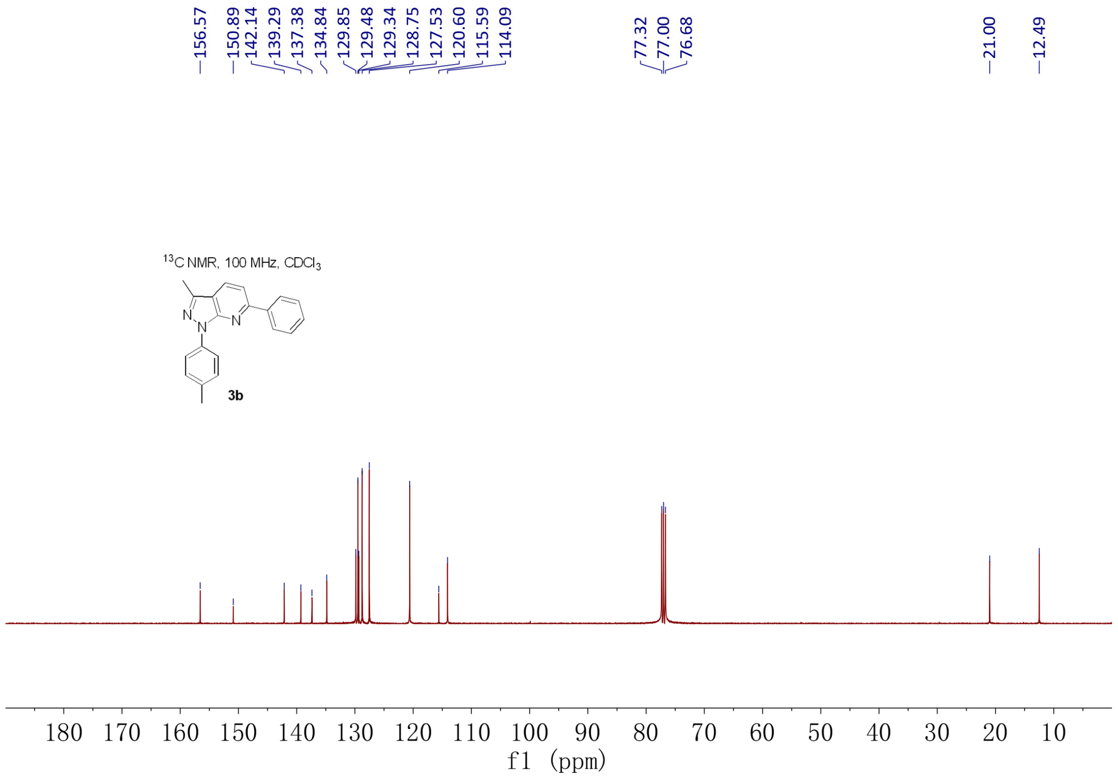
156.57
150.89
142.14
139.29
137.38
134.84
129.85
129.48
129.34
128.75
127.53
120.60
115.59
114.09
77.32
77.00
76.68
21.00
12.49
¹³C NMR, 100 MHz, CDCl₃
N
N
N
3b
180
170
160
150
140
130
120
110
100
90
80
70
60
50
40
30
20
10
f1 (ppm)

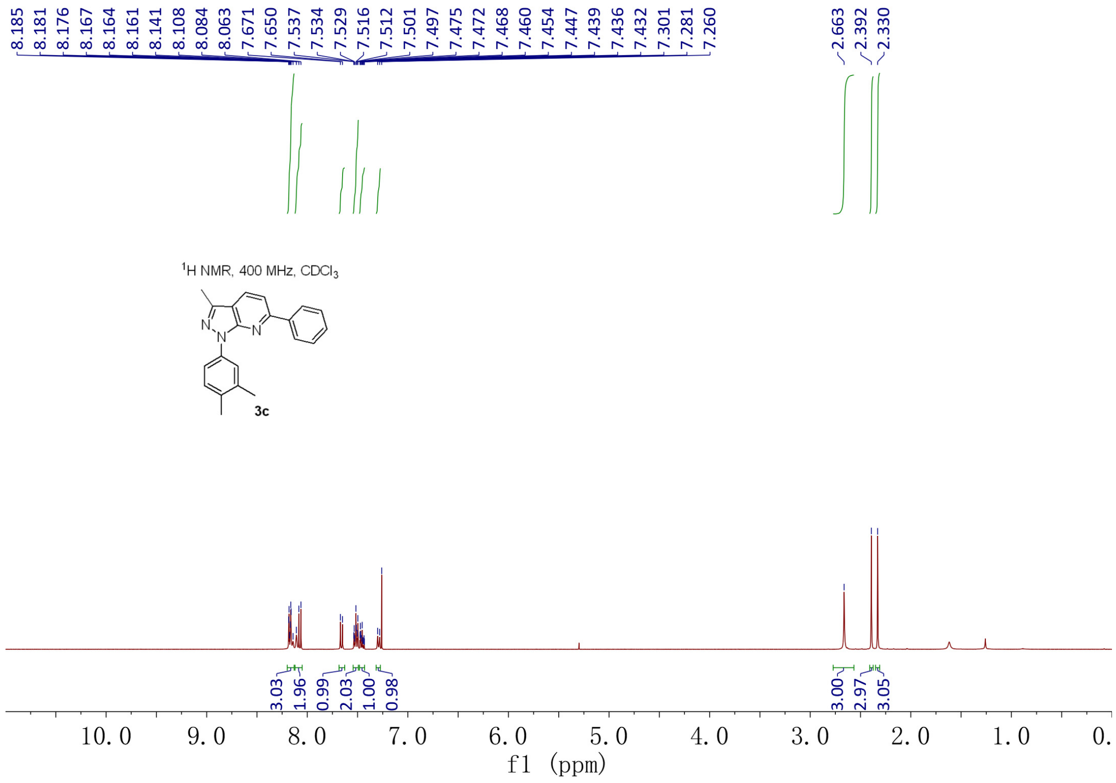
8.185
8.181
8.176
8.167
8.164
8.161
8.141
8.108
8.084
8.063
7.671
7.650
7.537
7.534
7.529
7.516
7.512
7.501
7.497
7.475
7.472
7.468
7.460
7.454
7.447
7.439
7.436
7.432
7.301
7.281
7.260
2.663
2.392
2.330
^{1}H NMR, 400 MHz, CDCl$_3$
N
N
N
3c
3.03
1.96
0.99
2.03
1.00
0.98
3.00
2.97
3.05
10.0
9.0
8.0
7.0
6.0
5.0
4.0
3.0
2.0
1.0
0.
f1 (ppm)

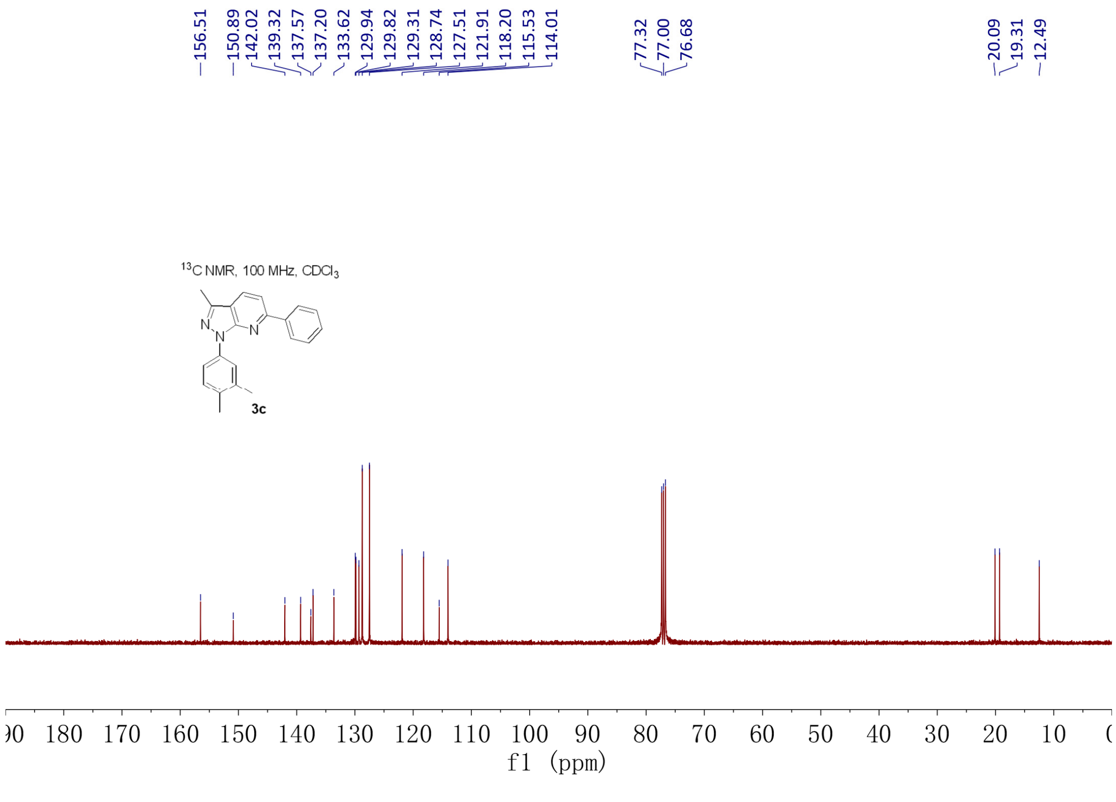
156.51
150.89
142.02
139.32
137.57
137.20
133.62
129.94
129.82
129.31
128.74
127.51
121.91
118.20
115.53
114.01
77.32
77.00
76.68
20.09
19.31
12.49
^{13}C NMR, 100 MHz, CDCl$_3$
N
N
N
3c
180
170
160
150
140
130
120
110
100
90
80
70
60
50
40
30
20
10
f1 (ppm)

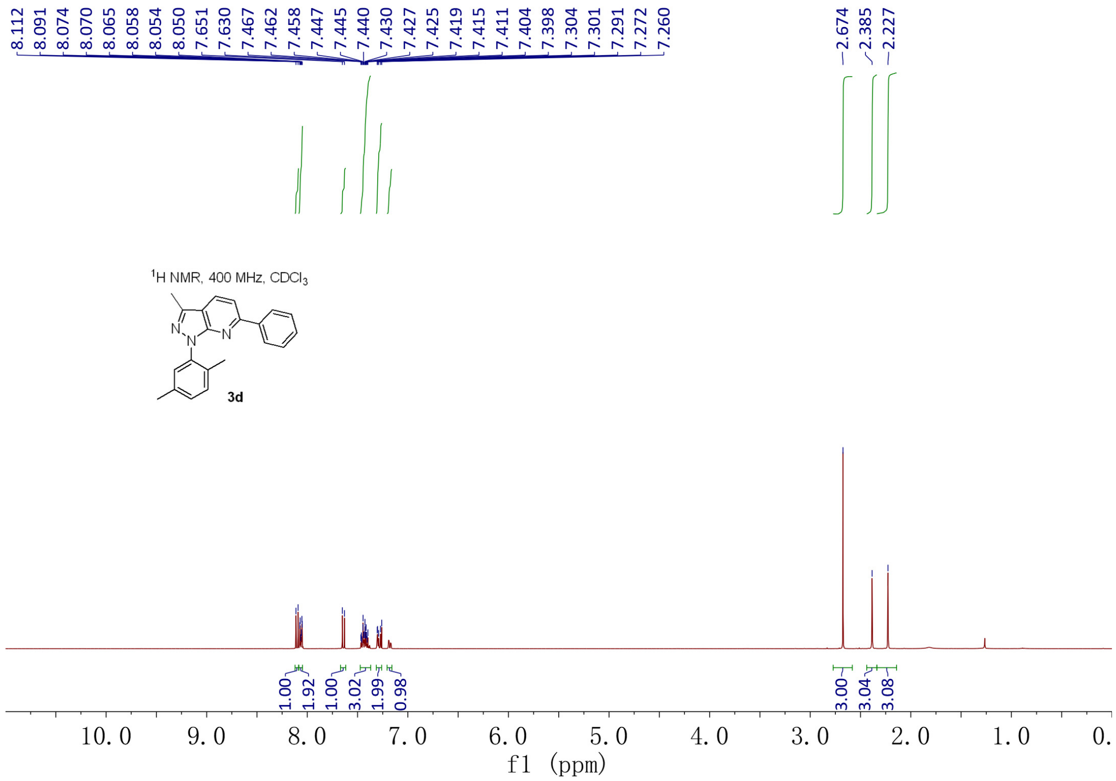
8.112
8.091
8.074
8.070
8.065
8.058
8.054
8.050
7.651
7.630
7.467
7.462
7.458
7.447
7.445
7.440
7.430
7.427
7.425
7.419
7.415
7.411
7.404
7.398
7.304
7.301
7.291
7.272
7.260
2.674
2.385
2.227
1H NMR, 400 MHz, CDCl3
N
N
N
3d
1.00
1.92
1.00
3.02
1.99
0.98
3.00
3.04
3.08
10.0
9.0
8.0
7.0
6.0
5.0
4.0
3.0
2.0
1.0
0.
f1 (ppm)

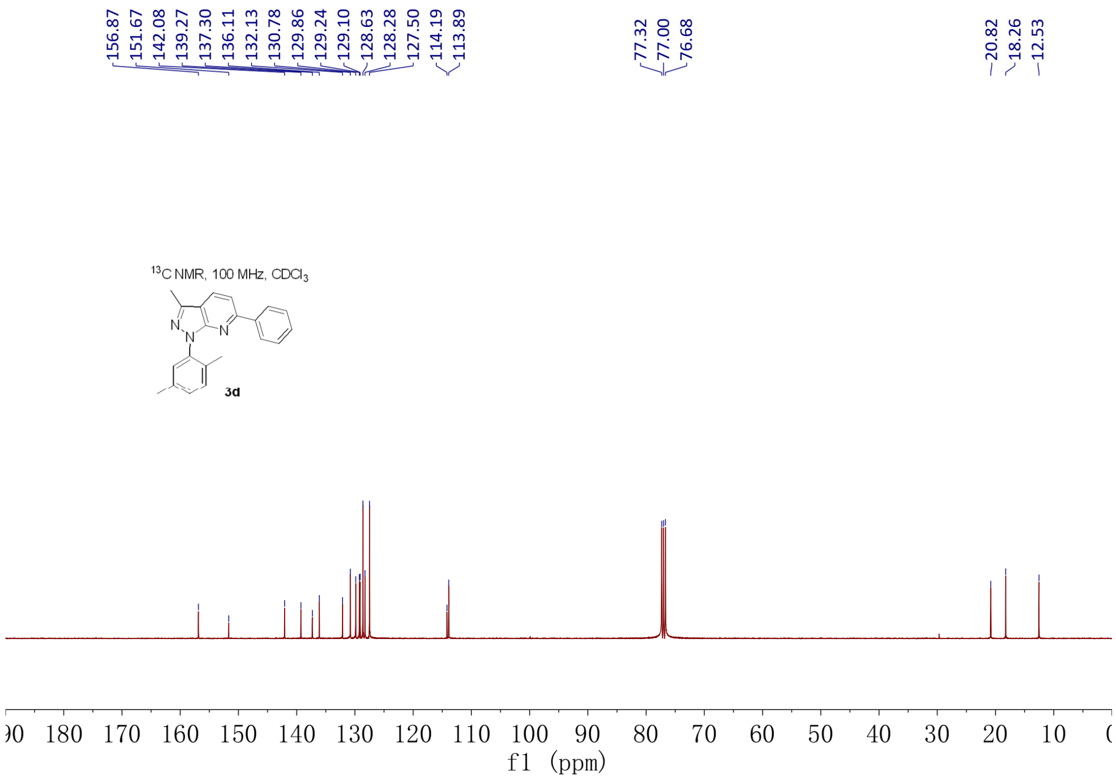
156.87
151.67
142.08
139.27
137.30
136.11
132.13
130.78
129.86
129.24
129.10
128.63
128.28
127.50
114.19
113.89
77.32
77.00
76.68
20.82
18.26
12.53
13C NMR, 100 MHz, CDCl3
N
N
N
3d
180
170
160
150
140
130
120
110
100
90
80
70
60
50
40
30
20
10
f1 (ppm)

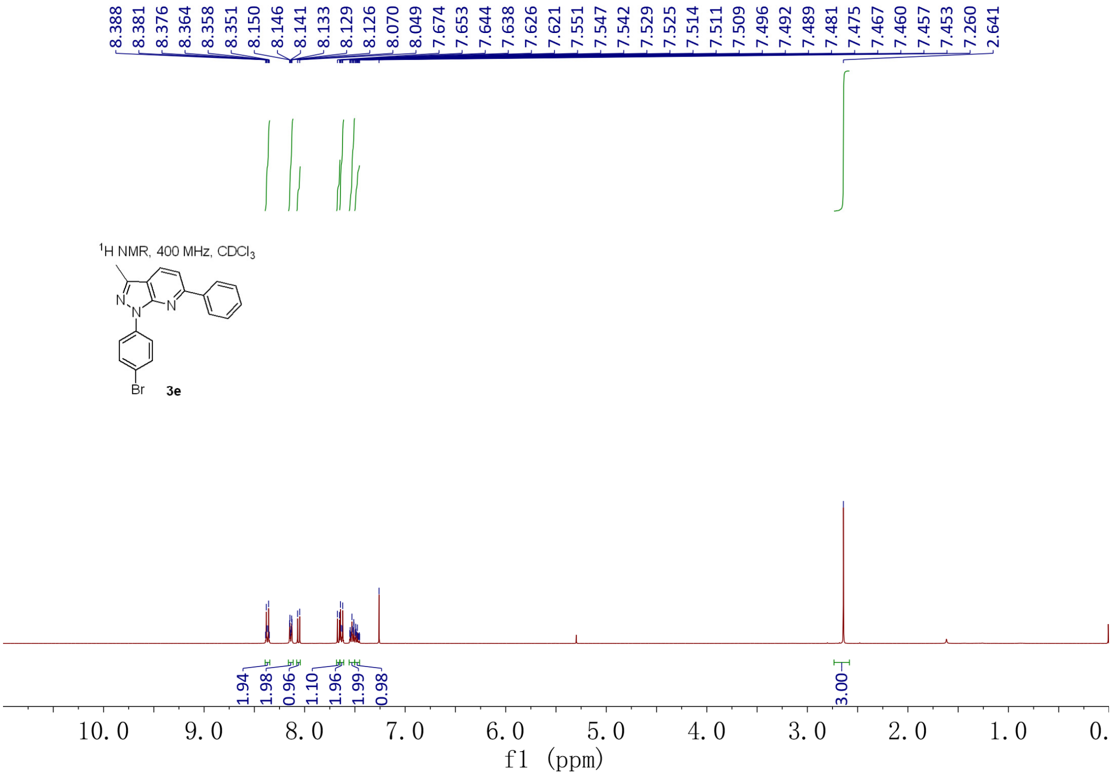
8.388
8.381
8.376
8.364
8.358
8.351
8.150
8.146
8.141
8.133
8.129
8.126
8.070
8.049
7.674
7.653
7.644
7.638
7.626
7.621
7.551
7.547
7.542
7.529
7.525
7.514
7.511
7.509
7.496
7.492
7.489
7.481
7.475
7.467
7.460
7.457
7.453
7.260
2.641
^{1}H NMR, 400 MHz, CDCl$_3$
N
N
N
Br
3e
1.94
1.98
0.96
1.10
1.96
1.99
0.98
3.00
10.0
9.0
8.0
7.0
6.0
5.0
4.0
3.0
2.0
1.0
f1 (ppm)

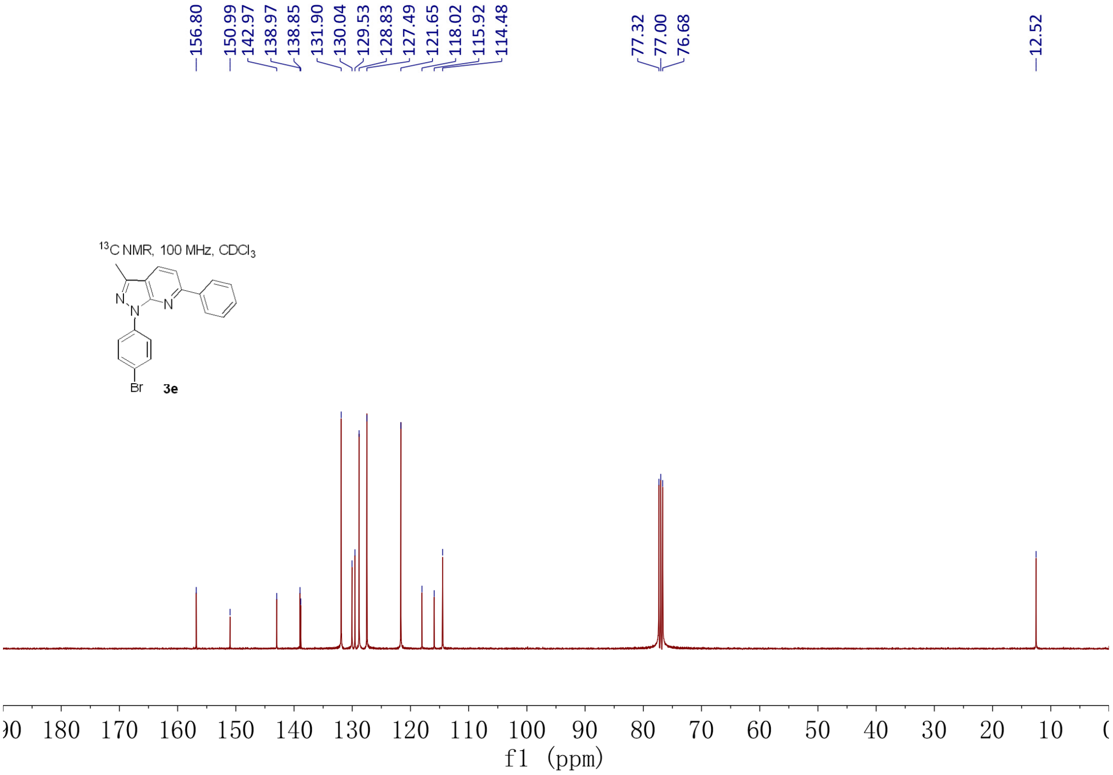
156.80
150.99
142.97
138.97
138.85
131.90
130.04
129.53
128.83
127.49
121.65
118.02
115.92
114.48
77.32
77.00
76.68
12.52
^{13}C NMR, 100 MHz, CDCl$_3$
N
N
N
Br
3e
180
170
160
150
140
130
120
110
100
90
80
70
60
50
40
30
20
10
f1 (ppm)

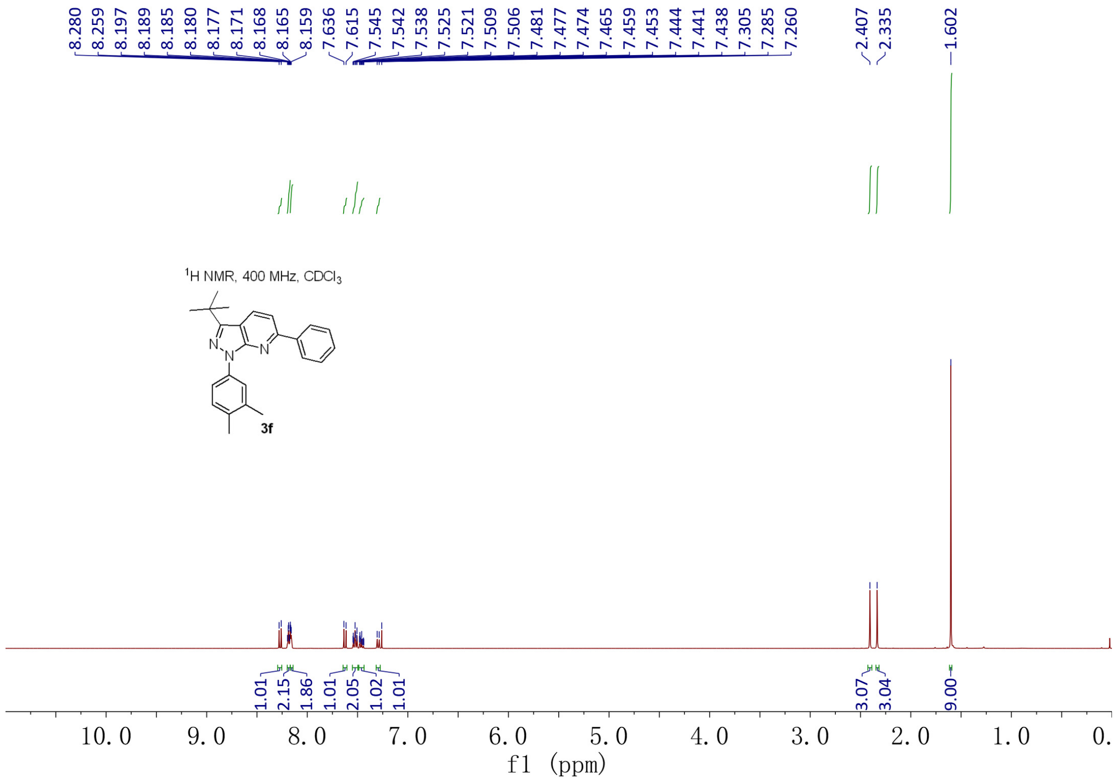

8.280
8.259
8.197
8.189
8.185
8.180
8.177
8.171
8.168
8.165
8.159
7.636
7.615
7.545
7.542
7.538
7.525
7.521
7.509
7.506
7.481
7.477
7.474
7.465
7.459
7.453
7.444
7.441
7.438
7.305
7.285
7.260
2.407
2.335
1.602
1H NMR, 400 MHz, CDCl3
N
N
N
3f
1.01
2.15
1.86
1.01
2.05
1.02
1.01
3.07
3.04
9.00
10.0
9.0
8.0
7.0
6.0
5.0
4.0
3.0
2.0
1.0
f1 (ppm)

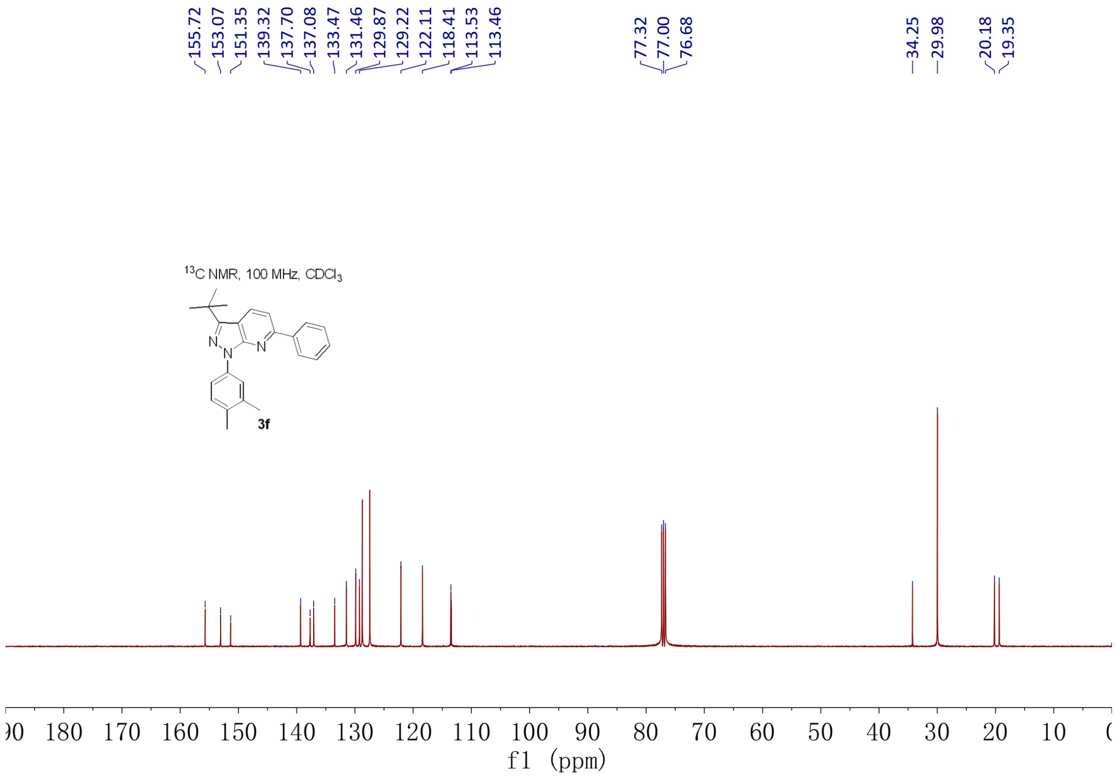

155.72
153.07
151.35
139.32
137.70
137.08
133.47
131.46
129.87
129.22
122.11
118.41
113.53
113.46
77.32
77.00
76.68
34.25
29.98
20.18
19.35
13C NMR, 100 MHz, CDCl3
N
N
N
3f
180
170
160
150
140
130
120
110
100
90
80
70
60
50
40
30
20
10
f1 (ppm)

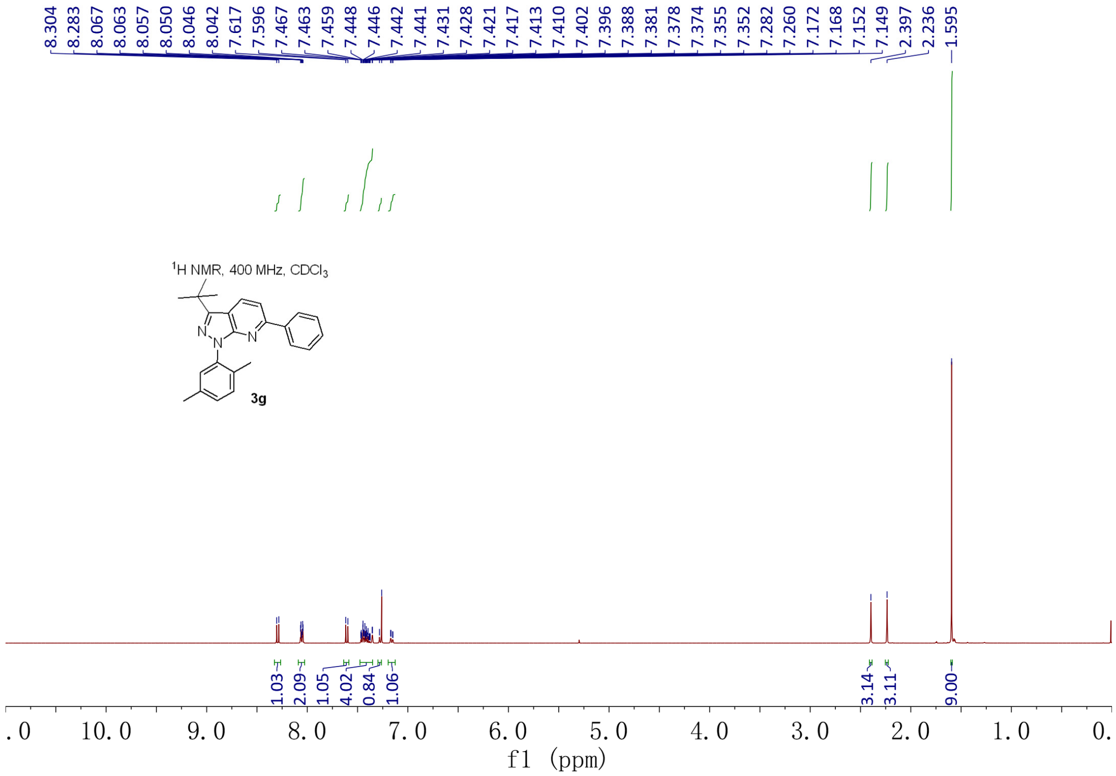
8.304 8.283 8.067 8.063 8.057 8.050 8.046 8.042 7.617 7.596 7.467 7.463 7.459 7.448 7.446 7.442 7.441 7.431 7.428 7.421 7.417 7.413 7.410 7.402 7.396 7.388 7.381 7.378 7.374 7.355 7.352 7.282 7.260 7.172 7.168 7.152 7.149 2.397 2.236 1.595
^{1}H NMR, 400 MHz, CDCl$_3$
N
N
N
3g
1.03 2.09 1.05 4.02 0.84 1.06 3.14 3.11 9.00
11.0 10.0 9.0 8.0 7.0 6.0 5.0 4.0 3.0 2.0 1.0 0.
f1 (ppm)

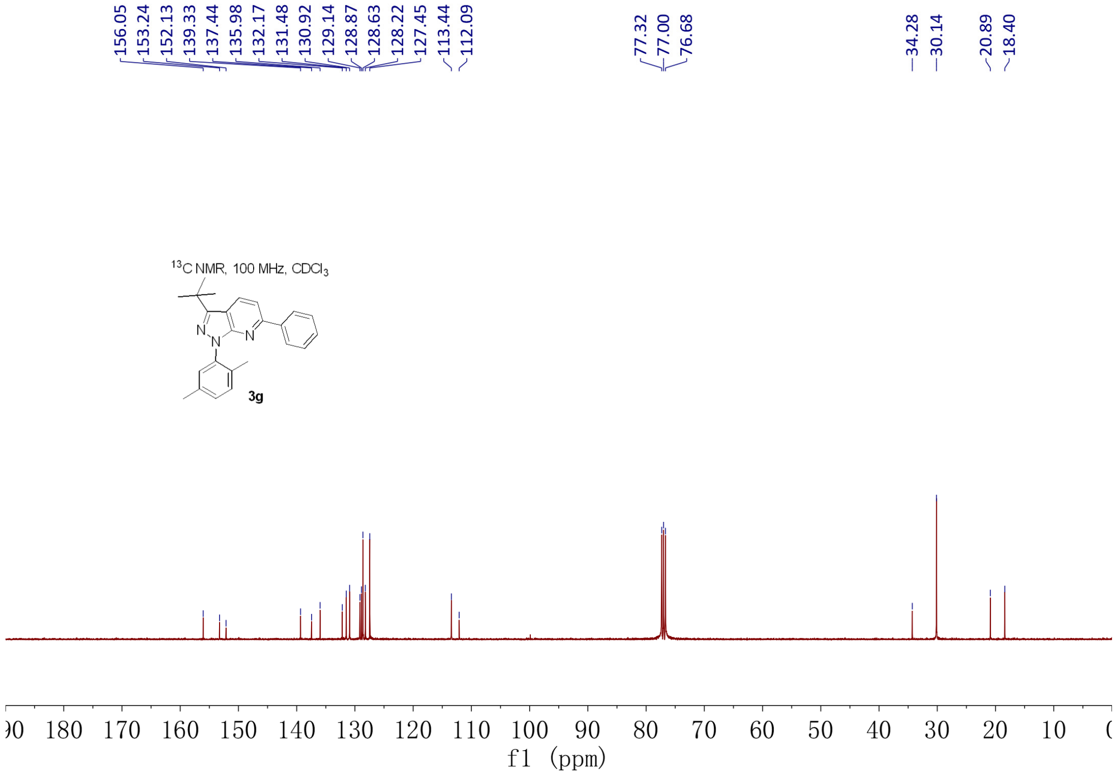
156.05 153.24 152.13 139.33 137.44 135.98 132.17 131.48 130.92 129.14 128.87 128.63 128.22 127.45 113.44 112.09
77.32 77.00 76.68
34.28 30.14 20.89 18.40
^{13}C NMR, 100 MHz, CDCl$_3$
N
N
N
3g
190 180 170 160 150 140 130 120 110 100 90 80 70 60 50 40 30 20 10 0
f1 (ppm)

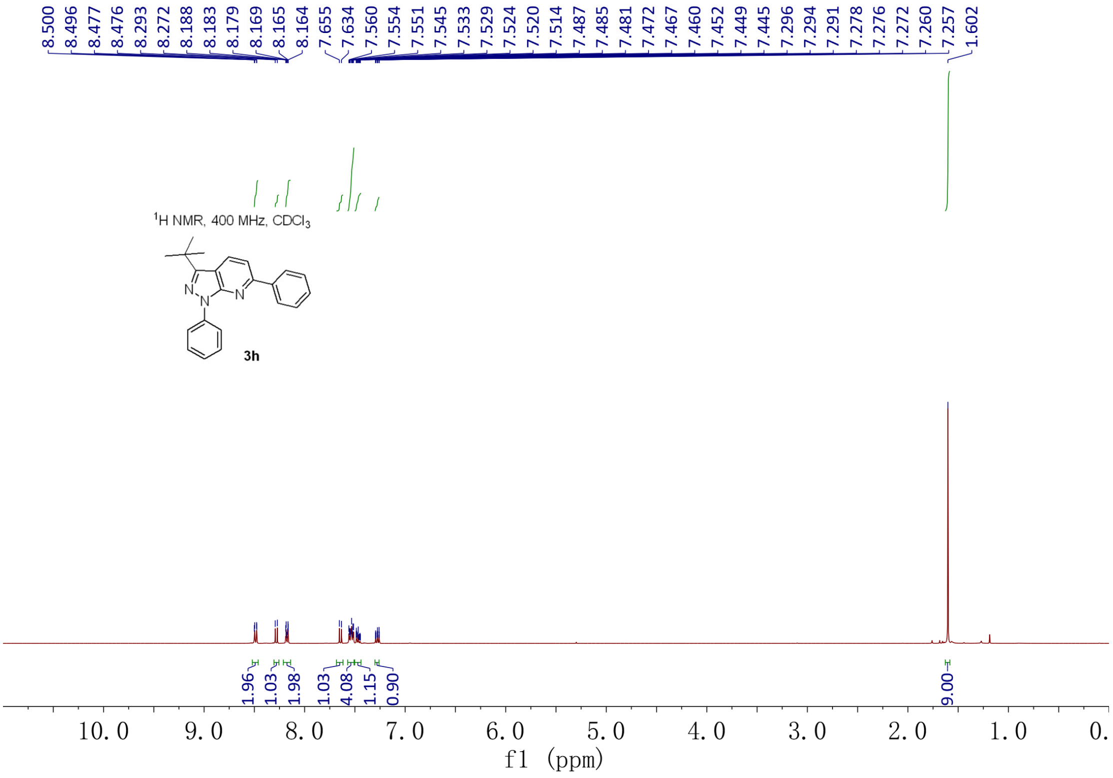
8.500 8.496 8.477 8.476 8.293 8.272 8.188 8.183 8.179 8.169 8.165 8.164 7.655 7.634 7.560 7.554 7.551 7.545 7.533 7.529 7.524 7.520 7.514 7.487 7.485 7.481 7.472 7.467 7.460 7.452 7.449 7.445 7.296 7.294 7.291 7.278 7.276 7.272 7.260 7.257 1.602
1H NMR, 400 MHz, CDCl3
3h
1.96 1.03 1.98 1.03 4.08 1.15 0.90 9.00
10.0 9.0 8.0 7.0 6.0 5.0 4.0 3.0 2.0 1.0 0.
f1 (ppm)

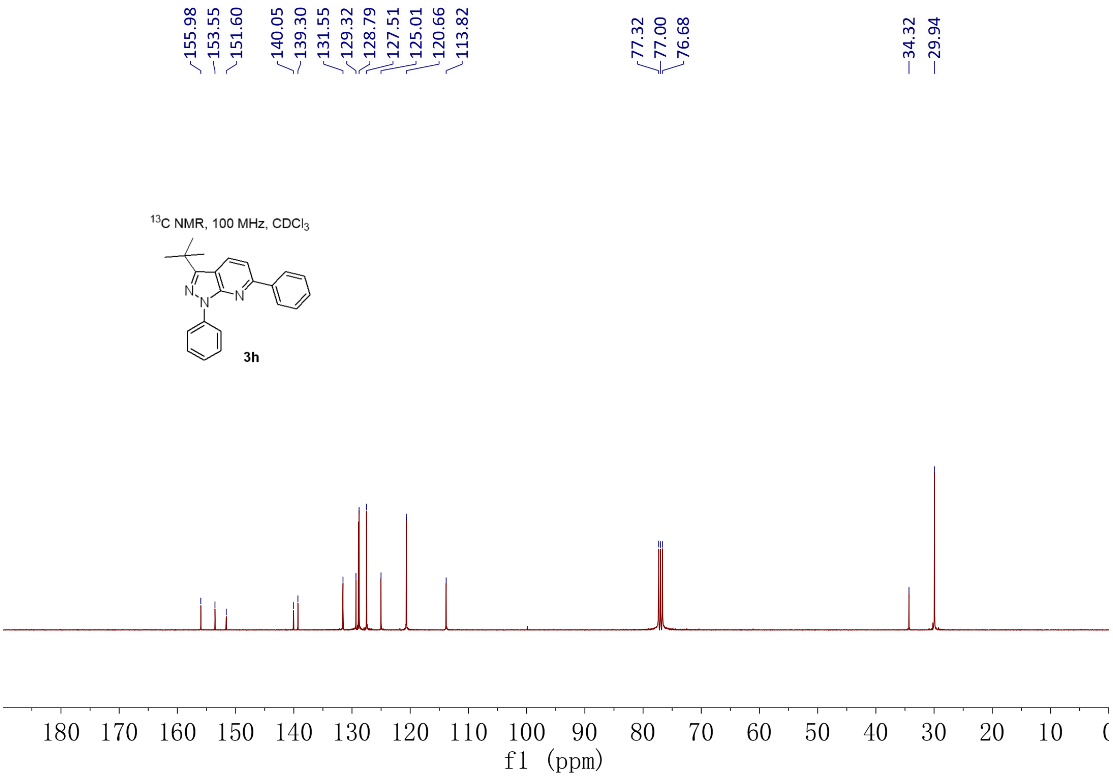
155.98 153.55 151.60 140.05 139.30 131.55 129.32 128.79 127.51 125.01 120.66 113.82 77.32 77.00 76.68 34.32 29.94
13C NMR, 100 MHz, CDCl3
3h
180 170 160 150 140 130 120 110 100 90 80 70 60 50 40 30 20 10
f1 (ppm)

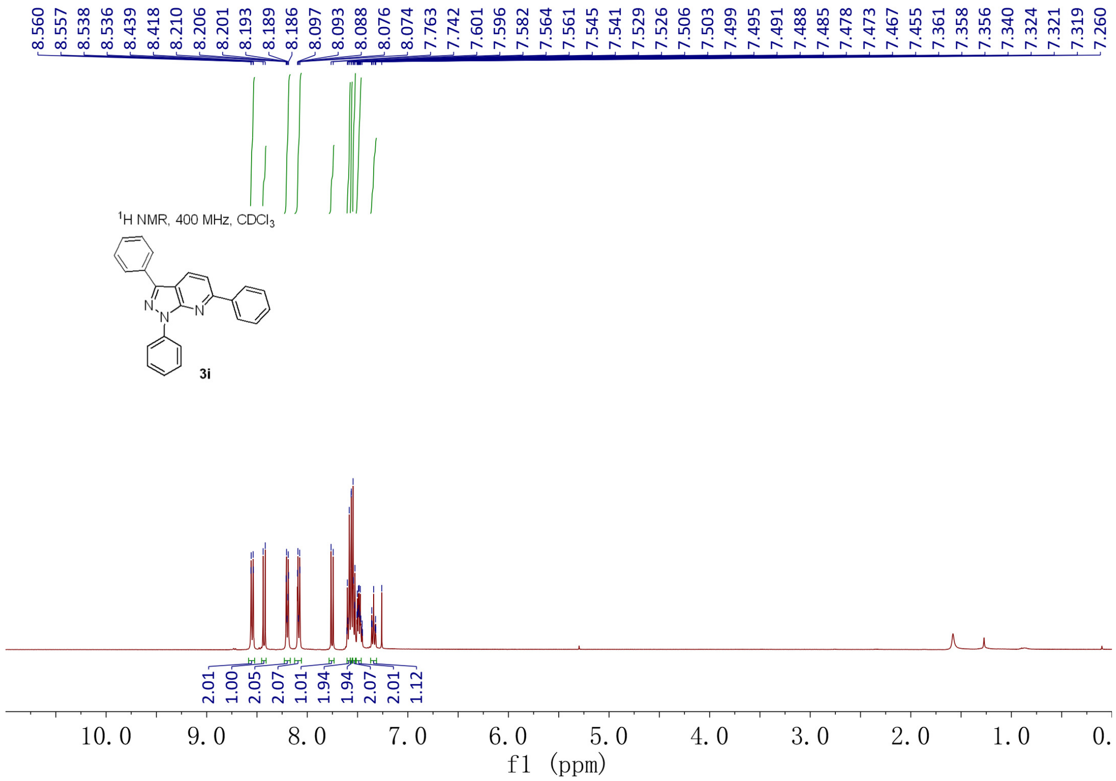
8.560 8.557 8.538 8.536 8.439 8.418 8.210 8.206 8.201 8.193 8.189 8.186 8.097 8.093 8.088 8.076 8.074 7.763 7.742 7.601 7.596 7.582 7.564 7.561 7.545 7.541 7.529 7.526 7.506 7.503 7.499 7.495 7.491 7.488 7.485 7.478 7.473 7.467 7.455 7.361 7.358 7.356 7.340 7.324 7.321 7.319 7.260
^{1}H NMR, 400 MHz, CDCl$_3$
N
N
N
3i
2.01 1.00 2.05 2.07 1.01 1.94 1.94 2.07 2.01 1.12
10.0 9.0 8.0 7.0 6.0 5.0 4.0 3.0 2.0 1.0 0.
f1 (ppm)

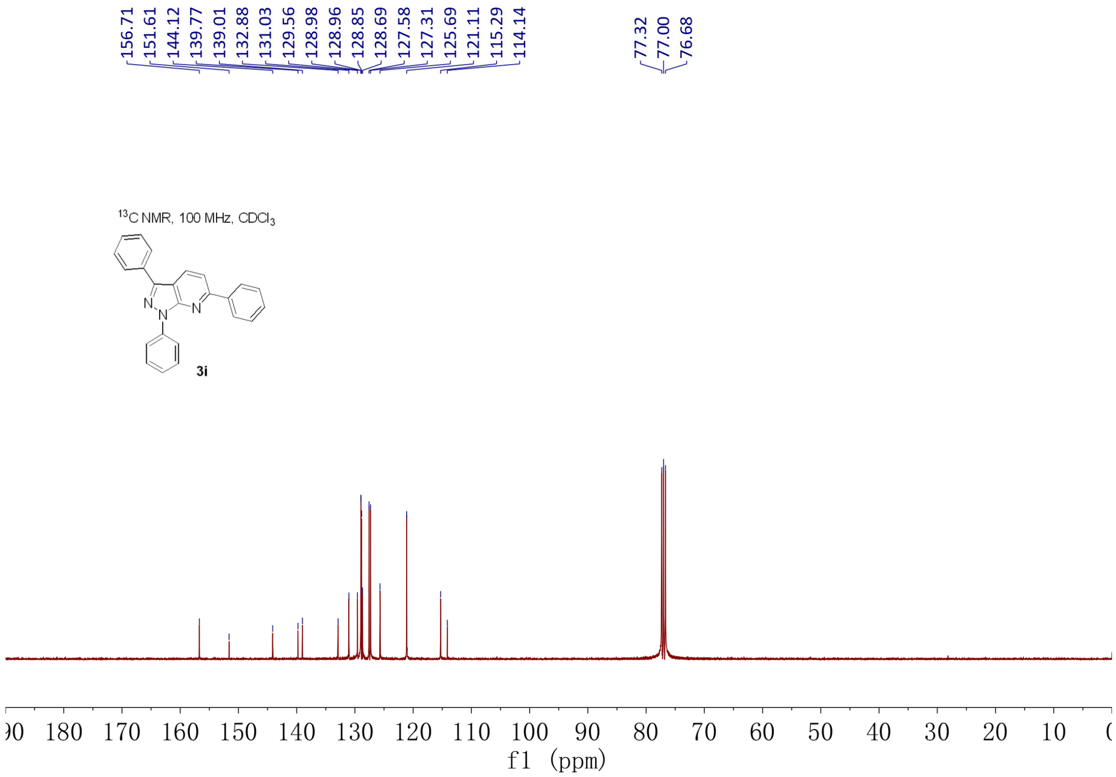
156.71 151.61 144.12 139.77 139.01 132.88 131.03 129.56 128.98 128.96 128.85 128.69 127.58 127.31 125.69 121.11 115.29 114.14
77.32 77.00 76.68
^{13}C NMR, 100 MHz, CDCl$_3$
N
N
N
3i
180 170 160 150 140 130 120 110 100 90 80 70 60 50 40 30 20 10
f1 (ppm)

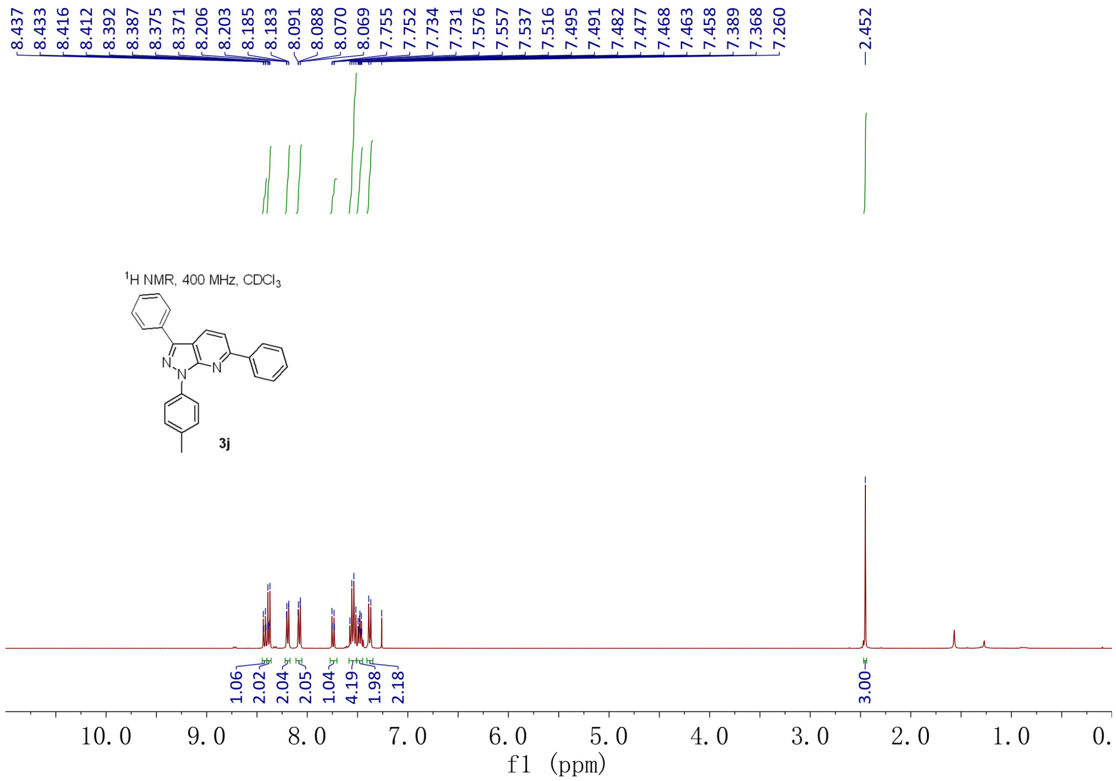
8.437 8.433 8.416 8.412 8.392 8.387 8.375 8.371 8.206 8.203 8.185 8.183 8.091 8.088 8.070 8.069 7.755 7.752 7.734 7.731 7.576 7.557 7.537 7.516 7.495 7.491 7.482 7.477 7.468 7.463 7.458 7.389 7.368 7.260
2.452
1H NMR, 400 MHz, CDCl3
N
N
N
3j
1.06 2.02 2.04 2.05 1.04 4.19 1.98 2.18
3.00
10.0 9.0 8.0 7.0 6.0 5.0 4.0 3.0 2.0 1.0
f1 (ppm)

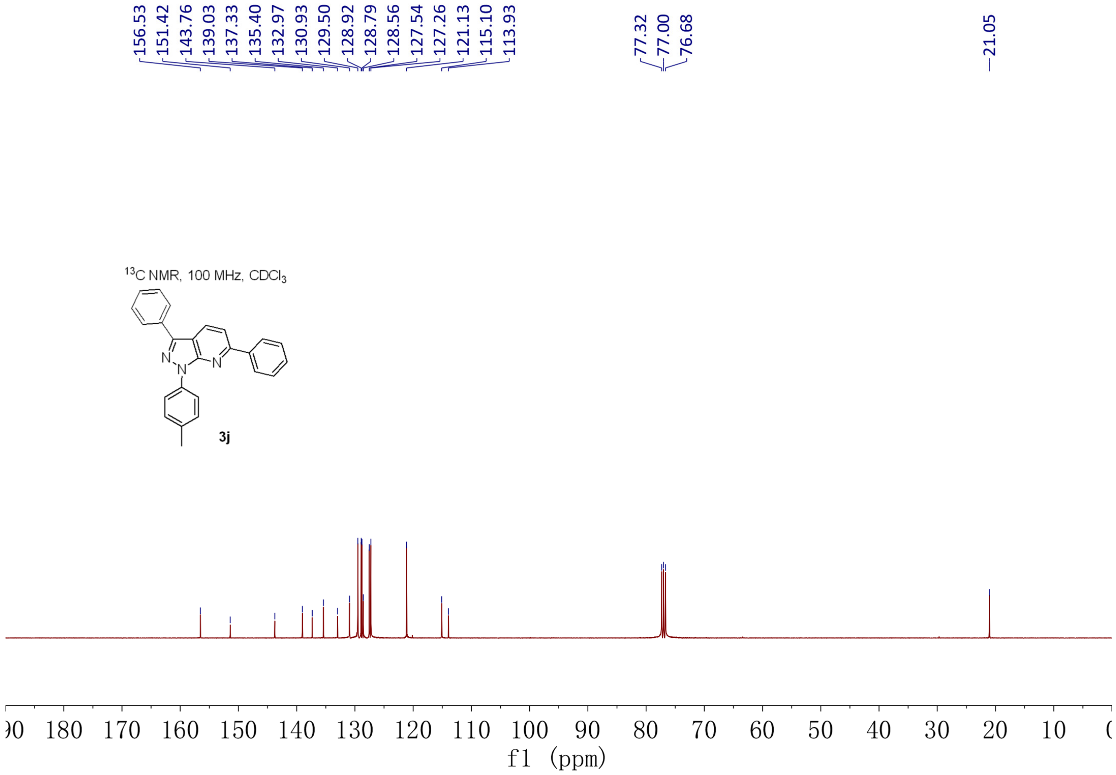
156.53 151.42 143.76 139.03 137.33 135.40 132.97 130.93 129.50 128.92 128.79 128.56 127.54 127.26 121.13 115.10 113.93
77.32 77.00 76.68
21.05
13C NMR, 100 MHz, CDCl3
N
N
N
3j
180 170 160 150 140 130 120 110 100 90 80 70 60 50 40 30 20 10
f1 (ppm)

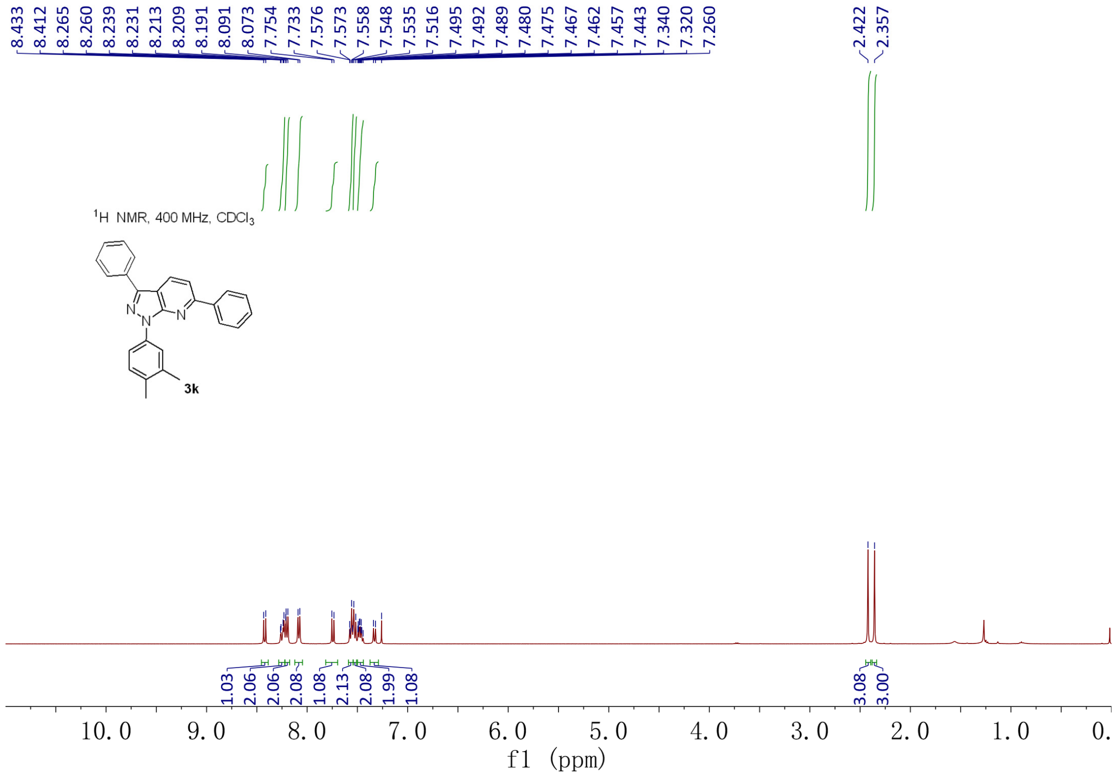
8.433 8.412 8.265 8.260 8.239 8.231 8.213 8.209 8.191 8.091 8.073 7.754 7.733 7.576 7.573 7.558 7.548 7.535 7.516 7.495 7.492 7.489 7.480 7.475 7.467 7.462 7.457 7.443 7.340 7.320 7.260
2.422 2.357
^{1}H NMR, 400 MHz, CDCl$_3$
N
N
N
3k
1.03 2.06 2.06 2.08 1.08 2.13 2.08 1.99 1.08
3.08 3.00
10.0 9.0 8.0 7.0 6.0 5.0 4.0 3.0 2.0 1.0 0.
f1 (ppm)

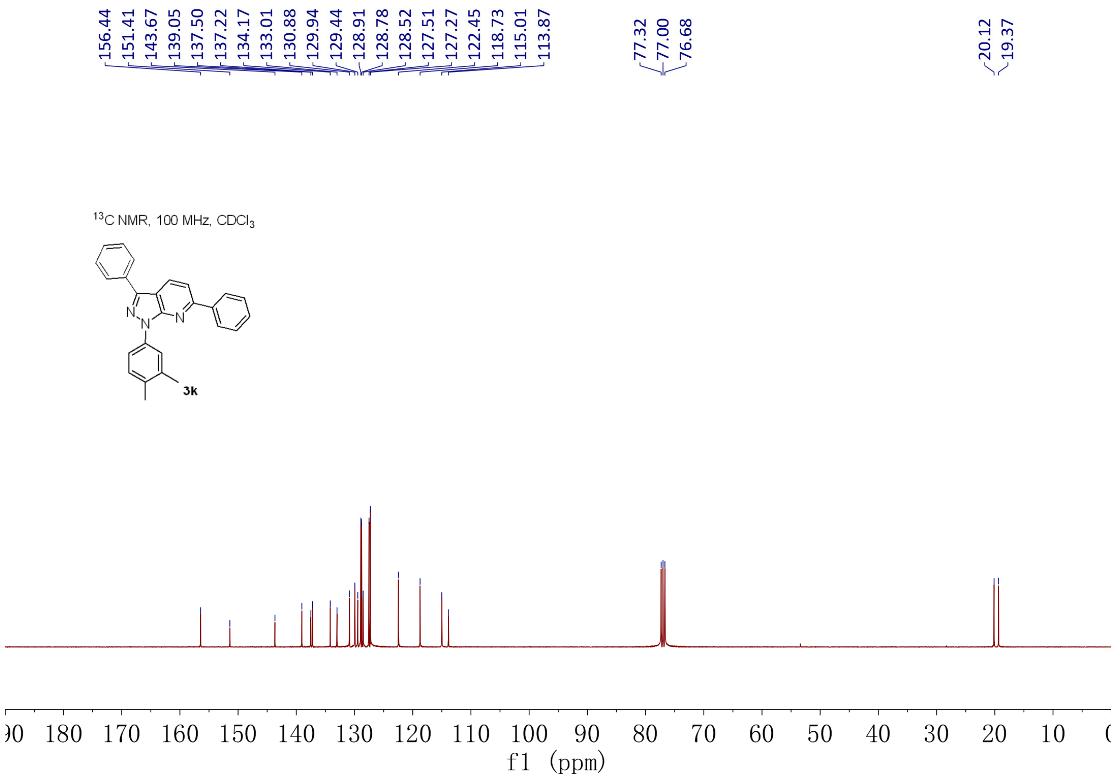
156.44 151.41 143.67 139.05 137.50 137.22 134.17 133.01 130.88 129.94 129.44 128.91 128.78 128.52 127.51 127.27 122.45 118.73 115.01 113.87
77.32 77.00 76.68
20.12 19.37
^{13}C NMR, 100 MHz, CDCl$_3$
N
N
N
3k
180 170 160 150 140 130 120 110 100 90 80 70 60 50 40 30 20 10
f1 (ppm)

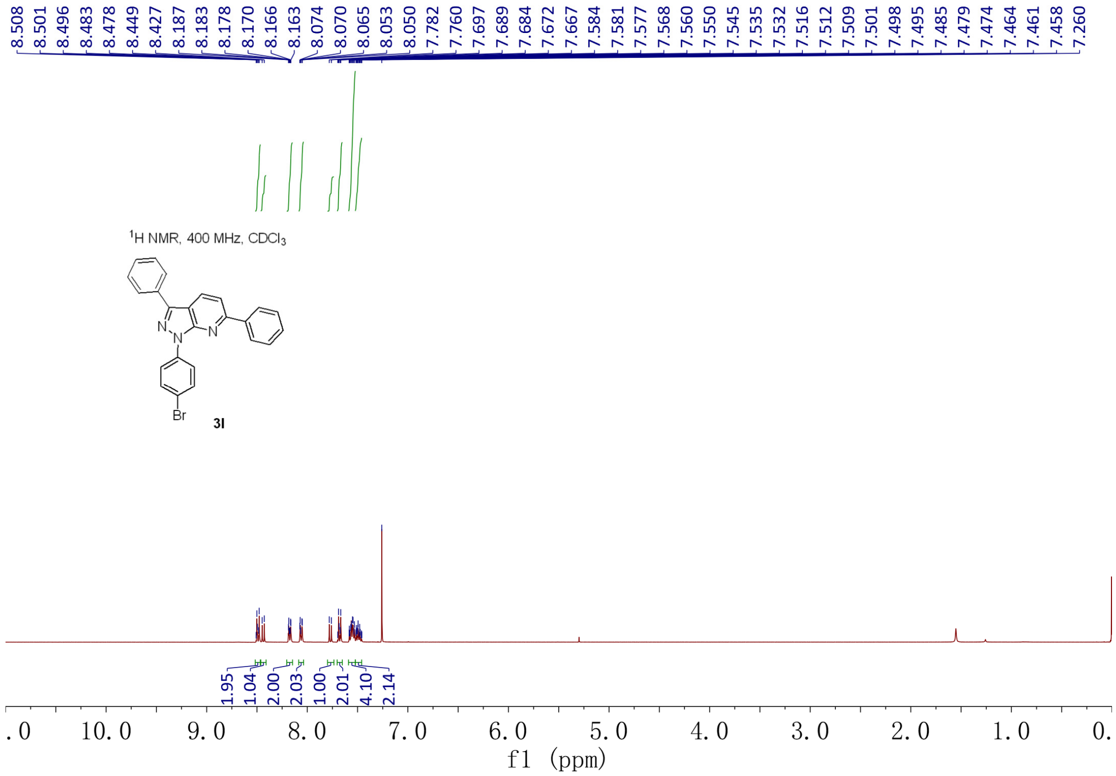
8.508 8.501 8.496 8.483 8.478 8.449 8.427 8.187 8.183 8.178 8.170 8.166 8.163 8.074 8.070 8.065 8.053 8.050 7.782 7.760 7.697 7.689 7.684 7.672 7.667 7.584 7.581 7.577 7.568 7.560 7.550 7.545 7.535 7.532 7.516 7.512 7.509 7.501 7.498 7.495 7.485 7.479 7.474 7.464 7.461 7.458 7.260
^{1}H NMR, 400 MHz, CDCl$_3$
N
N
N
Br
3l
1.95 1.04 2.00 2.03 1.00 2.01 4.10 2.14
10.0 9.0 8.0 7.0 6.0 5.0 4.0 3.0 2.0 1.0
f1 (ppm)

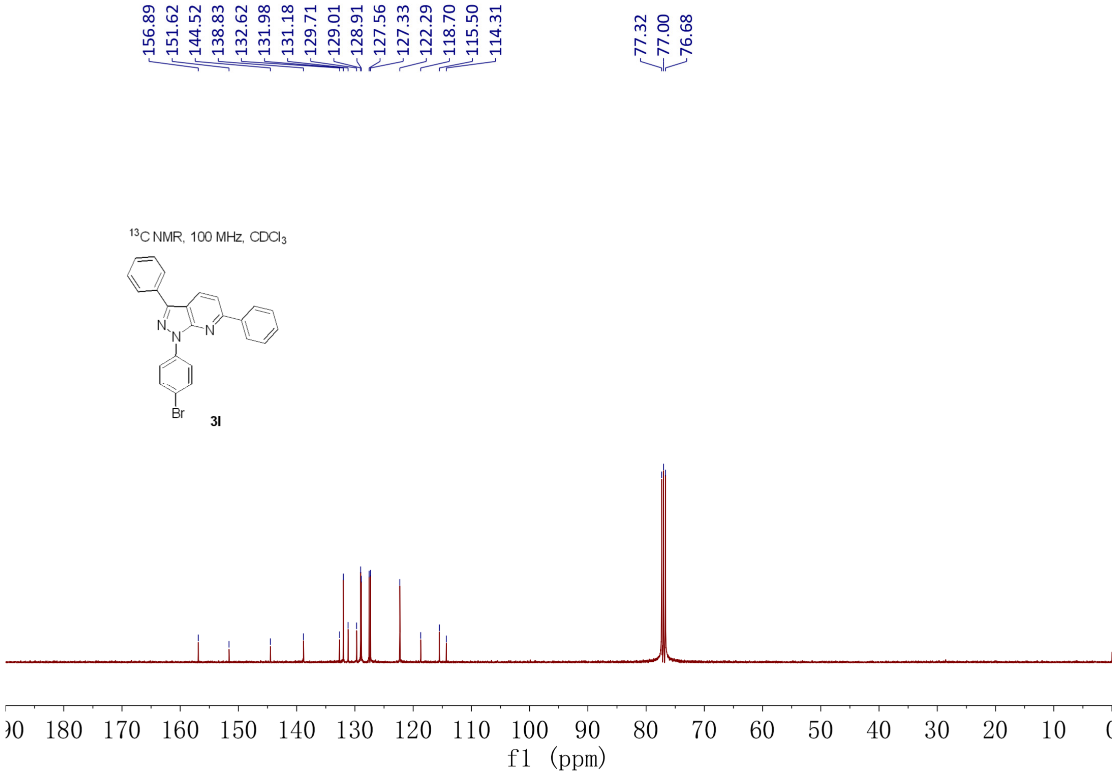
156.89 151.62 144.52 138.83 132.62 131.98 131.18 129.71 129.01 128.91 127.56 127.33 122.29 118.70 115.50 114.31
77.32 77.00 76.68
^{13}C NMR, 100 MHz, CDCl$_3$
N
N
N
Br
3l
180 170 160 150 140 130 120 110 100 90 80 70 60 50 40 30 20 10
f1 (ppm)

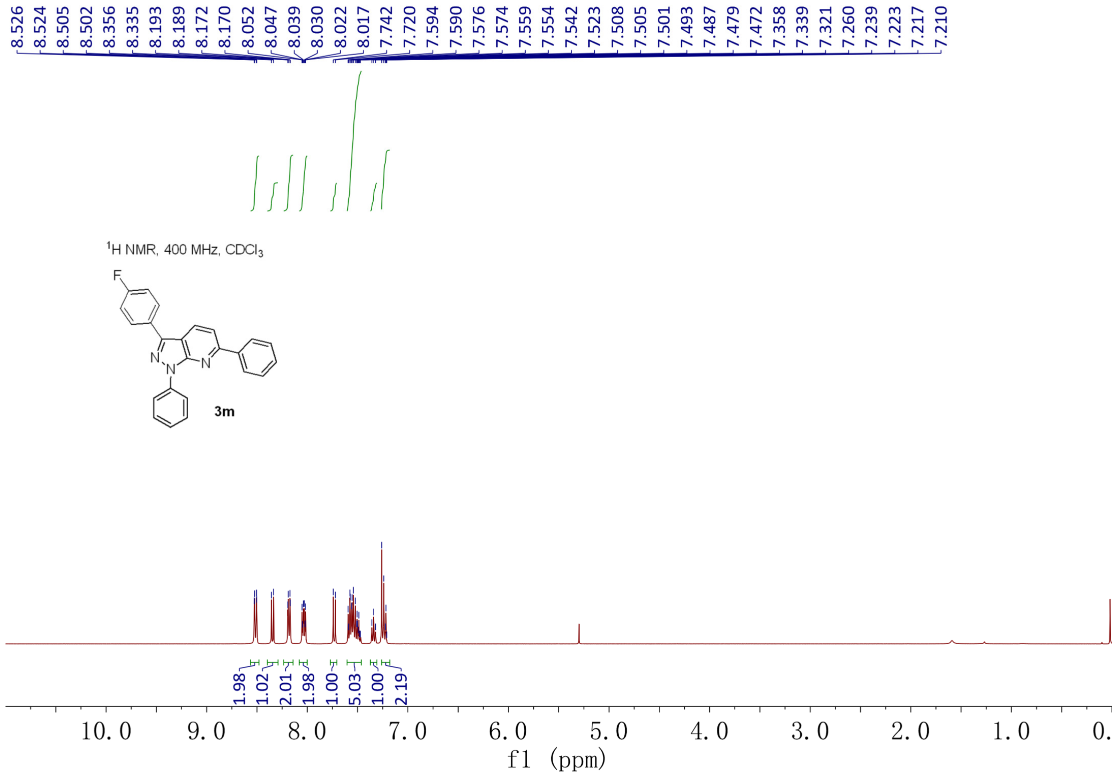
8.526 8.524 8.505 8.502 8.356 8.335 8.193 8.189 8.172 8.170 8.052 8.047 8.039 8.030 8.022 8.017 7.742 7.720 7.594 7.590 7.576 7.574 7.559 7.554 7.542 7.523 7.508 7.505 7.501 7.493 7.487 7.479 7.472 7.358 7.339 7.321 7.260 7.239 7.223 7.217 7.210
^{1}H NMR, 400 MHz, CDCl$_3$
F
N
N
N
3m
1.98 1.02 2.01 1.98 1.00 5.03 1.00 2.19
10.0 9.0 8.0 7.0 6.0 5.0 4.0 3.0 2.0 1.0 0.
f1 (ppm)

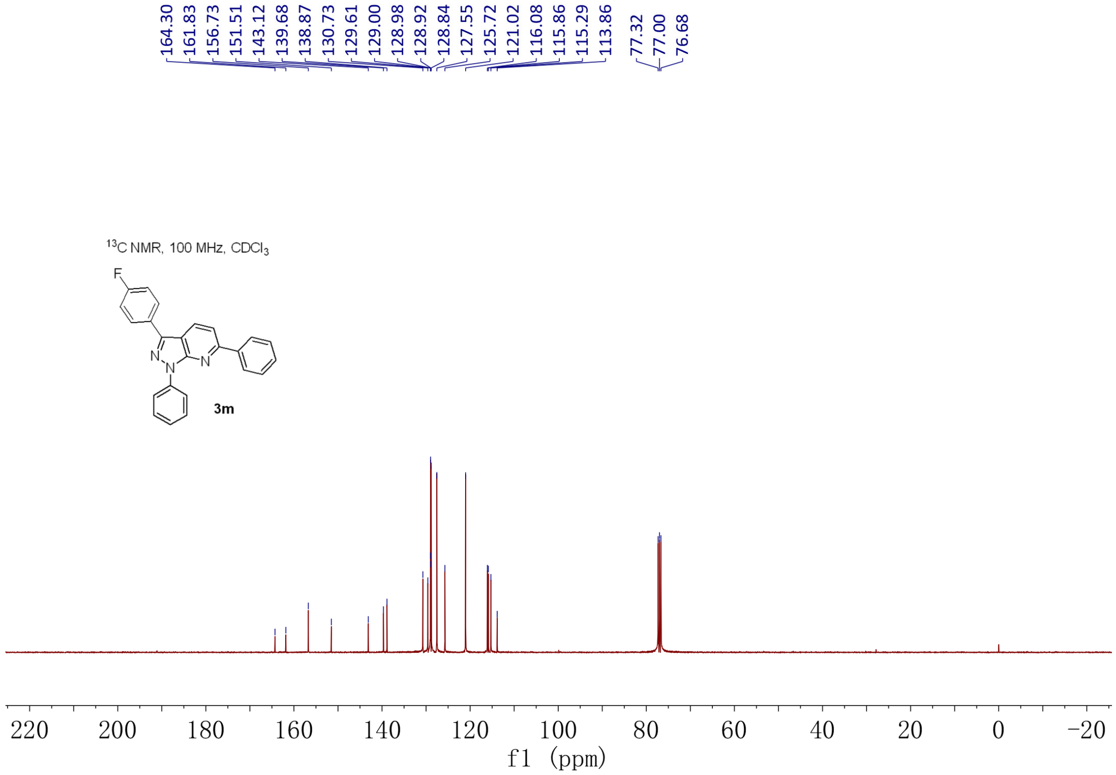
164.30 161.83 156.73 151.51 143.12 139.68 138.87 130.73 129.61 129.00 128.98 128.92 128.84 127.55 125.72 121.02 116.08 115.86 115.29 113.86 77.32 77.00 76.68
^{13}C NMR, 100 MHz, CDCl$_3$
F
N
N
N
3m
220 200 180 160 140 120 100 80 60 40 20 0 −20
f1 (ppm)

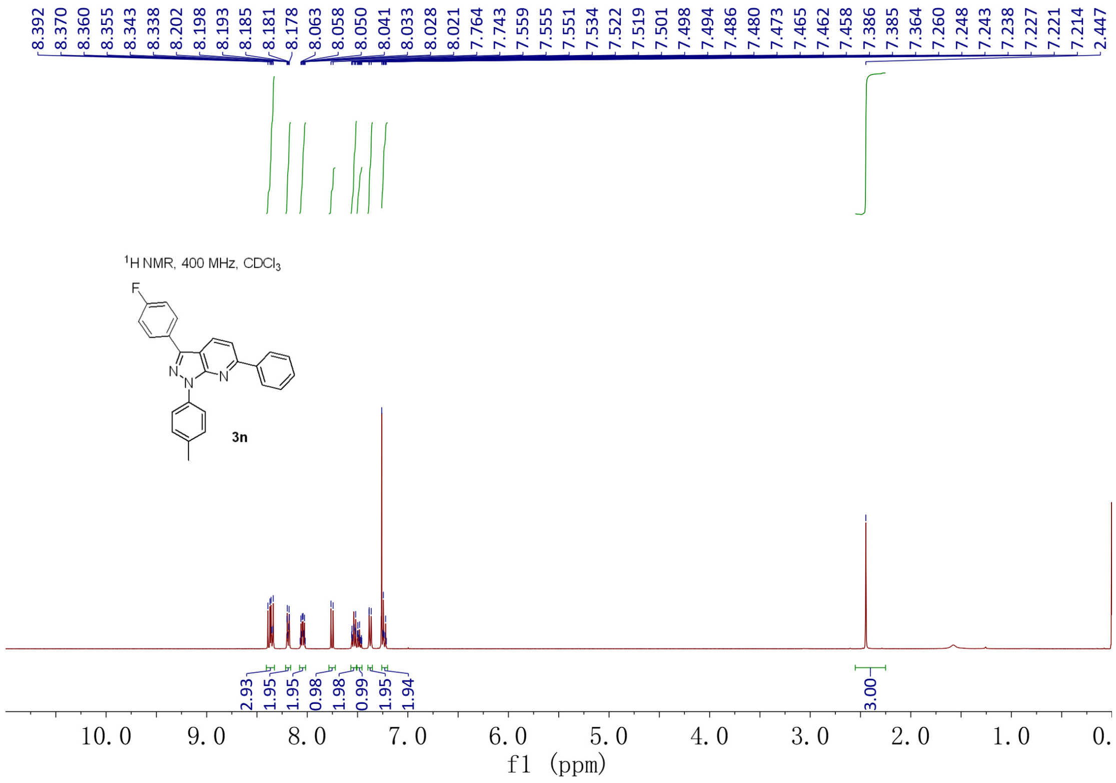
8.392 8.370 8.360 8.355 8.343 8.338 8.202 8.198 8.193 8.185 8.181 8.178 8.063 8.058 8.050 8.041 8.033 8.028 8.021 7.764 7.743 7.559 7.555 7.551 7.534 7.522 7.519 7.501 7.498 7.494 7.486 7.480 7.473 7.465 7.462 7.458 7.386 7.385 7.364 7.260 7.248 7.243 7.238 7.227 7.221 7.214 2.447
1H NMR, 400 MHz, CDCl3
F
N
N
N
3n
2.93 1.95 1.95 0.98 1.98 0.99 1.95 1.94
3.00
10.0 9.0 8.0 7.0 6.0 5.0 4.0 3.0 2.0 1.0 0.
f1 (ppm)

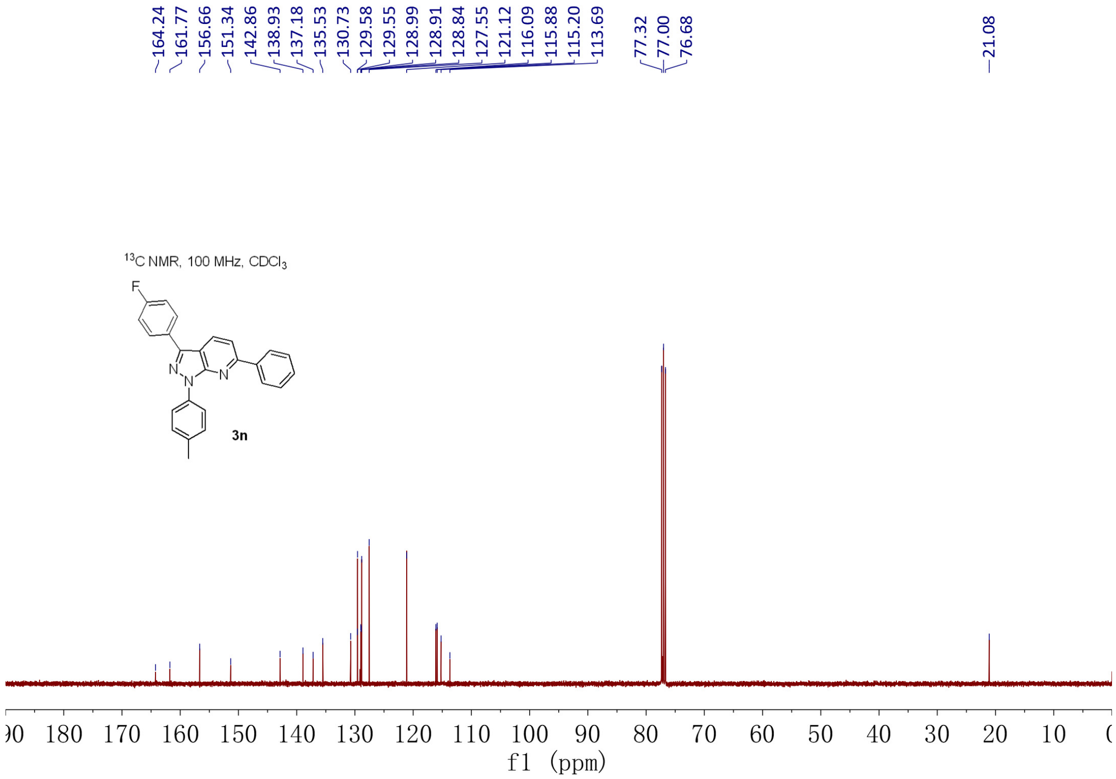
164.24 161.77 156.66 151.34 142.86 138.93 137.18 135.53 130.73 129.58 129.55 128.99 128.91 128.84 127.55 121.12 116.09 115.88 115.20 113.69
77.32 77.00 76.68
21.08
13C NMR, 100 MHz, CDCl3
F
N
N
N
3n
180 170 160 150 140 130 120 110 100 90 80 70 60 50 40 30 20 10
f1 (ppm)

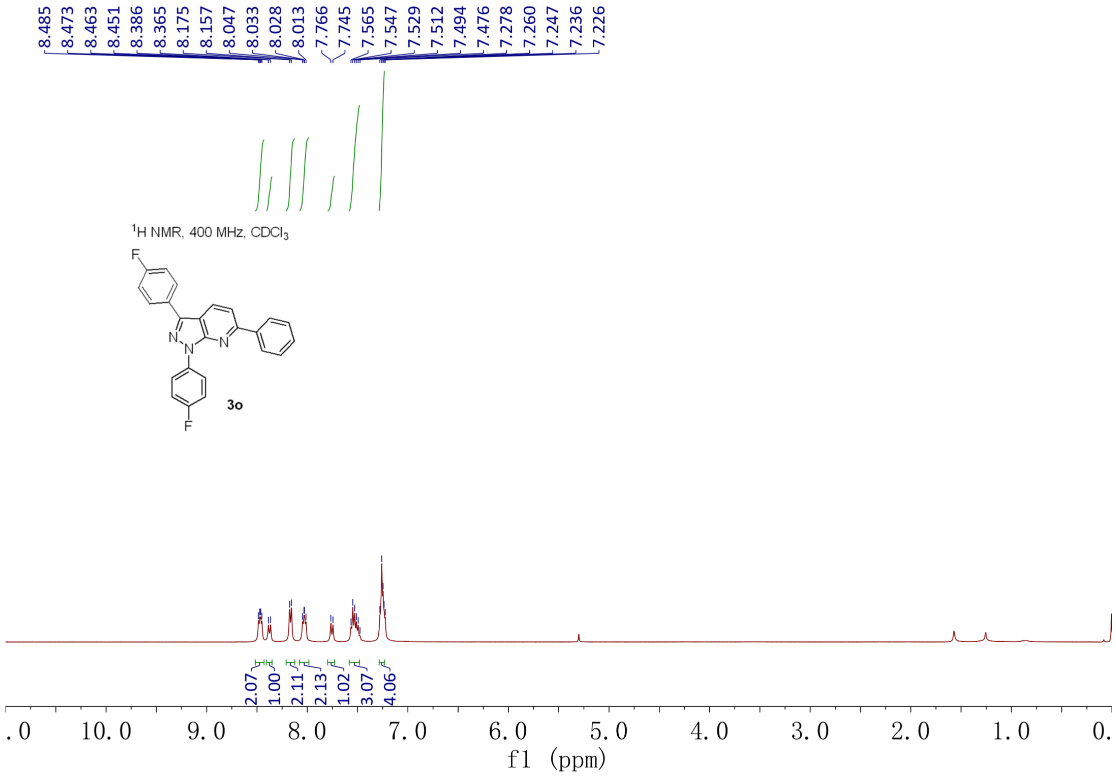
8.485
8.473
8.463
8.451
8.386
8.365
8.175
8.157
8.047
8.033
8.028
8.013
7.766
7.745
7.565
7.547
7.529
7.512
7.494
7.476
7.278
7.260
7.247
7.236
7.226
^{1}H NMR, 400 MHz, CDCl$_3$
F
N
N
N
F
3o
2.07
1.00
2.11
2.13
1.02
3.07
4.06
.0
10.0
9.0
8.0
7.0
6.0
5.0
4.0
3.0
2.0
1.0
0.
f1 (ppm)

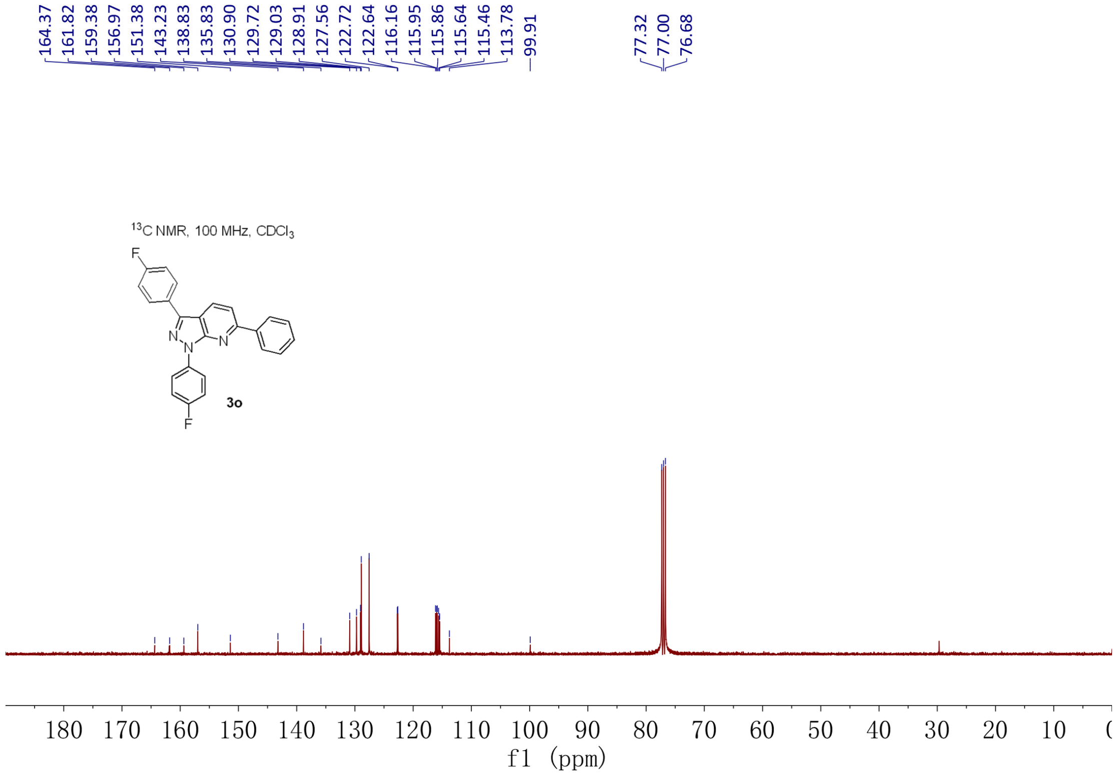
164.37
161.82
159.38
156.97
151.38
143.23
138.83
135.83
130.90
129.72
129.03
128.91
127.56
122.72
122.64
116.16
115.95
115.86
115.64
115.46
113.78
99.91
77.32
77.00
76.68
^{13}C NMR, 100 MHz, CDCl$_3$
F
N
N
N
F
3o
180
170
160
150
140
130
120
110
100
90
80
70
60
50
40
30
20
10
f1 (ppm)

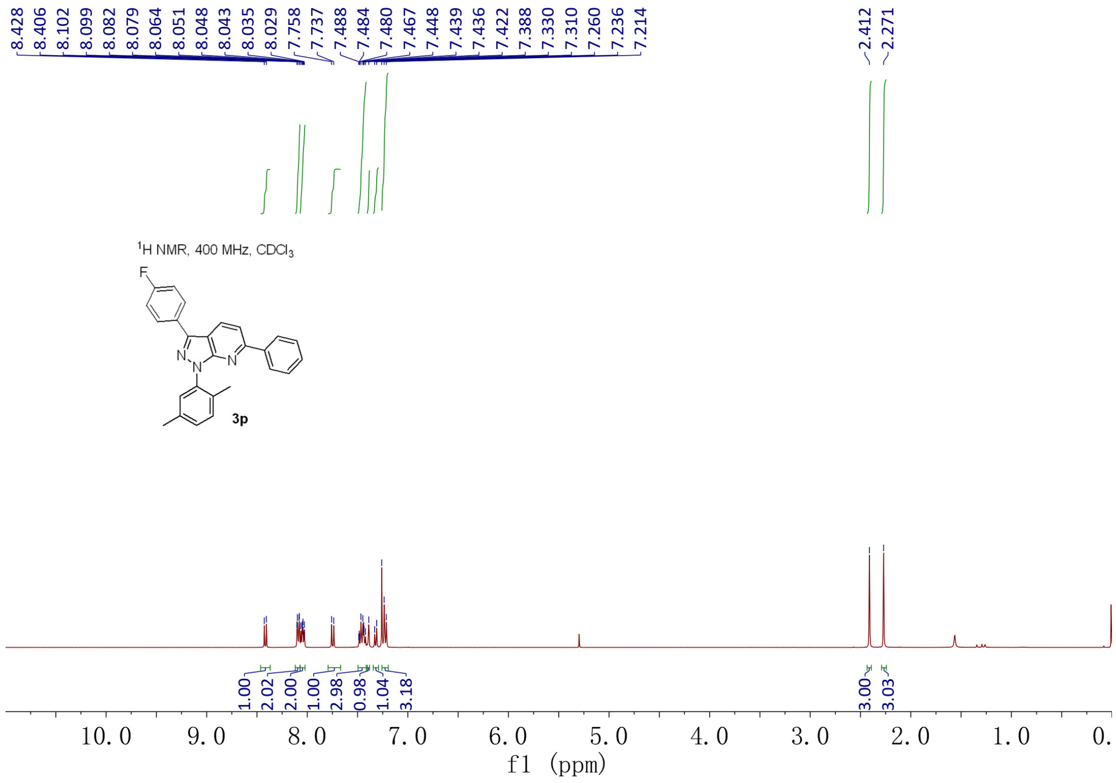
8.428 8.406 8.102 8.099 8.082 8.079 8.064 8.051 8.048 8.043 8.035 8.029 7.758 7.737 7.488 7.484 7.480 7.467 7.448 7.439 7.436 7.422 7.388 7.330 7.310 7.260 7.236 7.214
2.412 2.271
1H NMR, 400 MHz, CDCl3
F
N
N
N
3p
1.00 2.02 2.00 1.00 2.98 0.98 1.04 3.18
3.00 3.03
10.0 9.0 8.0 7.0 6.0 5.0 4.0 3.0 2.0 1.0 0.
f1 (ppm)

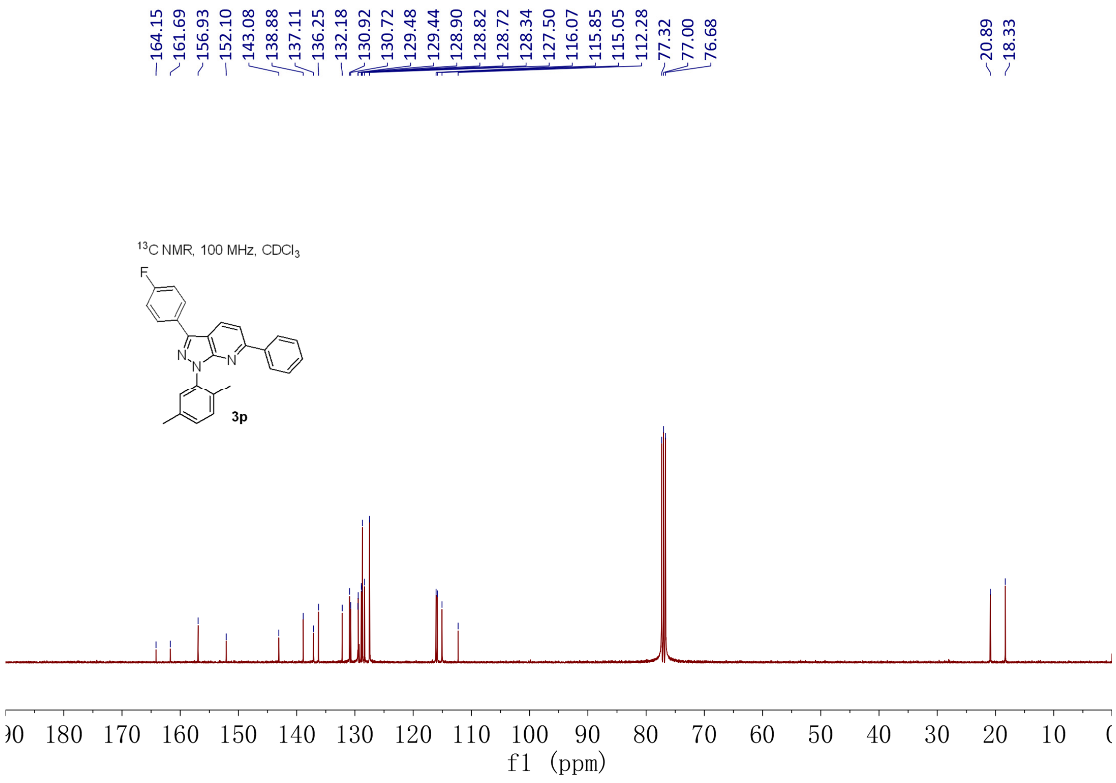
164.15 161.69 156.93 152.10 143.08 138.88 137.11 136.25 132.18 130.92 130.72 129.48 129.44 128.90 128.82 128.72 128.34 127.50 116.07 115.85 115.05 112.28 77.32 77.00 76.68
20.89 18.33
13C NMR, 100 MHz, CDCl3
F
N
N
N
3p
180 170 160 150 140 130 120 110 100 90 80 70 60 50 40 30 20 10
f1 (ppm)

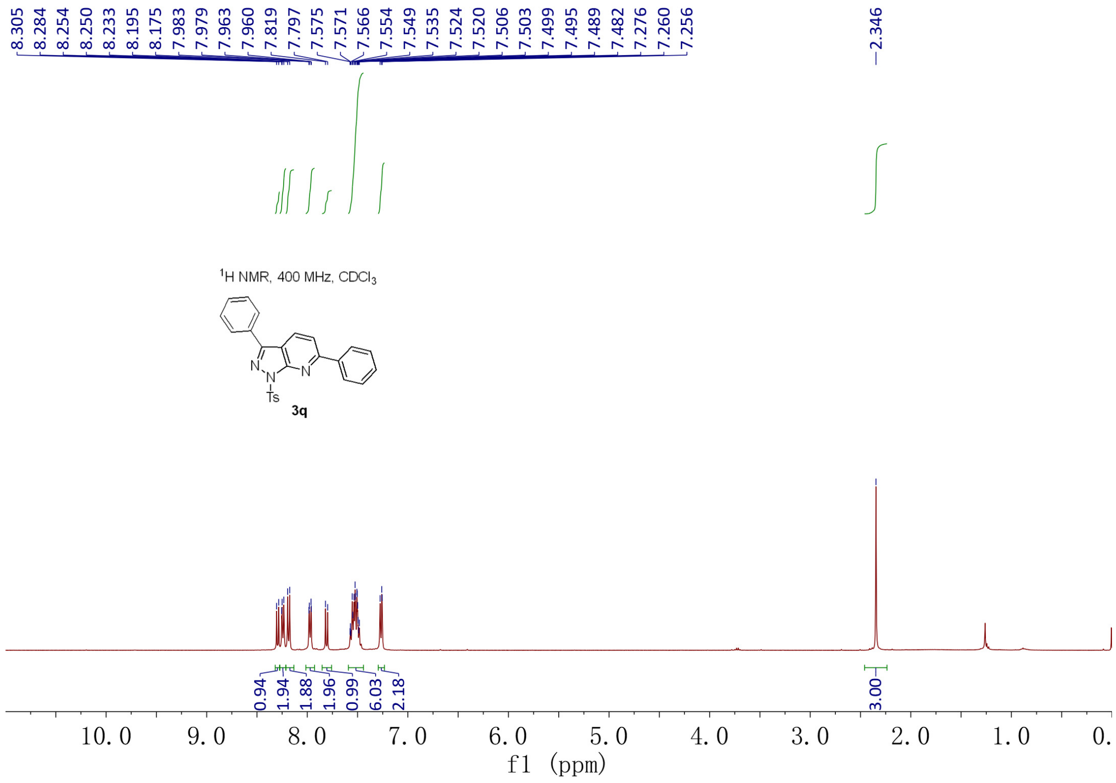
8.305
8.284
8.254
8.250
8.233
8.195
8.175
7.983
7.979
7.963
7.960
7.819
7.797
7.575
7.571
7.566
7.554
7.549
7.535
7.524
7.520
7.506
7.503
7.499
7.495
7.489
7.482
7.276
7.260
7.256
2.346
^{1}H NMR, 400 MHz, CDCl$_3$
N
N
N
Ts
3q
0.94
1.94
1.88
1.96
0.99
6.03
2.18
3.00
10.0
9.0
8.0
7.0
6.0
5.0
4.0
3.0
2.0
1.0
0.
f1 (ppm)

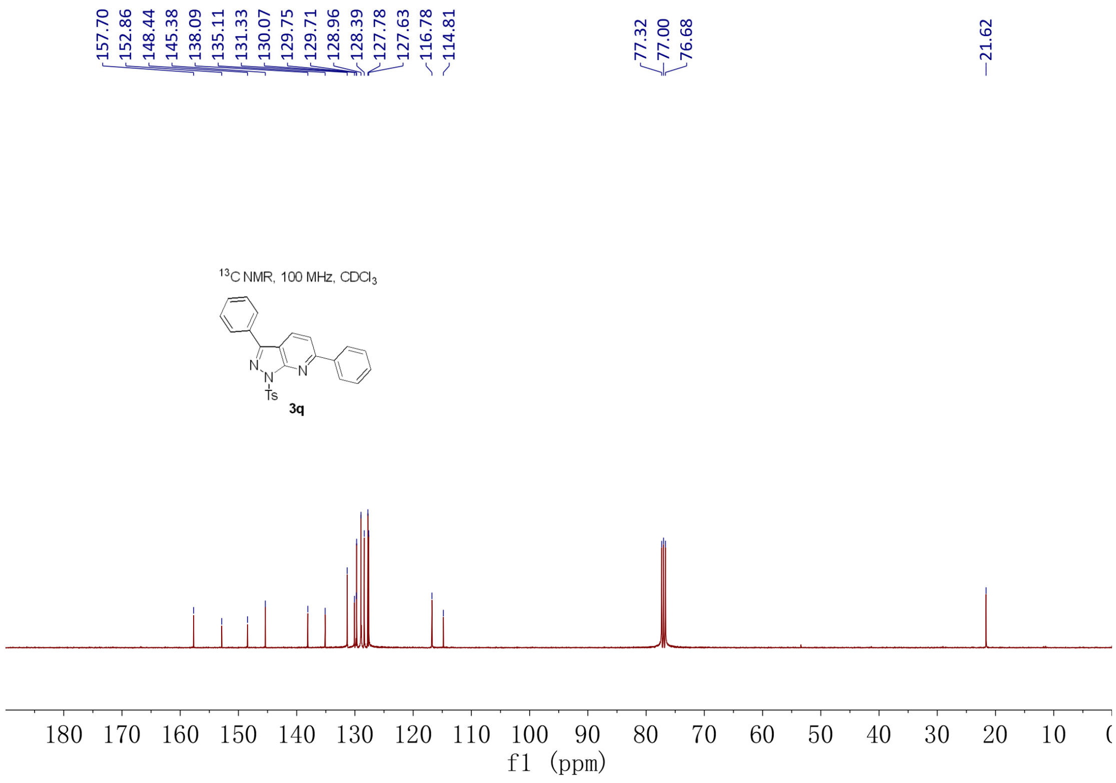
157.70
152.86
148.44
145.38
138.09
135.11
131.33
130.07
129.75
129.71
128.96
128.39
127.78
127.63
116.78
114.81
77.32
77.00
76.68
21.62
^{13}C NMR, 100 MHz, CDCl$_3$
N
N
N
Ts
3q
180
170
160
150
140
130
120
110
100
90
80
70
60
50
40
30
20
10
f1 (ppm)

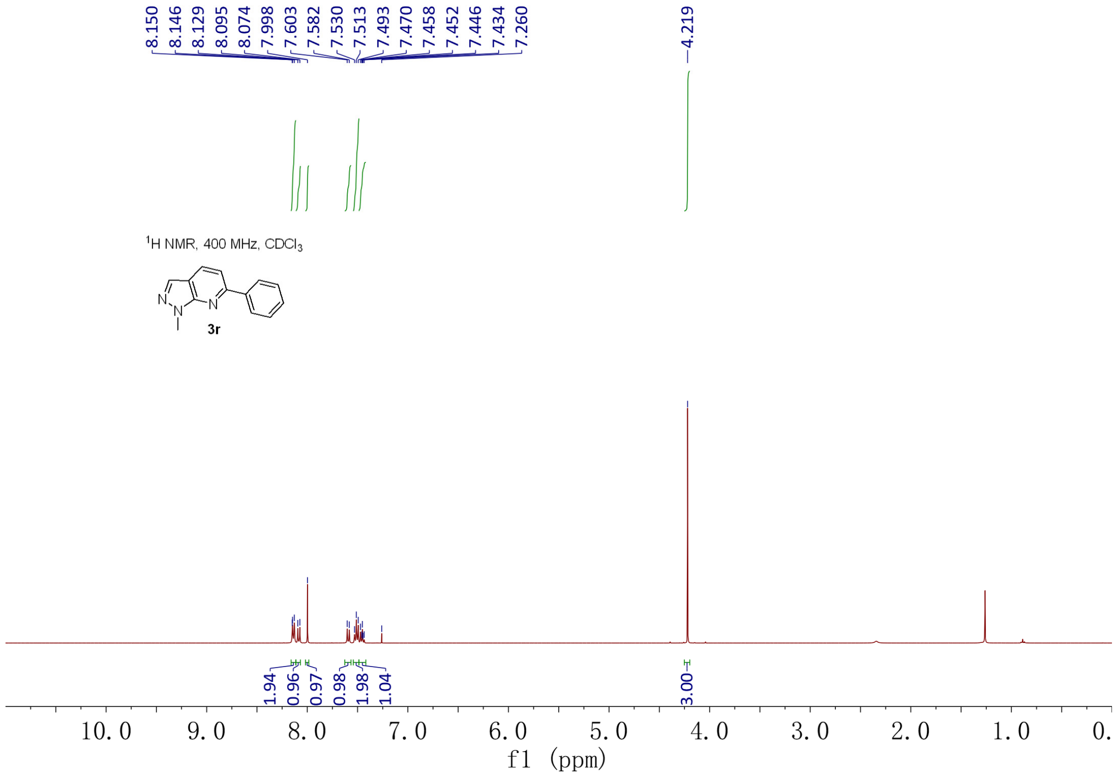
8.150
8.146
8.129
8.095
8.074
7.998
7.603
7.582
7.530
7.513
7.493
7.470
7.458
7.452
7.446
7.434
7.260
4.219
1H NMR, 400 MHz, CDCl3
N
N
N
3r
1.94
0.96
0.97
0.98
1.98
1.04
3.00
10.0
9.0
8.0
7.0
6.0
5.0
4.0
3.0
2.0
1.0
0.
f1 (ppm)

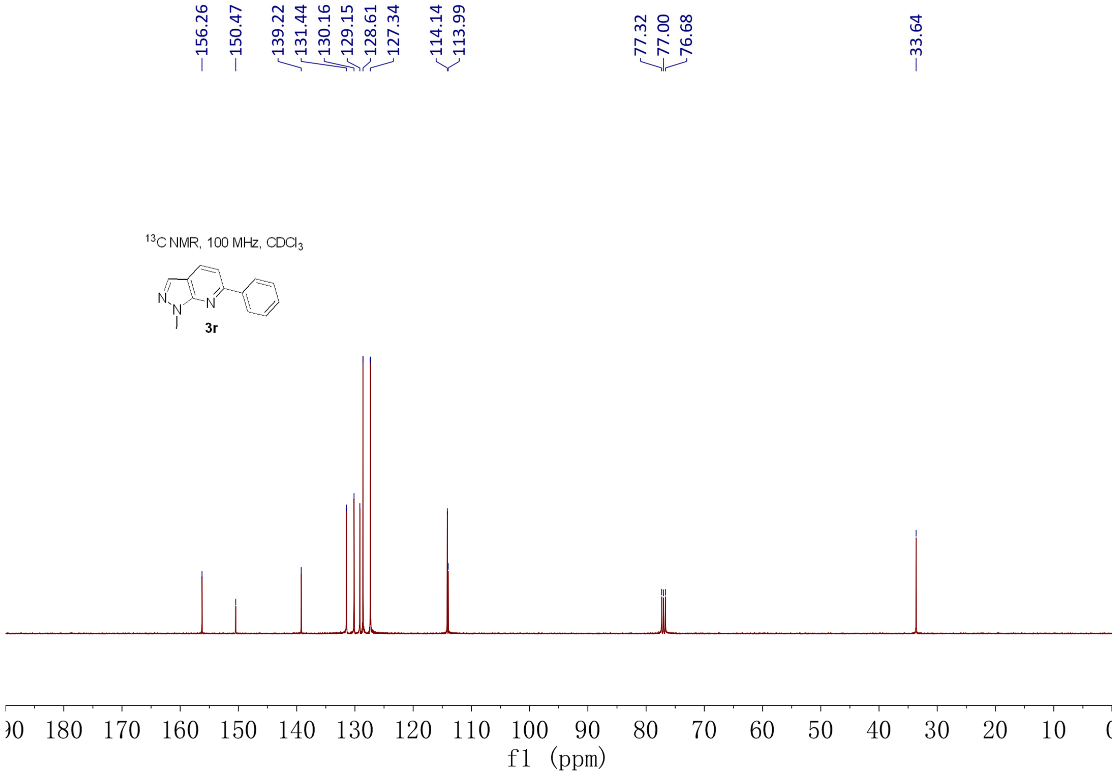
156.26
150.47
139.22
131.44
130.16
129.15
128.61
127.34
114.14
113.99
77.32
77.00
76.68
33.64
13C NMR, 100 MHz, CDCl3
N
N
N
3r
90
180
170
160
150
140
130
120
110
100
90
80
70
60
50
40
30
20
10
0
f1 (ppm)

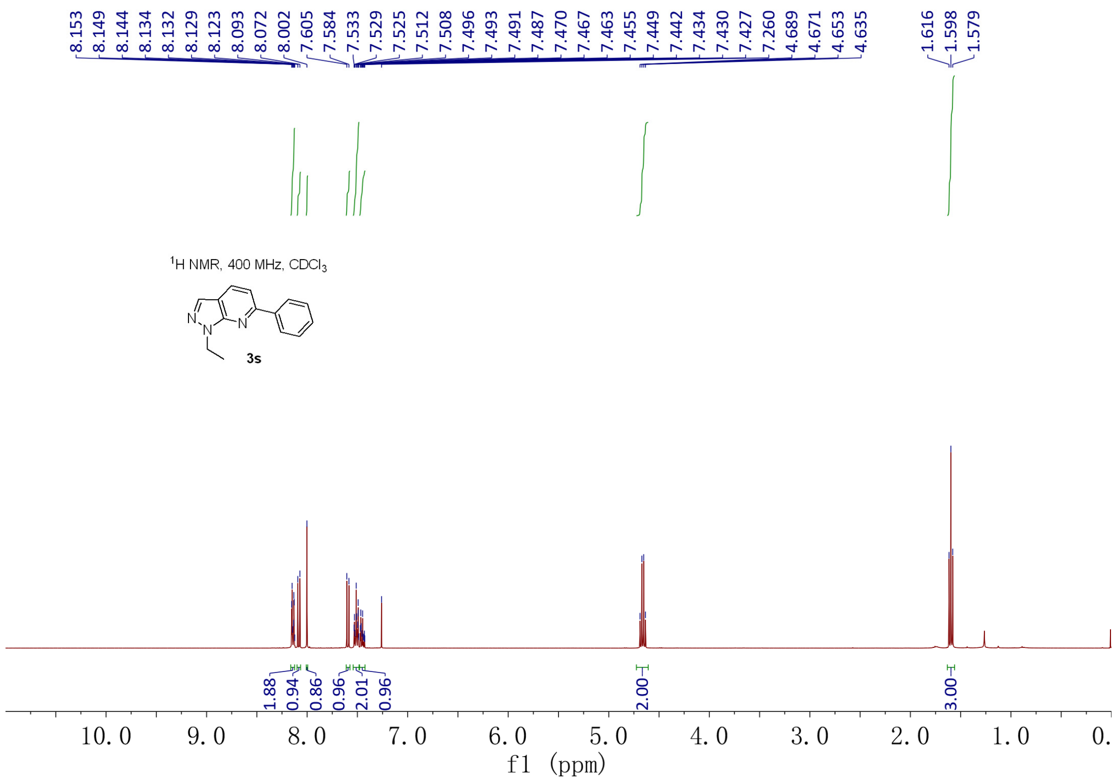

8.153 8.149 8.144 8.134 8.132 8.129 8.123 8.093 8.072 8.002 7.605 7.584 7.533 7.529 7.525 7.512 7.508 7.496 7.493 7.491 7.487 7.470 7.467 7.463 7.455 7.449 7.442 7.434 7.430 7.427 7.260 4.689 4.671 4.653 4.635 1.616 1.598 1.579
^{1}H NMR, 400 MHz, CDCl$_3$
3s
1.88 0.94 0.86 0.96 2.01 0.96 2.00 3.00
10.0 9.0 8.0 7.0 6.0 5.0 4.0 3.0 2.0 1.0 0.
f1 (ppm)

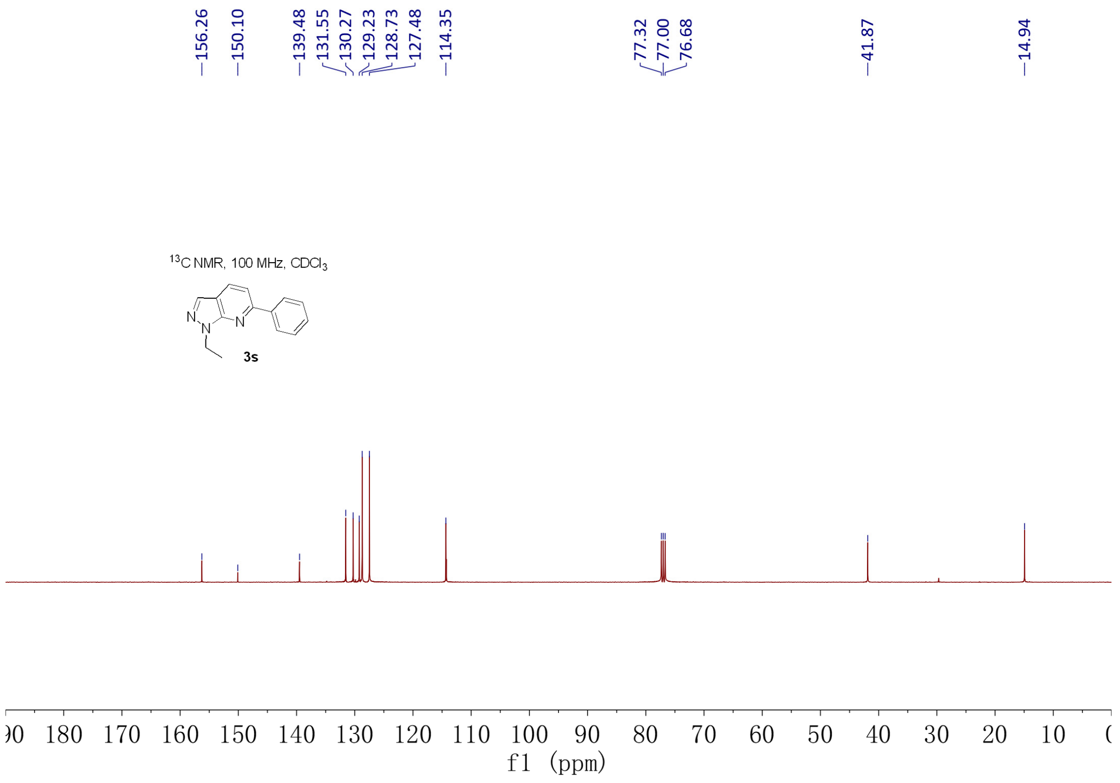

156.26 150.10 139.48 131.55 130.27 129.23 128.73 127.48 114.35 77.32 77.00 76.68 41.87 14.94
^{13}C NMR, 100 MHz, CDCl$_3$
3s
180 170 160 150 140 130 120 110 100 90 80 70 60 50 40 30 20 10
f1 (ppm)

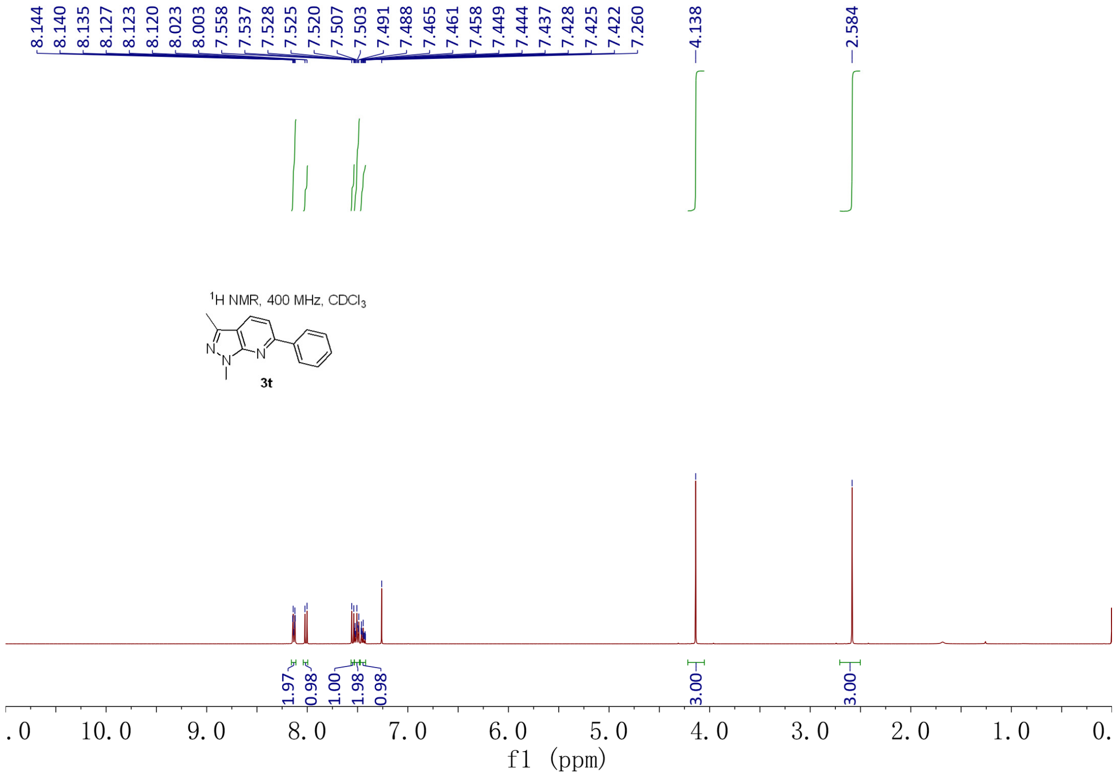
8.144
8.140
8.135
8.127
8.123
8.120
8.023
8.003
7.558
7.537
7.528
7.525
7.520
7.507
7.503
7.491
7.488
7.465
7.461
7.458
7.449
7.444
7.437
7.428
7.425
7.422
7.260
4.138
2.584
1H NMR, 400 MHz, CDCl3
N
N
N
3t
1.97
0.98
1.00
1.98
0.98
3.00
3.00
10.0
9.0
8.0
7.0
6.0
5.0
4.0
3.0
2.0
1.0
f1 (ppm)

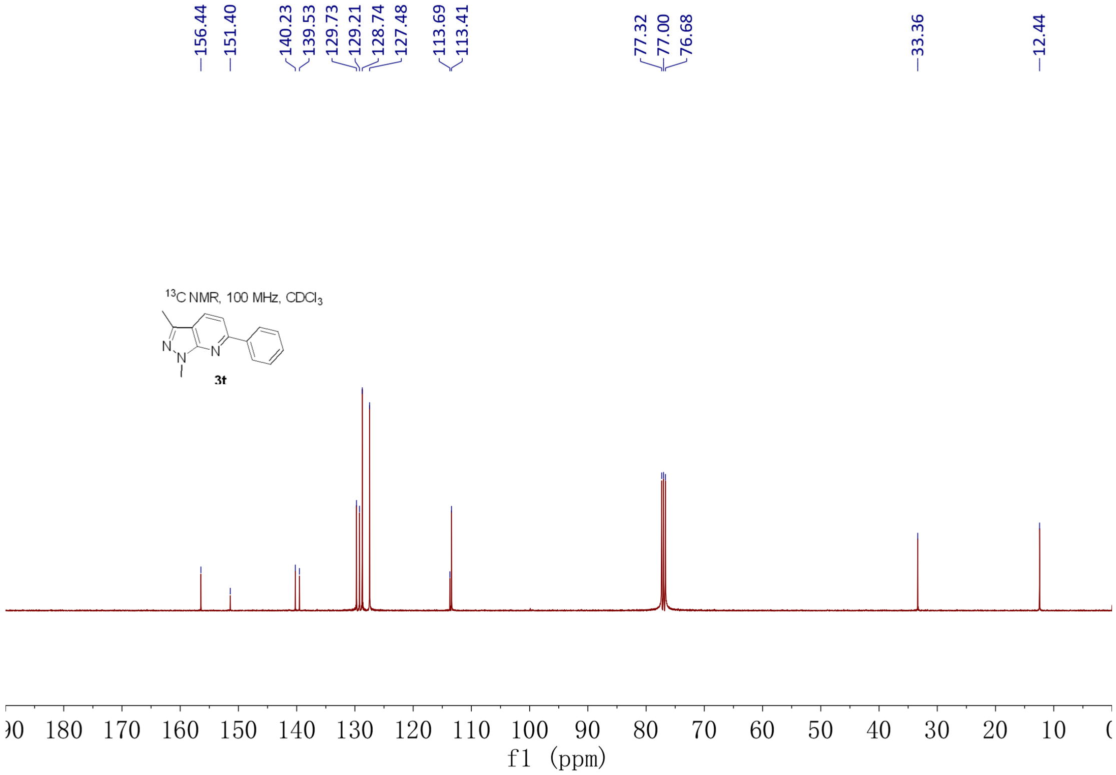
156.44
151.40
140.23
139.53
129.73
129.21
128.74
127.48
113.69
113.41
77.32
77.00
76.68
33.36
12.44
13C NMR, 100 MHz, CDCl3
N
N
N
3t
180
170
160
150
140
130
120
110
100
90
80
70
60
50
40
30
20
10
f1 (ppm)

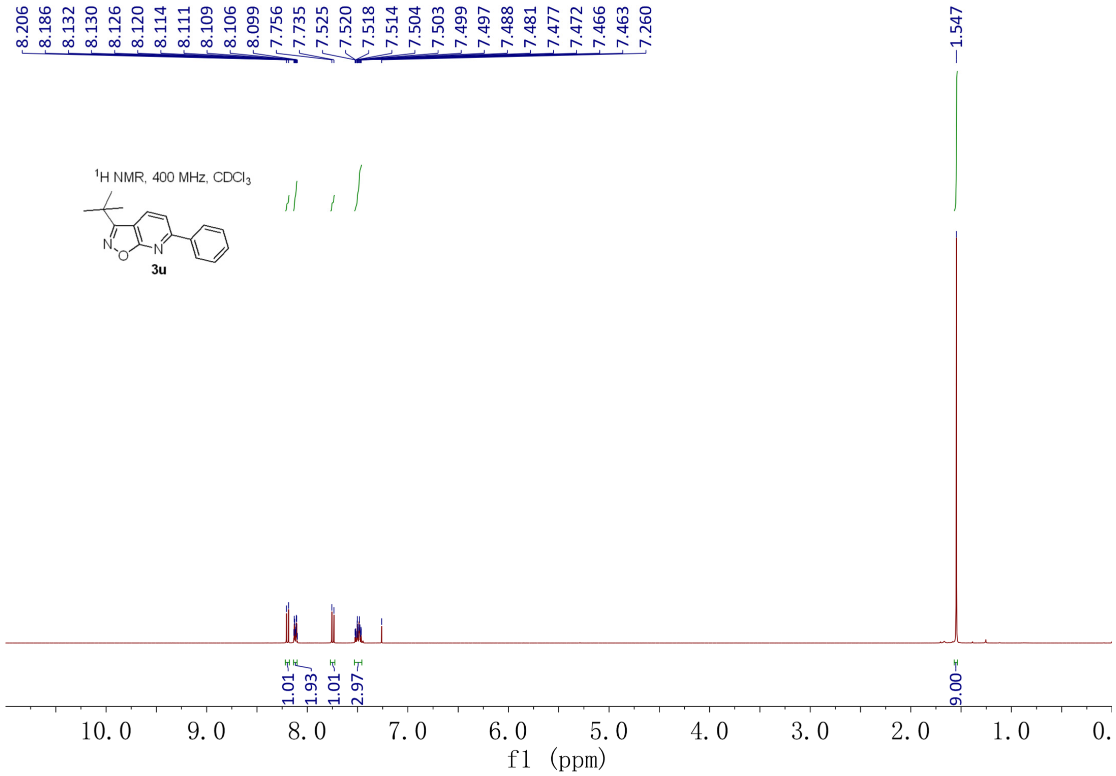
8.206
8.186
8.132
8.130
8.126
8.120
8.114
8.111
8.109
8.106
8.099
7.756
7.735
7.525
7.520
7.518
7.514
7.504
7.503
7.499
7.497
7.488
7.481
7.477
7.472
7.466
7.463
7.260
1.547
¹H NMR, 400 MHz, CDCl₃
N
O
N
3u
1.01
1.93
1.01
2.97
9.00
10.0
9.0
8.0
7.0
6.0
5.0
4.0
3.0
2.0
1.0
0.
f1 (ppm)

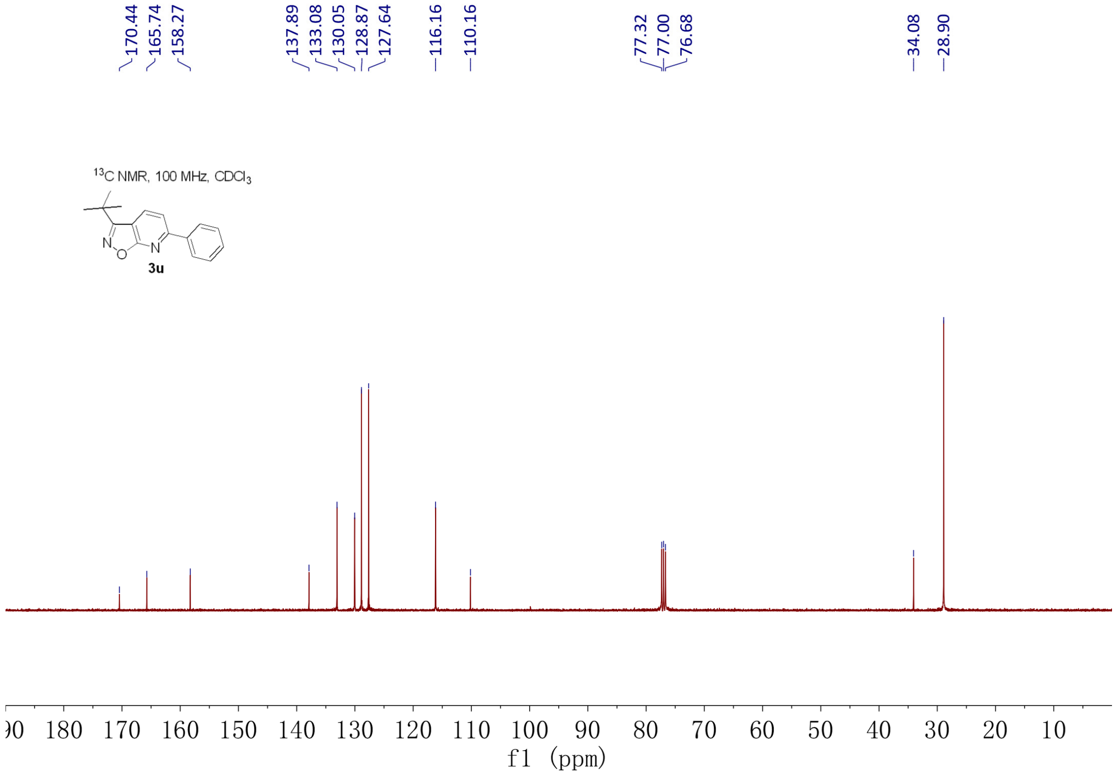
170.44
165.74
158.27
137.89
133.08
130.05
128.87
127.64
116.16
110.16
77.32
77.00
76.68
34.08
28.90
¹³C NMR, 100 MHz, CDCl₃
N
O
N
3u
180
170
160
150
140
130
120
110
100
90
80
70
60
50
40
30
20
10
f1 (ppm)

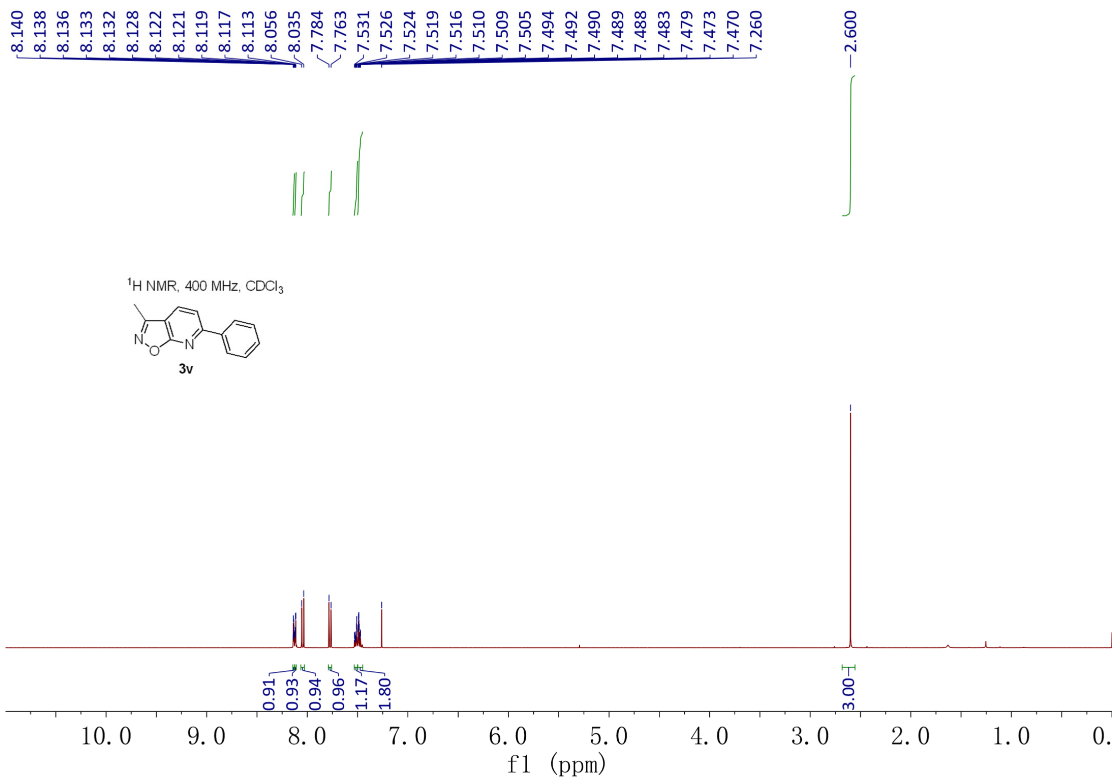

8.140 8.138 8.136 8.133 8.132 8.128 8.122 8.121 8.119 8.117 8.113 8.056 8.035 7.784 7.763 7.531 7.526 7.524 7.519 7.516 7.510 7.509 7.505 7.494 7.492 7.490 7.489 7.488 7.483 7.479 7.473 7.470 7.260
2.600
¹H NMR, 400 MHz, CDCl₃
3v
0.91 0.93 0.94 0.96 1.17 1.80
3.00
10.0 9.0 8.0 7.0 6.0 5.0 4.0 3.0 2.0 1.0 0.
f1 (ppm)

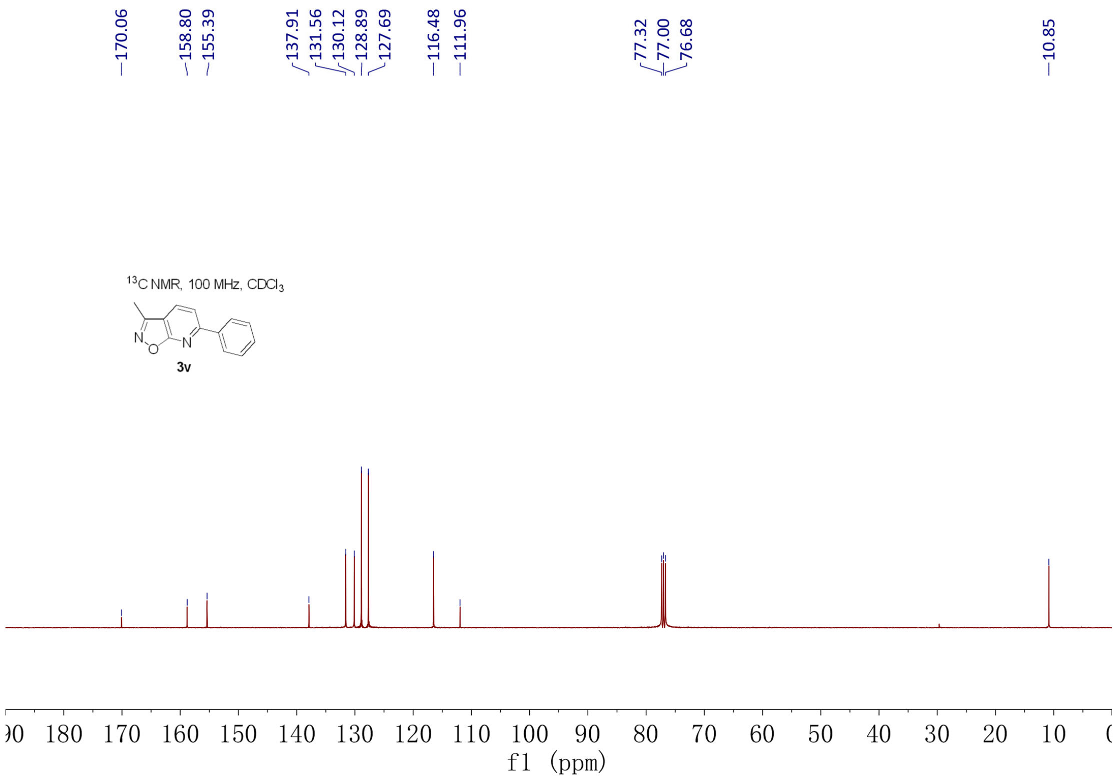

170.06 158.80 155.39 137.91 131.56 130.12 128.89 127.69 116.48 111.96 77.32 77.00 76.68 10.85
¹³C NMR, 100 MHz, CDCl₃
3v
180 170 160 150 140 130 120 110 100 90 80 70 60 50 40 30 20 10
f1 (ppm)

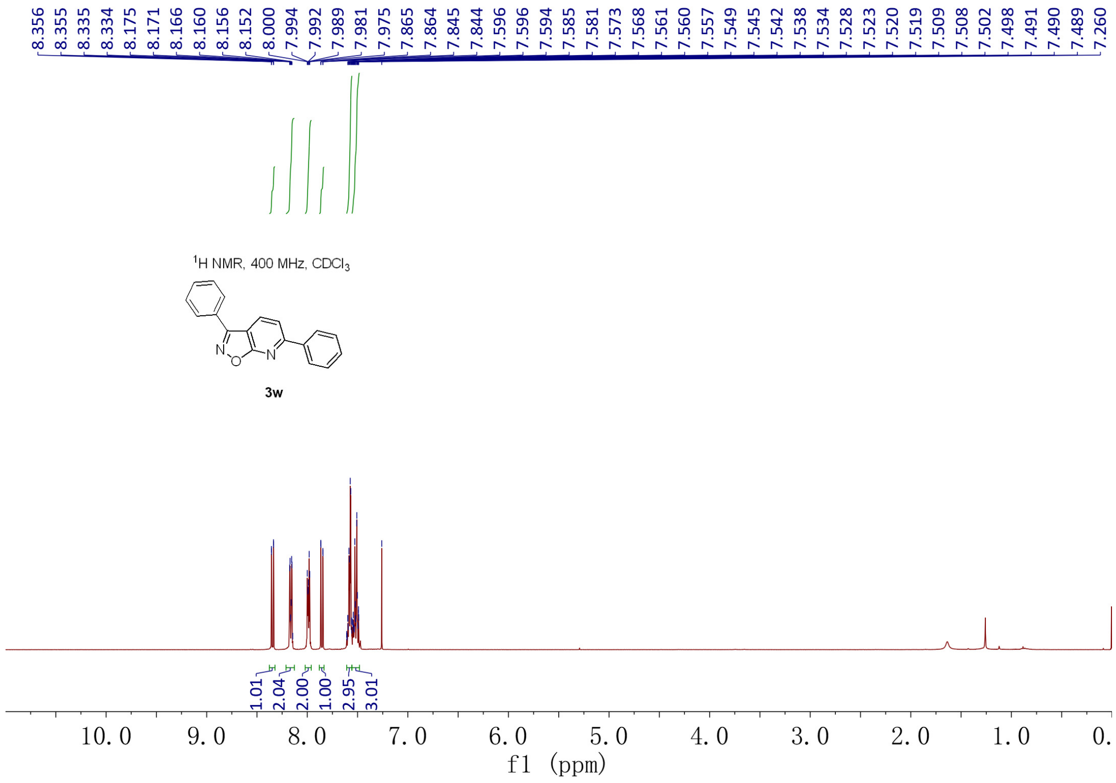
8.356
8.355
8.335
8.334
8.175
8.171
8.166
8.160
8.156
8.152
8.000
7.994
7.992
7.989
7.981
7.975
7.865
7.864
7.845
7.844
7.596
7.596
7.594
7.585
7.581
7.573
7.568
7.561
7.560
7.557
7.549
7.545
7.542
7.538
7.534
7.528
7.523
7.520
7.519
7.509
7.508
7.502
7.498
7.491
7.490
7.489
7.260
1H NMR, 400 MHz, CDCl3
N
O
N
3w
1.01
2.04
2.00
1.00
2.95
3.01
10.0
9.0
8.0
7.0
6.0
5.0
4.0
3.0
2.0
1.0
0.
f1 (ppm)

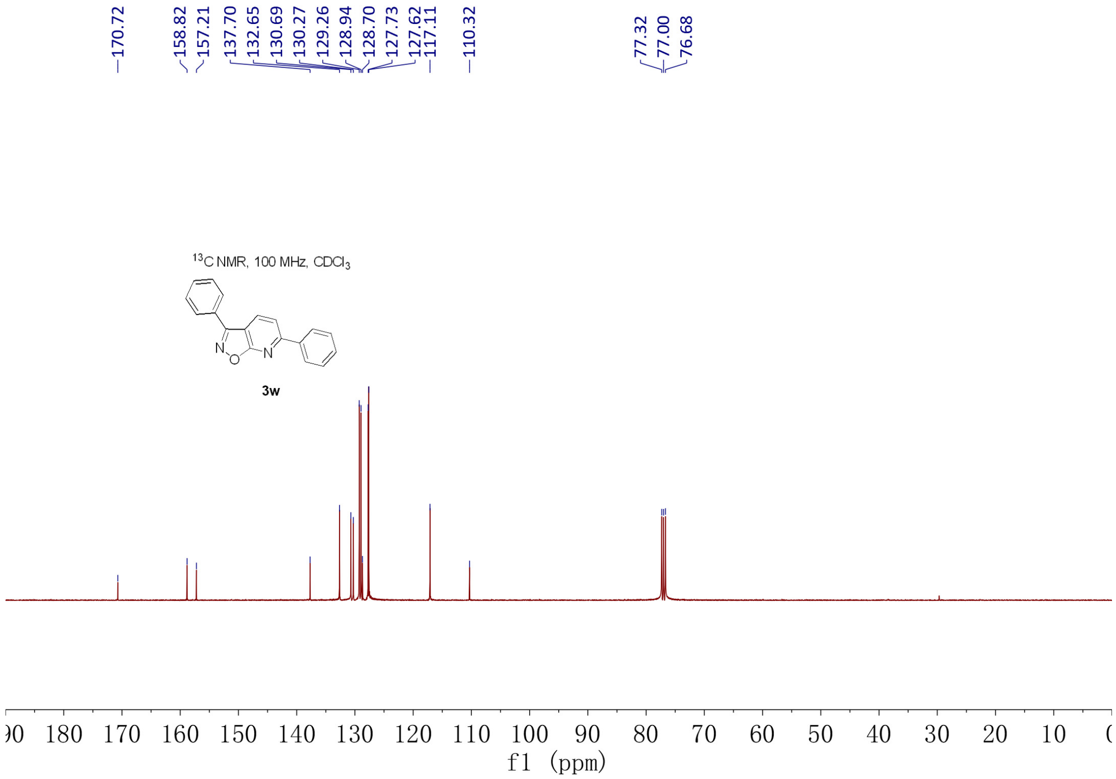
170.72
158.82
157.21
137.70
132.65
130.69
130.27
129.26
128.94
128.70
127.73
127.62
117.11
110.32
77.32
77.00
76.68
13C NMR, 100 MHz, CDCl3
N
O
N
3w
180
170
160
150
140
130
120
110
100
90
80
70
60
50
40
30
20
10
f1 (ppm)

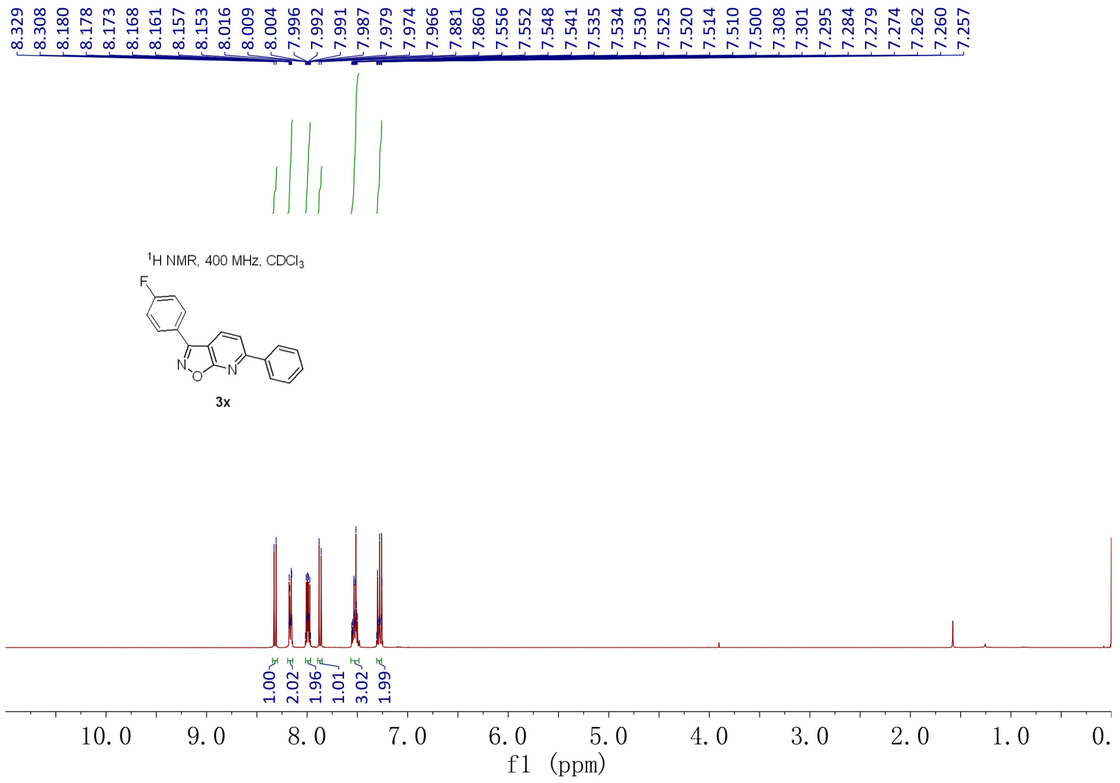
8.329 8.308 8.180 8.178 8.173 8.168 8.161 8.157 8.153 8.016 8.009 8.004 7.996 7.992 7.991 7.987 7.979 7.974 7.966 7.881 7.860 7.556 7.552 7.548 7.541 7.535 7.534 7.530 7.525 7.520 7.514 7.510 7.500 7.308 7.301 7.295 7.284 7.279 7.274 7.262 7.260 7.257
1H NMR, 400 MHz, CDCl3
F
N
O
N
3x
1.00
2.02
1.96
1.01
3.02
1.99
10.0 9.0 8.0 7.0 6.0 5.0 4.0 3.0 2.0 1.0 0.
f1 (ppm)

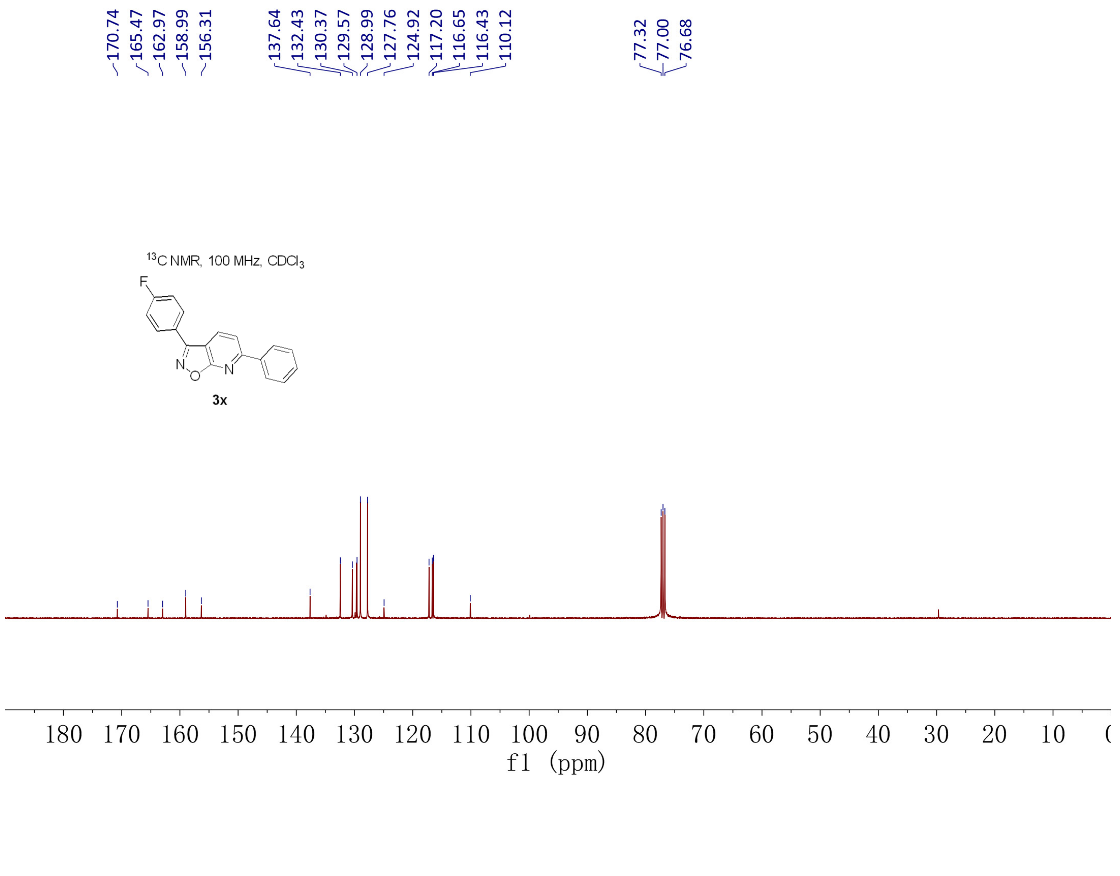
170.74 165.47 162.97 158.99 156.31
137.64 132.43 130.37 129.57 128.99 127.76 124.92 117.20 116.65 116.43 110.12
77.32 77.00 76.68
13C NMR, 100 MHz, CDCl3
F
N
O
N
3x
180 170 160 150 140 130 120 110 100 90 80 70 60 50 40 30 20 10
f1 (ppm)

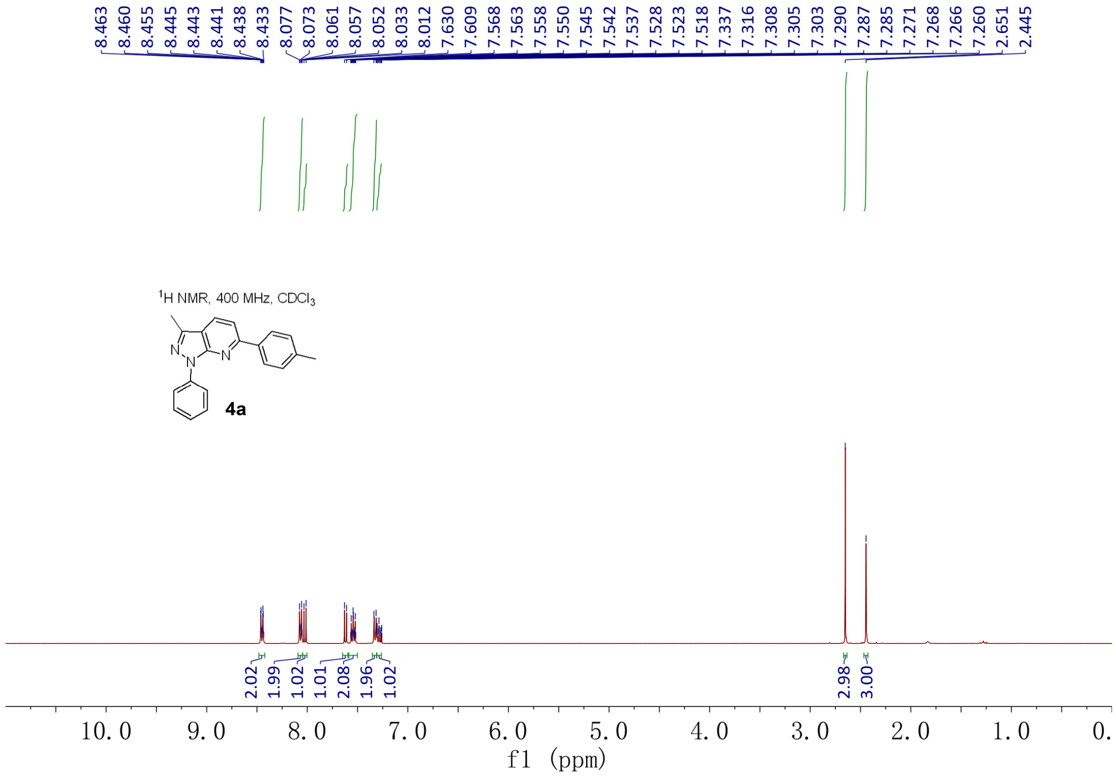
8.463 8.460 8.455 8.445 8.443 8.441 8.438 8.433 8.077 8.073 8.061 8.057 8.052 8.033 8.012 7.630 7.609 7.568 7.563 7.558 7.550 7.545 7.542 7.537 7.528 7.523 7.518 7.337 7.316 7.308 7.305 7.303 7.290 7.287 7.285 7.271 7.268 7.266 7.260 2.651 2.445
1H NMR, 400 MHz, CDCl3
N
N
N
4a
2.02 1.99 1.02 1.01 2.08 1.96 1.02
2.98 3.00
10.0 9.0 8.0 7.0 6.0 5.0 4.0 3.0 2.0 1.0
f1 (ppm)

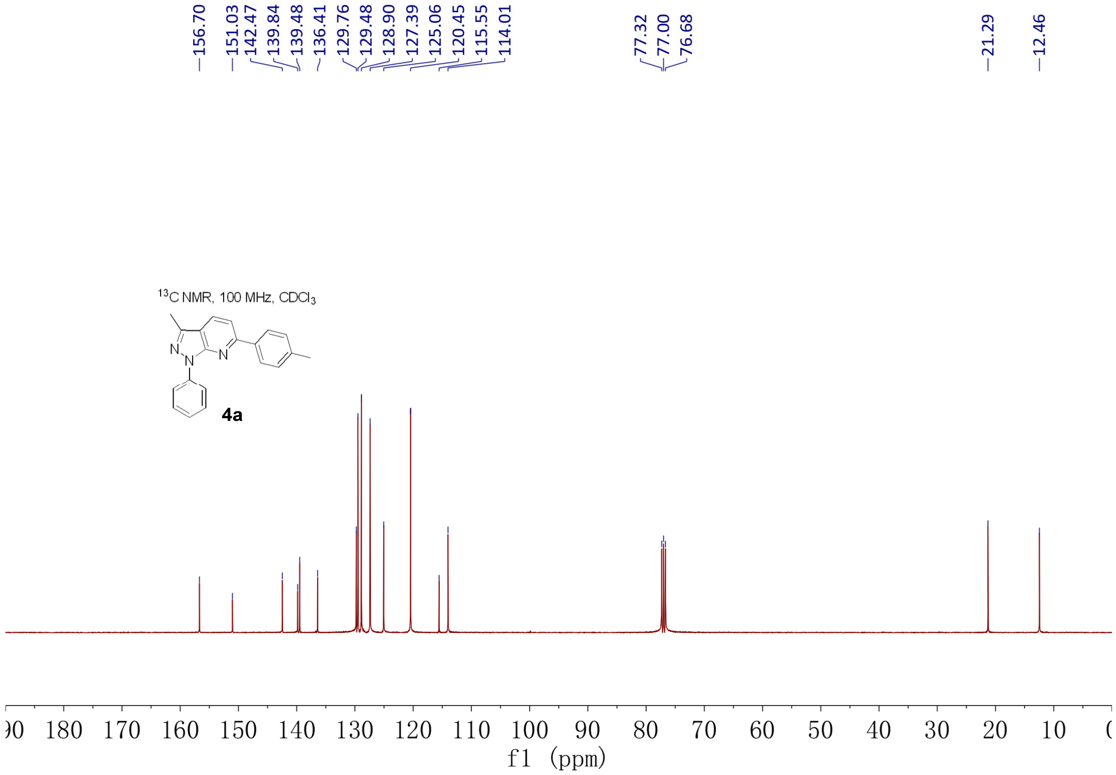
156.70 151.03 142.47 139.84 139.48 136.41 129.76 129.48 128.90 127.39 125.06 120.45 115.55 114.01
77.32 77.00 76.68
21.29 12.46
13C NMR, 100 MHz, CDCl3
N
N
N
4a
180 170 160 150 140 130 120 110 100 90 80 70 60 50 40 30 20 10
f1 (ppm)

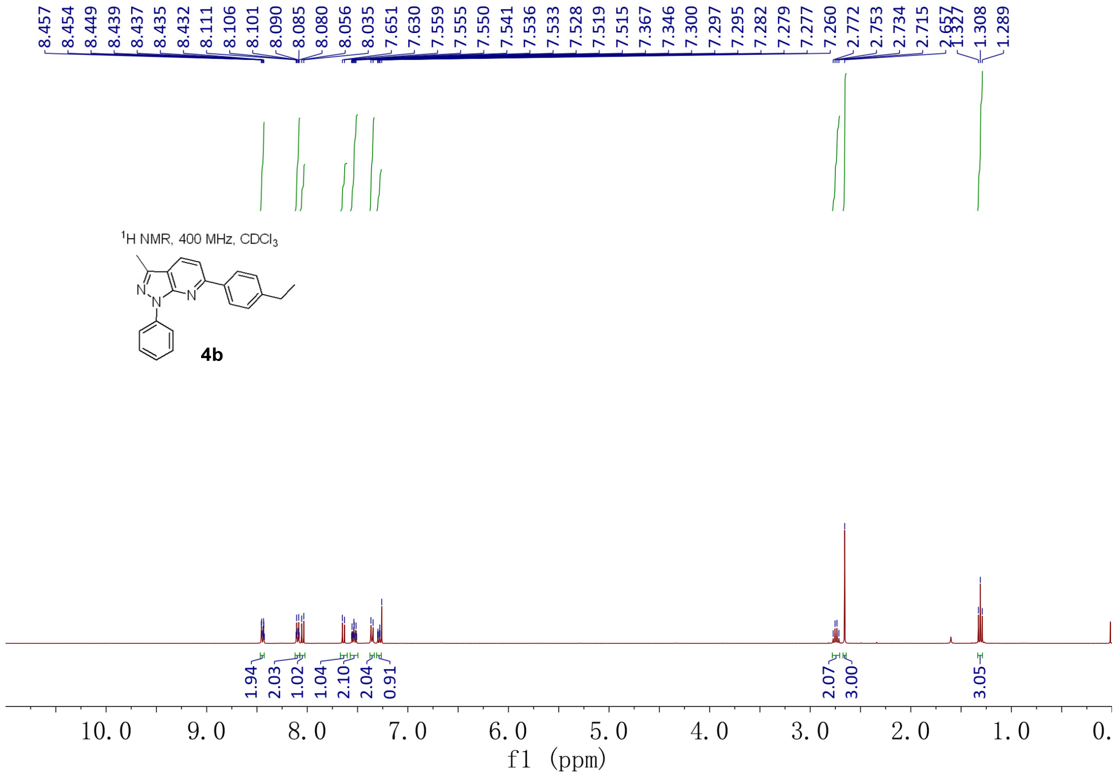
8.457 8.454 8.449 8.439 8.437 8.435 8.432 8.111 8.106 8.101 8.090 8.085 8.080 8.056 8.035 7.651 7.630 7.559 7.555 7.550 7.541 7.536 7.533 7.528 7.519 7.515 7.367 7.346 7.300 7.297 7.295 7.282 7.279 7.277 7.260 2.772 2.753 2.734 2.715 2.657 1.327 1.308 1.289
^{1}H NMR, 400 MHz, $CDCl_3$
4b
1.94 2.03 1.02 1.04 2.10 2.04 0.91 2.07 3.00 3.05
f1 (ppm)

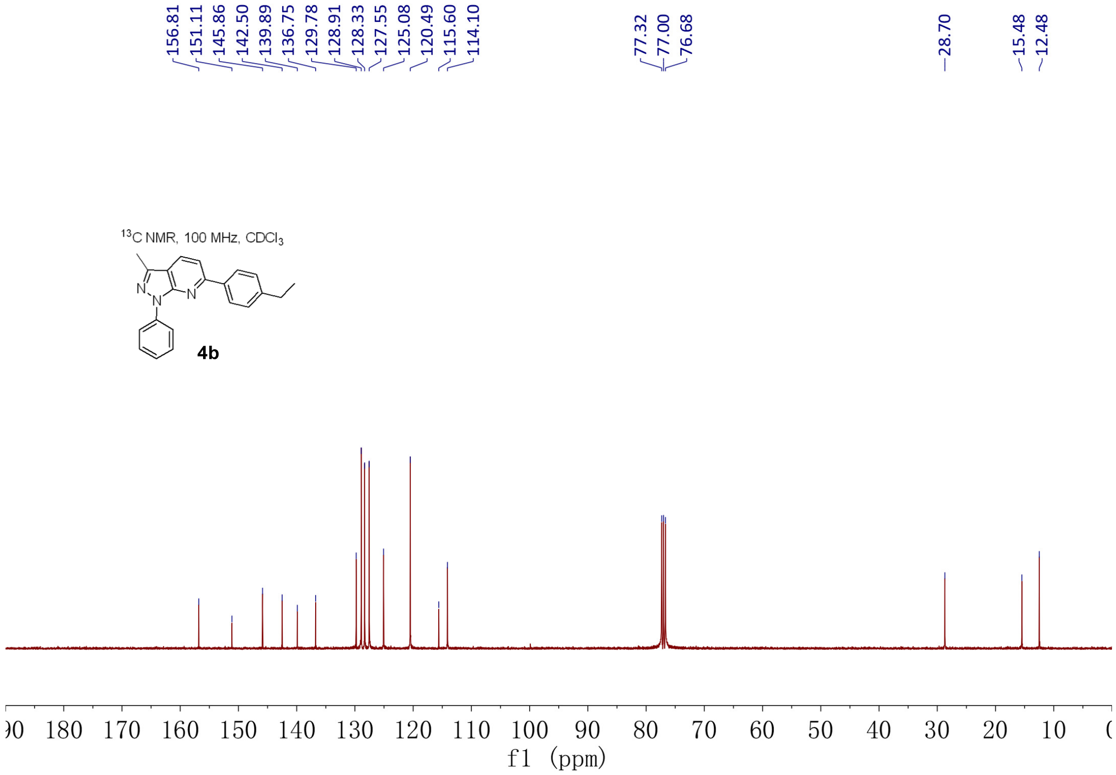
156.81 151.11 145.86 142.50 139.89 136.75 129.78 128.91 128.33 127.55 125.08 120.49 115.60 114.10 77.32 77.00 76.68 28.70 15.48 12.48
^{13}C NMR, 100 MHz, $CDCl_3$
4b
f1 (ppm)

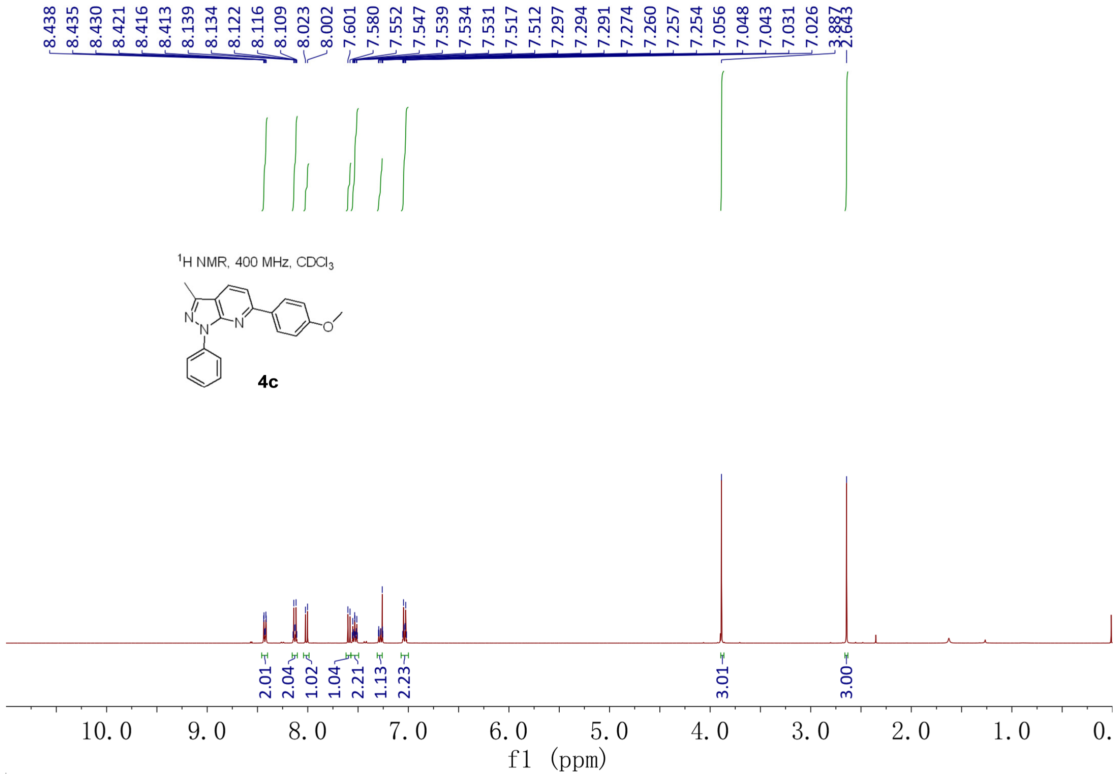
8.438 8.435 8.430 8.421 8.416 8.413 8.139 8.134 8.122 8.116 8.109 8.023 8.002 7.601 7.580 7.552 7.547 7.539 7.534 7.531 7.517 7.512 7.297 7.294 7.291 7.274 7.260 7.257 7.254 7.056 7.048 7.043 7.031 7.026 3.887 2.643
^{1}H NMR, 400 MHz, CDCl$_3$
N
N
N
O
4c
2.01 2.04 1.02 1.04 2.21 1.13 2.23 3.01 3.00
10.0 9.0 8.0 7.0 6.0 5.0 4.0 3.0 2.0 1.0 0.
f1 (ppm)

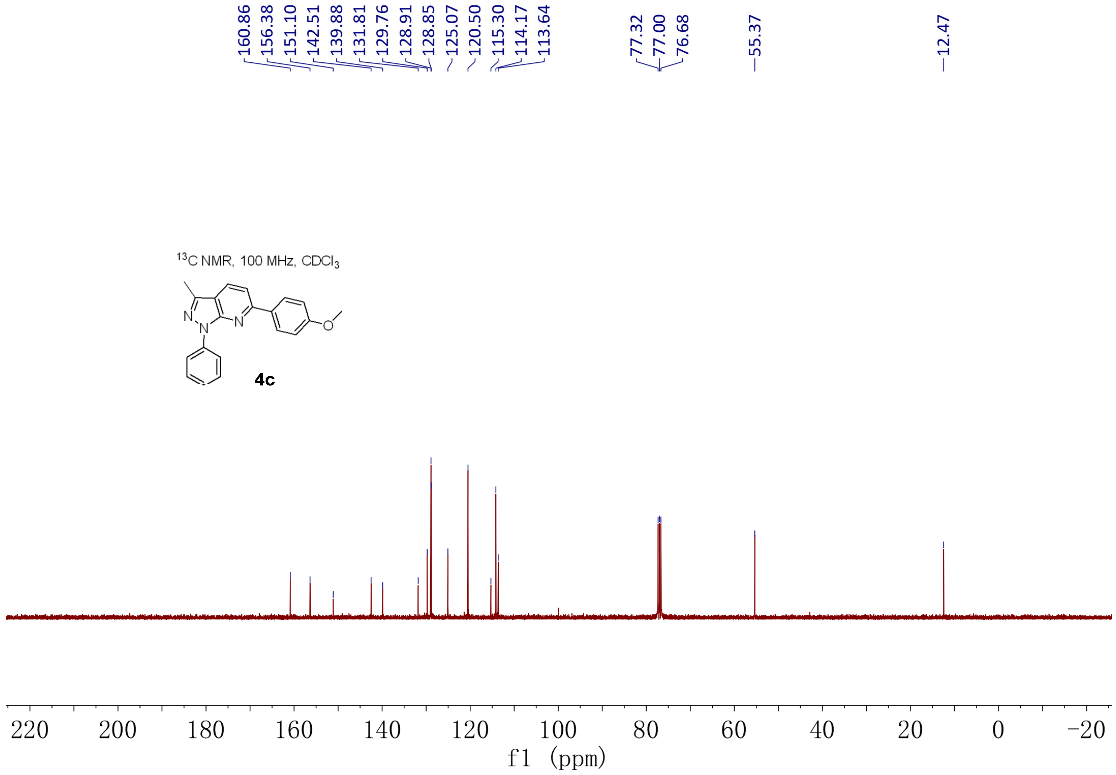
160.86 156.38 151.10 142.51 139.88 131.81 129.76 128.91 128.85 125.07 120.50 115.30 114.17 113.64
77.32 77.00 76.68
55.37
12.47
^{13}C NMR, 100 MHz, CDCl$_3$
N
N
N
O
4c
220 200 180 160 140 120 100 80 60 40 20 0 −20
f1 (ppm)

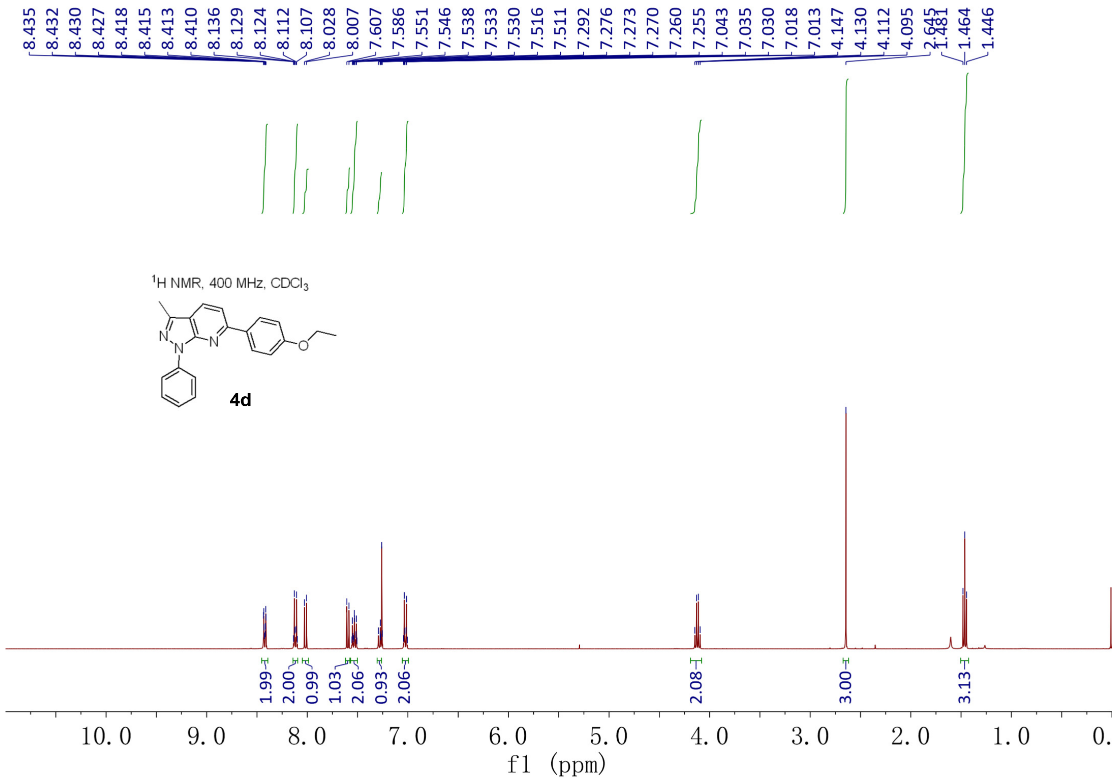
8.435
8.432
8.430
8.427
8.418
8.415
8.413
8.410
8.136
8.129
8.124
8.112
8.107
8.028
8.007
7.607
7.586
7.551
7.546
7.538
7.533
7.530
7.516
7.511
7.292
7.276
7.273
7.270
7.260
7.255
7.043
7.035
7.030
7.018
7.013
4.147
4.130
4.112
4.095
2.645
1.481
1.464
1.446
^{1}H NMR, 400 MHz, CDCl$_3$
4d
1.99
2.00
0.99
1.03
2.06
0.93
2.06
2.08
3.00
3.13
10.0 9.0 8.0 7.0 6.0 5.0 4.0 3.0 2.0 1.0 0.
f1 (ppm)

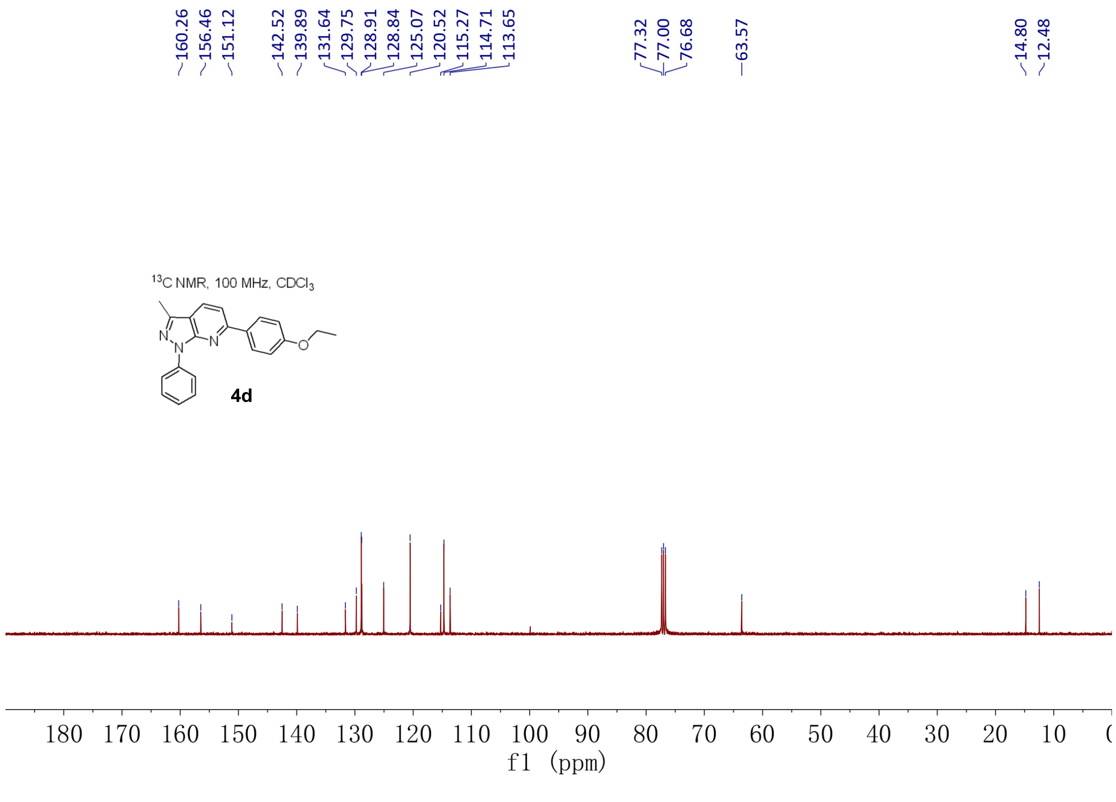
160.26
156.46
151.12
142.52
139.89
131.64
129.75
128.91
128.84
125.07
120.52
115.27
114.71
113.65
77.32
77.00
76.68
63.57
14.80
12.48
^{13}C NMR, 100 MHz, CDCl$_3$
4d
180 170 160 150 140 130 120 110 100 90 80 70 60 50 40 30 20 10
f1 (ppm)

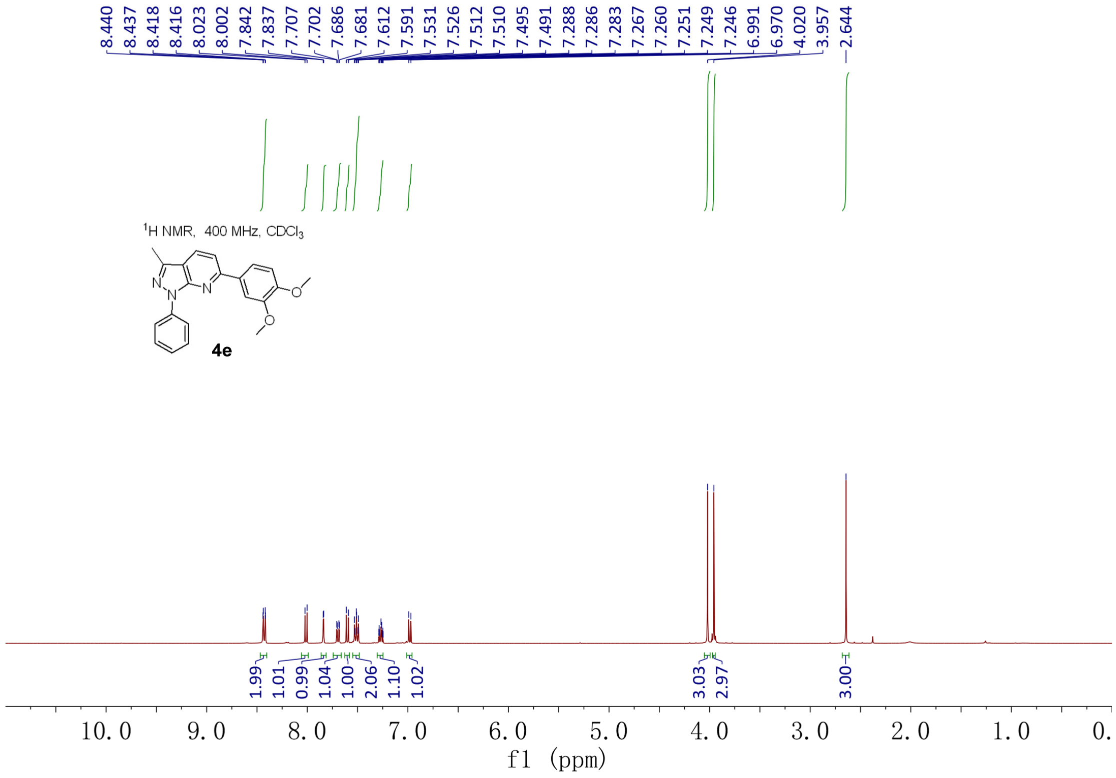
8.440
8.437
8.418
8.416
8.023
8.002
7.842
7.837
7.707
7.702
7.686
7.681
7.612
7.591
7.531
7.526
7.512
7.510
7.495
7.491
7.288
7.286
7.283
7.267
7.260
7.251
7.249
7.246
6.991
6.970
4.020
3.957
2.644
1H NMR, 400 MHz, CDCl3
4e
1.99
1.01
0.99
1.04
1.00
2.06
1.10
1.02
3.03
2.97
3.00
10.0
9.0
8.0
7.0
6.0
5.0
4.0
3.0
2.0
1.0
0.
f1 (ppm)

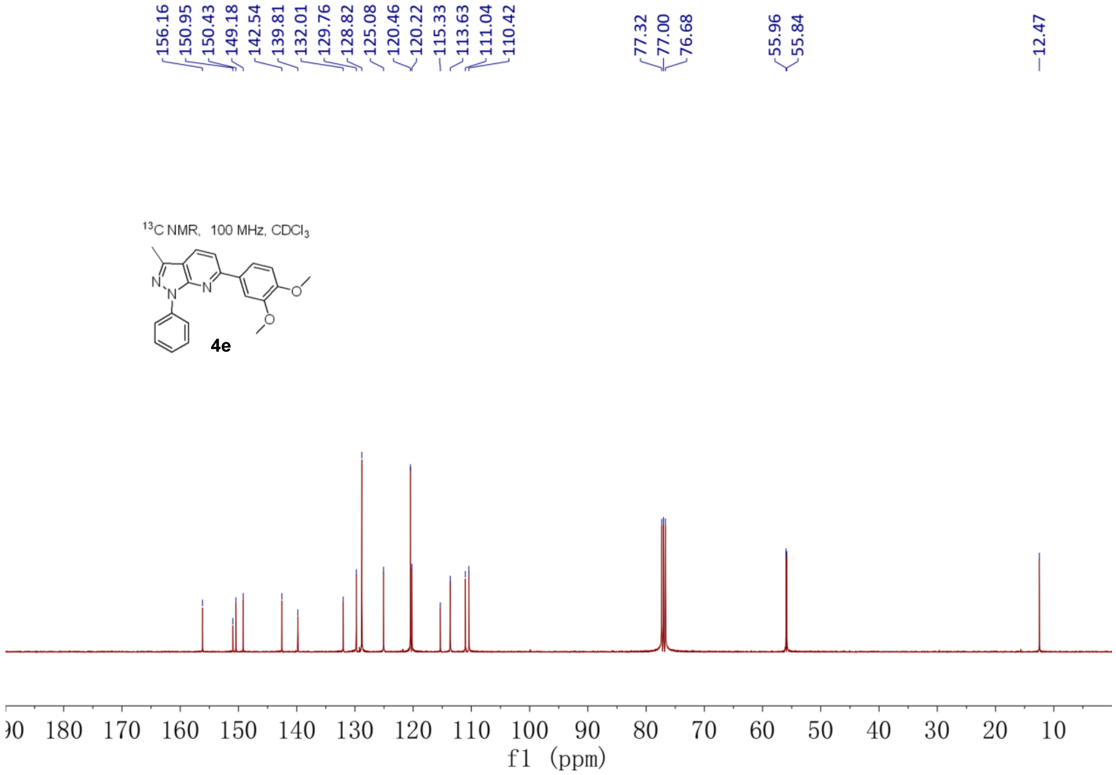
156.16
150.95
150.43
149.18
142.54
139.81
132.01
129.76
128.82
125.08
120.46
120.22
115.33
113.63
111.04
110.42
77.32
77.00
76.68
55.96
55.84
12.47
13C NMR, 100 MHz, CDCl3
4e
180
170
160
150
140
130
120
110
100
90
80
70
60
50
40
30
20
10
f1 (ppm)

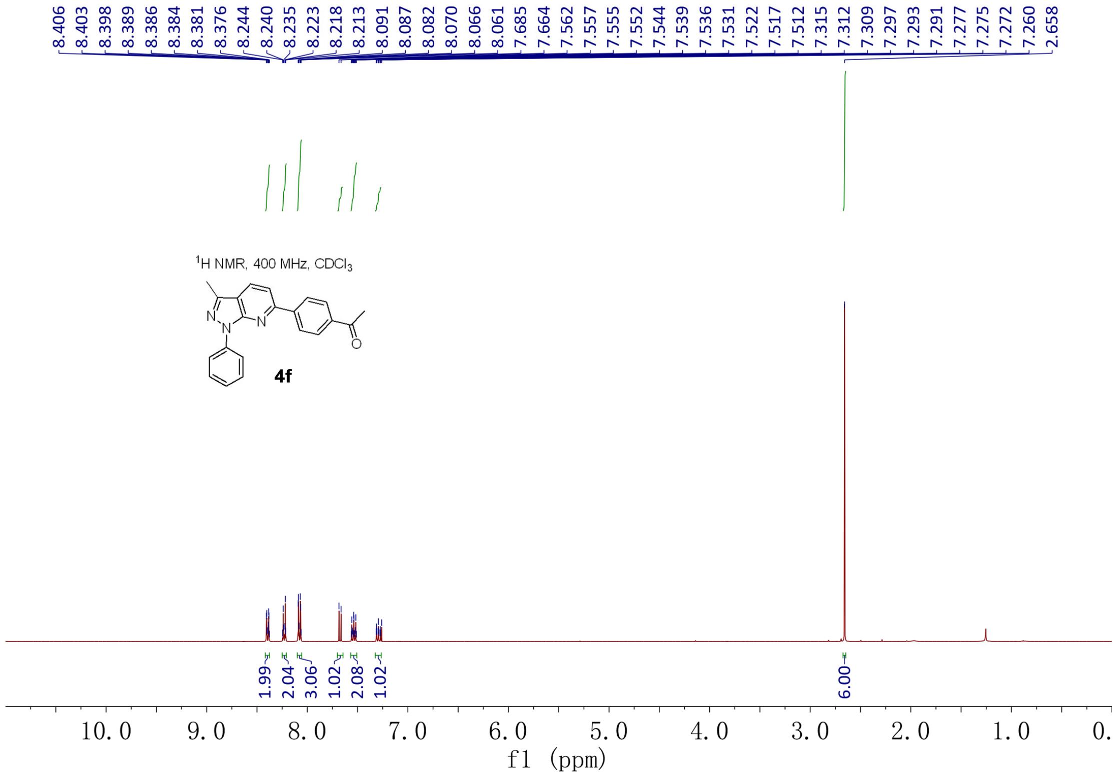
8.406 8.403 8.398 8.389 8.386 8.384 8.381 8.376 8.244 8.240 8.235 8.223 8.218 8.213 8.091 8.087 8.082 8.070 8.066 8.061 7.685 7.664 7.562 7.557 7.555 7.552 7.544 7.539 7.536 7.531 7.522 7.517 7.512 7.315 7.312 7.309 7.297 7.293 7.291 7.277 7.275 7.272 7.260 2.658
1H NMR, 400 MHz, CDCl3
N
N
N
O
4f
1.99 2.04 3.06 1.02 2.08 1.02 6.00
10.0 9.0 8.0 7.0 6.0 5.0 4.0 3.0 2.0 1.0 0.
f1 (ppm)

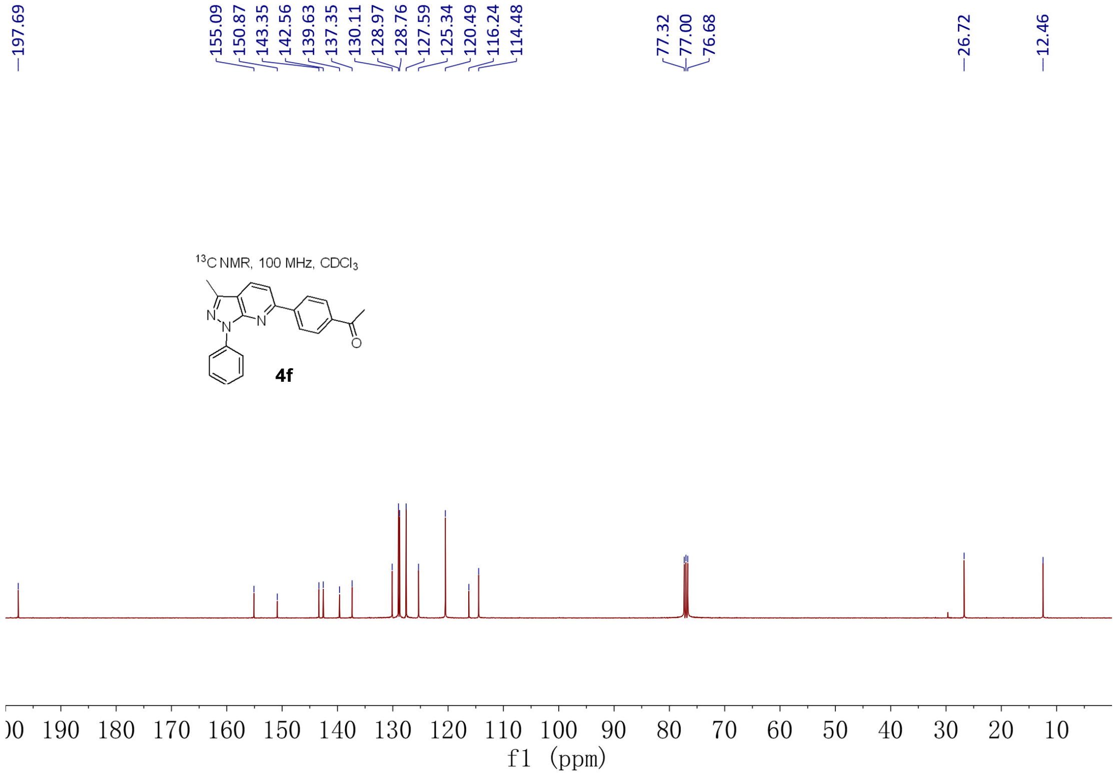
197.69
155.09 150.87 143.35 142.56 139.63 137.35 130.11 128.97 128.76 127.59 125.34 120.49 116.24 114.48
77.32 77.00 76.68
26.72
12.46
13C NMR, 100 MHz, CDCl3
N
N
N
O
4f
190 180 170 160 150 140 130 120 110 100 90 80 70 60 50 40 30 20 10
f1 (ppm)

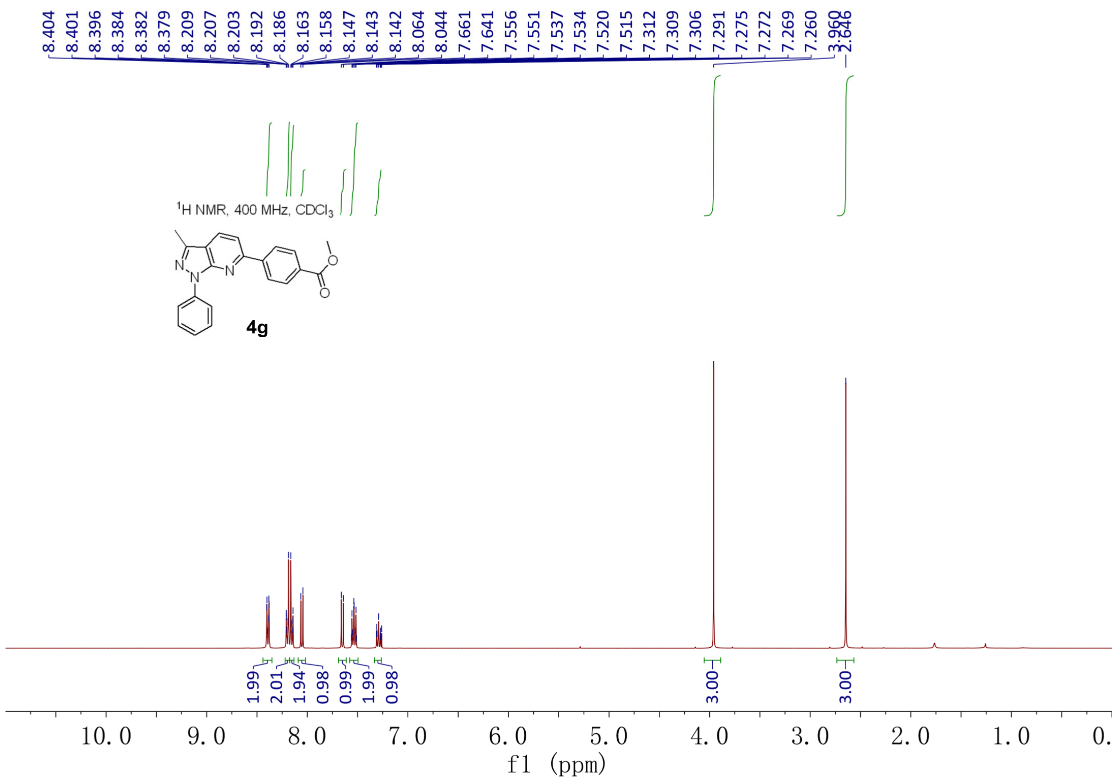
8.404
8.401
8.396
8.384
8.382
8.379
8.209
8.207
8.203
8.192
8.186
8.163
8.158
8.147
8.143
8.142
8.064
8.044
7.661
7.641
7.556
7.551
7.537
7.534
7.520
7.515
7.312
7.309
7.306
7.291
7.275
7.272
7.269
7.260
3.960
2.646
1H NMR, 400 MHz, CDCl3
N
N
N
O
O
4g
1.99
2.01
1.94
0.98
0.99
1.99
0.98
3.00
3.00
10.0
9.0
8.0
7.0
6.0
5.0
4.0
3.0
2.0
1.0
0.
f1 (ppm)

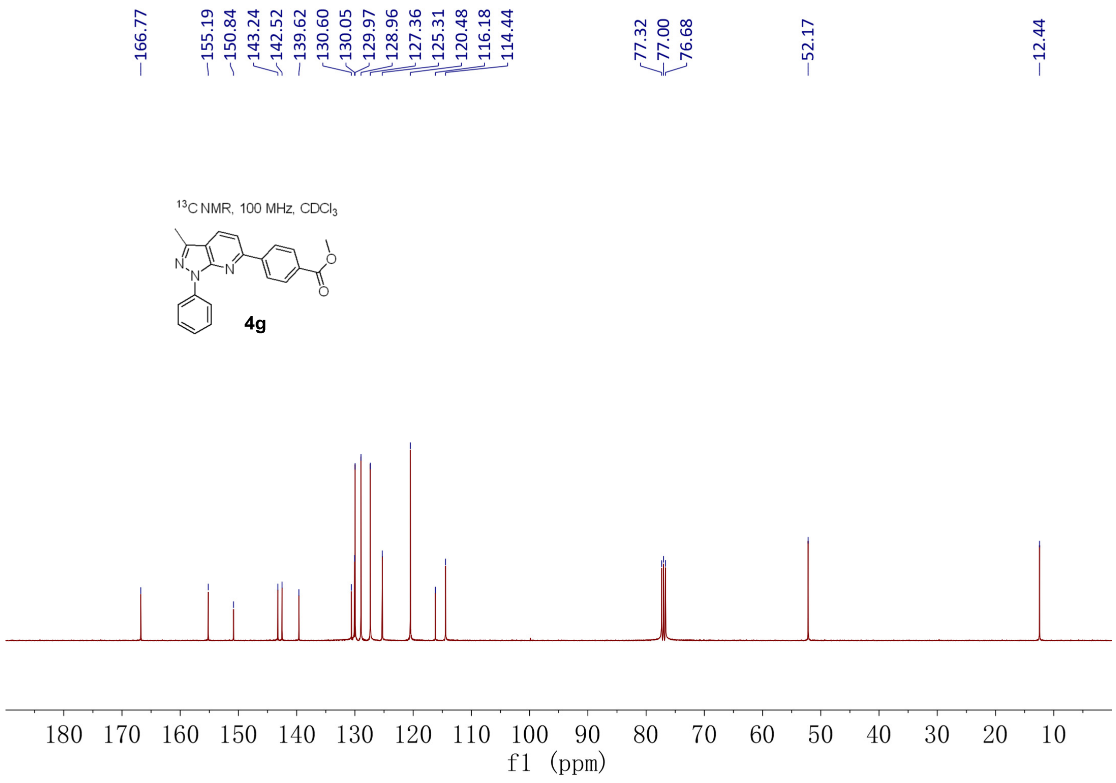
166.77
155.19
150.84
143.24
142.52
139.62
130.60
130.05
129.97
128.96
127.36
125.31
120.48
116.18
114.44
77.32
77.00
76.68
52.17
12.44
13C NMR, 100 MHz, CDCl3
N
N
N
O
O
4g
180
170
160
150
140
130
120
110
100
90
80
70
60
50
40
30
20
10
f1 (ppm)

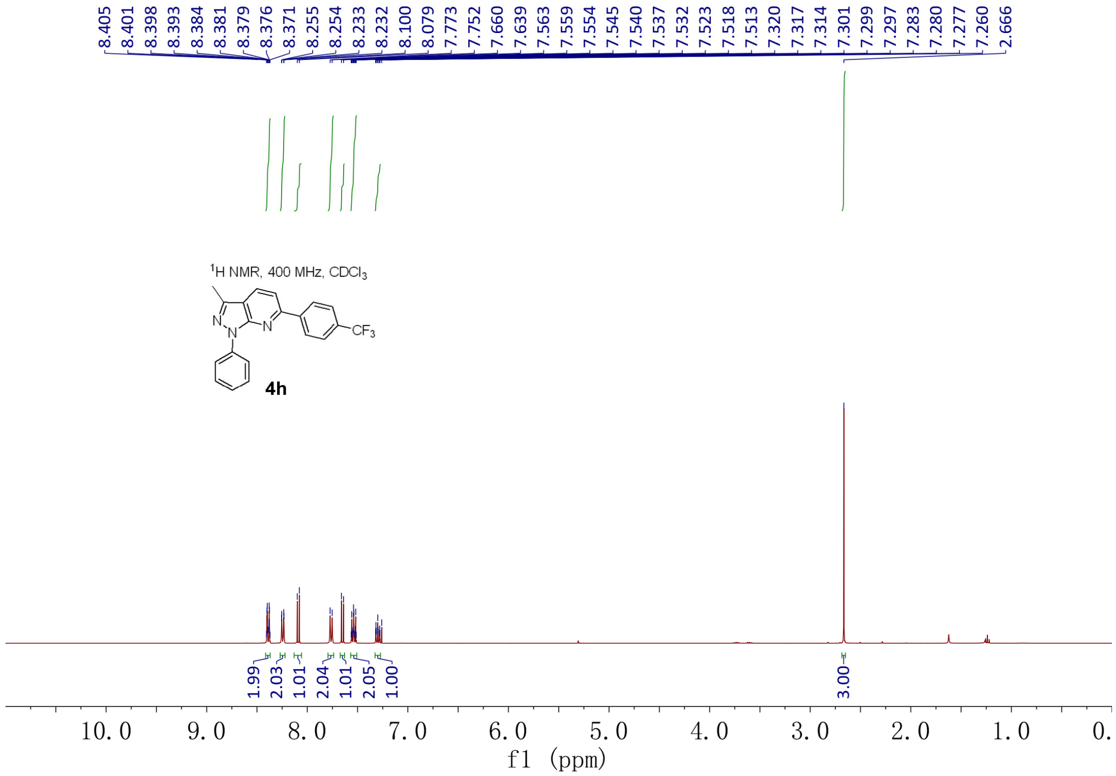
8.405 8.401 8.398 8.393 8.384 8.381 8.379 8.376 8.371 8.255 8.254 8.233 8.232 8.100 8.079 7.773 7.752 7.660 7.639 7.563 7.559 7.554 7.545 7.540 7.537 7.532 7.523 7.518 7.513 7.320 7.317 7.314 7.301 7.299 7.297 7.283 7.280 7.277 7.260 2.666
1H NMR, 400 MHz, CDCl3
N
N
N
CF3
4h
1.99 2.03 1.01 2.04 1.01 2.05 1.00
3.00
10.0 9.0 8.0 7.0 6.0 5.0 4.0 3.0 2.0 1.0 0.
f1 (ppm)

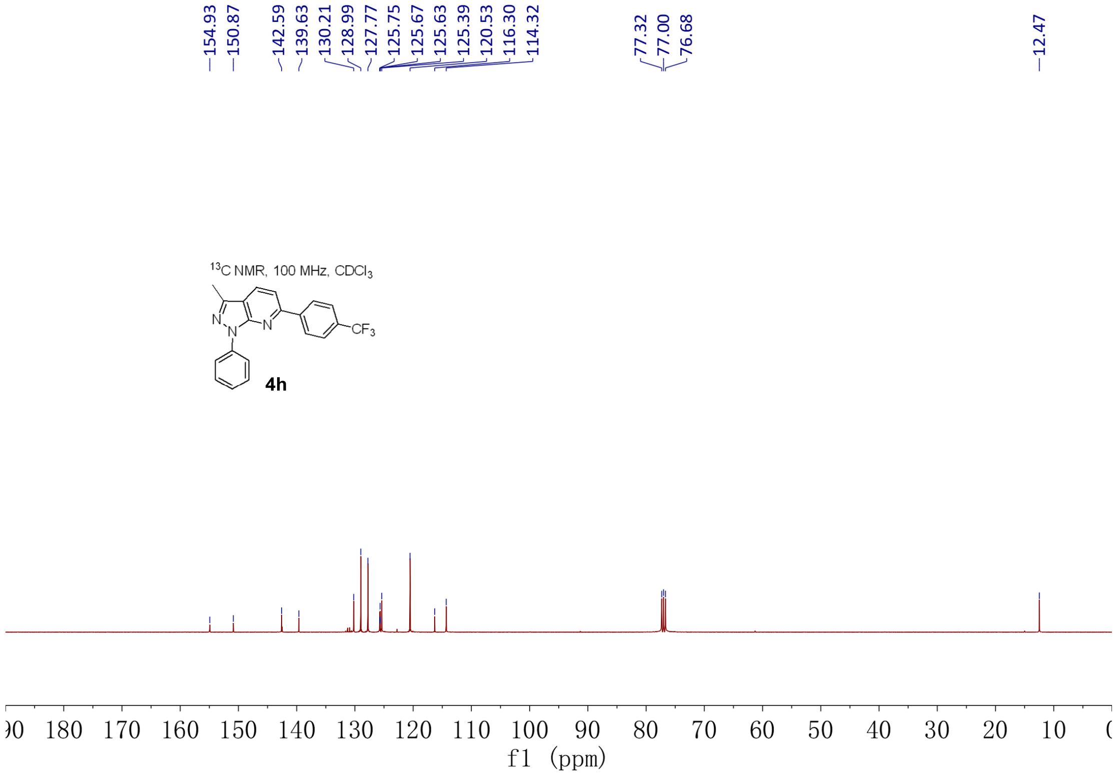
154.93 150.87 142.59 139.63 130.21 128.99 127.77 125.75 125.67 125.63 125.39 120.53 116.30 114.32
77.32 77.00 76.68
12.47
13C NMR, 100 MHz, CDCl3
N
N
N
CF3
4h
180 170 160 150 140 130 120 110 100 90 80 70 60 50 40 30 20 10
f1 (ppm)

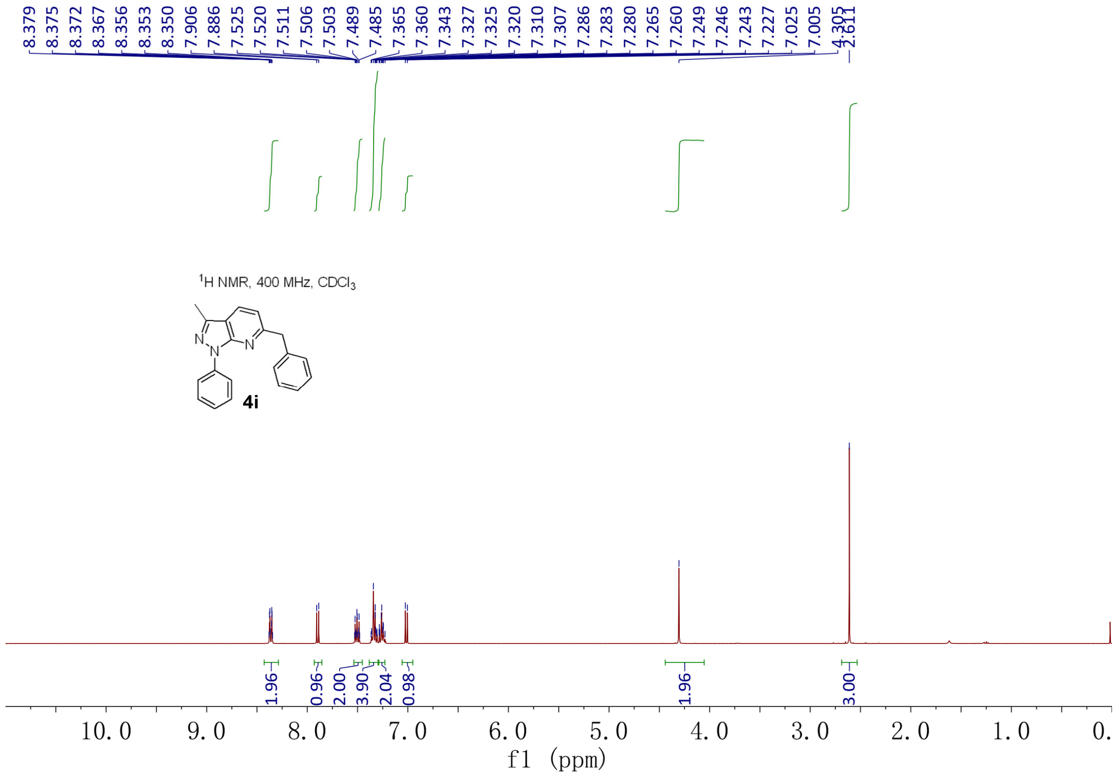
8.379
8.375
8.372
8.367
8.356
8.353
8.350
7.906
7.886
7.525
7.520
7.511
7.506
7.503
7.489
7.485
7.365
7.360
7.343
7.327
7.325
7.320
7.310
7.307
7.286
7.283
7.280
7.265
7.260
7.249
7.246
7.243
7.227
7.025
7.005
4.305
2.611
1H NMR, 400 MHz, CDCl3
N
N
N
4i
1.96
0.96
2.00
3.90
2.04
0.98
1.96
3.00
10.0 9.0 8.0 7.0 6.0 5.0 4.0 3.0 2.0 1.0 0.
f1 (ppm)

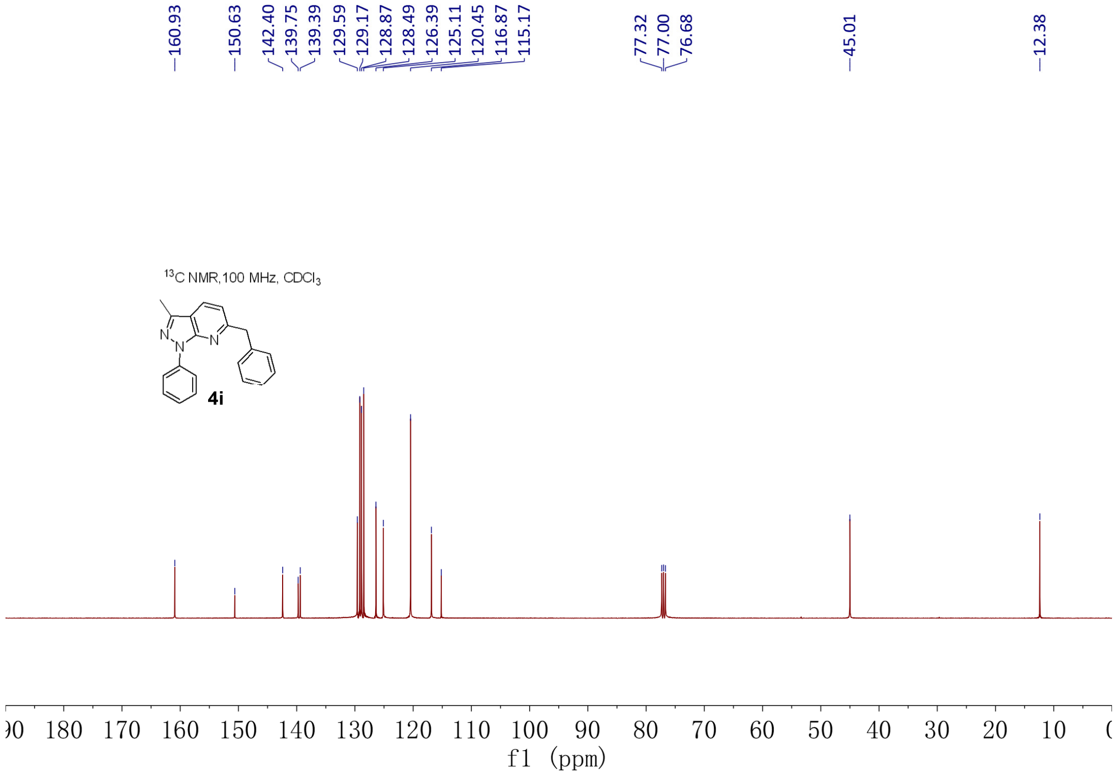
160.93
150.63
142.40
139.75
139.39
129.59
129.17
128.87
128.49
126.39
125.11
120.45
116.87
115.17
77.32
77.00
76.68
45.01
12.38
13C NMR,100 MHz, CDCl3
N
N
N
4i
90 180 170 160 150 140 130 120 110 100 90 80 70 60 50 40 30 20 10 0
f1 (ppm)

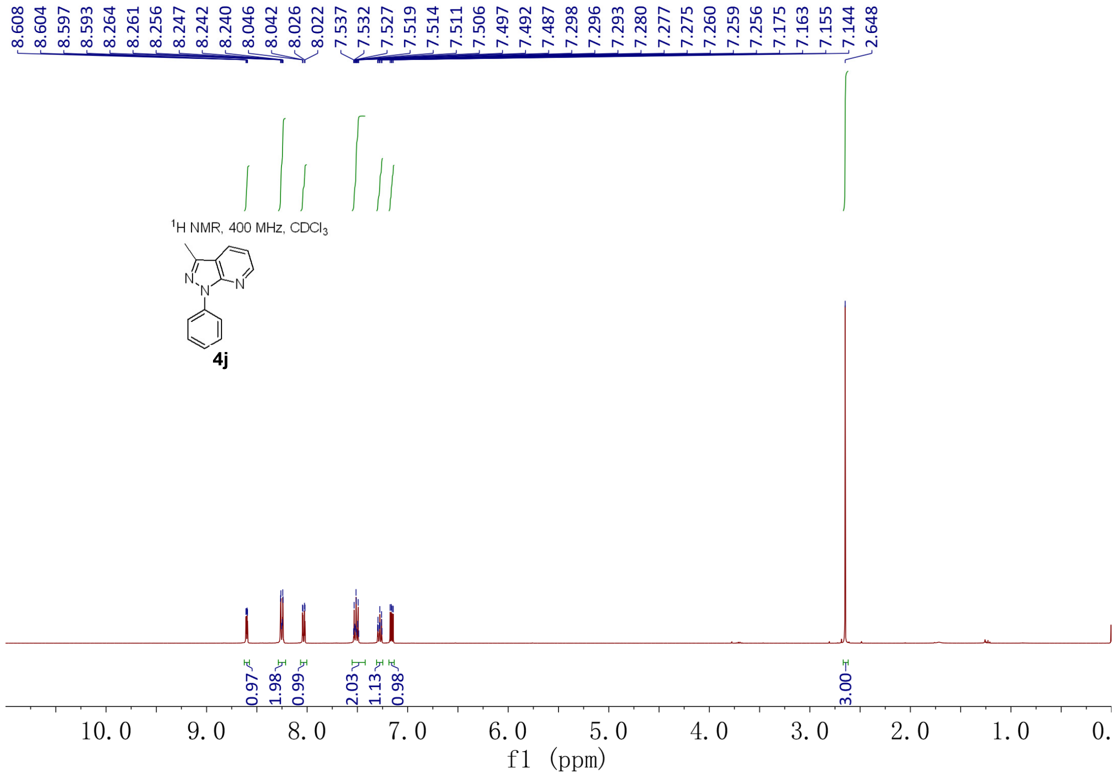
8.608
8.604
8.597
8.593
8.264
8.261
8.256
8.247
8.242
8.240
8.046
8.042
8.026
8.022
7.537
7.532
7.527
7.519
7.514
7.511
7.506
7.497
7.492
7.487
7.298
7.296
7.293
7.280
7.277
7.275
7.260
7.259
7.256
7.175
7.163
7.155
7.144
2.648
1H NMR, 400 MHz, CDCl3
4j
0.97
1.98
0.99
2.03
1.13
0.98
3.00
10.0 9.0 8.0 7.0 6.0 5.0 4.0 3.0 2.0 1.0 0.
f1 (ppm)

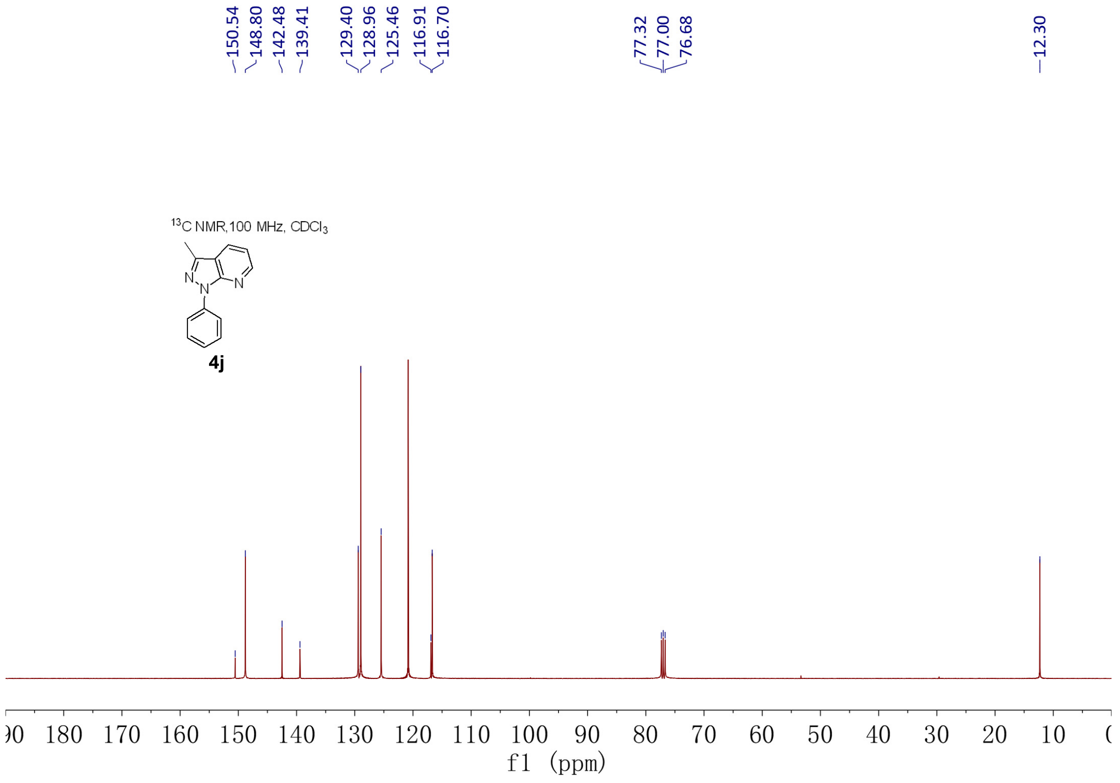
150.54
148.80
142.48
139.41
129.40
128.96
125.46
116.91
116.70
77.32
77.00
76.68
12.30
13C NMR,100 MHz, CDCl3
4j
180 170 160 150 140 130 120 110 100 90 80 70 60 50 40 30 20 10
f1 (ppm)

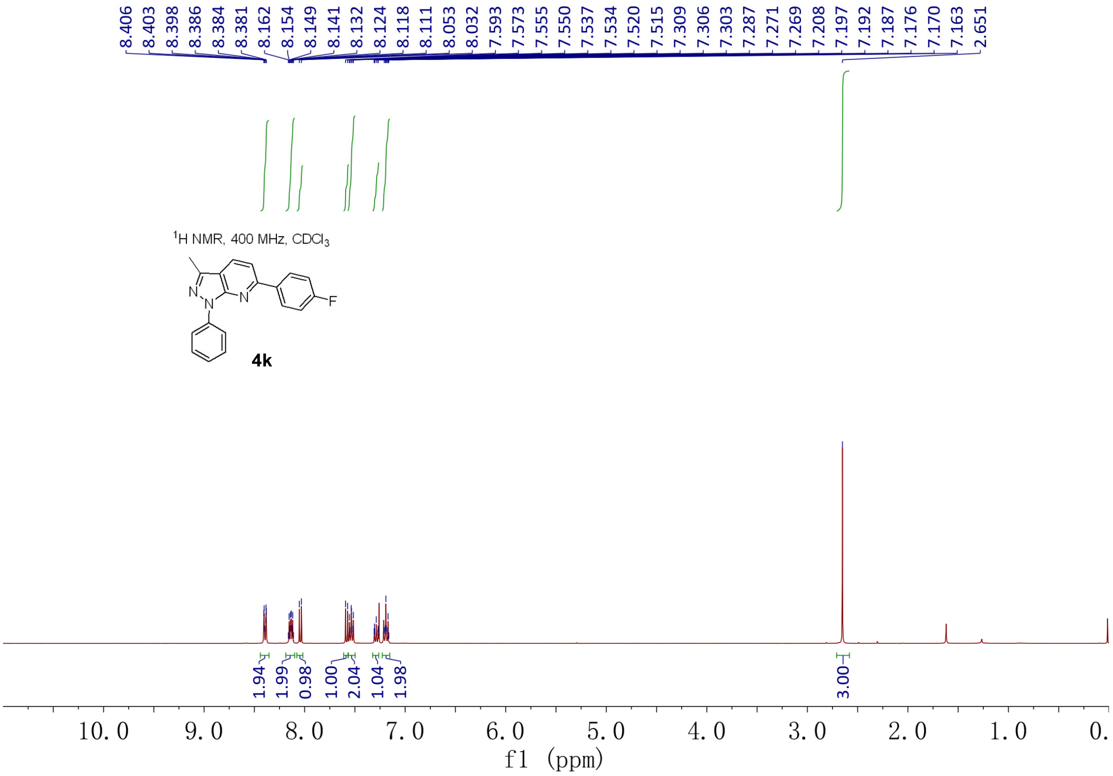
8.406 8.403 8.398 8.386 8.384 8.381 8.162 8.154 8.149 8.141 8.132 8.124 8.118 8.111 8.053 8.032 7.593 7.573 7.555 7.550 7.537 7.534 7.520 7.515 7.309 7.306 7.303 7.287 7.271 7.269 7.208 7.197 7.192 7.187 7.176 7.170 7.163 2.651
^{1}H NMR, 400 MHz, CDCl$_3$
N
N
N
F
4k
1.94 1.99 0.98 1.00 2.04 1.04 1.98 3.00
10.0 9.0 8.0 7.0 6.0 5.0 4.0 3.0 2.0 1.0 0.
f1 (ppm)

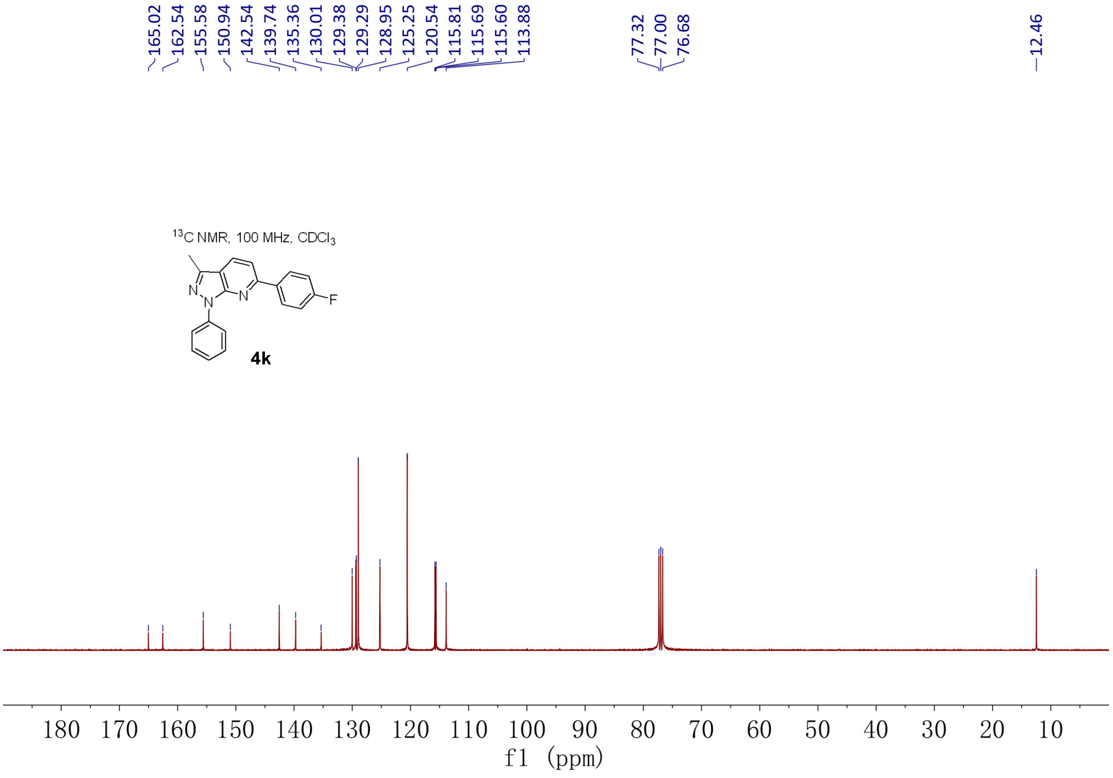
165.02 162.54 155.58 150.94 142.54 139.74 135.36 130.01 129.38 129.29 128.95 125.25 120.54 115.81 115.69 115.60 113.88
77.32 77.00 76.68
12.46
^{13}C NMR, 100 MHz, CDCl$_3$
N
N
N
F
4k
180 170 160 150 140 130 120 110 100 90 80 70 60 50 40 30 20 10
f1 (ppm)

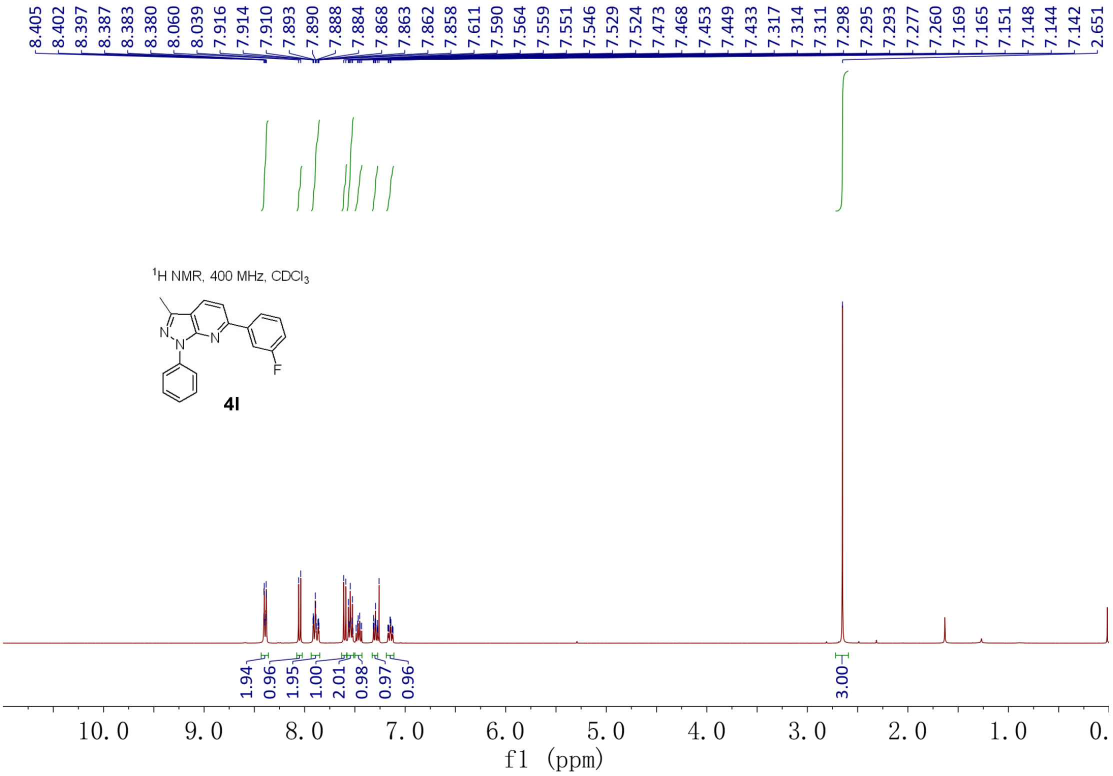
8.405 8.402 8.397 8.387 8.383 8.380 8.060 8.039 7.916 7.914 7.910 7.893 7.890 7.888 7.884 7.868 7.863 7.862 7.858 7.611 7.590 7.564 7.559 7.551 7.546 7.529 7.524 7.473 7.468 7.453 7.449 7.433 7.317 7.314 7.311 7.298 7.295 7.293 7.277 7.260 7.169 7.165 7.151 7.148 7.144 7.142 2.651
1H NMR, 400 MHz, CDCl3
N
N
N
F
4l
1.94 0.96 1.95 1.00 2.01 0.98 0.97 0.96
3.00
10.0 9.0 8.0 7.0 6.0 5.0 4.0 3.0 2.0 1.0 0.
f1 (ppm)

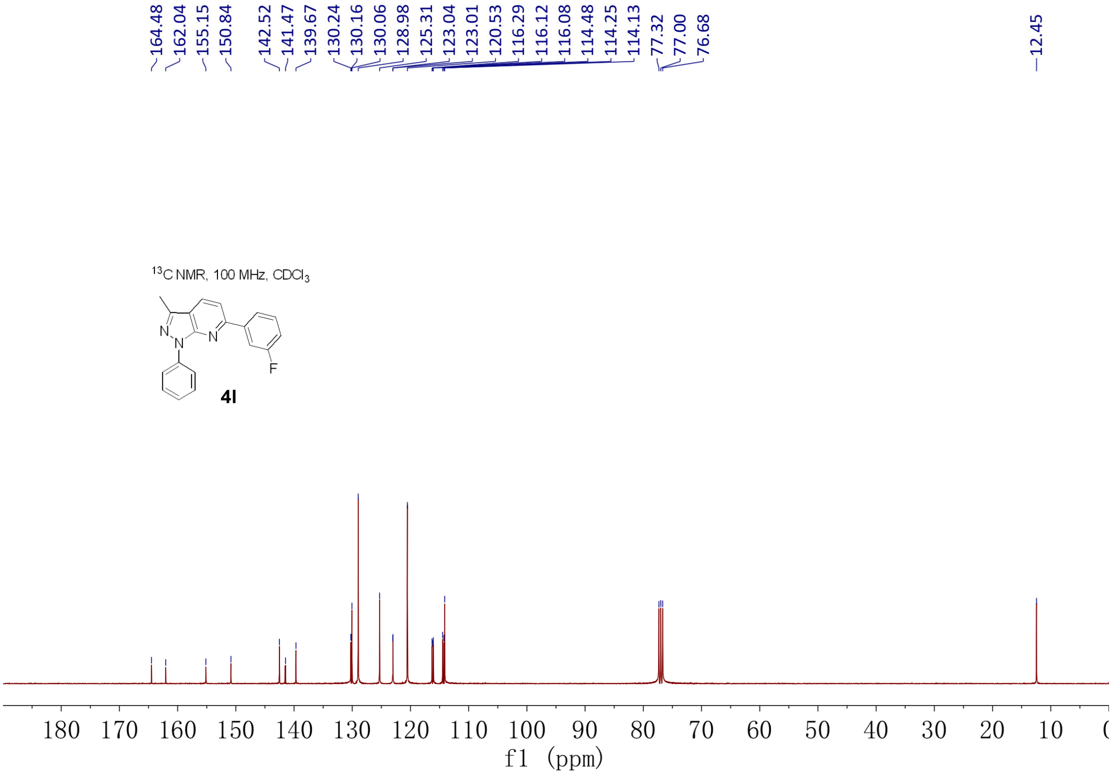
164.48 162.04 155.15 150.84 142.52 141.47 139.67 130.24 130.16 130.06 128.98 125.31 123.04 123.01 120.53 116.29 116.12 116.08 114.48 114.25 114.13 77.32 77.00 76.68
12.45
13C NMR, 100 MHz, CDCl3
N
N
N
F
4l
180 170 160 150 140 130 120 110 100 90 80 70 60 50 40 30 20 10
f1 (ppm)

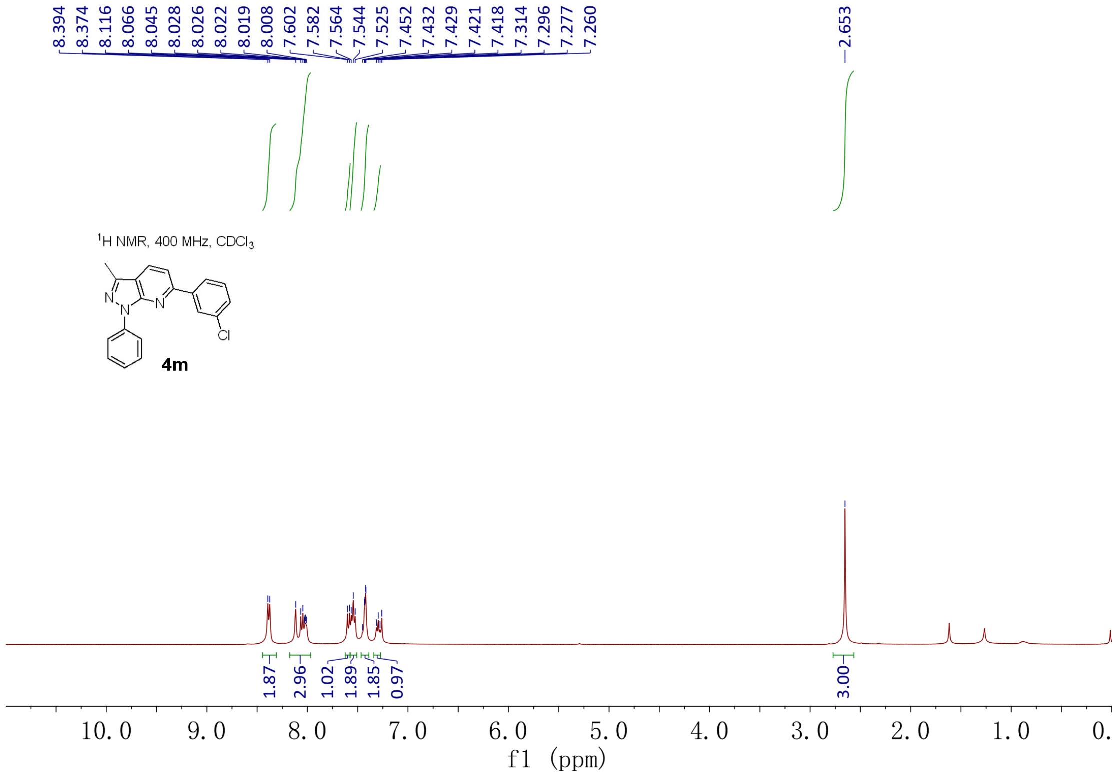
8.394
8.374
8.116
8.066
8.045
8.028
8.026
8.022
8.019
8.008
7.602
7.582
7.564
7.544
7.525
7.452
7.432
7.429
7.421
7.418
7.314
7.296
7.277
7.260
2.653
^{1}H NMR, 400 MHz, $CDCl_3$
N
N
N
Cl
4m
1.87
2.96
1.02
1.89
1.85
0.97
3.00
10.0
9.0
8.0
7.0
6.0
5.0
4.0
3.0
2.0
1.0
0.
f1 (ppm)

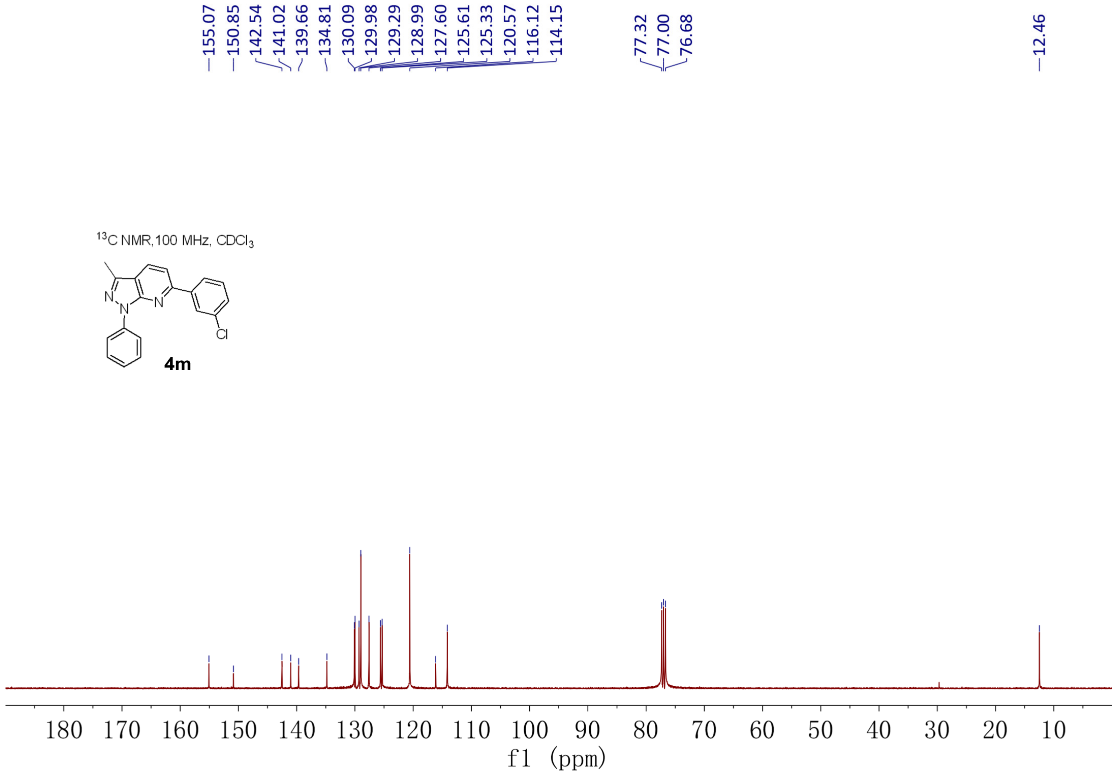
155.07
150.85
142.54
141.02
139.66
134.81
130.09
129.98
129.29
128.99
127.60
125.61
125.33
120.57
116.12
114.15
77.32
77.00
76.68
12.46
^{13}C NMR,100 MHz, $CDCl_3$
N
N
N
Cl
4m
180
170
160
150
140
130
120
110
100
90
80
70
60
50
40
30
20
10
f1 (ppm)

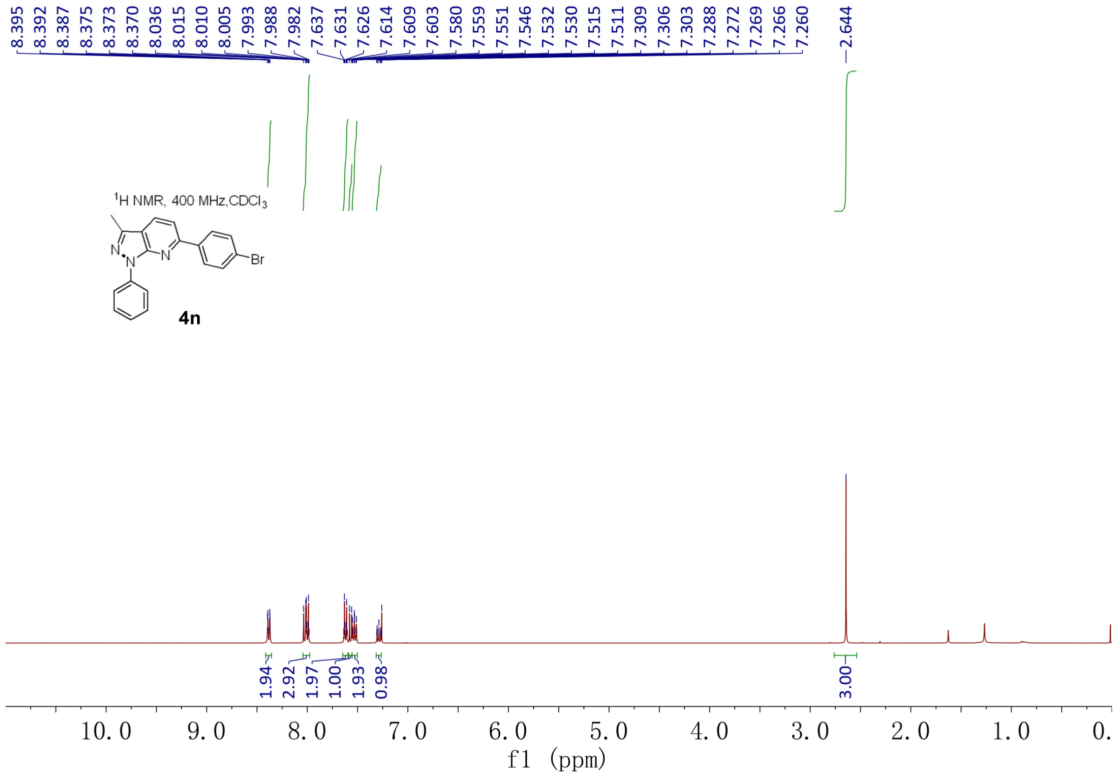
8.395 8.392 8.387 8.375 8.373 8.370 8.036 8.015 8.010 8.005 7.993 7.988 7.982 7.637 7.631 7.626 7.614 7.609 7.603 7.580 7.559 7.551 7.546 7.532 7.530 7.515 7.511 7.309 7.306 7.303 7.288 7.272 7.269 7.266 7.260
2.644
1H NMR, 400 MHz,CDCl3
Br
N
4n
1.94 2.92 1.97 1.00 1.93 0.98
3.00
10.0 9.0 8.0 7.0 6.0 5.0 4.0 3.0 2.0 1.0
f1 (ppm)

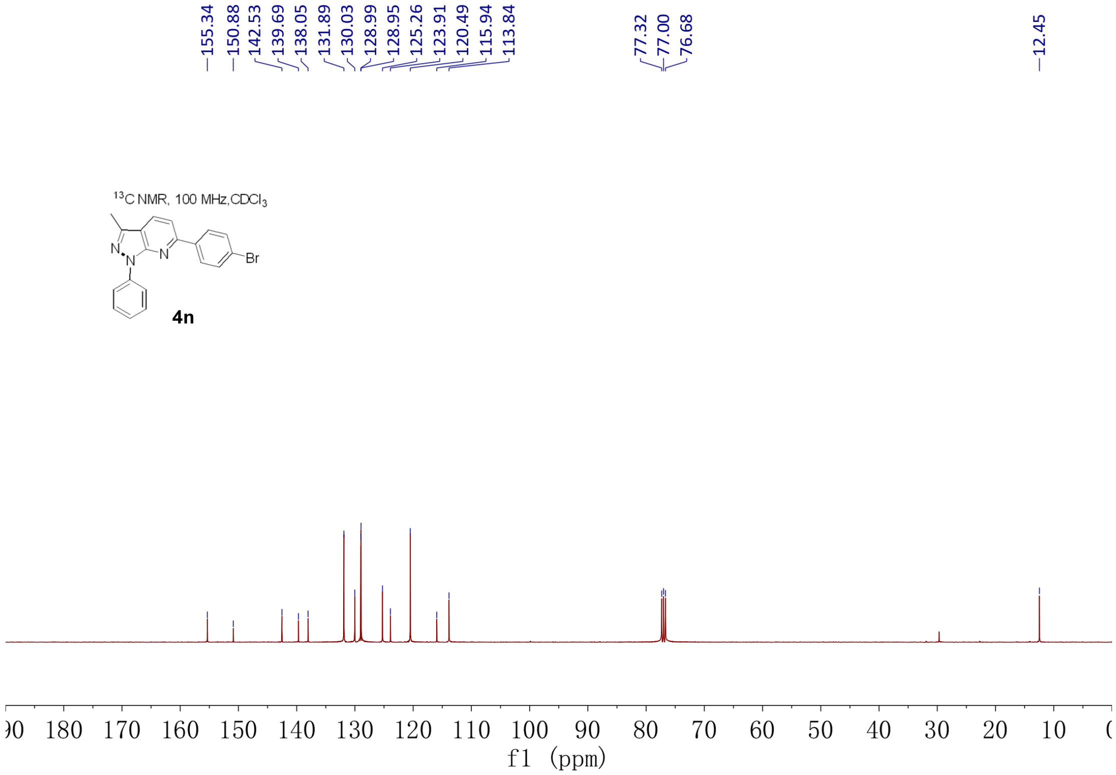
155.34 150.88 142.53 139.69 138.05 131.89 130.03 128.99 128.95 125.26 123.91 120.49 115.94 113.84
77.32 77.00 76.68
12.45
13C NMR, 100 MHz,CDCl3
Br
N
4n
180 170 160 150 140 130 120 110 100 90 80 70 60 50 40 30 20 10
f1 (ppm)

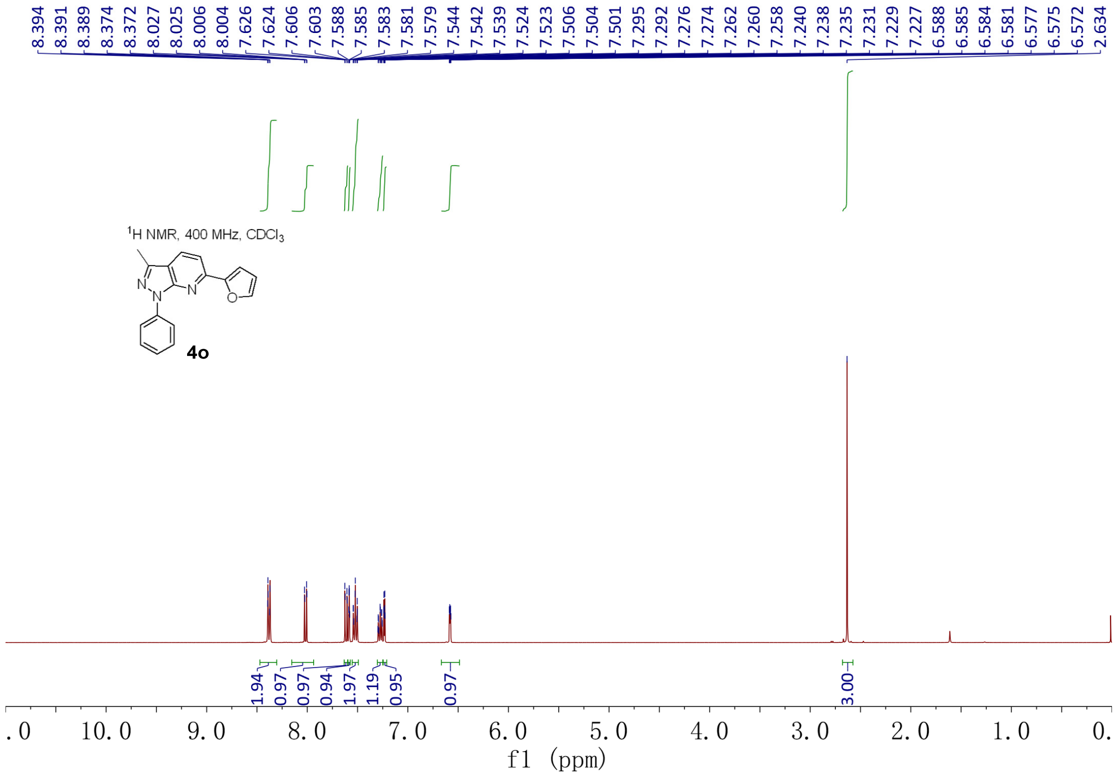
8.394 8.391 8.389 8.374 8.372 8.027 8.025 8.006 8.004 7.626 7.624 7.606 7.603 7.588 7.585 7.583 7.581 7.579 7.544 7.542 7.539 7.524 7.523 7.506 7.504 7.501 7.295 7.292 7.276 7.274 7.262 7.260 7.258 7.240 7.238 7.235 7.231 7.229 7.227 6.588 6.585 6.584 6.581 6.577 6.575 6.572 2.634
^{1}H NMR, 400 MHz, CDCl3
N
N
N
O
4o
1.94 0.97 0.97 0.94 1.97 1.19 0.95 0.97 3.00
f1 (ppm)

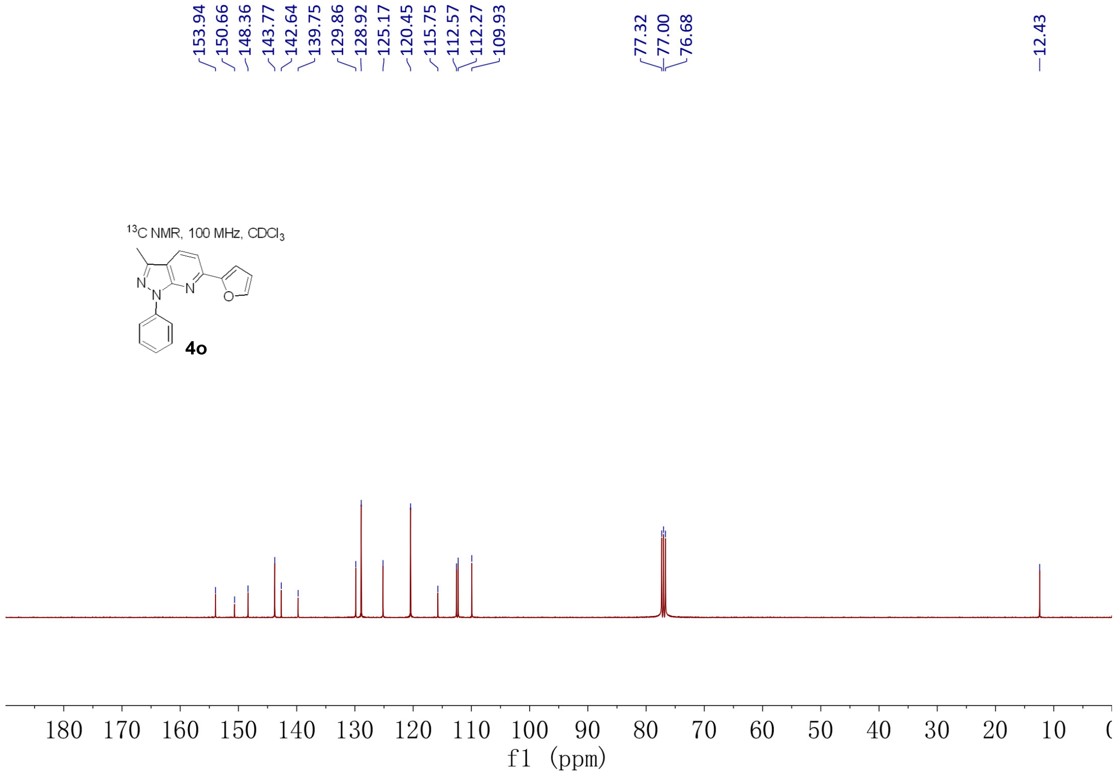
153.94 150.66 148.36 143.77 142.64 139.75 129.86 128.92 125.17 120.45 115.75 112.57 112.27 109.93 77.32 77.00 76.68 12.43
^{13}C NMR, 100 MHz, CDCl3
N
N
N
O
4o
180 170 160 150 140 130 120 110 100 90 80 70 60 50 40 30 20 10
f1 (ppm)

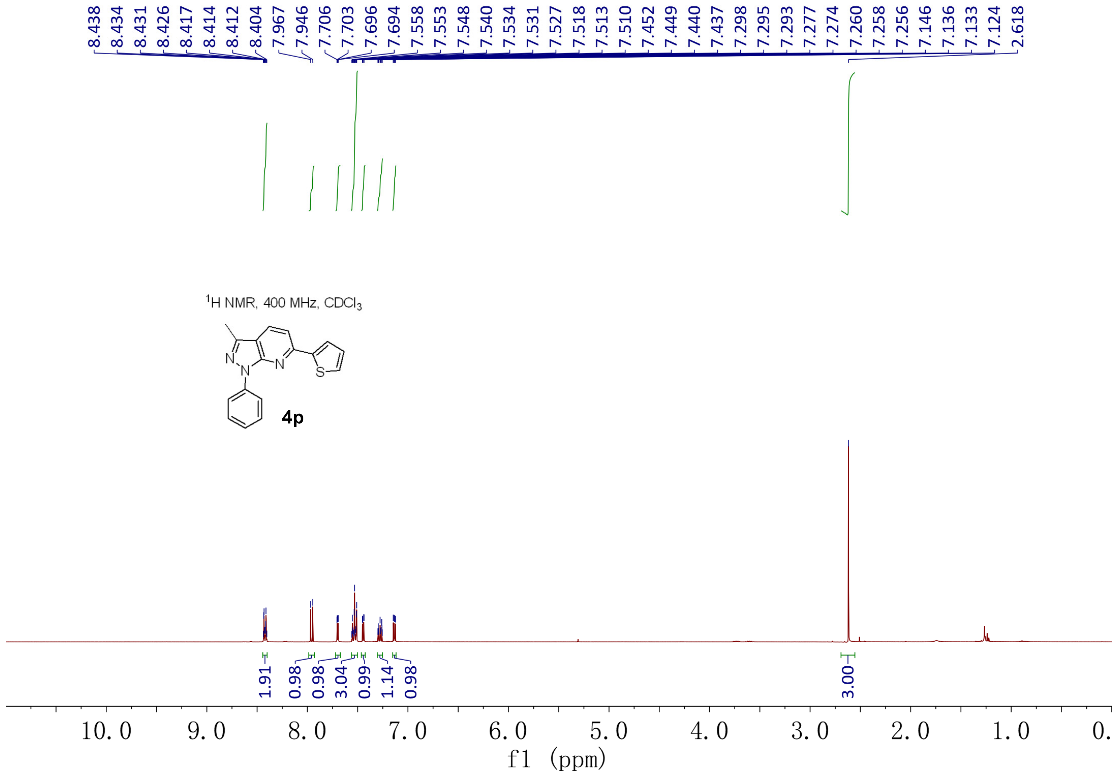
8.438 8.434 8.431 8.426 8.417 8.414 8.412 8.404 7.967 7.946 7.706 7.703 7.696 7.694 7.558 7.553 7.548 7.540 7.534 7.531 7.527 7.518 7.513 7.510 7.452 7.449 7.440 7.437 7.298 7.295 7.293 7.277 7.274 7.260 7.258 7.256 7.146 7.136 7.133 7.124 2.618
^{1}H NMR, 400 MHz, $CDCl_3$
N
N
N
S
4p
1.91 0.98 0.98 3.04 0.99 1.14 0.98
3.00
10.0 9.0 8.0 7.0 6.0 5.0 4.0 3.0 2.0 1.0 0.
f1 (ppm)

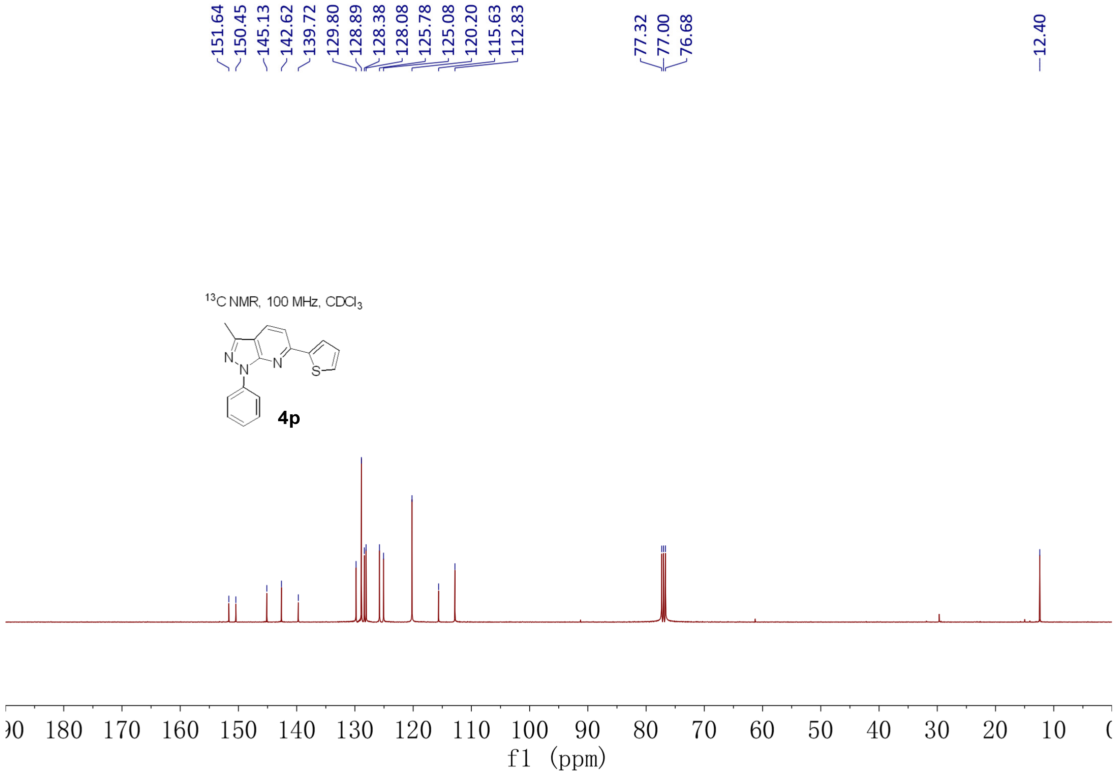
151.64 150.45 145.13 142.62 139.72 129.80 128.89 128.38 128.08 125.78 125.08 120.20 115.63 112.83
77.32 77.00 76.68
12.40
^{13}C NMR, 100 MHz, $CDCl_3$
N
N
N
S
4p
180 170 160 150 140 130 120 110 100 90 80 70 60 50 40 30 20 10
f1 (ppm)

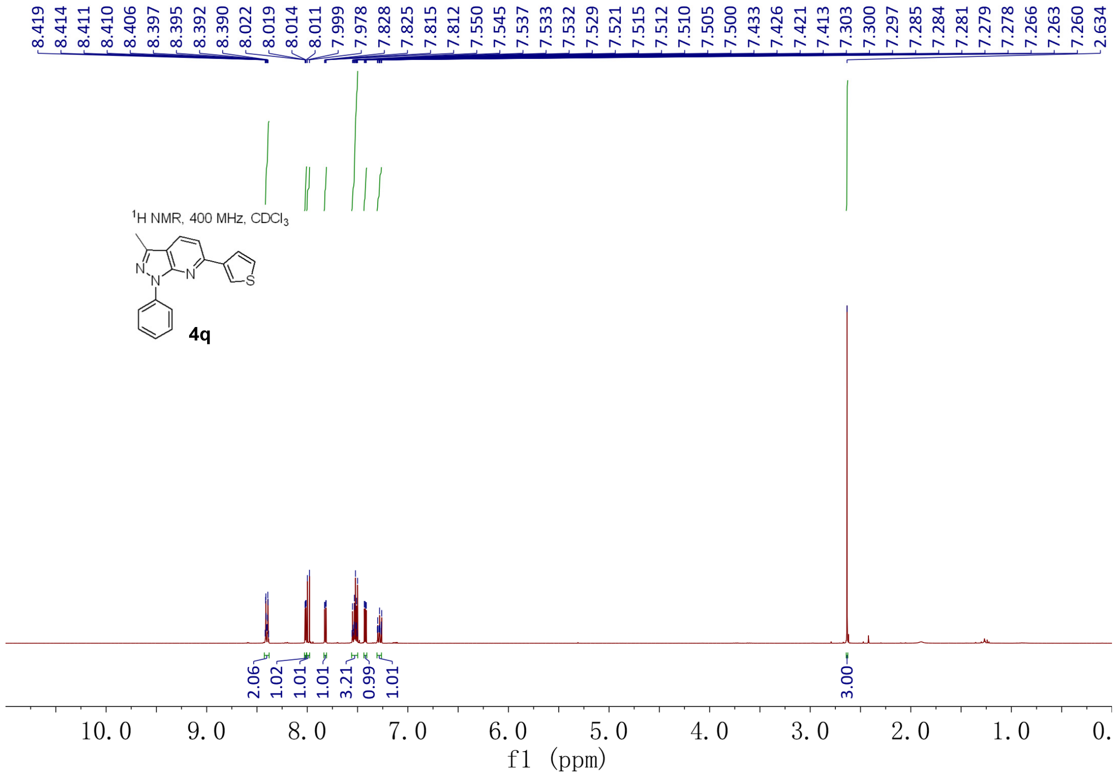
8.419
8.414
8.411
8.410
8.406
8.397
8.395
8.392
8.390
8.022
8.019
8.014
8.011
7.999
7.978
7.828
7.825
7.815
7.812
7.550
7.545
7.537
7.533
7.532
7.529
7.521
7.515
7.512
7.510
7.505
7.500
7.433
7.426
7.421
7.413
7.303
7.300
7.297
7.285
7.284
7.281
7.279
7.278
7.266
7.263
7.260
2.634
1H NMR, 400 MHz, CDCl3
N
N
N
S
4q
2.06
1.02
1.01
1.01
3.21
0.99
1.01
3.00
10.0
9.0
8.0
7.0
6.0
5.0
4.0
3.0
2.0
1.0
f1 (ppm)

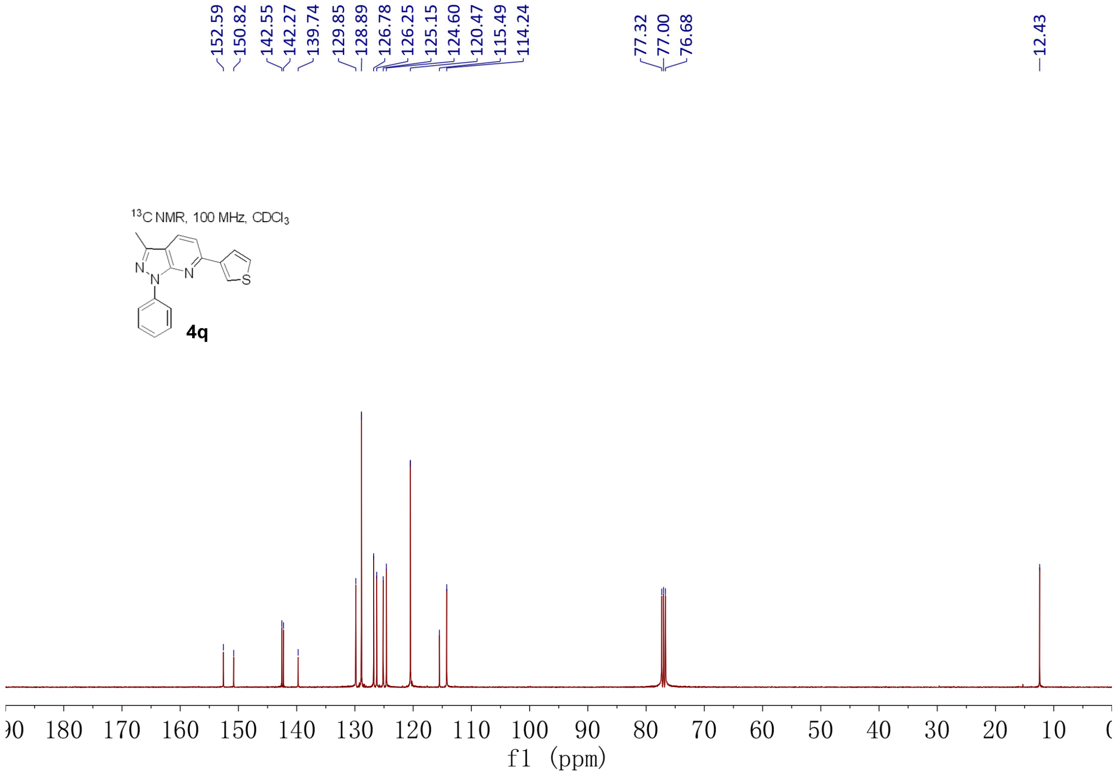
152.59
150.82
142.55
142.27
139.74
129.85
128.89
126.78
126.25
125.15
124.60
120.47
115.49
114.24
77.32
77.00
76.68
12.43
13C NMR, 100 MHz, CDCl3
N
N
N
S
4q
180
170
160
150
140
130
120
110
100
90
80
70
60
50
40
30
20
10
f1 (ppm)

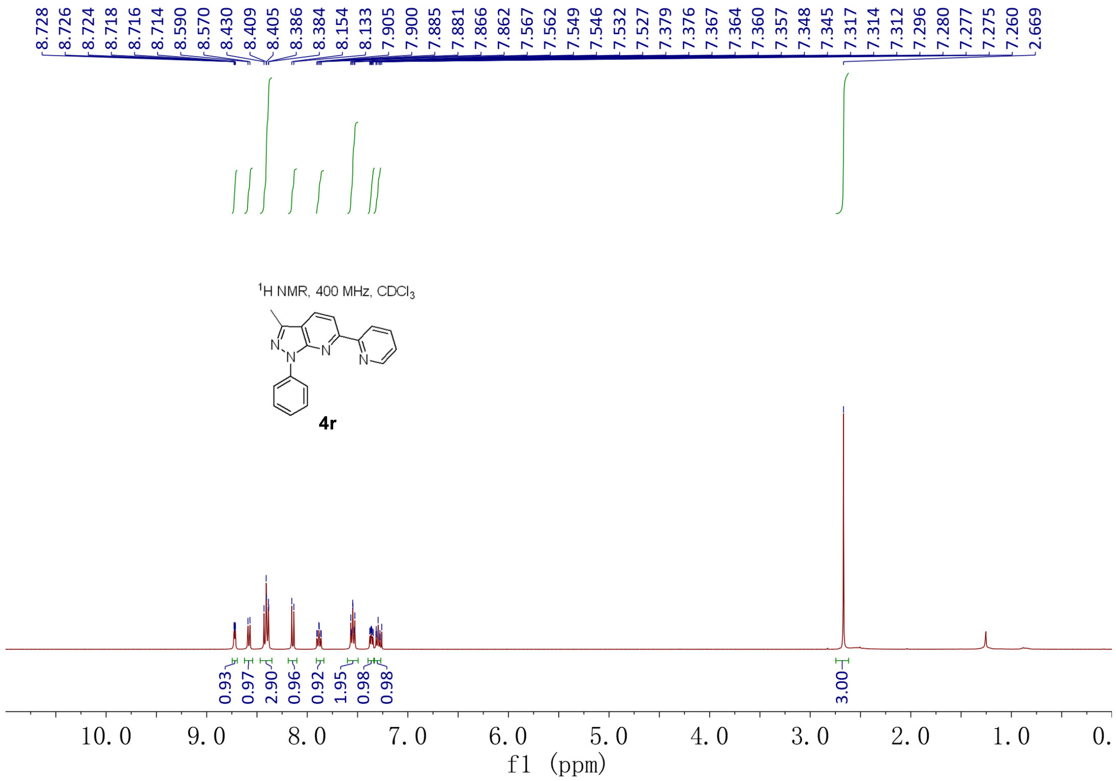
8.728 8.726 8.724 8.718 8.716 8.714 8.590 8.570 8.430 8.409 8.405 8.386 8.384 8.154 8.133 7.905 7.900 7.885 7.881 7.866 7.862 7.567 7.562 7.549 7.546 7.532 7.527 7.379 7.376 7.367 7.364 7.360 7.357 7.348 7.345 7.317 7.314 7.312 7.296 7.280 7.277 7.275 7.260 2.669
^{1}H NMR, 400 MHz, $CDCl_3$
4r
0.93 0.97 2.90 0.96 0.92 1.95 0.98 0.98 3.00
10.0 9.0 8.0 7.0 6.0 5.0 4.0 3.0 2.0 1.0 0.
f1 (ppm)

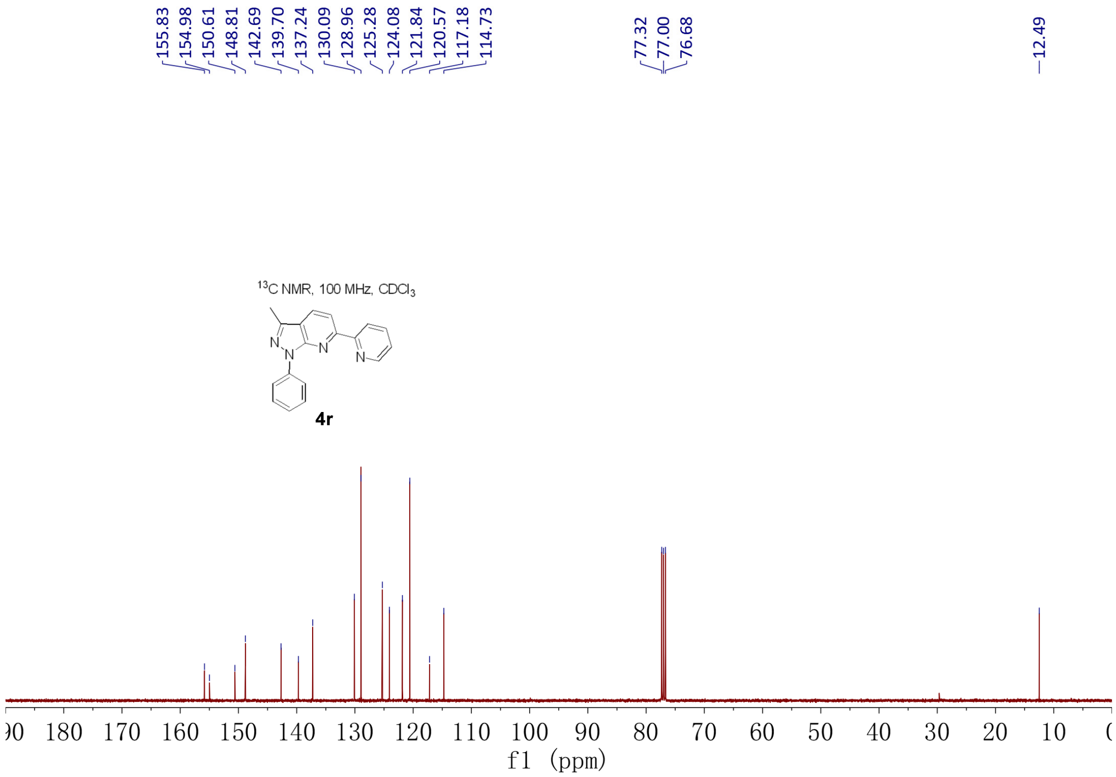
155.83 154.98 150.61 148.81 142.69 139.70 137.24 130.09 128.96 125.28 124.08 121.84 120.57 117.18 114.73
77.32 77.00 76.68
12.49
^{13}C NMR, 100 MHz, $CDCl_3$
4r
180 170 160 150 140 130 120 110 100 90 80 70 60 50 40 30 20 10
f1 (ppm)

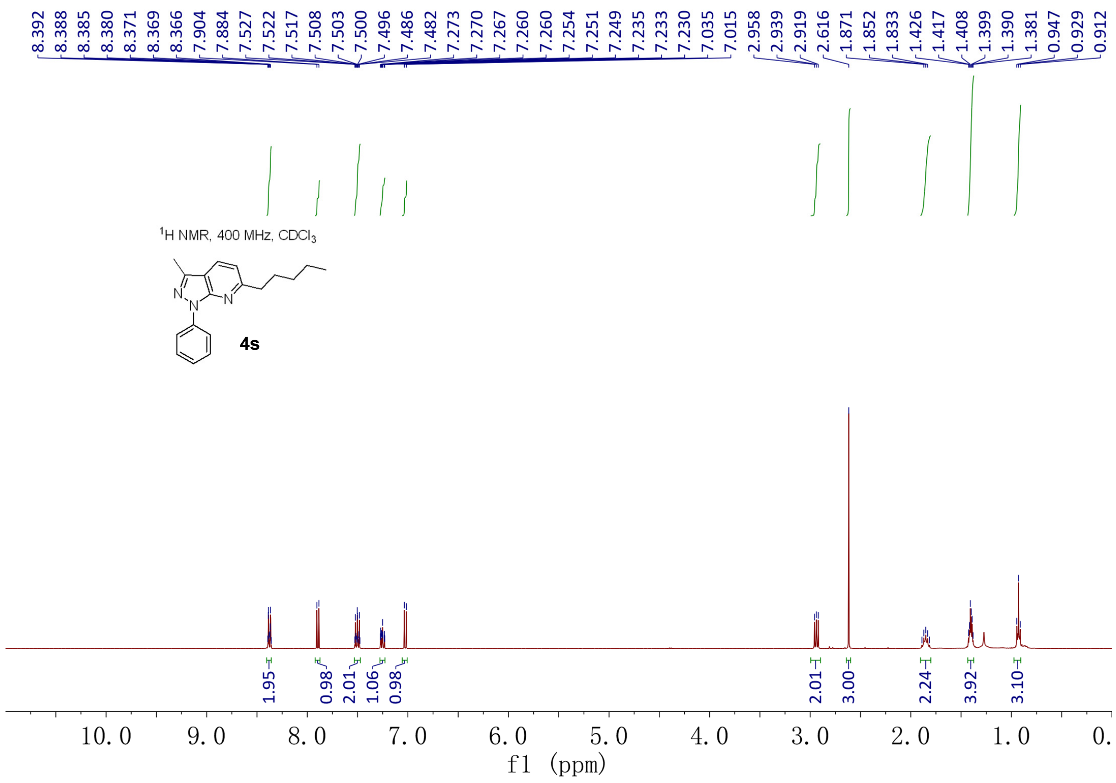
8.392 8.388 8.385 8.380 8.371 8.369 8.366 7.904 7.884 7.527 7.522 7.517 7.508 7.503 7.500 7.496 7.486 7.482 7.273 7.270 7.267 7.260 7.260 7.254 7.251 7.249 7.235 7.233 7.230 7.035 7.015 2.958 2.939 2.919 2.616 1.871 1.852 1.833 1.426 1.417 1.408 1.399 1.390 1.381 0.947 0.929 0.912
1H NMR, 400 MHz, CDCl3
N
N
N
4s
1.95
0.98
2.01
1.06
0.98
2.01
3.00
2.24
3.92
3.10
10.0 9.0 8.0 7.0 6.0 5.0 4.0 3.0 2.0 1.0 0.
f1 (ppm)

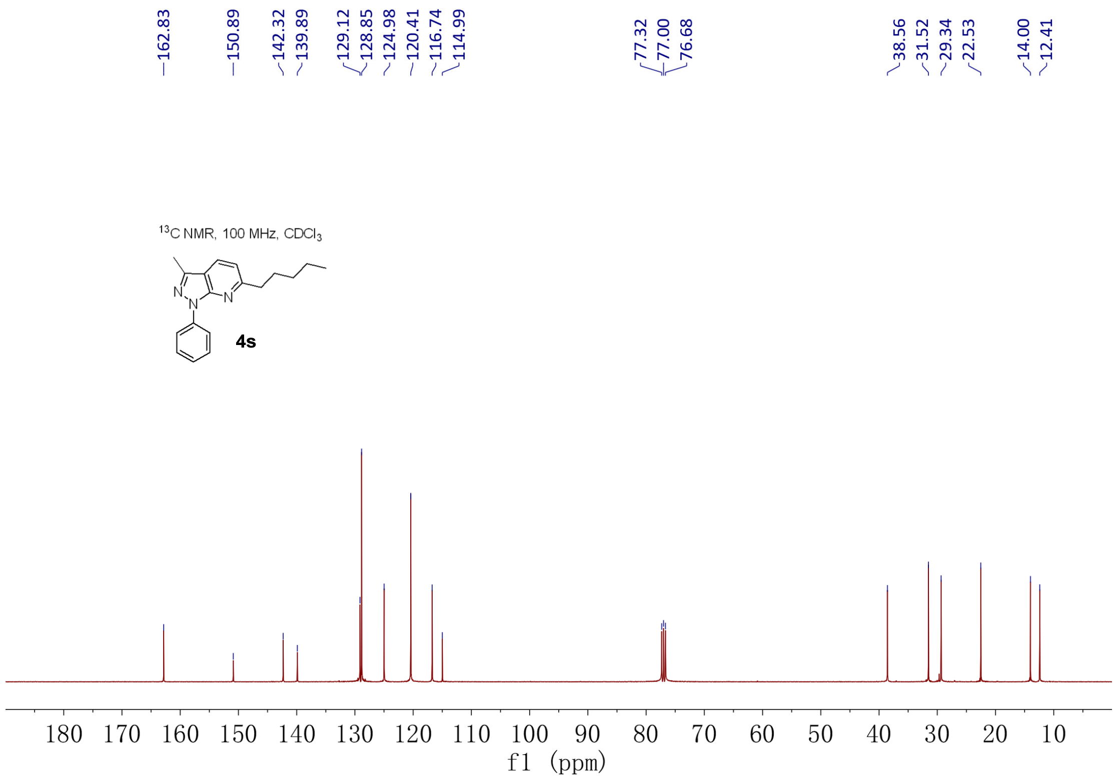
162.83
150.89
142.32 139.89
129.12 128.85 124.98 120.41 116.74 114.99
77.32 77.00 76.68
38.56 31.52 29.34 22.53
14.00 12.41
13C NMR, 100 MHz, CDCl3
N
N
N
4s
180 170 160 150 140 130 120 110 100 90 80 70 60 50 40 30 20 10
f1 (ppm)

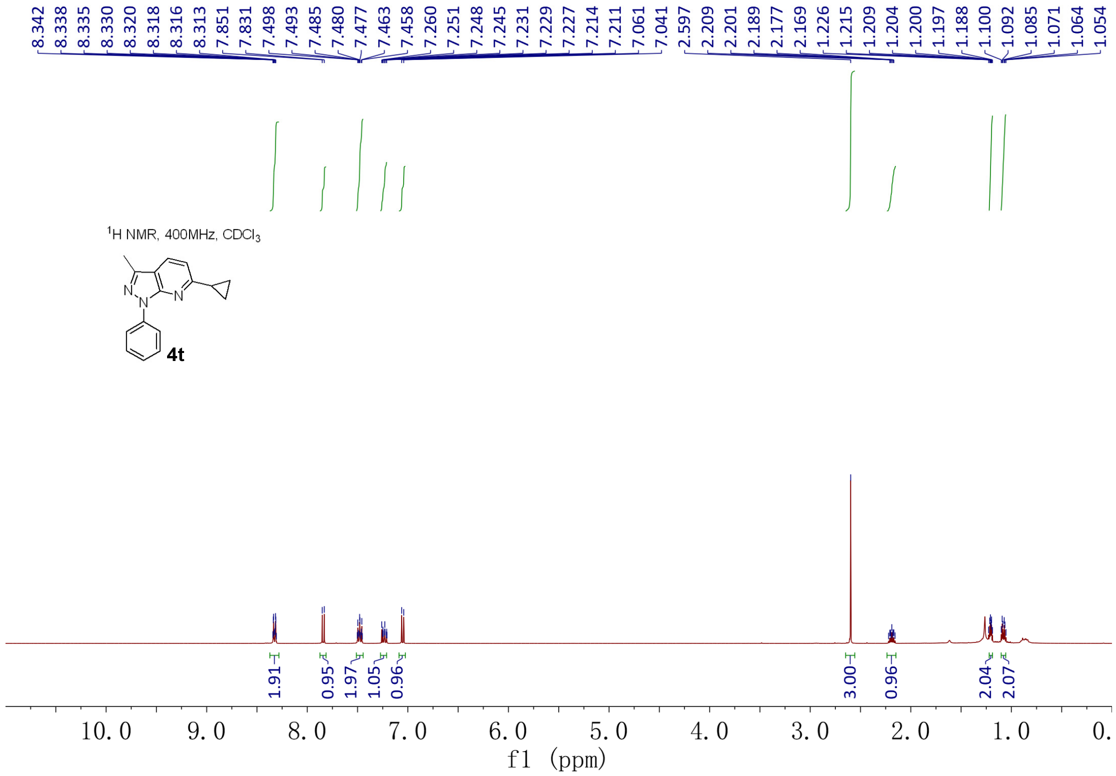
8.342 8.338 8.335 8.330 8.320 8.318 8.316 8.313 7.851 7.831 7.498 7.493 7.485 7.480 7.477 7.463 7.458 7.260 7.251 7.248 7.245 7.231 7.229 7.227 7.214 7.211 7.061 7.041 2.597 2.209 2.201 2.189 2.177 2.169 1.226 1.215 1.209 1.204 1.200 1.197 1.188 1.100 1.092 1.085 1.071 1.064 1.054
1H NMR, 400MHz, CDCl3
4t
1.91 0.95 1.97 1.05 0.96 3.00 0.96 2.04 2.07
10.0 9.0 8.0 7.0 6.0 5.0 4.0 3.0 2.0 1.0 0.
f1 (ppm)

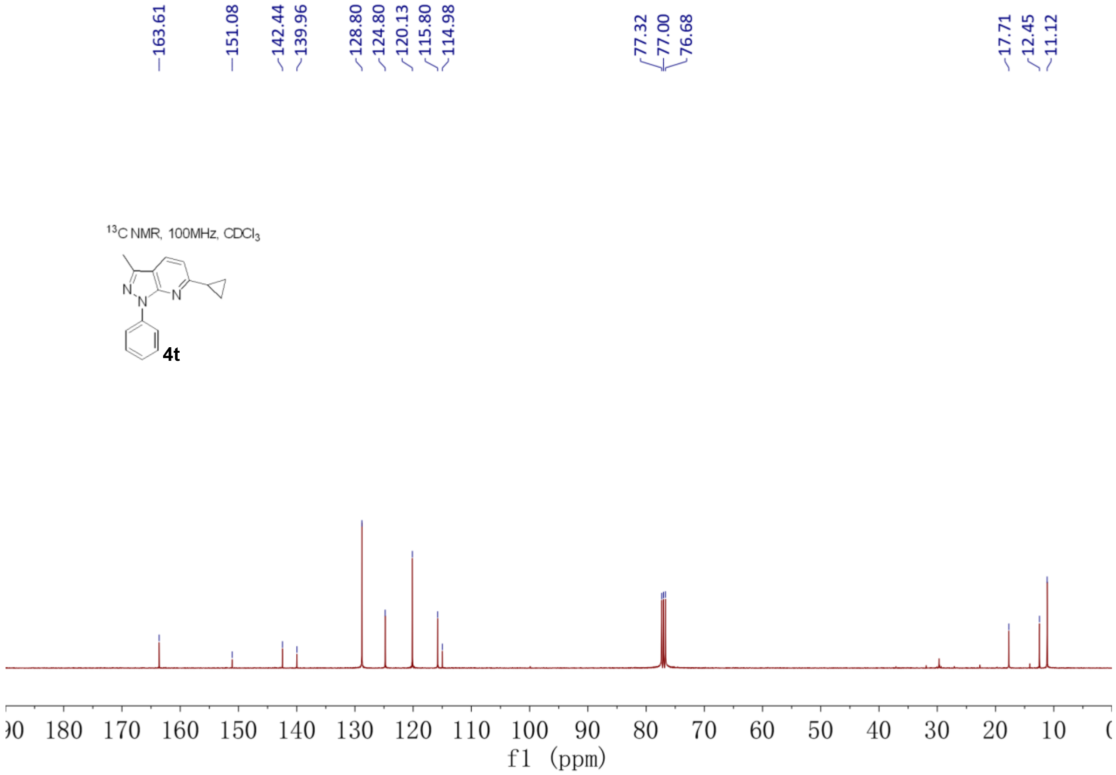
163.61 151.08 142.44 139.96 128.80 124.80 120.13 115.80 114.98 77.32 77.00 76.68 17.71 12.45 11.12
13C NMR, 100MHz, CDCl3
4t
180 170 160 150 140 130 120 110 100 90 80 70 60 50 40 30 20 10
f1 (ppm)

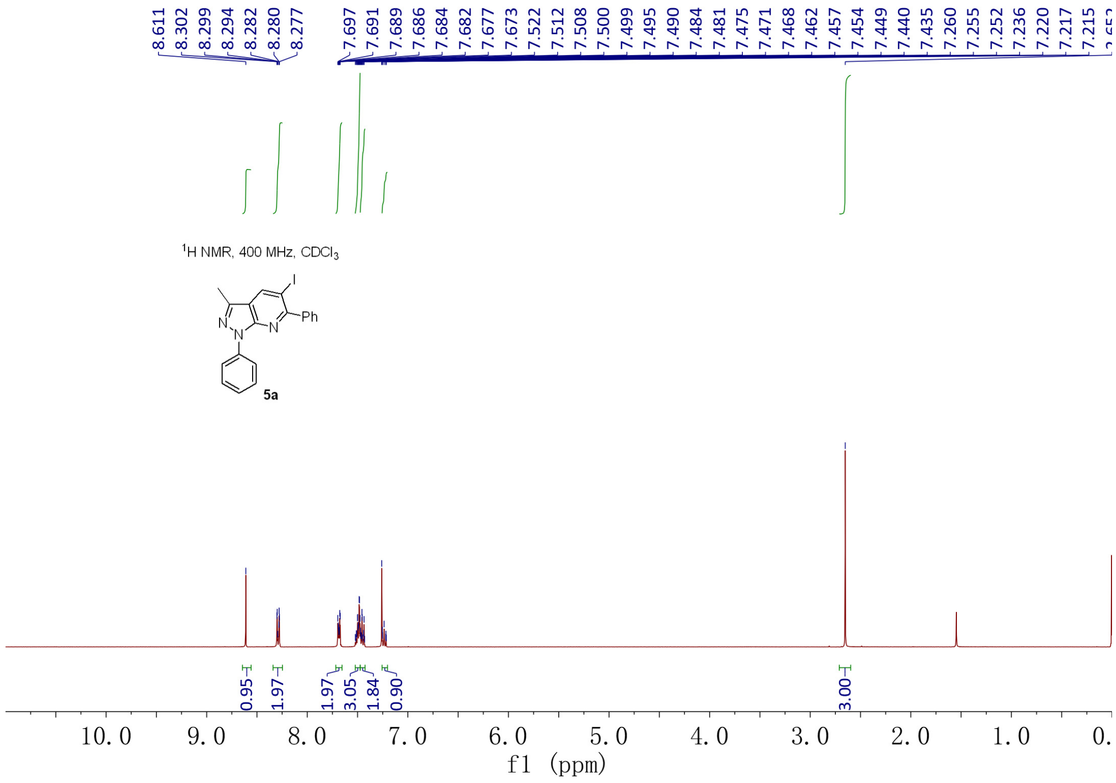

8.611
8.302
8.299
8.294
8.282
8.280
8.277
7.697
7.691
7.689
7.686
7.684
7.682
7.677
7.673
7.522
7.512
7.508
7.500
7.499
7.495
7.490
7.484
7.481
7.475
7.471
7.468
7.462
7.457
7.454
7.449
7.440
7.435
7.260
7.255
7.252
7.236
7.220
7.217
7.215
¹H NMR, 400 MHz, CDCl₃
Ph
I
N
5a
0.95
1.97
1.97
3.05
1.84
0.90
3.00
10.0
9.0
8.0
7.0
6.0
5.0
4.0
3.0
2.0
1.0
f1 (ppm)

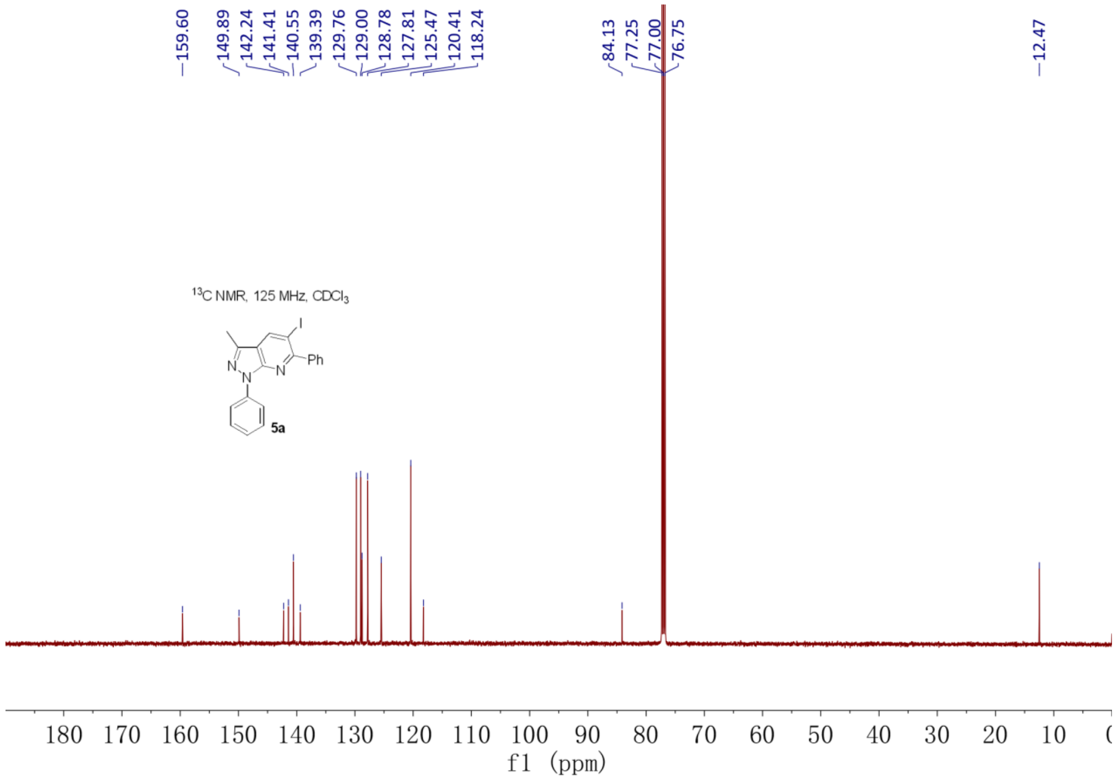

159.60
149.89
142.24
141.41
140.55
139.39
129.76
129.00
128.78
127.81
125.47
120.41
118.24
84.13
77.25
77.00
76.75
12.47
¹³C NMR, 125 MHz, CDCl₃
Ph
I
N
5a
180
170
160
150
140
130
120
110
100
90
80
70
60
50
40
30
20
10
f1 (ppm)

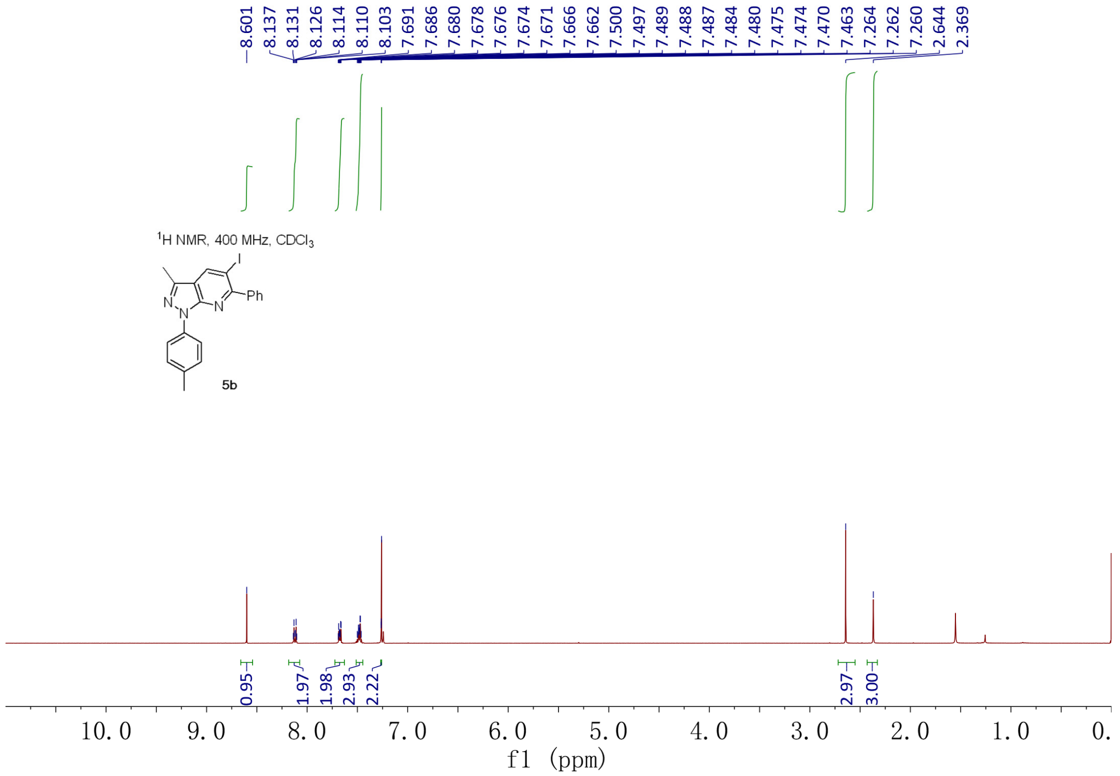
8.601
8.137
8.131
8.126
8.114
8.110
8.103
7.691
7.686
7.680
7.678
7.676
7.674
7.671
7.666
7.662
7.500
7.497
7.489
7.488
7.487
7.484
7.480
7.475
7.474
7.470
7.463
7.264
7.262
7.260
2.644
2.369
¹H NMR, 400 MHz, CDCl₃
Ph
N
N
N
I
5b
0.95
1.97
1.98
2.93
2.22
2.97
3.00
10.0
9.0
8.0
7.0
6.0
5.0
4.0
3.0
2.0
1.0
0.
f1 (ppm)

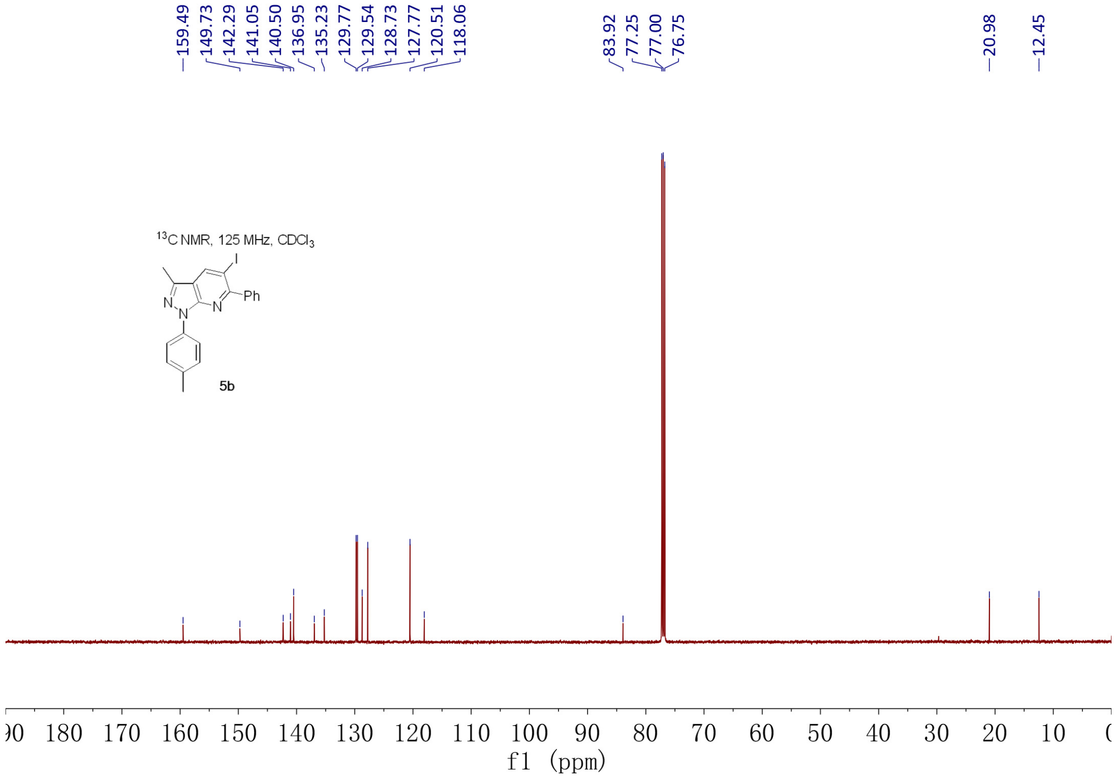
159.49
149.73
142.29
141.05
140.50
136.95
135.23
129.77
129.54
128.73
127.77
120.51
118.06
83.92
77.25
77.00
76.75
20.98
12.45
¹³C NMR, 125 MHz, CDCl₃
Ph
N
N
N
I
5b
180
170
160
150
140
130
120
110
100
90
80
70
60
50
40
30
20
10
f1 (ppm)

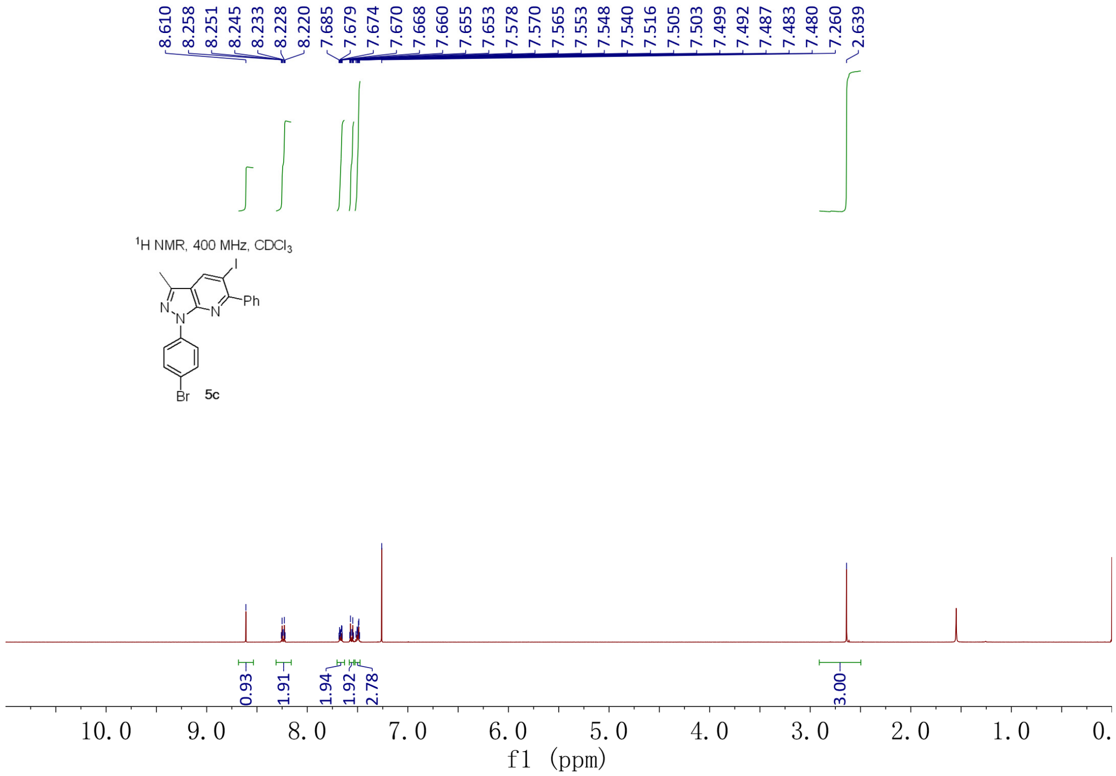
8.610
8.258
8.251
8.245
8.233
8.228
8.220
7.685
7.679
7.674
7.670
7.668
7.660
7.655
7.653
7.578
7.570
7.565
7.553
7.548
7.540
7.516
7.505
7.503
7.499
7.492
7.487
7.483
7.480
7.260
2.639
^{1}H NMR, 400 MHz, $CDCl_3$
I
Ph
N
N
N
Br
5c
0.93
1.91
1.94
1.92
2.78
3.00
10.0 9.0 8.0 7.0 6.0 5.0 4.0 3.0 2.0 1.0 0.
f1 (ppm)

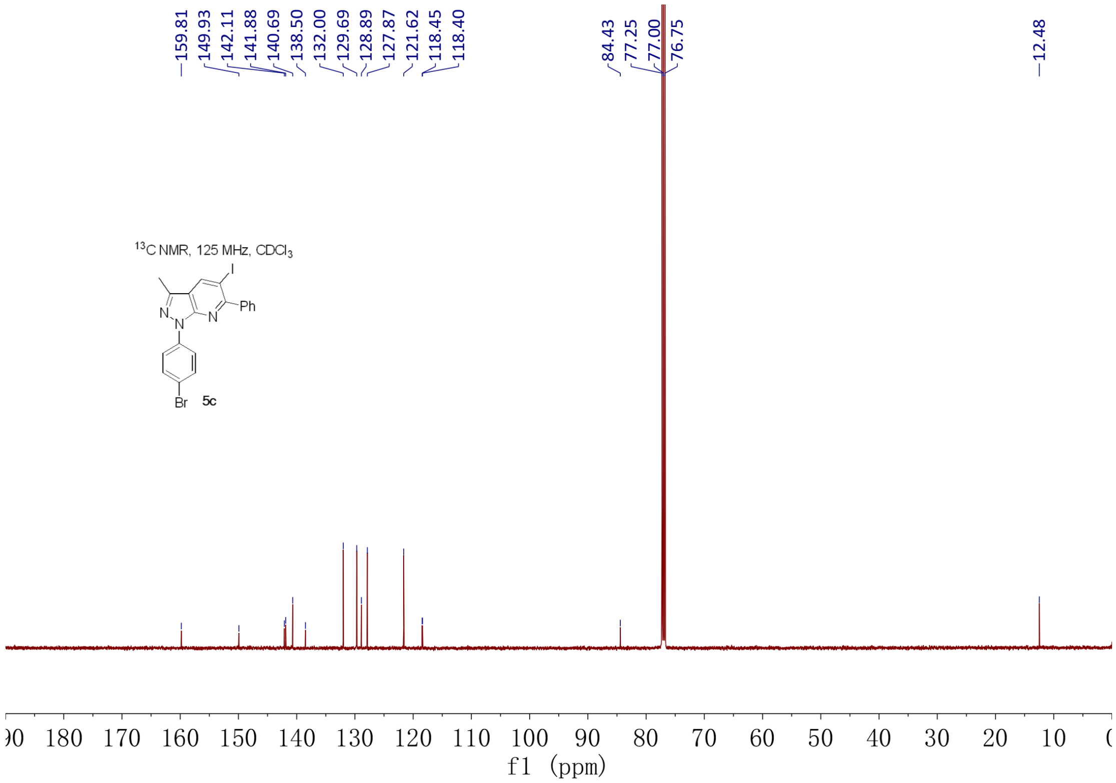
159.81
149.93
142.11
141.88
140.69
138.50
132.00
129.69
128.89
127.87
121.62
118.45
118.40
84.43
77.25
77.00
76.75
12.48
^{13}C NMR, 125 MHz, $CDCl_3$
I
Ph
N
N
N
Br
5c
180 170 160 150 140 130 120 110 100 90 80 70 60 50 40 30 20 10
f1 (ppm)

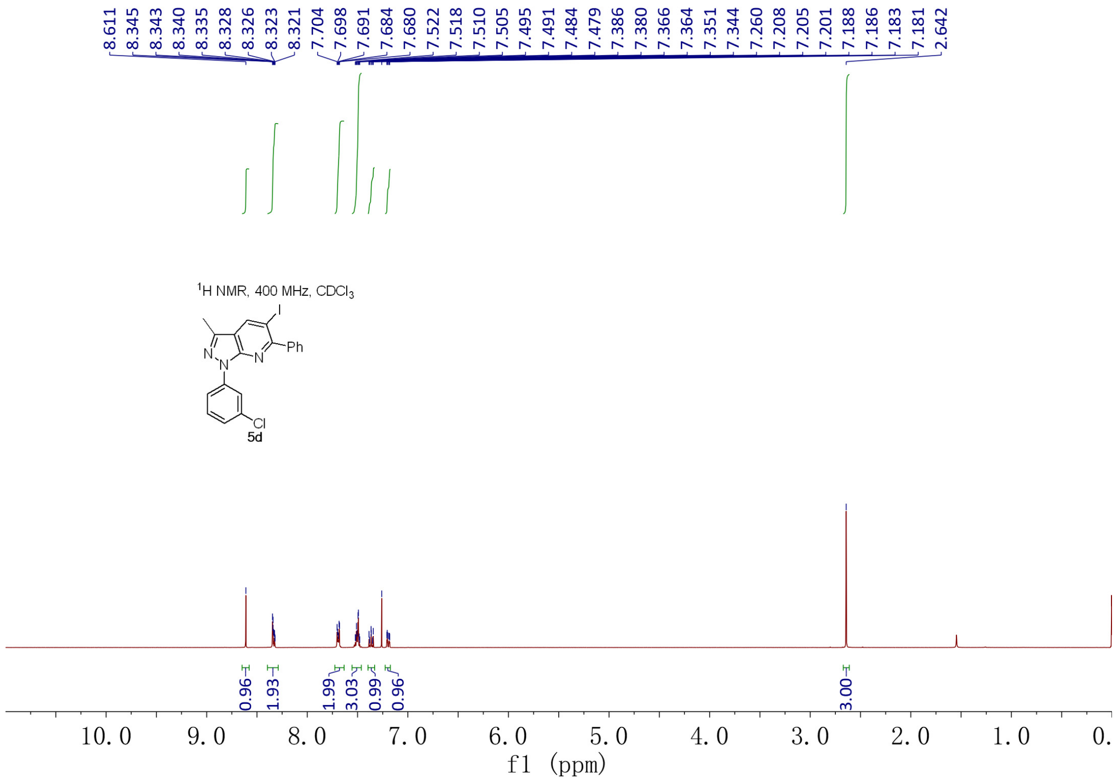
8.611
8.345
8.343
8.340
8.335
8.328
8.326
8.323
8.321
7.704
7.698
7.691
7.684
7.680
7.522
7.518
7.510
7.505
7.495
7.491
7.484
7.479
7.386
7.380
7.366
7.364
7.351
7.344
7.260
7.208
7.205
7.201
7.188
7.186
7.183
7.181
2.642
^{1}H NMR, 400 MHz, CDCl$_3$
I
Ph
N
N
N
Cl
5d
0.96
1.93
1.99
3.03
0.99
0.96
3.00
10.0
9.0
8.0
7.0
6.0
5.0
4.0
3.0
2.0
1.0
f1 (ppm)

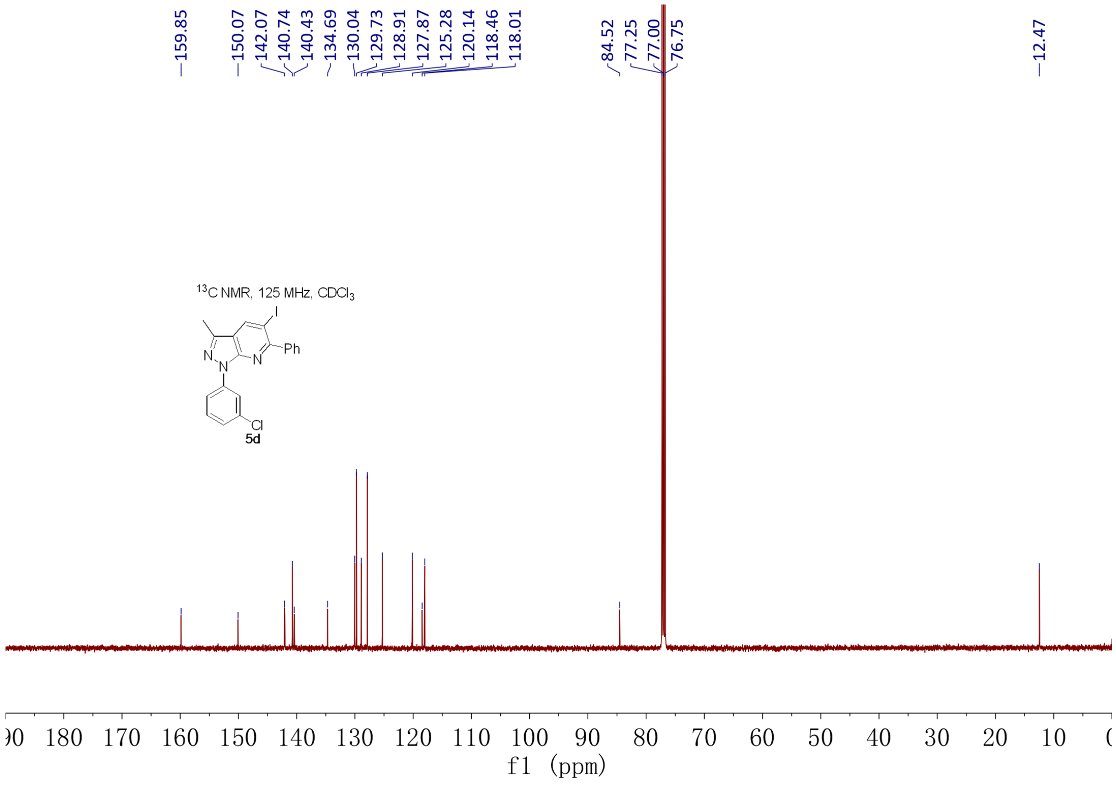
159.85
150.07
142.07
140.74
140.43
134.69
130.04
129.73
128.91
127.87
125.28
120.14
118.46
118.01
84.52
77.25
77.00
76.75
12.47
^{13}C NMR, 125 MHz, CDCl$_3$
I
Ph
N
N
N
Cl
5d
180
170
160
150
140
130
120
110
100
90
80
70
60
50
40
30
20
10
f1 (ppm)

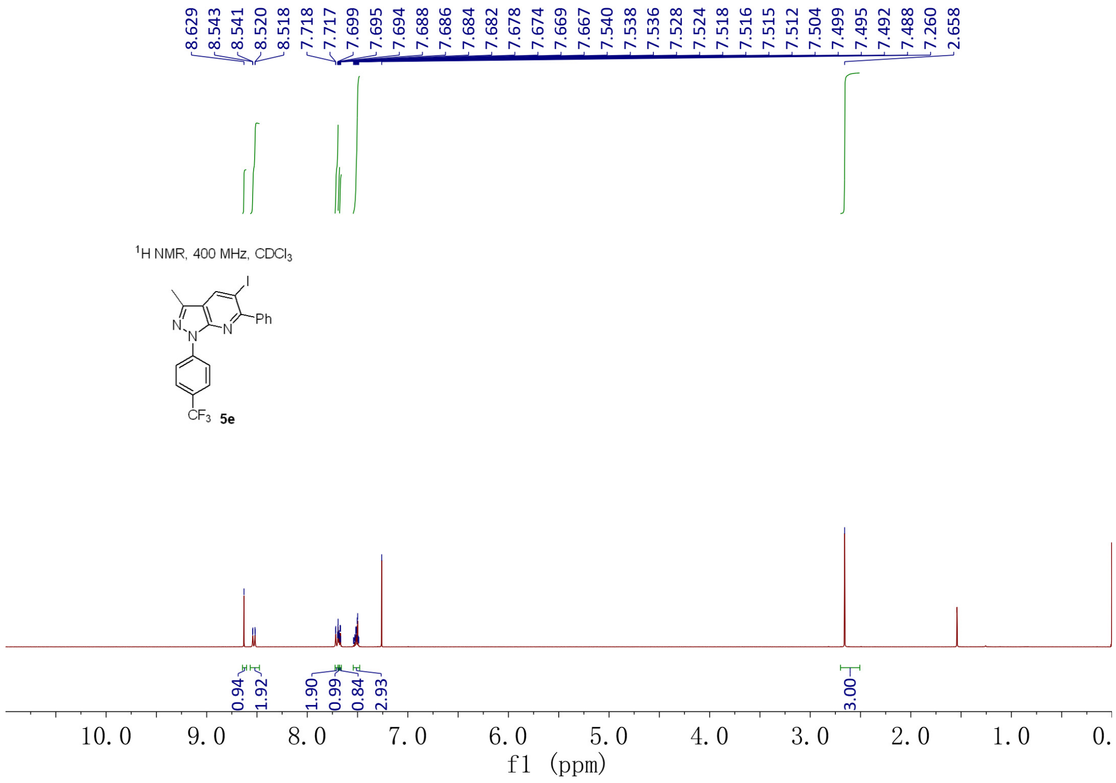
8.629
8.543
8.541
8.520
8.518
7.718
7.717
7.699
7.695
7.694
7.688
7.686
7.684
7.682
7.678
7.674
7.669
7.667
7.540
7.538
7.536
7.528
7.524
7.518
7.516
7.515
7.512
7.504
7.499
7.495
7.492
7.488
7.260
2.658
^{1}H NMR, 400 MHz, CDCl$_3$
I
Ph
N
N
N
CF$_3$
5e
0.94
1.92
1.90
0.99
0.84
2.93
3.00
10.0
9.0
8.0
7.0
6.0
5.0
4.0
3.0
2.0
1.0
f1 (ppm)

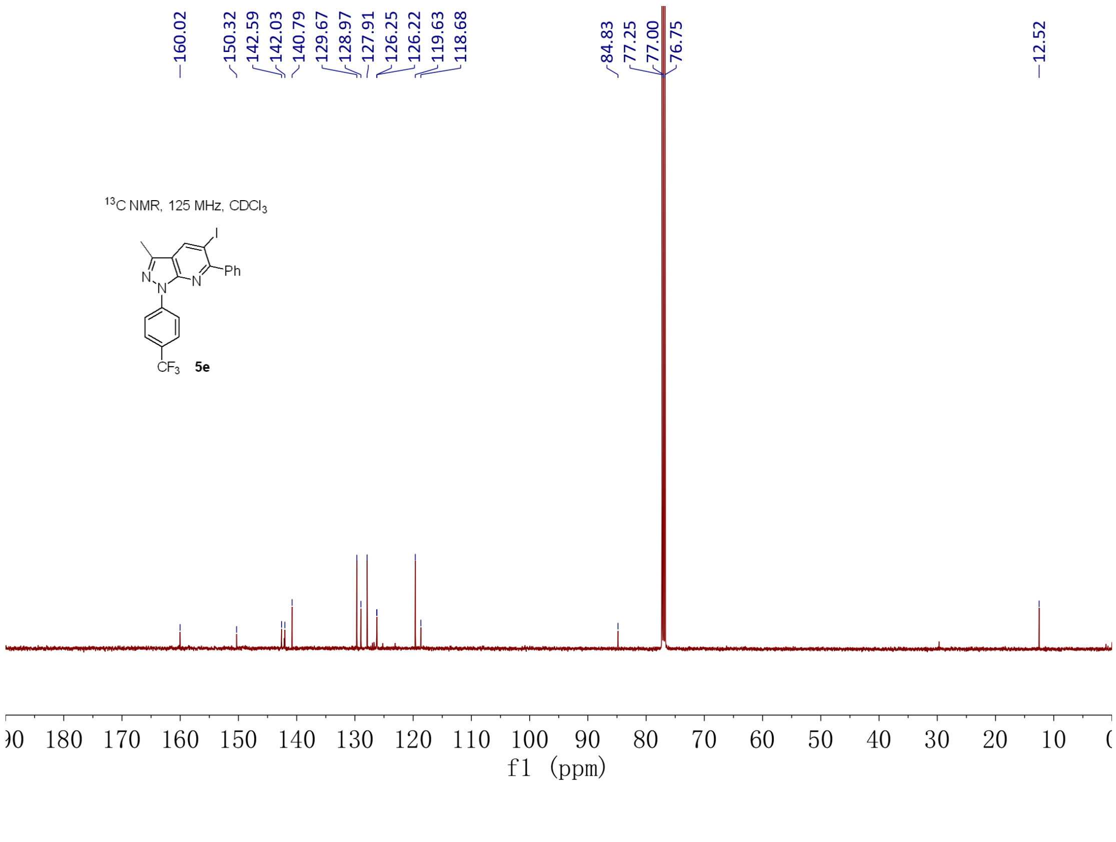
160.02
150.32
142.59
142.03
140.79
129.67
128.97
127.91
126.25
126.22
119.63
118.68
84.83
77.25
77.00
76.75
12.52
^{13}C NMR, 125 MHz, CDCl$_3$
I
Ph
N
N
N
CF$_3$
5e
180
170
160
150
140
130
120
110
100
90
80
70
60
50
40
30
20
10
f1 (ppm)

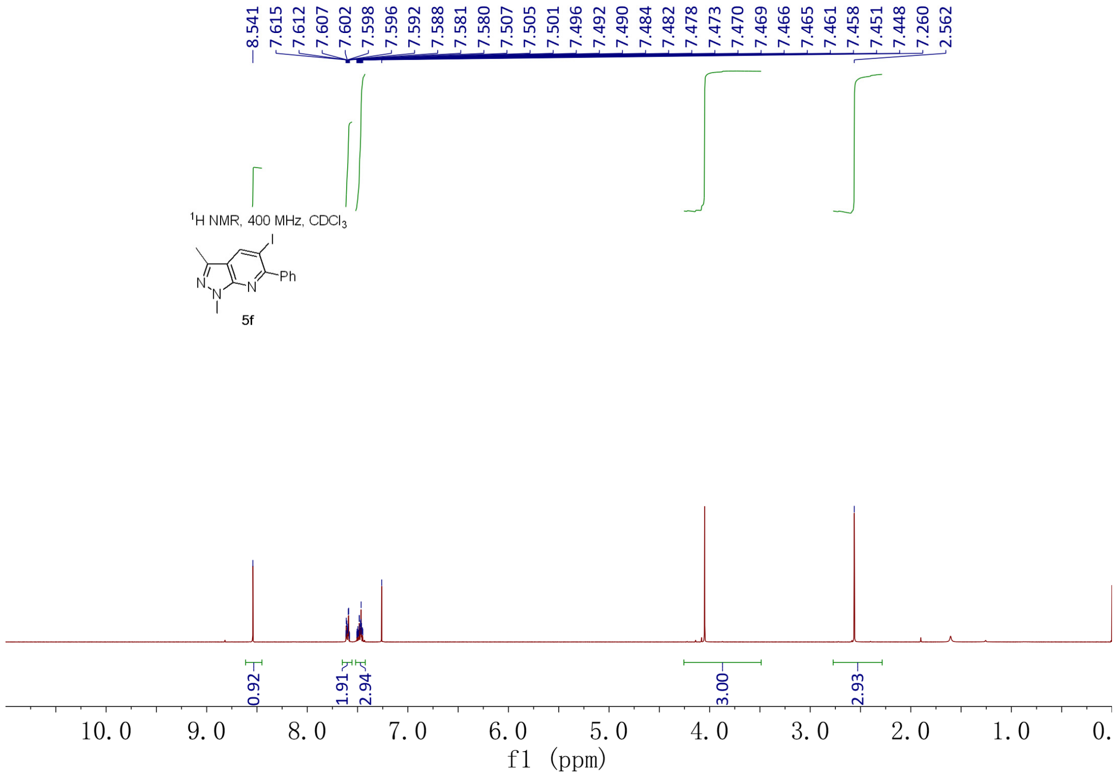
8.541
7.615
7.612
7.607
7.602
7.598
7.596
7.592
7.588
7.581
7.580
7.507
7.505
7.501
7.496
7.492
7.490
7.484
7.482
7.478
7.473
7.470
7.469
7.466
7.465
7.461
7.458
7.451
7.448
7.260
2.562
1H NMR, 400 MHz, CDCl3
I
Ph
N
N
N
5f
0.92
1.91
2.94
3.00
2.93
10.0 9.0 8.0 7.0 6.0 5.0 4.0 3.0 2.0 1.0 0.
f1 (ppm)

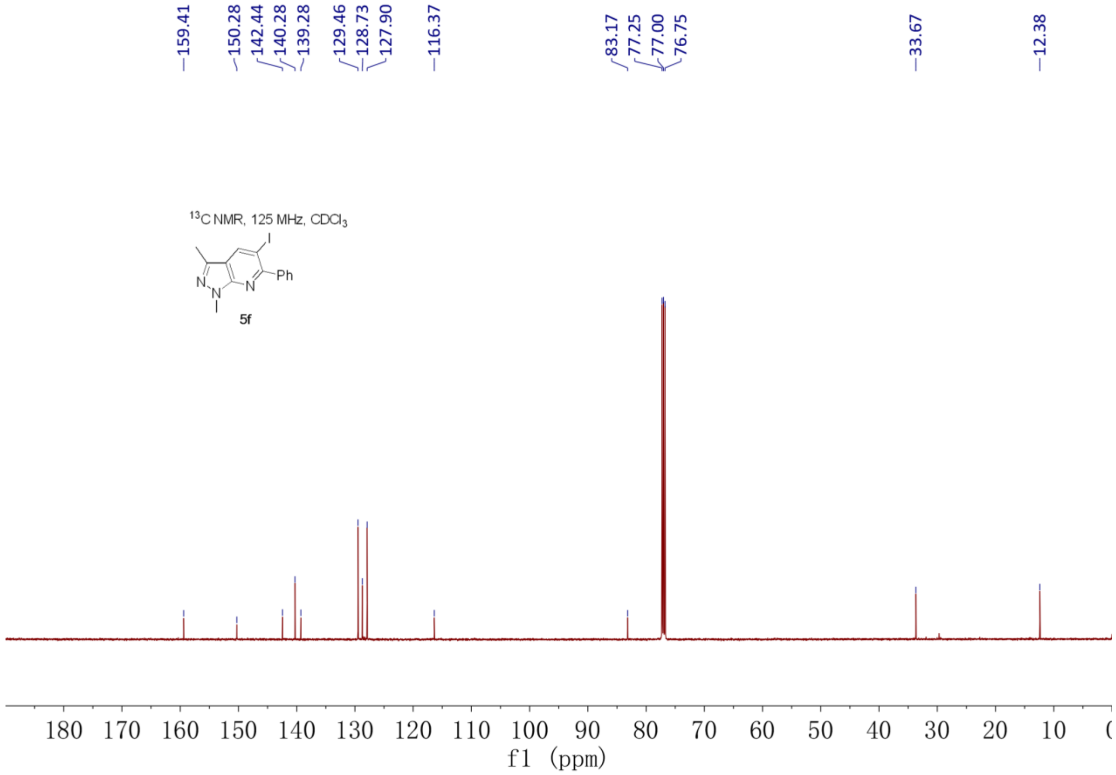
159.41
150.28
142.44
140.28
139.28
129.46
128.73
127.90
116.37
83.17
77.25
77.00
76.75
33.67
12.38
13C NMR, 125 MHz, CDCl3
I
Ph
N
N
N
5f
180 170 160 150 140 130 120 110 100 90 80 70 60 50 40 30 20 10
f1 (ppm)

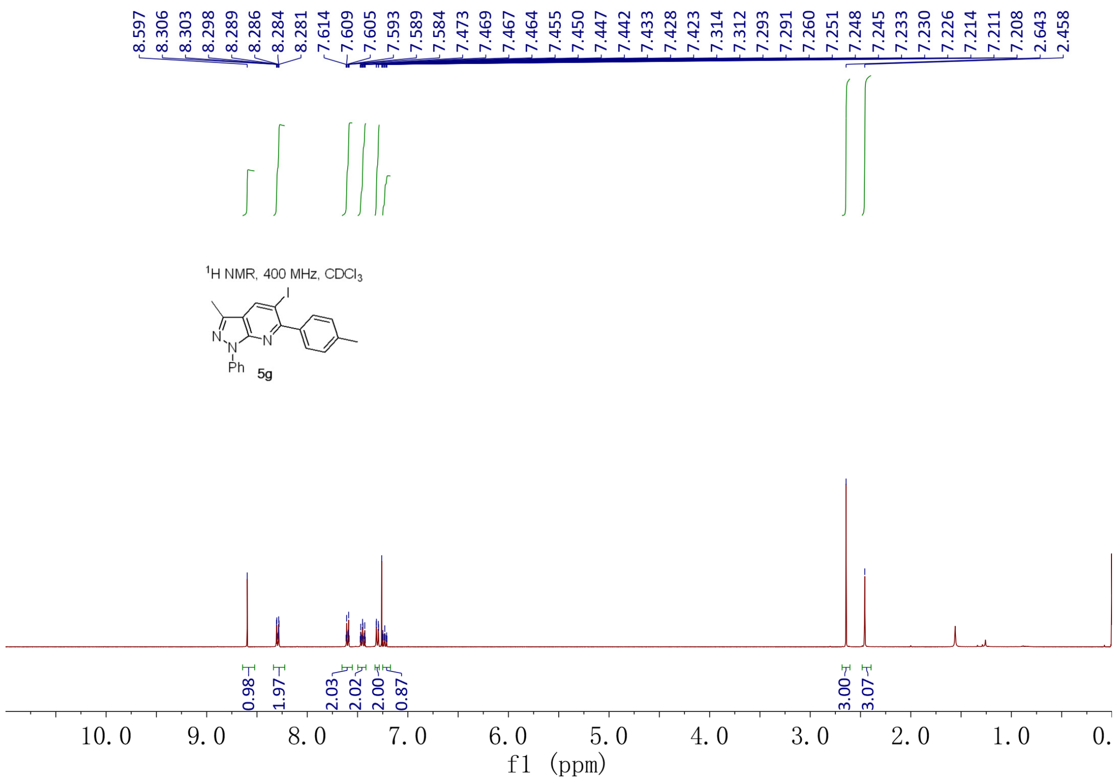
8.597
8.306
8.303
8.298
8.289
8.286
8.284
8.281
7.614
7.609
7.605
7.593
7.589
7.584
7.473
7.469
7.467
7.464
7.455
7.450
7.447
7.442
7.433
7.428
7.423
7.314
7.312
7.293
7.291
7.260
7.251
7.248
7.245
7.233
7.230
7.226
7.214
7.211
7.208
2.643
2.458
¹H NMR, 400 MHz, CDCl3
I
N
N
N
Ph
5g
0.98
1.97
2.03
2.02
2.00
0.87
3.00
3.07
10.0
9.0
8.0
7.0
6.0
5.0
4.0
3.0
2.0
1.0
f1 (ppm)

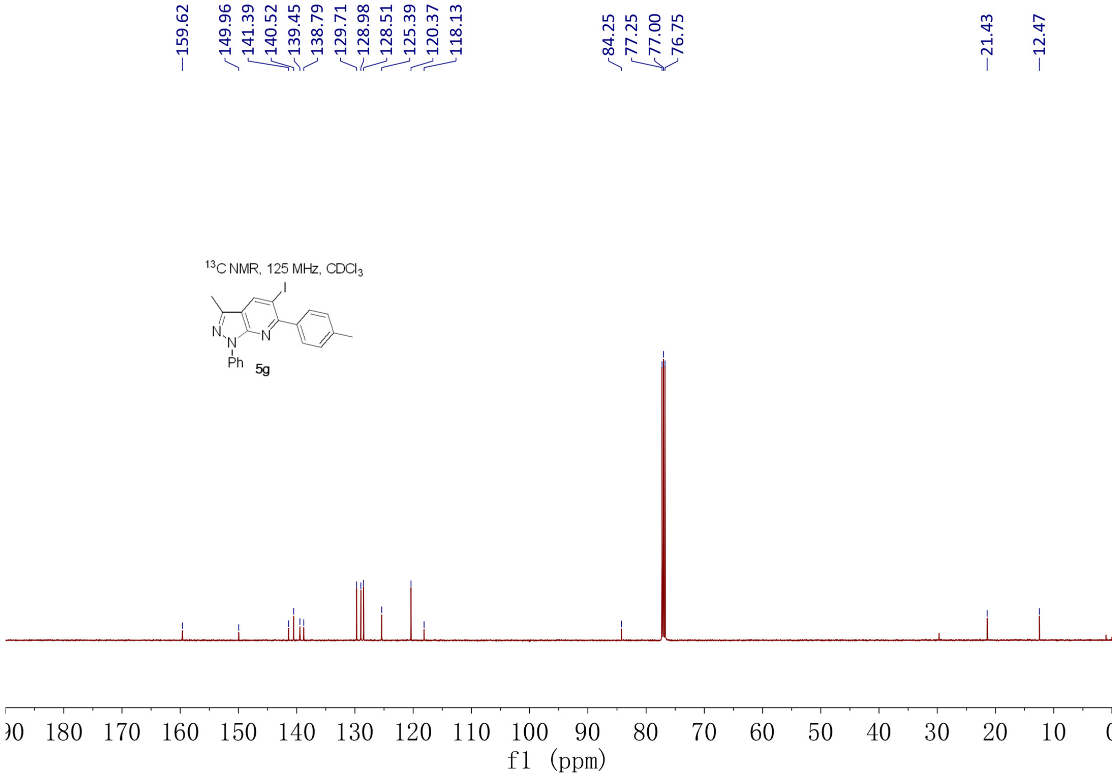
159.62
149.96
141.39
140.52
139.45
138.79
129.71
128.98
128.51
125.39
120.37
118.13
84.25
77.25
77.00
76.75
21.43
12.47
¹³C NMR, 125 MHz, CDCl3
I
N
N
N
Ph
5g
180
170
160
150
140
130
120
110
100
90
80
70
60
50
40
30
20
10
f1 (ppm)

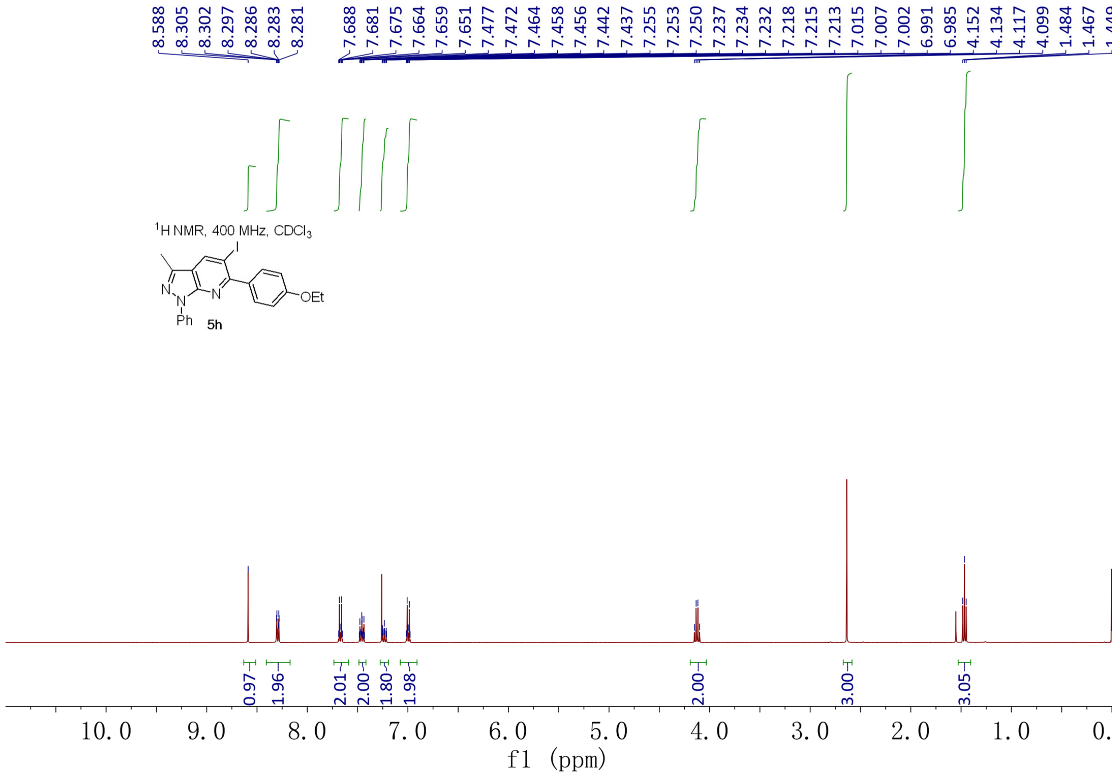
8.588
8.305
8.302
8.297
8.286
8.283
8.281
7.688
7.681
7.675
7.664
7.659
7.651
7.477
7.472
7.464
7.458
7.456
7.442
7.437
7.255
7.253
7.250
7.237
7.234
7.232
7.218
7.215
7.213
7.015
7.007
7.002
6.991
6.985
4.152
4.134
4.117
4.099
1.484
1.467
1H NMR, 400 MHz, CDCl3
I
N
N
N
Ph
OEt
5h
0.97
1.96
2.01
2.00
1.80
1.98
2.00
3.00
3.05
10.0
9.0
8.0
7.0
6.0
5.0
4.0
3.0
2.0
1.0
f1 (ppm)

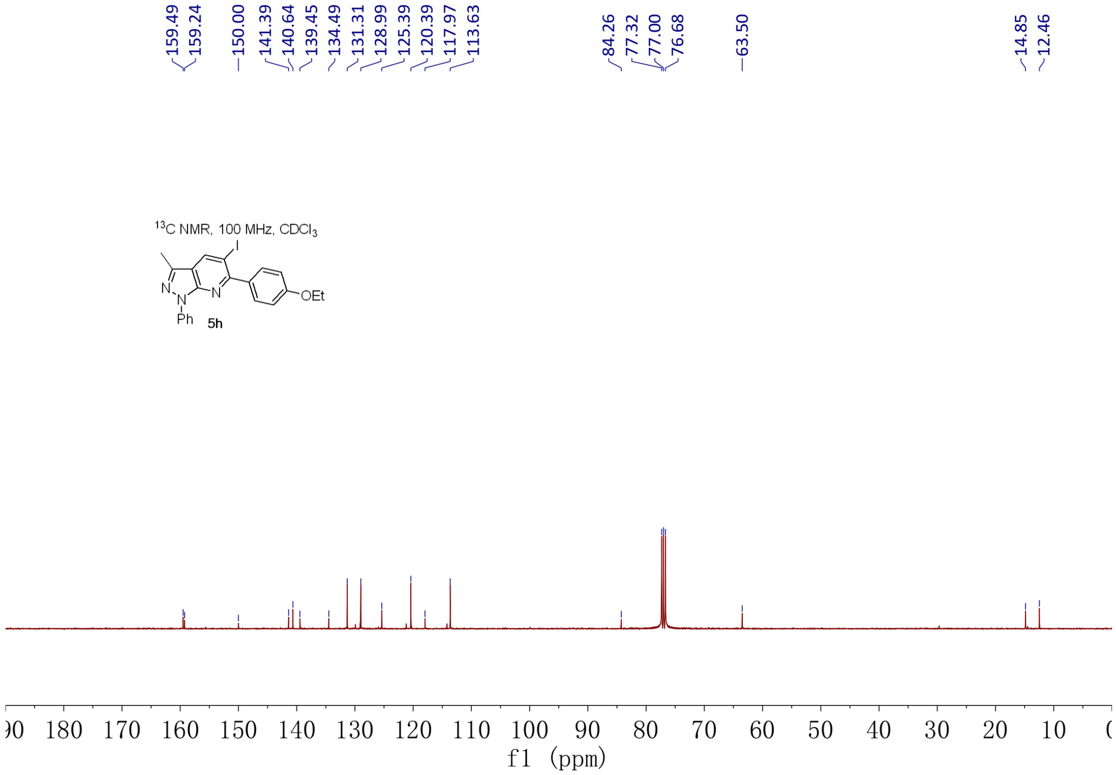
159.49
159.24
150.00
141.39
140.64
139.45
134.49
131.31
128.99
125.39
120.39
117.97
113.63
84.26
77.32
77.00
76.68
63.50
14.85
12.46
13C NMR, 100 MHz, CDCl3
I
N
N
N
Ph
OEt
5h
180
170
160
150
140
130
120
110
100
90
80
70
60
50
40
30
20
10
f1 (ppm)

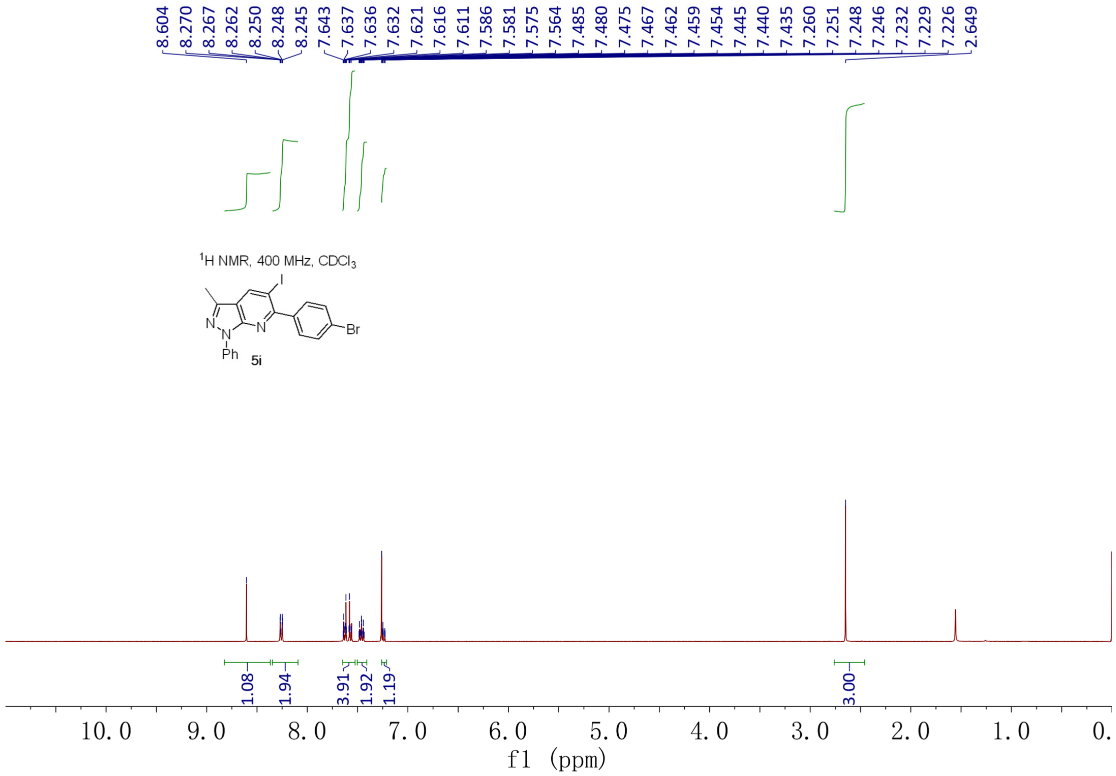
8.604
8.270
8.267
8.262
8.250
8.248
8.245
7.643
7.637
7.636
7.632
7.621
7.616
7.611
7.586
7.581
7.575
7.564
7.485
7.480
7.475
7.467
7.462
7.459
7.454
7.445
7.440
7.435
7.260
7.251
7.248
7.246
7.232
7.229
7.226
2.649
1H NMR, 400 MHz, CDCl3
I
N
N
N
Ph
Br
5i
1.08
1.94
3.91
1.92
1.19
3.00
10.0
9.0
8.0
7.0
6.0
5.0
4.0
3.0
2.0
1.0
f1 (ppm)

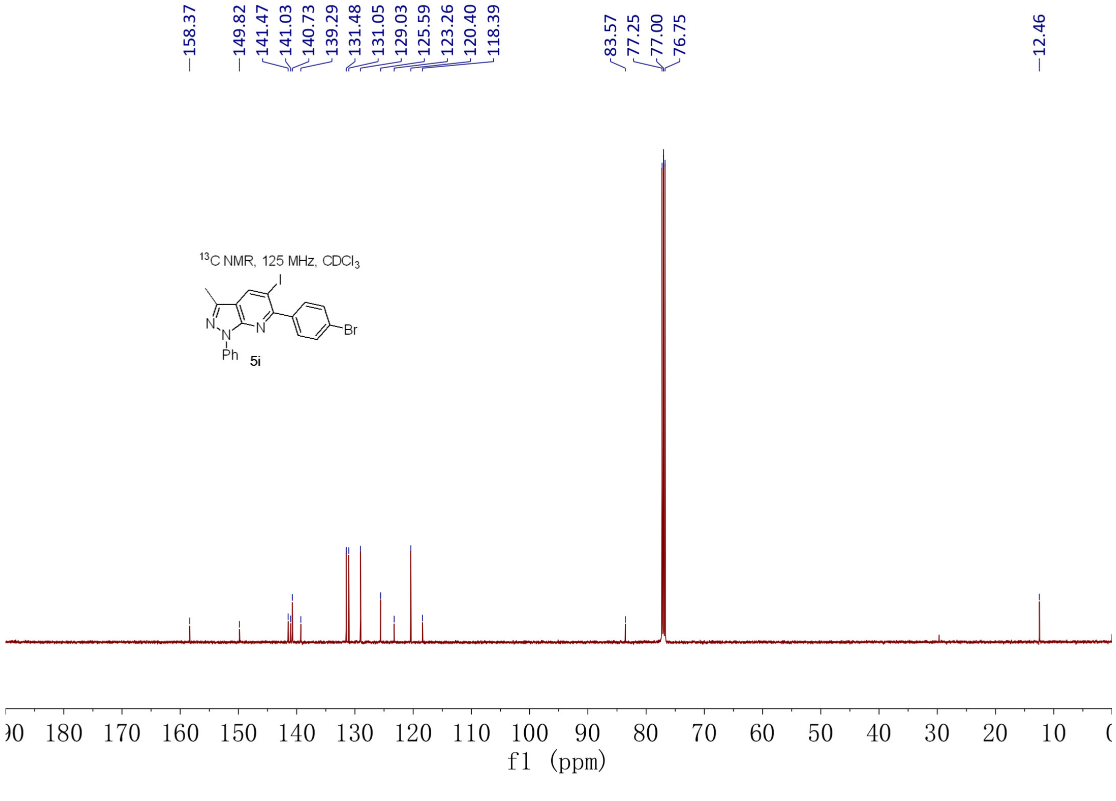
158.37
149.82
141.47
141.03
140.73
139.29
131.48
131.05
129.03
125.59
123.26
120.40
118.39
83.57
77.25
77.00
76.75
12.46
13C NMR, 125 MHz, CDCl3
I
N
N
N
Ph
Br
5i
180
170
160
150
140
130
120
110
100
90
80
70
60
50
40
30
20
10
f1 (ppm)

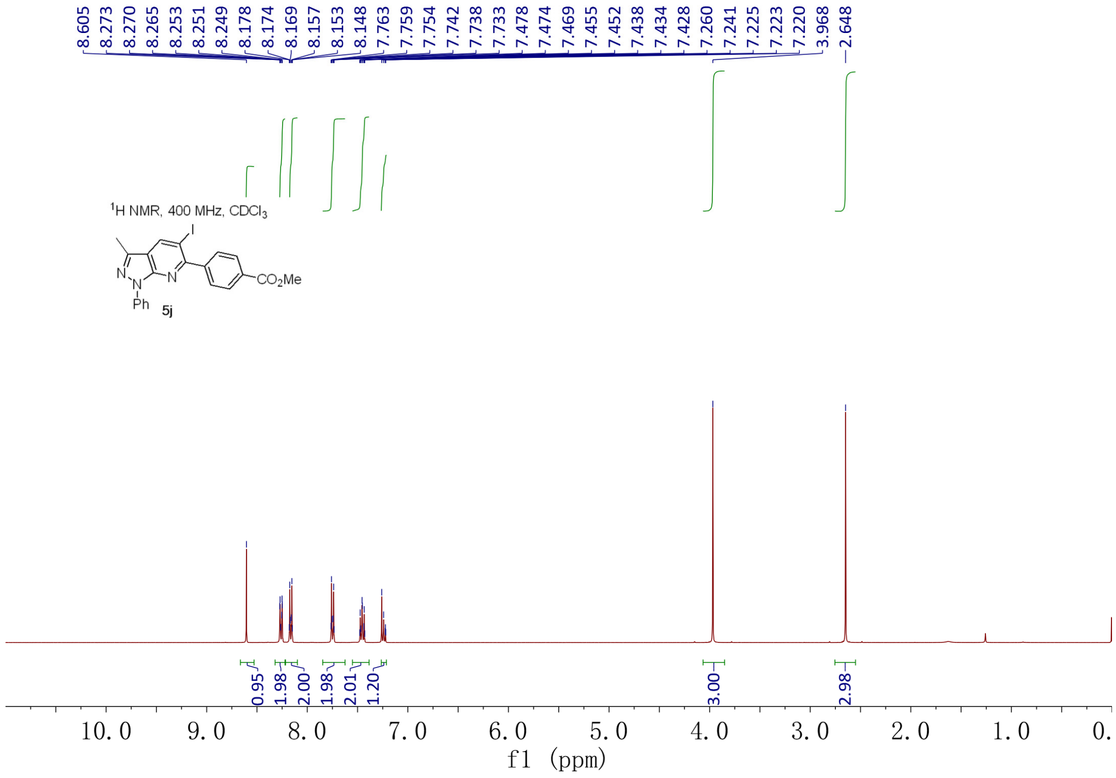
8.605 8.273 8.270 8.265 8.253 8.251 8.249 8.178 8.174 8.169 8.157 8.153 8.148 7.763 7.759 7.754 7.742 7.738 7.733 7.478 7.474 7.469 7.455 7.452 7.438 7.434 7.428 7.260 7.241 7.225 7.223 7.220 3.968 2.648
¹H NMR, 400 MHz, CDCl3
CO2Me
Ph
5j
0.95 1.98 2.00 1.98 2.01 1.20 3.00 2.98
10.0 9.0 8.0 7.0 6.0 5.0 4.0 3.0 2.0 1.0 0.
f1 (ppm)

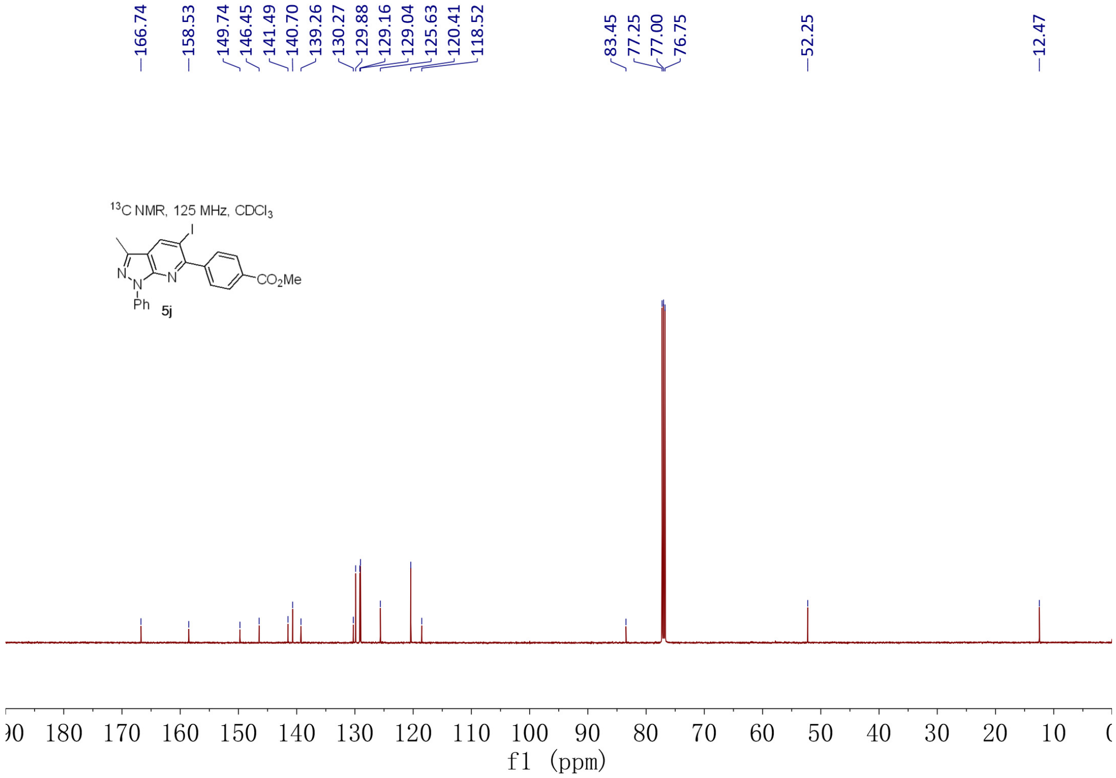
166.74 158.53 149.74 146.45 141.49 140.70 139.26 130.27 129.88 129.16 129.04 125.63 120.41 118.52 83.45 77.25 77.00 76.75 52.25 12.47
¹³C NMR, 125 MHz, CDCl3
CO2Me
Ph
5j
180 170 160 150 140 130 120 110 100 90 80 70 60 50 40 30 20 10
f1 (ppm)

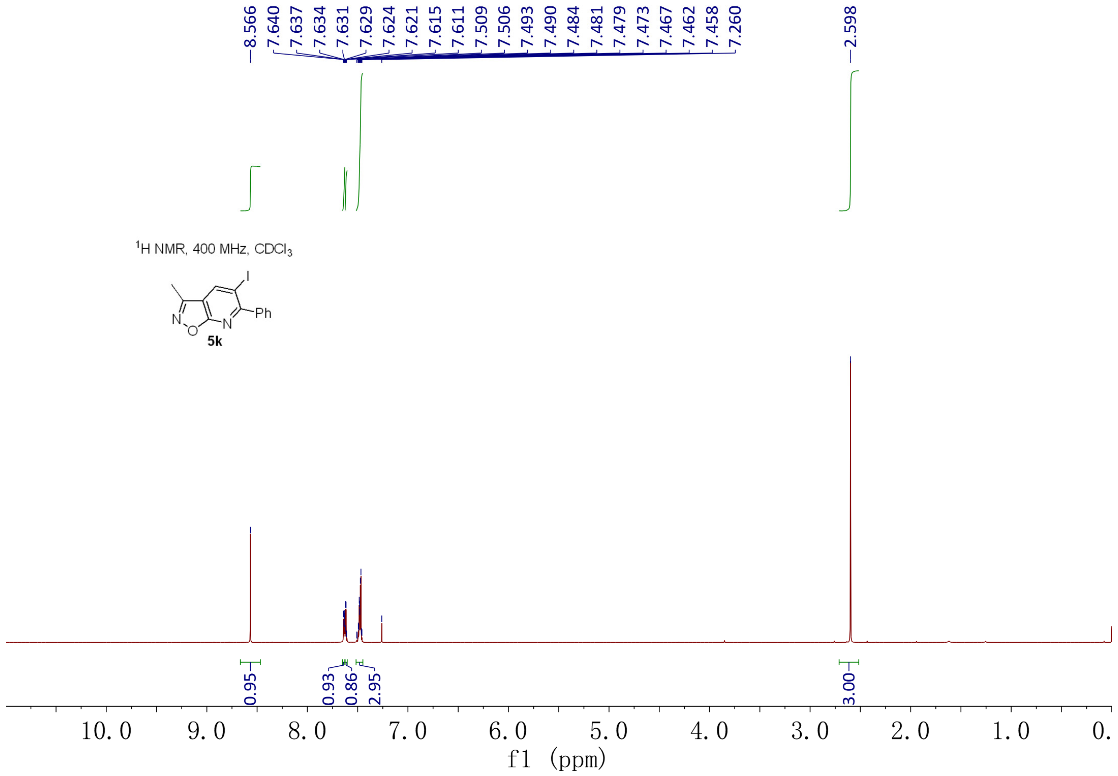
8.566
7.640
7.637
7.634
7.631
7.629
7.624
7.621
7.615
7.611
7.509
7.506
7.493
7.490
7.484
7.481
7.479
7.473
7.467
7.462
7.458
7.260
2.598
^{1}H NMR, 400 MHz, $CDCl_3$
I
Ph
N
O
N
5k
0.95
0.93
0.86
2.95
3.00
10.0
9.0
8.0
7.0
6.0
5.0
4.0
3.0
2.0
1.0
f1 (ppm)

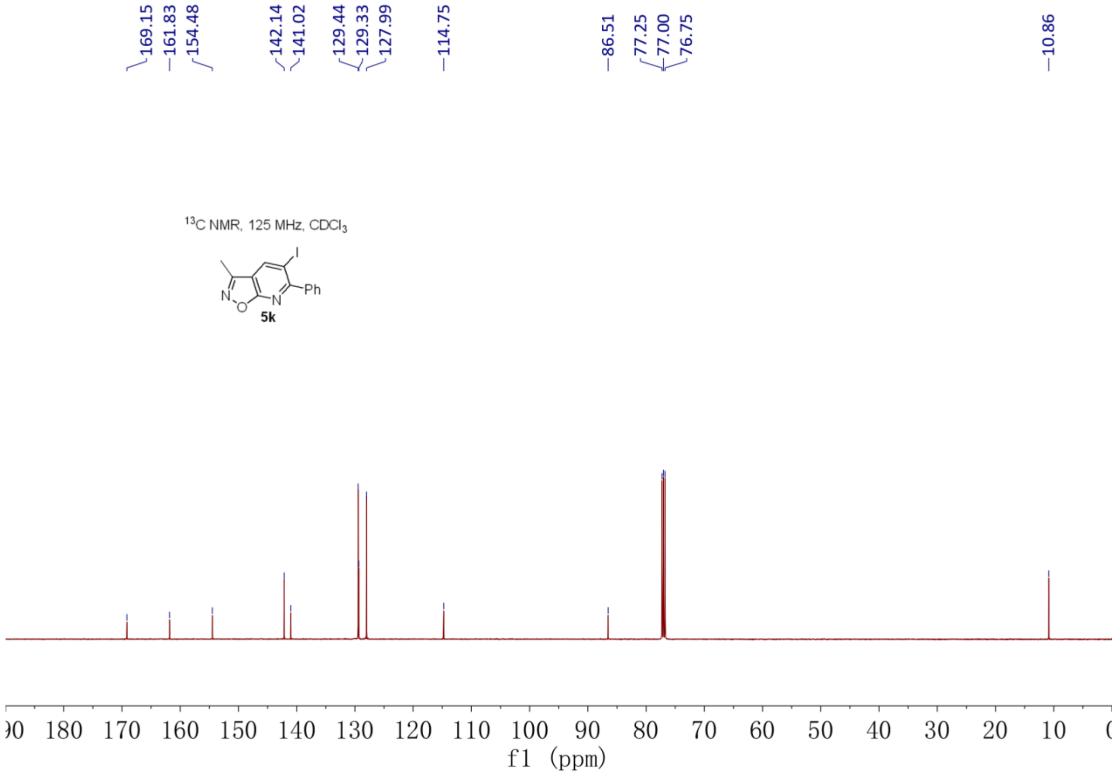
169.15
161.83
154.48
142.14
141.02
129.44
129.33
127.99
114.75
86.51
77.25
77.00
76.75
10.86
^{13}C NMR, 125 MHz, $CDCl_3$
I
Ph
N
O
N
5k
180
170
160
150
140
130
120
110
100
90
80
70
60
50
40
30
20
10
f1 (ppm)

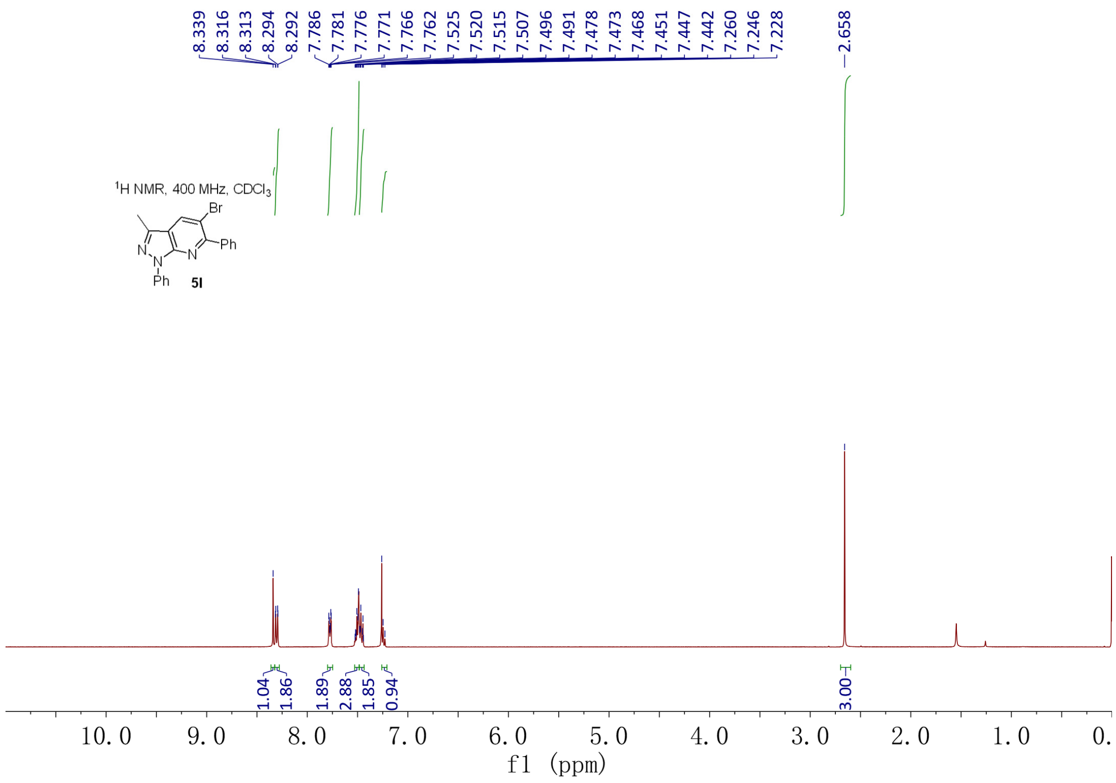

8.339
8.316
8.313
8.294
8.292
7.786
7.781
7.776
7.771
7.766
7.762
7.525
7.520
7.515
7.507
7.496
7.491
7.478
7.473
7.468
7.451
7.447
7.442
7.260
7.246
7.228
2.658
^{1}H NMR, 400 MHz, $CDCl_3$
Br
Ph
N
N
N
Ph
5l
1.04
1.86
1.89
2.88
1.85
0.94
3.00
10.0
9.0
8.0
7.0
6.0
5.0
4.0
3.0
2.0
1.0
0.
f1 (ppm)

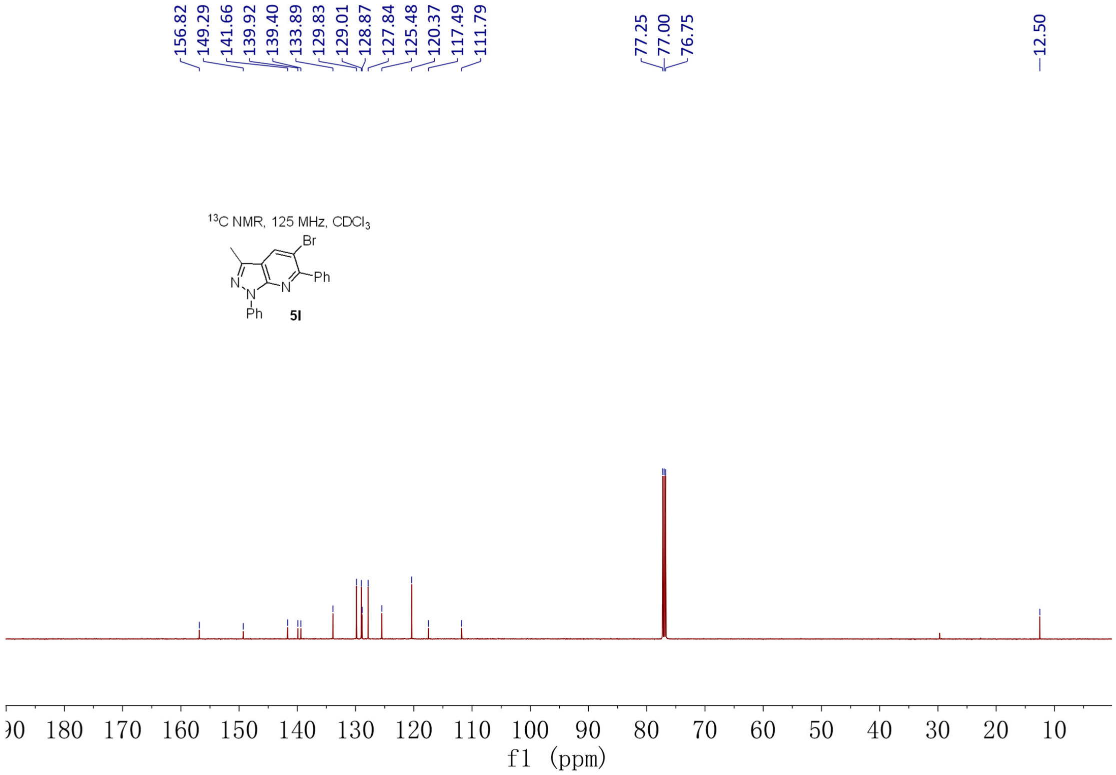

156.82
149.29
141.66
139.92
139.40
133.89
129.83
129.01
128.87
127.84
125.48
120.37
117.49
111.79
77.25
77.00
76.75
12.50
^{13}C NMR, 125 MHz, $CDCl_3$
Br
Ph
N
N
N
Ph
5l
180
170
160
150
140
130
120
110
100
90
80
70
60
50
40
30
20
10
f1 (ppm)

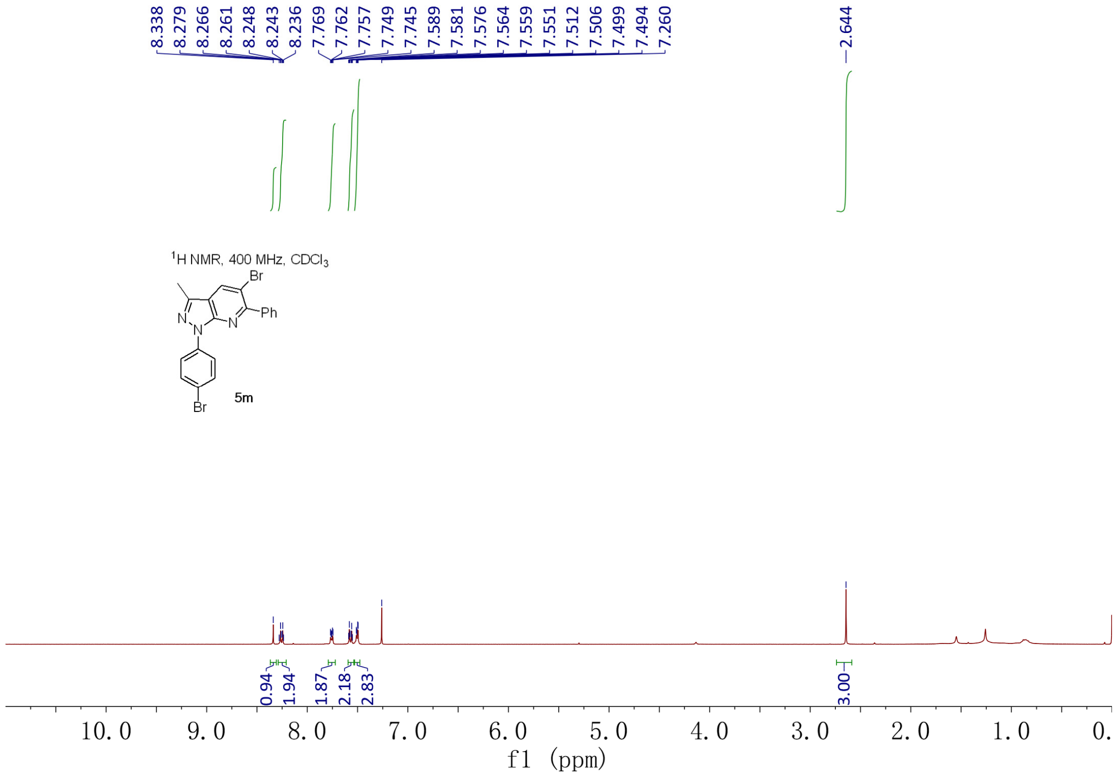
8.338
8.279
8.266
8.261
8.248
8.243
8.236
7.769
7.762
7.757
7.749
7.745
7.589
7.581
7.576
7.564
7.559
7.551
7.512
7.506
7.499
7.494
7.260
2.644
1H NMR, 400 MHz, CDCl3
Br
Ph
N
N
N
Br
5m
0.94
1.94
1.87
2.18
2.83
3.00
10.0
9.0
8.0
7.0
6.0
5.0
4.0
3.0
2.0
1.0
f1 (ppm)

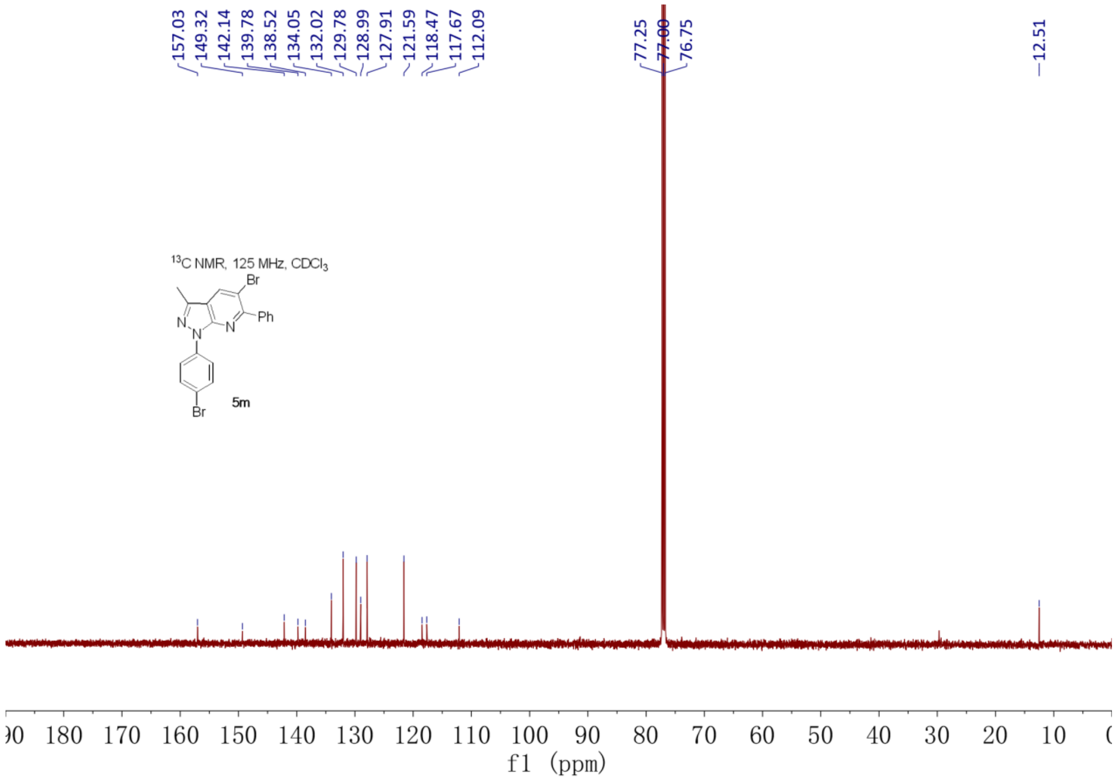
157.03
149.32
142.14
139.78
138.52
134.05
132.02
129.78
128.99
127.91
121.59
118.47
117.67
112.09
77.25
77.00
76.75
12.51
13C NMR, 125 MHz, CDCl3
Br
Ph
N
N
N
Br
5m
180
170
160
150
140
130
120
110
100
90
80
70
60
50
40
30
20
10
f1 (ppm)

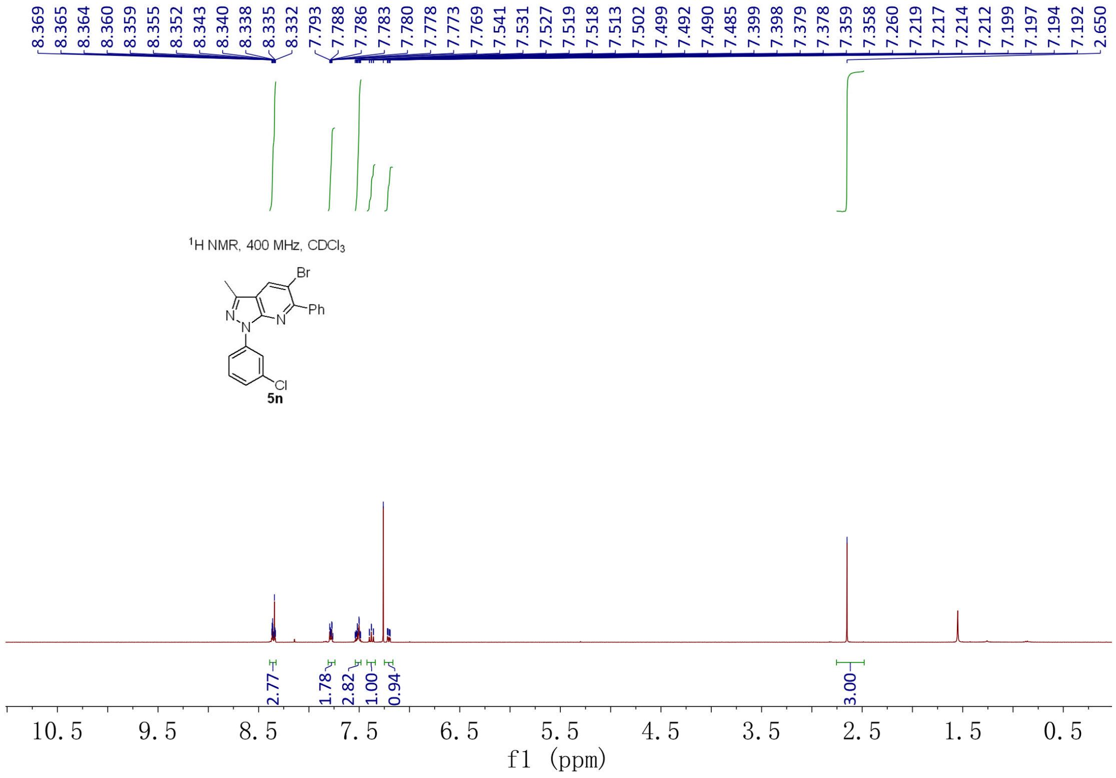
8.369
8.365
8.364
8.360
8.359
8.355
8.352
8.343
8.340
8.338
8.335
8.332
7.793
7.788
7.786
7.783
7.780
7.778
7.773
7.769
7.541
7.531
7.527
7.519
7.518
7.513
7.502
7.499
7.492
7.490
7.485
7.399
7.398
7.379
7.378
7.359
7.358
7.260
7.219
7.217
7.214
7.212
7.199
7.197
7.194
7.192
2.650
1H NMR, 400 MHz, CDCl3
Br
Ph
N
N
N
Cl
5n
2.77
1.78
2.82
1.00
0.94
3.00
10.5
9.5
8.5
7.5
6.5
5.5
4.5
3.5
2.5
1.5
0.5
f1 (ppm)

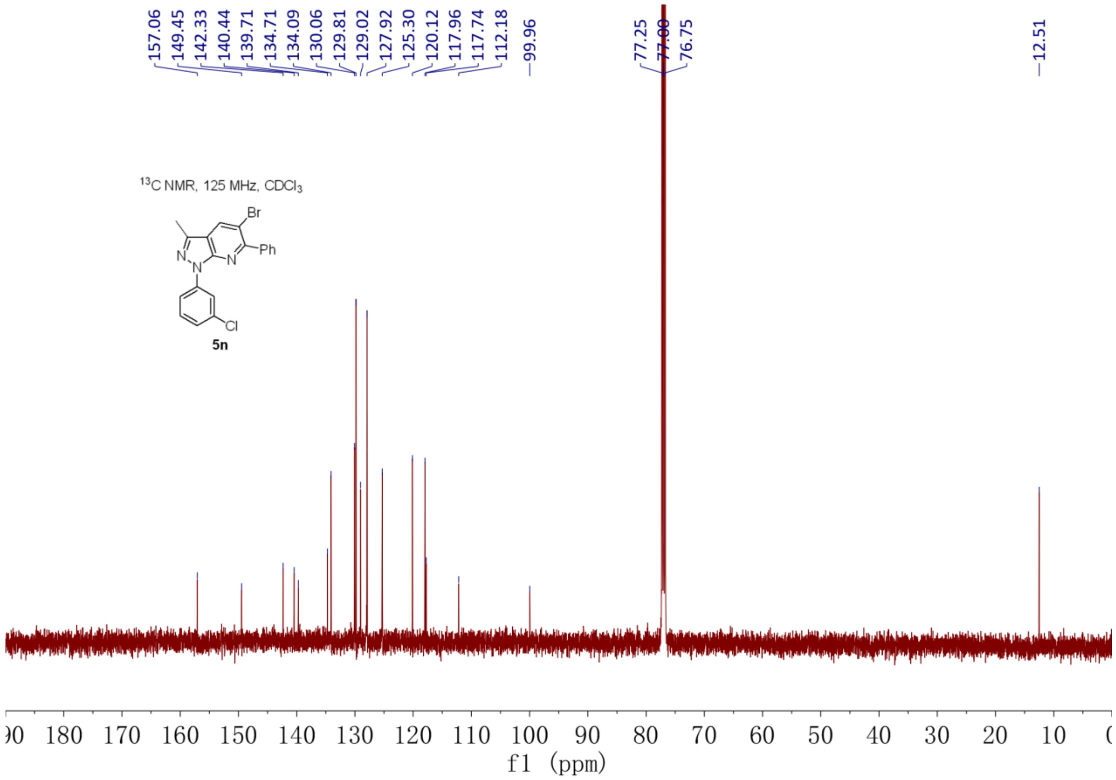
157.06
149.45
142.33
140.44
139.71
134.71
134.09
130.06
129.81
129.02
127.92
125.30
120.12
117.96
117.74
112.18
99.96
77.25
77.00
76.75
12.51
13C NMR, 125 MHz, CDCl3
Br
Ph
N
N
N
Cl
5n
180
170
160
150
140
130
120
110
100
90
80
70
60
50
40
30
20
10
f1 (ppm)

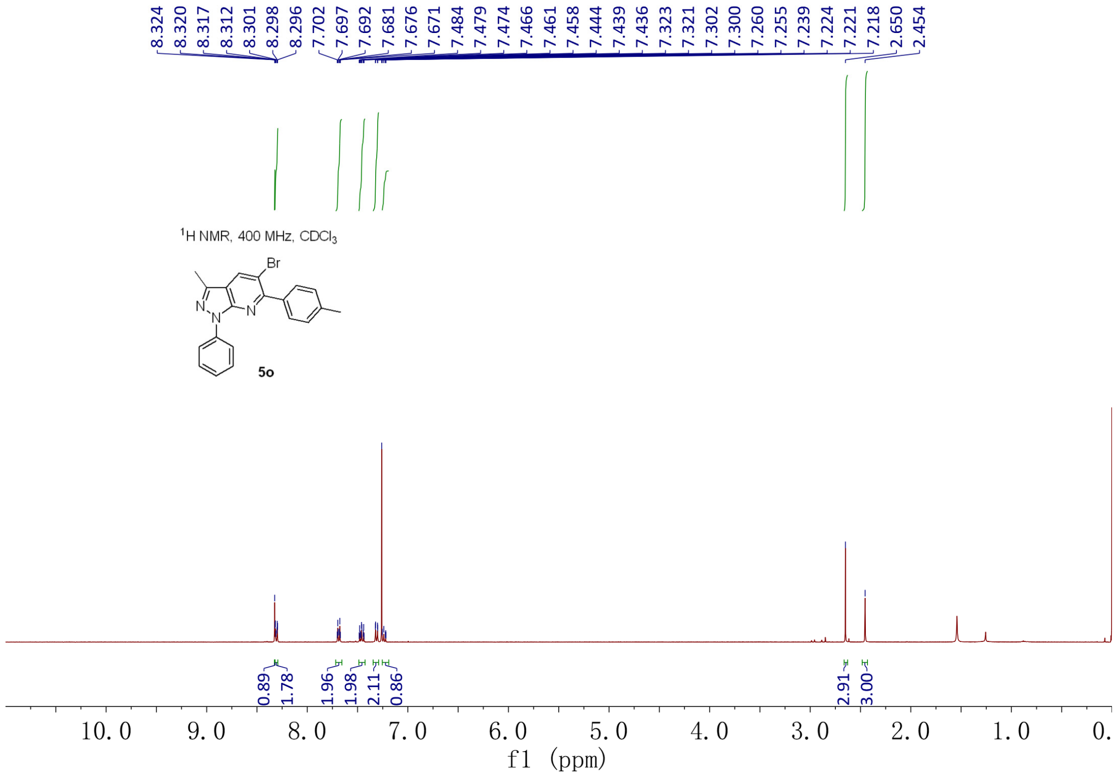
8.324
8.320
8.317
8.312
8.301
8.298
8.296
7.702
7.697
7.692
7.681
7.676
7.671
7.484
7.479
7.474
7.466
7.461
7.458
7.444
7.439
7.436
7.323
7.321
7.302
7.300
7.260
7.255
7.239
7.224
7.221
7.218
2.650
2.454
^{1}H NMR, 400 MHz, CDCl$_3$
Br
N
N
N
5o
0.89
1.78
1.96
1.98
2.11
0.86
2.91
3.00
10.0
9.0
8.0
7.0
6.0
5.0
4.0
3.0
2.0
1.0
f1 (ppm)

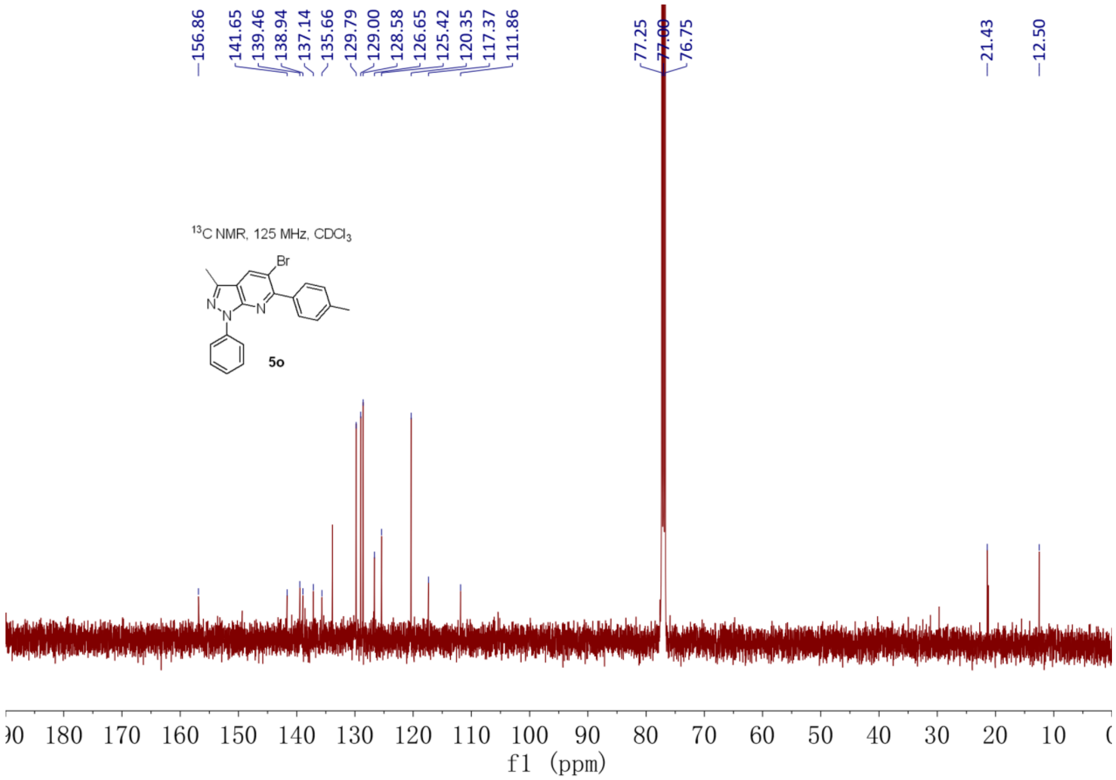
156.86
141.65
139.46
138.94
137.14
135.66
129.79
129.00
128.58
126.65
125.42
120.35
117.37
111.86
77.25
77.00
76.75
21.43
12.50
^{13}C NMR, 125 MHz, CDCl$_3$
Br
N
N
N
5o
180
170
160
150
140
130
120
110
100
90
80
70
60
50
40
30
20
10
f1 (ppm)

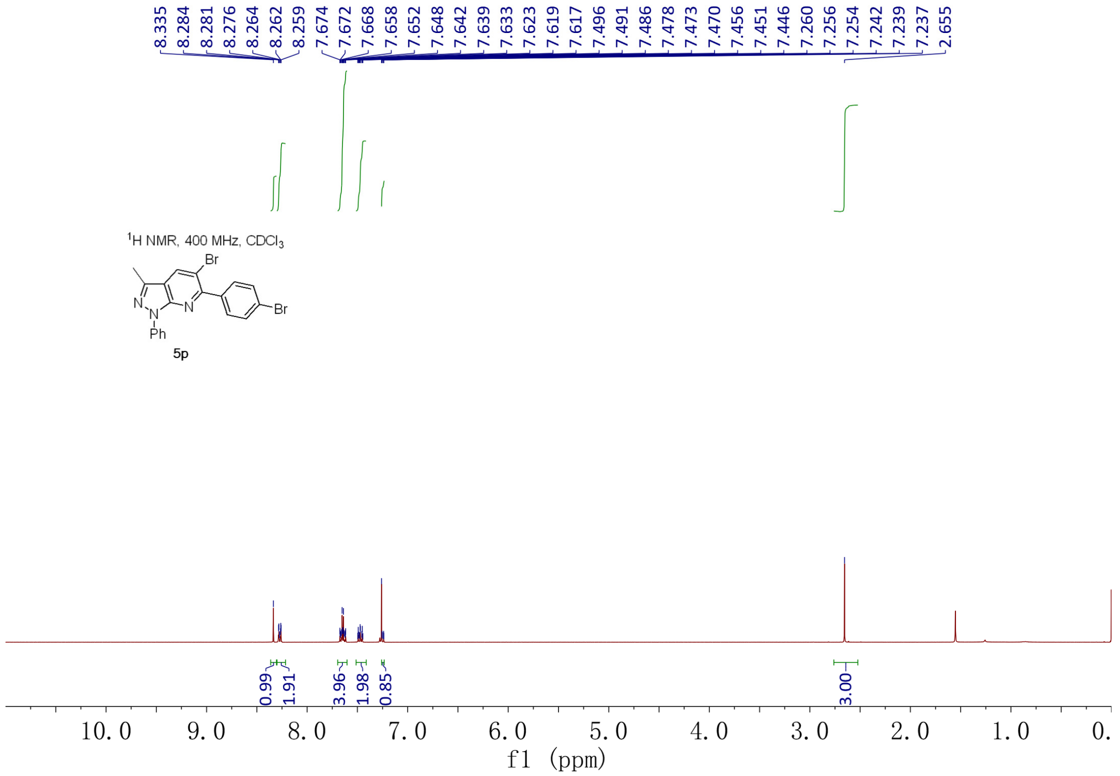
8.335
8.284
8.281
8.276
8.264
8.262
8.259
7.674
7.672
7.668
7.658
7.652
7.648
7.642
7.639
7.633
7.623
7.619
7.617
7.496
7.491
7.486
7.478
7.473
7.470
7.456
7.451
7.446
7.260
7.256
7.254
7.242
7.239
7.237
2.655
1H NMR, 400 MHz, CDCl3
Br
N
N
N
Br
Ph
5p
0.99
1.91
3.96
1.98
0.85
3.00
10.0 9.0 8.0 7.0 6.0 5.0 4.0 3.0 2.0 1.0 0.
f1 (ppm)

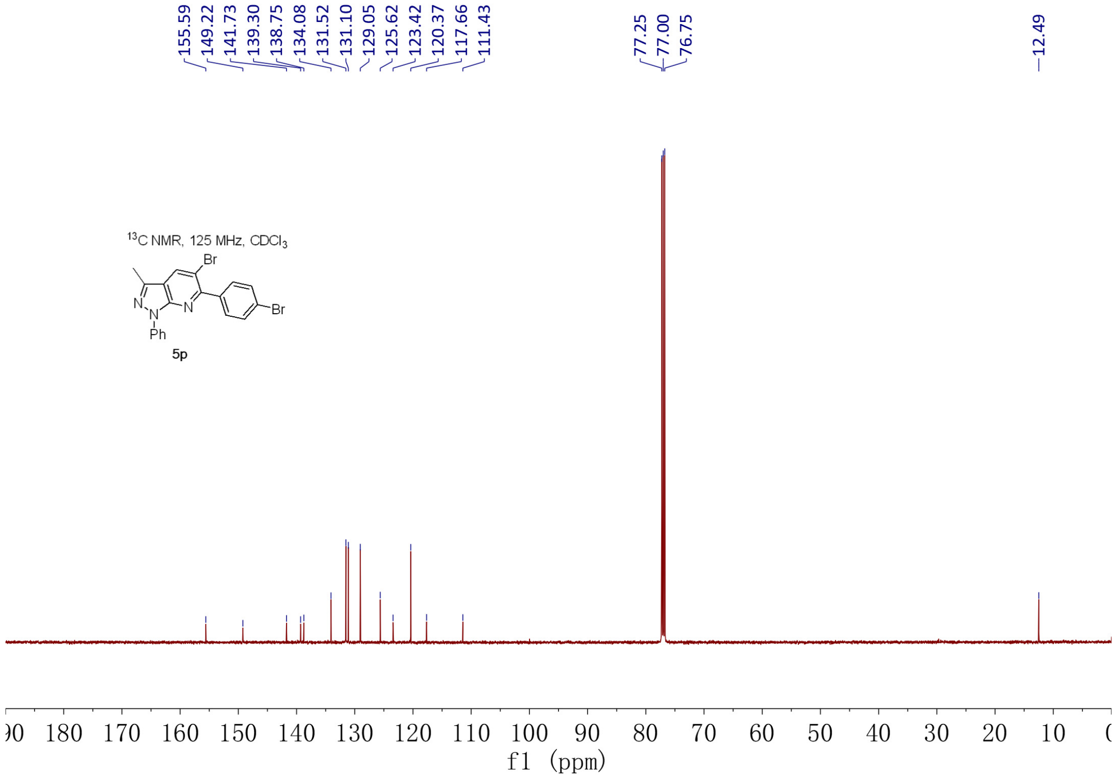
155.59
149.22
141.73
139.30
138.75
134.08
131.52
131.10
129.05
125.62
123.42
120.37
117.66
111.43
77.25
77.00
76.75
12.49
13C NMR, 125 MHz, CDCl3
Br
N
N
N
Br
Ph
5p
180 170 160 150 140 130 120 110 100 90 80 70 60 50 40 30 20 10
f1 (ppm)

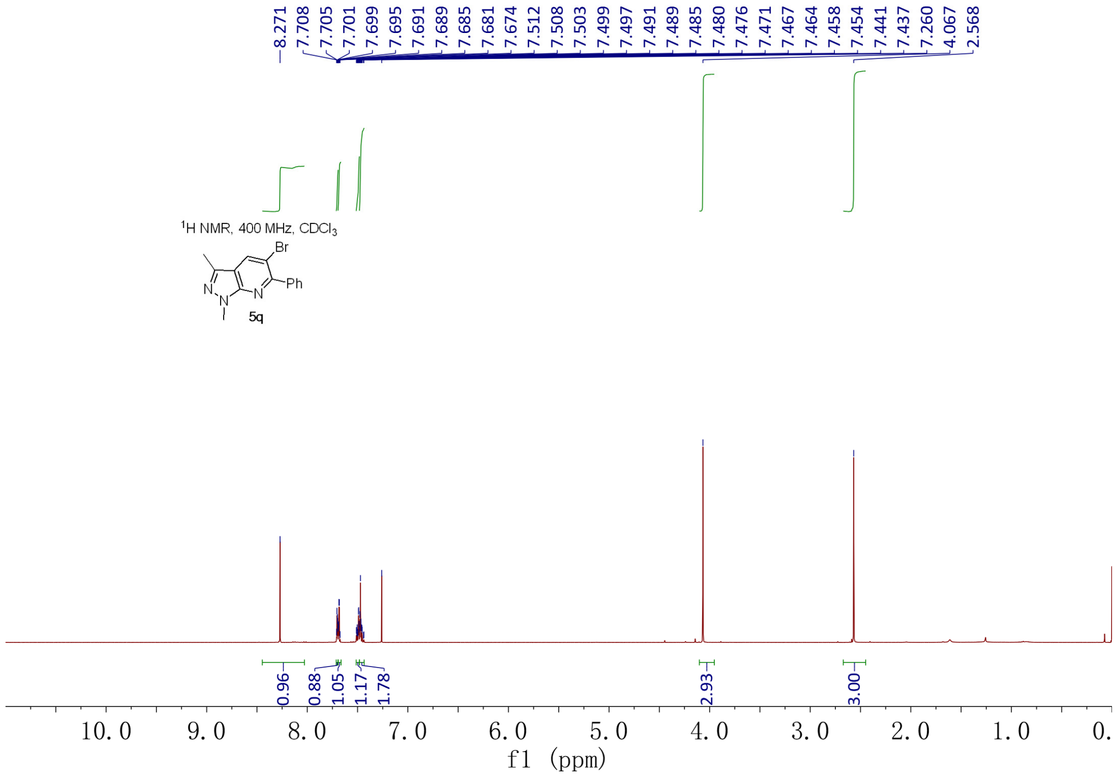

8.271
7.708
7.705
7.701
7.699
7.695
7.691
7.689
7.685
7.681
7.674
7.512
7.508
7.503
7.499
7.497
7.491
7.489
7.485
7.480
7.476
7.471
7.467
7.464
7.458
7.454
7.441
7.437
7.260
4.067
2.568
^{1}H NMR, 400 MHz, CDCl$_3$
Br
Ph
N
N
N
5q
0.96
0.88
1.05
1.17
1.78
2.93
3.00
10.0
9.0
8.0
7.0
6.0
5.0
4.0
3.0
2.0
1.0
0.
f1 (ppm)

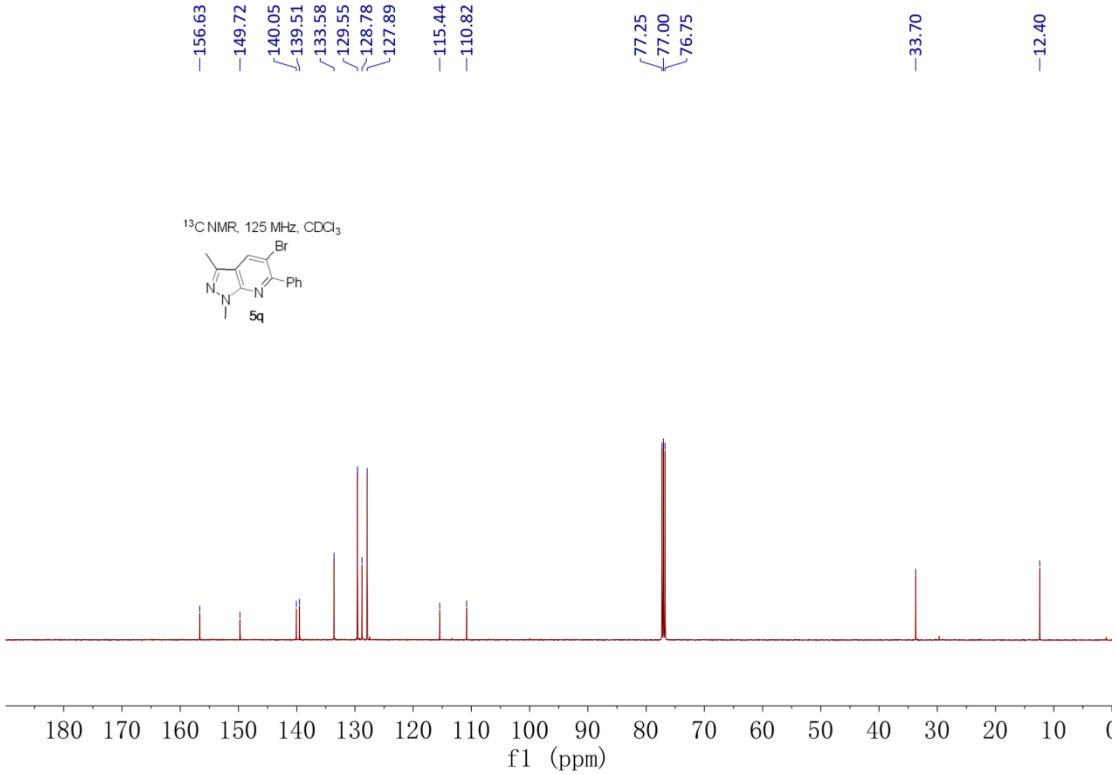

156.63
149.72
140.05
139.51
133.58
129.55
128.78
127.89
115.44
110.82
77.25
77.00
76.75
33.70
12.40
^{13}C NMR, 125 MHz, CDCl$_3$
Br
Ph
N
N
N
5q
180
170
160
150
140
130
120
110
100
90
80
70
60
50
40
30
20
10
f1 (ppm)

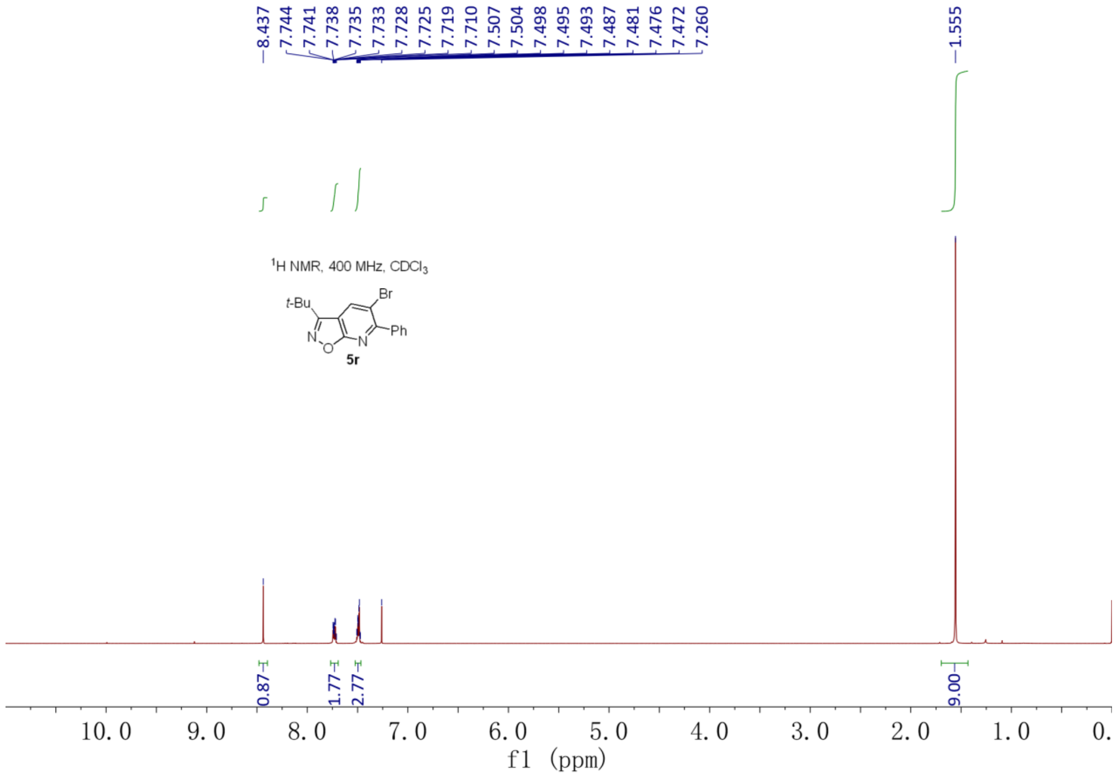
8.437
7.744
7.741
7.738
7.735
7.733
7.728
7.725
7.719
7.710
7.507
7.504
7.498
7.495
7.493
7.487
7.481
7.476
7.472
7.260
1.555
1H NMR, 400 MHz, CDCl3
t-Bu
Br
N
O
N
Ph
5r
0.87
1.77
2.77
9.00
10.0 9.0 8.0 7.0 6.0 5.0 4.0 3.0 2.0 1.0
f1 (ppm)

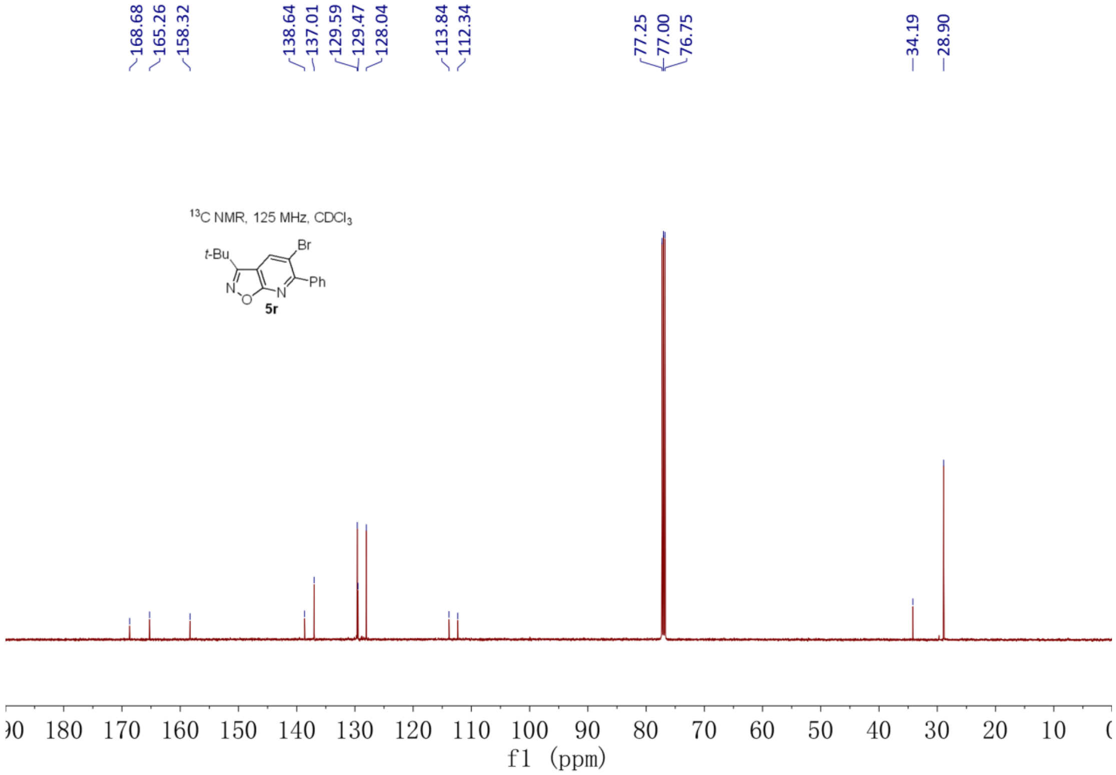
168.68
165.26
158.32
138.64
137.01
129.59
129.47
128.04
113.84
112.34
77.25
77.00
76.75
34.19
28.90
13C NMR, 125 MHz, CDCl3
t-Bu
Br
N
O
N
Ph
5r
180 170 160 150 140 130 120 110 100 90 80 70 60 50 40 30 20 10
f1 (ppm)

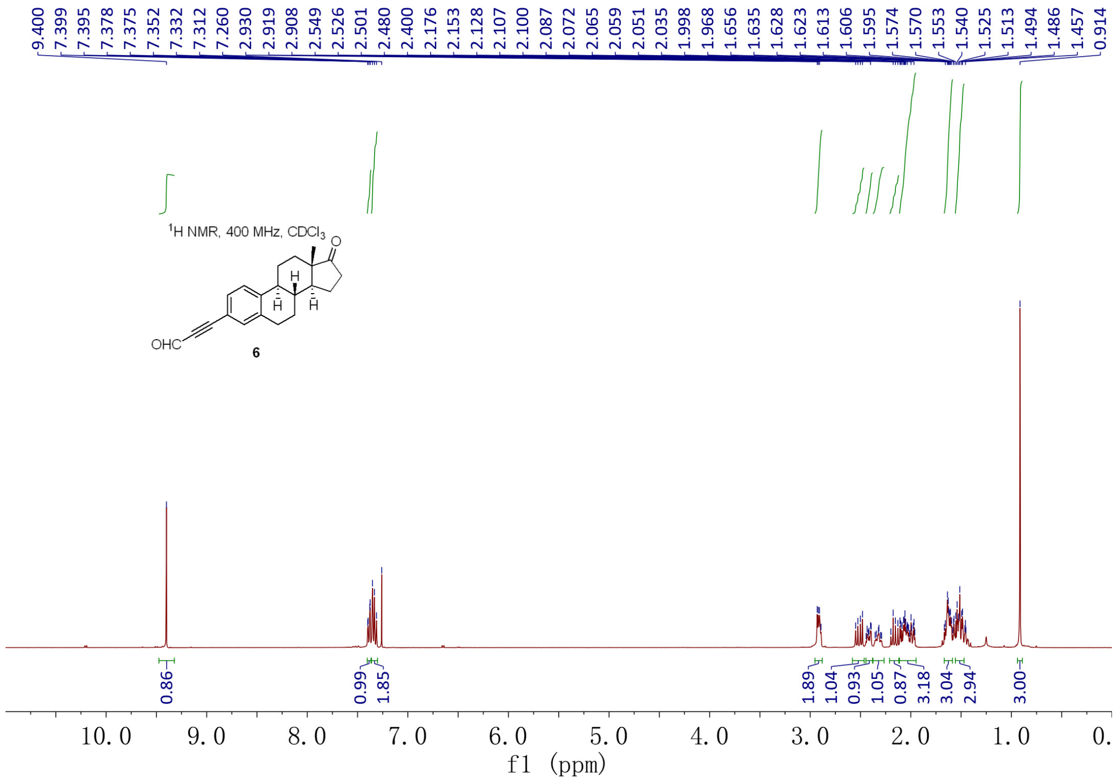
9.400 7.399 7.395 7.378 7.375 7.352 7.332 7.312 7.260 2.930 2.919 2.908 2.549 2.526 2.501 2.480 2.400 2.176 2.153 2.128 2.107 2.100 2.087 2.072 2.065 2.059 2.051 2.035 1.998 1.968 1.656 1.635 1.628 1.623 1.613 1.606 1.595 1.574 1.570 1.553 1.540 1.525 1.513 1.494 1.486 1.457 0.914
1H NMR, 400 MHz, CDCl3
OHC
6
0.86 0.99 1.85 1.89 1.04 0.93 1.05 0.87 3.18 3.04 2.94 3.00
10.0 9.0 8.0 7.0 6.0 5.0 4.0 3.0 2.0 1.0 0.
f1 (ppm)

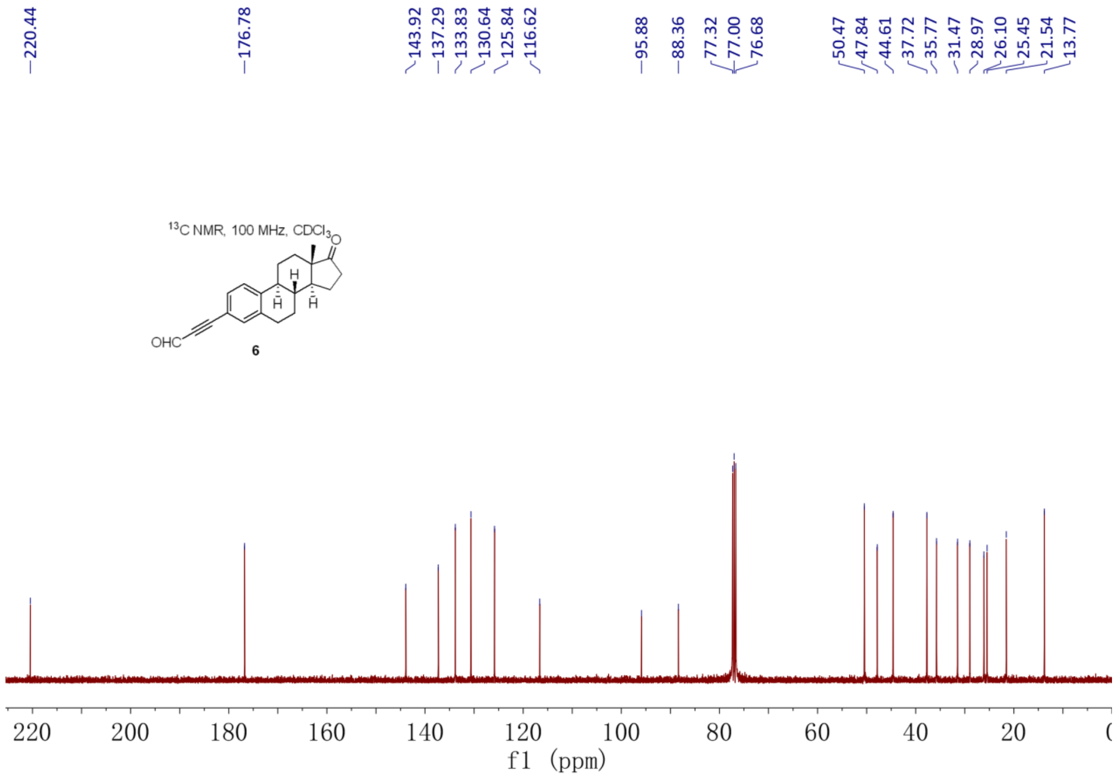
220.44 176.78 143.92 137.29 133.83 130.64 125.84 116.62 95.88 88.36 77.32 77.00 76.68 50.47 47.84 44.61 37.72 35.77 31.47 28.97 26.10 25.45 21.54 13.77
13C NMR, 100 MHz, CDCl3
OHC
6
220 200 180 160 140 120 100 80 60 40 20
f1 (ppm)

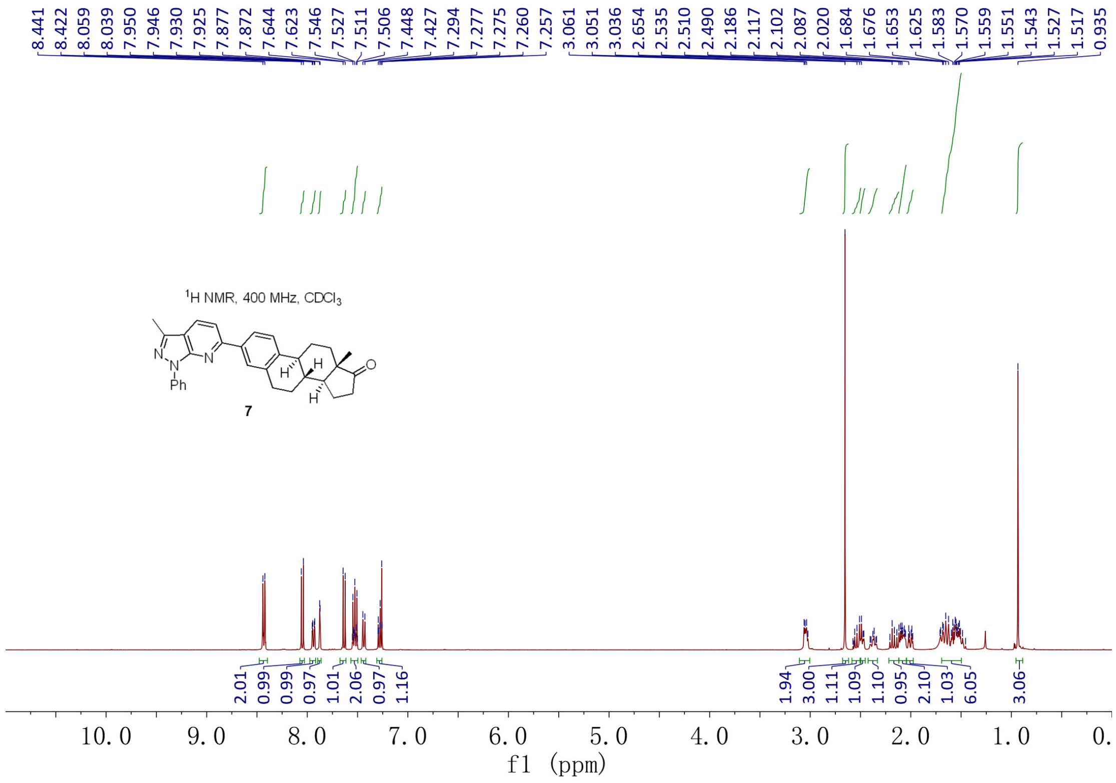
8.441 8.422 8.059 8.039 7.950 7.946 7.930 7.925 7.877 7.872 7.644 7.623 7.546 7.527 7.511 7.506 7.448 7.427 7.294 7.277 7.275 7.260 7.257 3.061 3.051 3.036 2.654 2.535 2.510 2.490 2.186 2.117 2.102 2.087 2.020 1.684 1.676 1.653 1.625 1.583 1.570 1.559 1.551 1.543 1.527 1.517 0.935
1H NMR, 400 MHz, CDCl3
7
2.01 0.99 0.99 0.97 1.01 2.06 0.97 1.16
1.94 3.00 1.11 1.09 1.10 0.95 2.10 1.03 6.05
3.06
f1 (ppm)

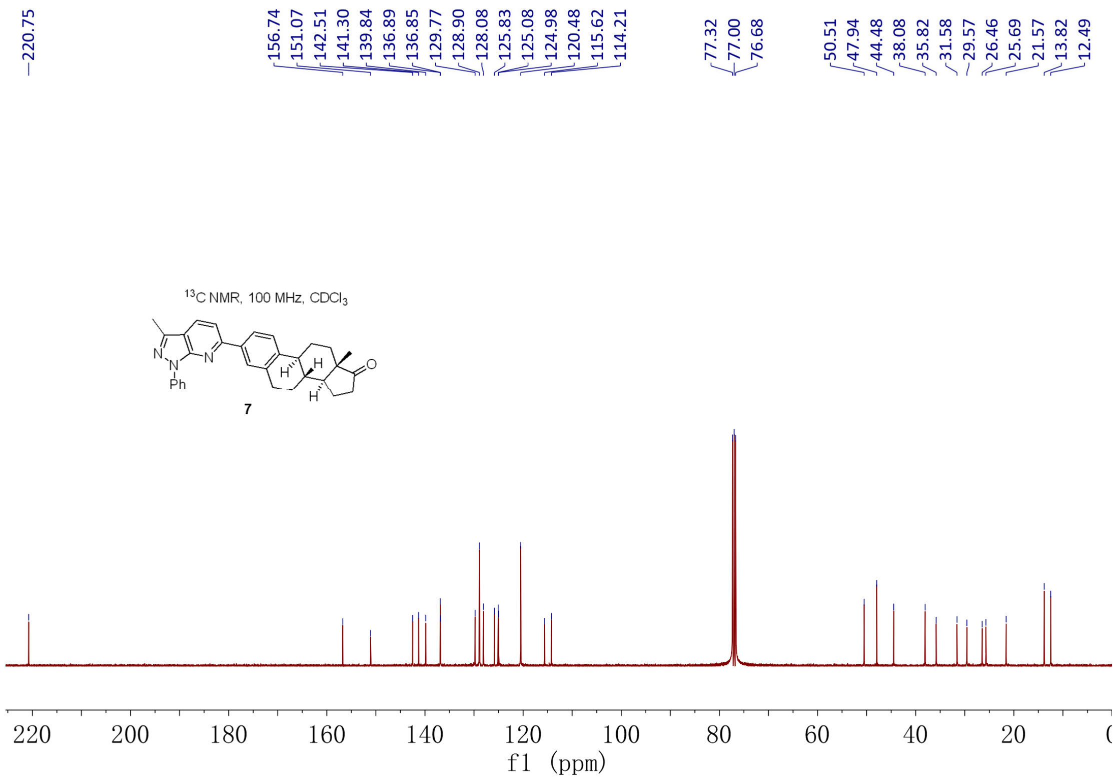
220.75
156.74 151.07 142.51 141.30 139.84 136.89 136.85 129.77 128.90 128.08 125.83 125.08 124.98 120.48 115.62 114.21
77.32 77.00 76.68
50.51 47.94 44.48 38.08 35.82 31.58 29.57 26.46 25.69 21.57 13.82 12.49
13C NMR, 100 MHz, CDCl3
7
f1 (ppm)

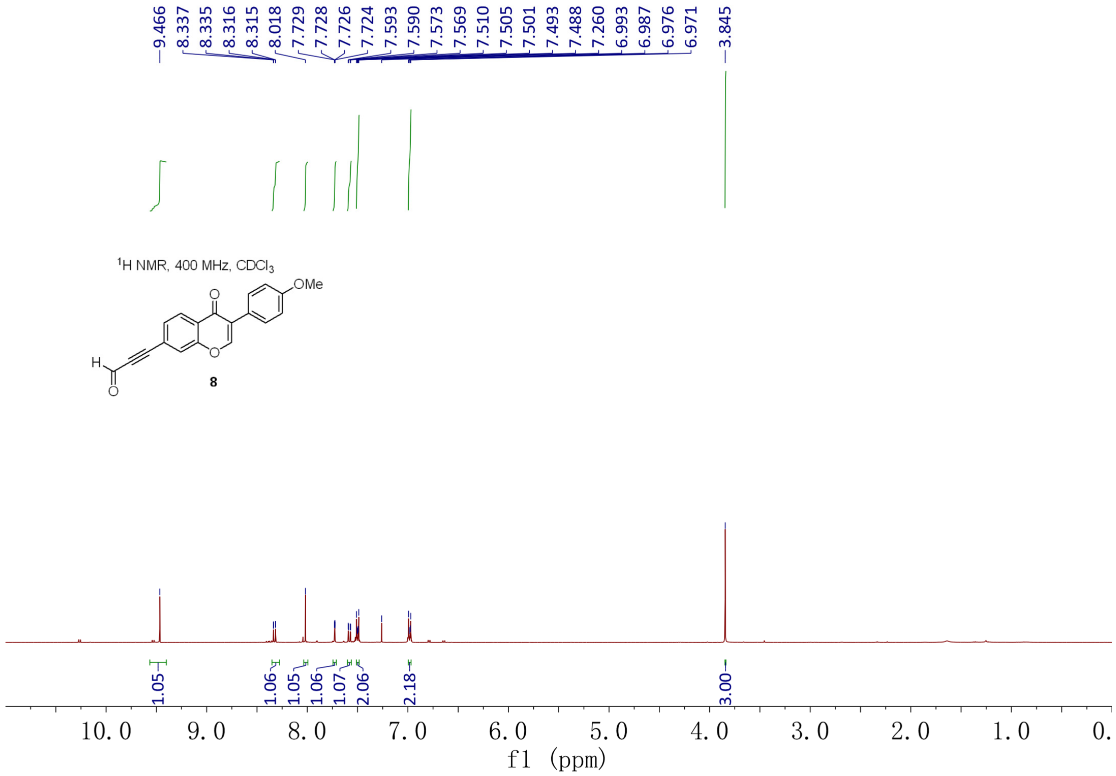
9.466 8.337 8.335 8.316 8.315 8.018 7.729 7.728 7.726 7.724 7.593 7.590 7.573 7.569 7.510 7.505 7.501 7.493 7.488 7.260 6.993 6.987 6.976 6.971 3.845
1H NMR, 400 MHz, CDCl3
OMe
8
1.05 1.06 1.05 1.06 1.07 2.06 2.18 3.00
10.0 9.0 8.0 7.0 6.0 5.0 4.0 3.0 2.0 1.0
f1 (ppm)

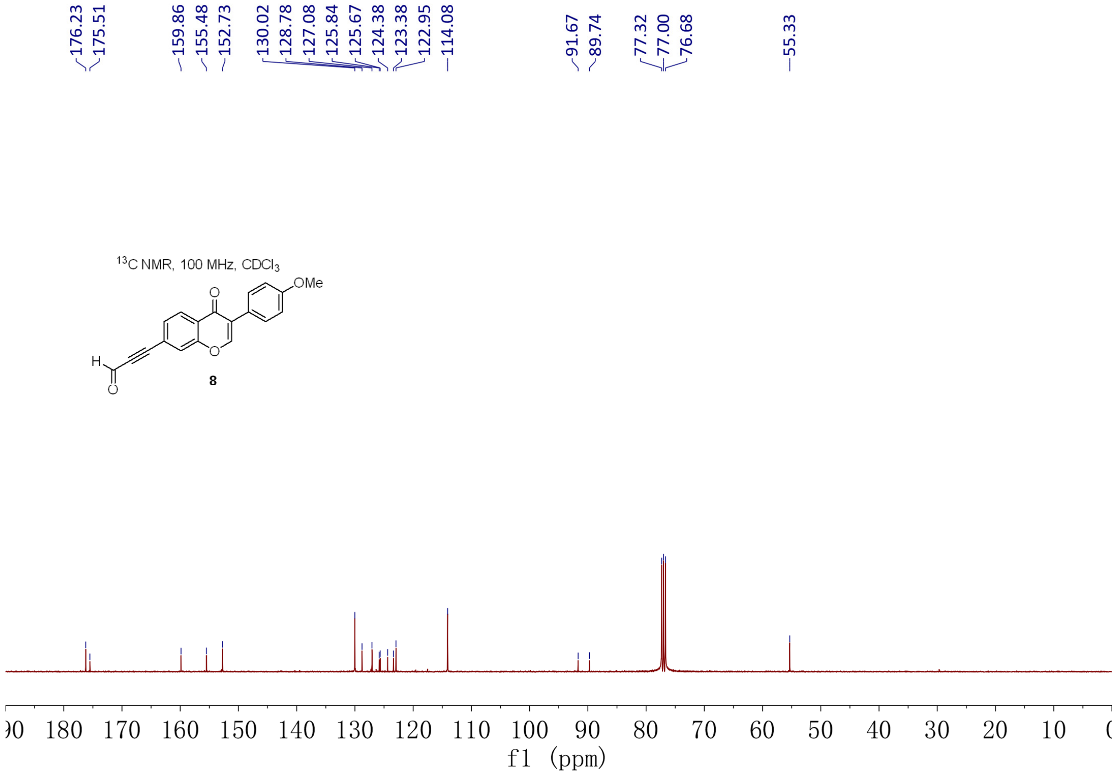
176.23 175.51 159.86 155.48 152.73 130.02 128.78 127.08 125.84 125.67 124.38 123.38 122.95 114.08 91.67 89.74 77.32 77.00 76.68 55.33
13C NMR, 100 MHz, CDCl3
OMe
8
180 170 160 150 140 130 120 110 100 90 80 70 60 50 40 30 20 10
f1 (ppm)

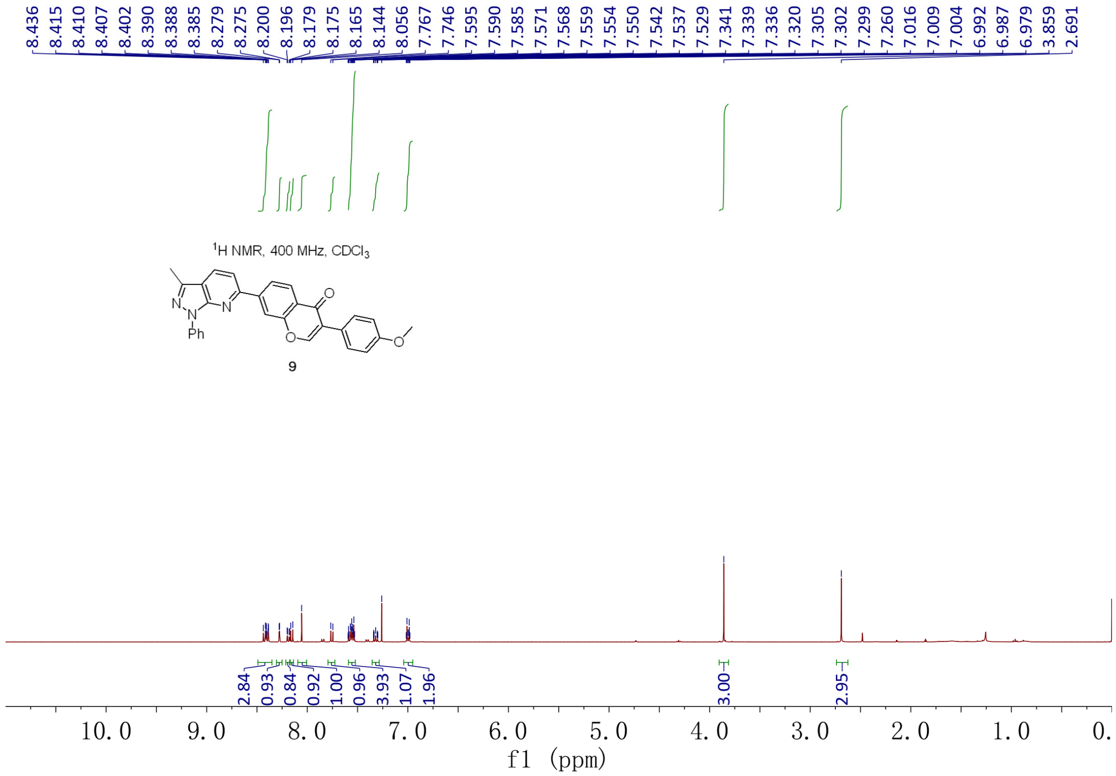
8.436 8.415 8.410 8.407 8.402 8.390 8.388 8.385 8.279 8.275 8.200 8.196 8.179 8.175 8.165 8.144 8.056 7.767 7.746 7.595 7.590 7.585 7.571 7.568 7.559 7.554 7.550 7.542 7.537 7.529 7.341 7.339 7.336 7.320 7.305 7.302 7.299 7.260 7.016 7.009 7.004 6.992 6.987 6.979 3.859 2.691
^{1}H NMR, 400 MHz, $CDCl_3$
N
N
Ph
N
O
O
O
9
2.84 0.93 0.84 0.92 1.00 0.96 3.93 1.07 1.96 3.00 2.95
10.0 9.0 8.0 7.0 6.0 5.0 4.0 3.0 2.0 1.0 0.
f1 (ppm)

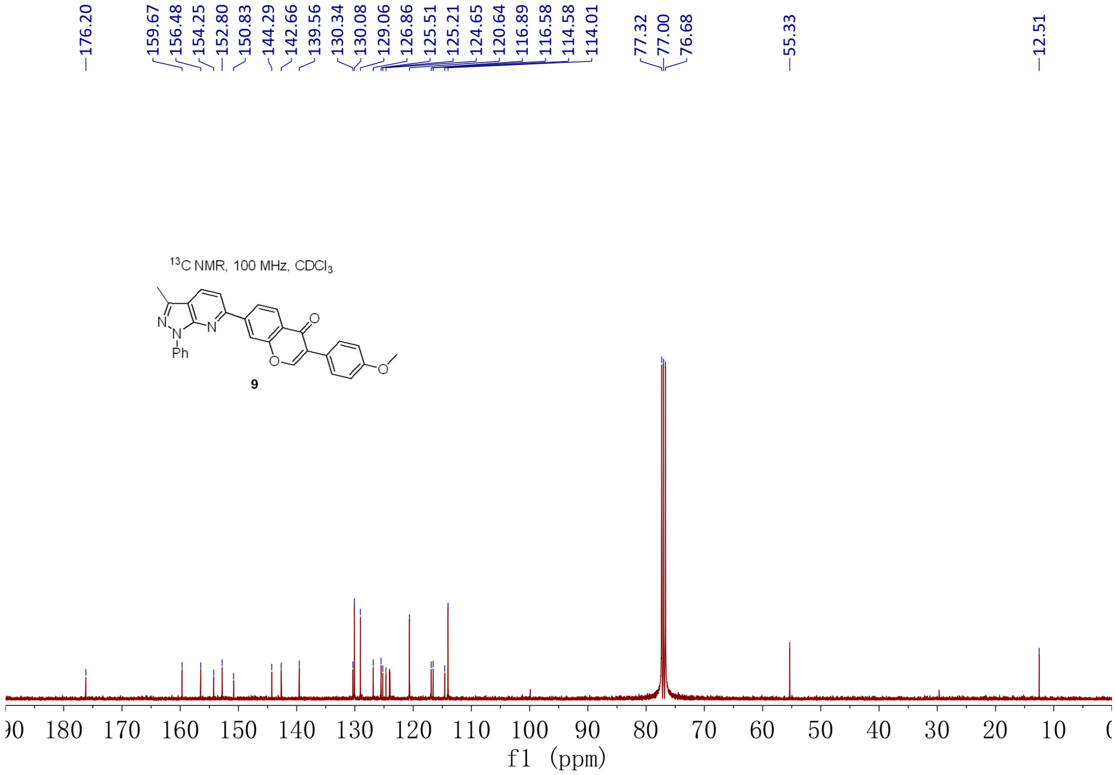
176.20 159.67 156.48 154.25 152.80 150.83 144.29 142.66 139.56 130.34 130.08 129.06 126.86 125.51 125.21 124.65 120.64 116.89 116.58 114.58 114.01 77.32 77.00 76.68 55.33 12.51
^{13}C NMR, 100 MHz, $CDCl_3$
N
N
Ph
N
O
O
O
9
180 170 160 150 140 130 120 110 100 90 80 70 60 50 40 30 20 10
f1 (ppm)

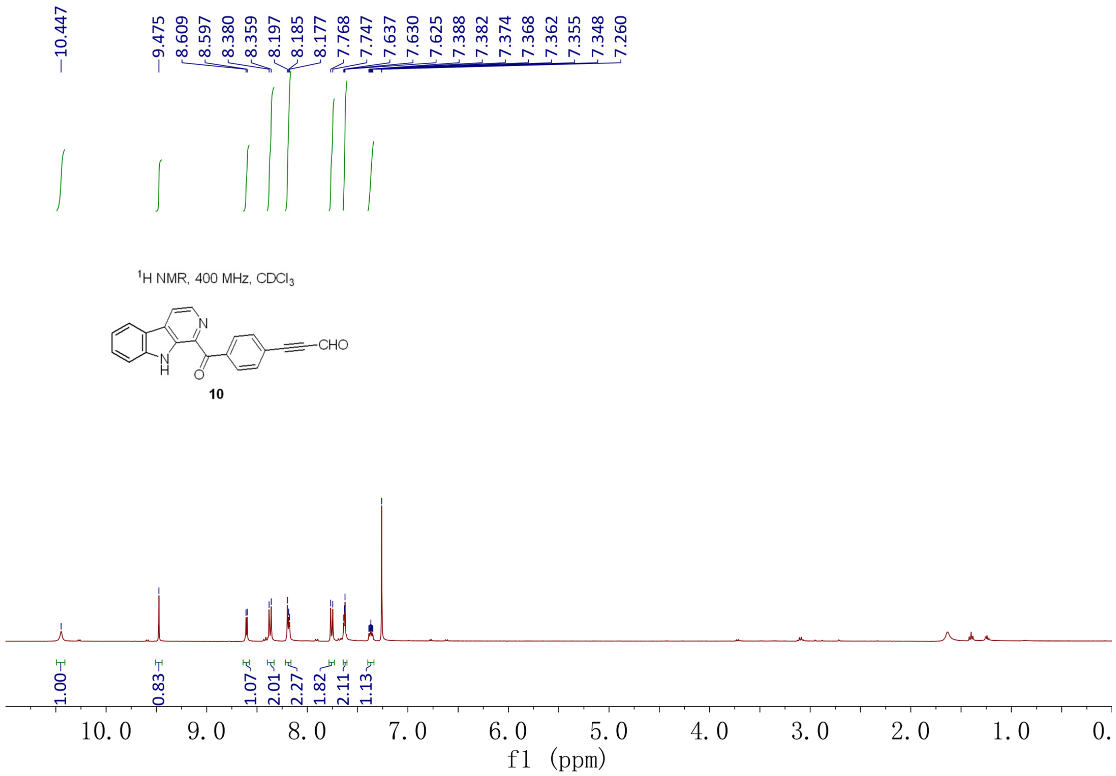
10.447
9.475
8.609
8.597
8.380
8.359
8.197
8.185
8.177
7.768
7.747
7.637
7.630
7.625
7.388
7.382
7.374
7.368
7.362
7.355
7.348
7.260
1H NMR, 400 MHz, CDCl3
CHO
N
N
H
O
10
1.00
0.83
1.07
2.01
2.27
1.82
2.11
1.13
10.0
9.0
8.0
7.0
6.0
5.0
4.0
3.0
2.0
1.0
f1 (ppm)

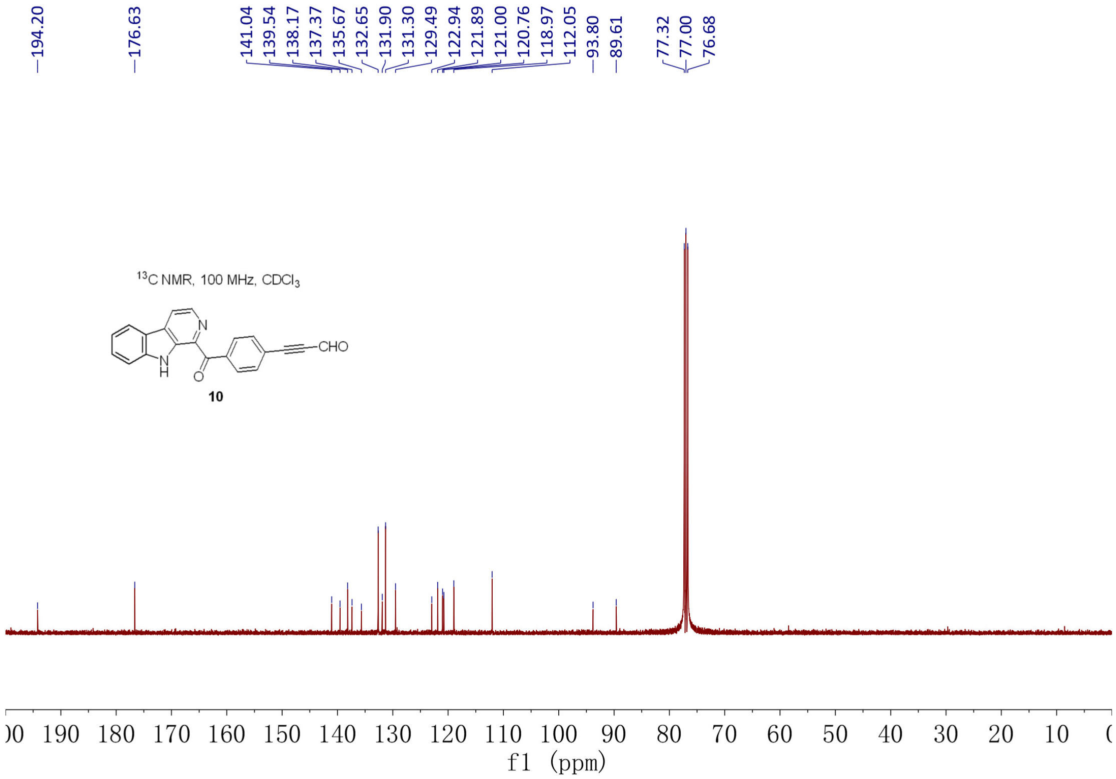
194.20
176.63
141.04
139.54
138.17
137.37
135.67
132.65
131.90
131.30
129.49
122.94
121.89
121.00
120.76
118.97
112.05
93.80
89.61
77.32
77.00
76.68
13C NMR, 100 MHz, CDCl3
CHO
N
N
H
O
10
190
180
170
160
150
140
130
120
110
100
90
80
70
60
50
40
30
20
10
f1 (ppm)

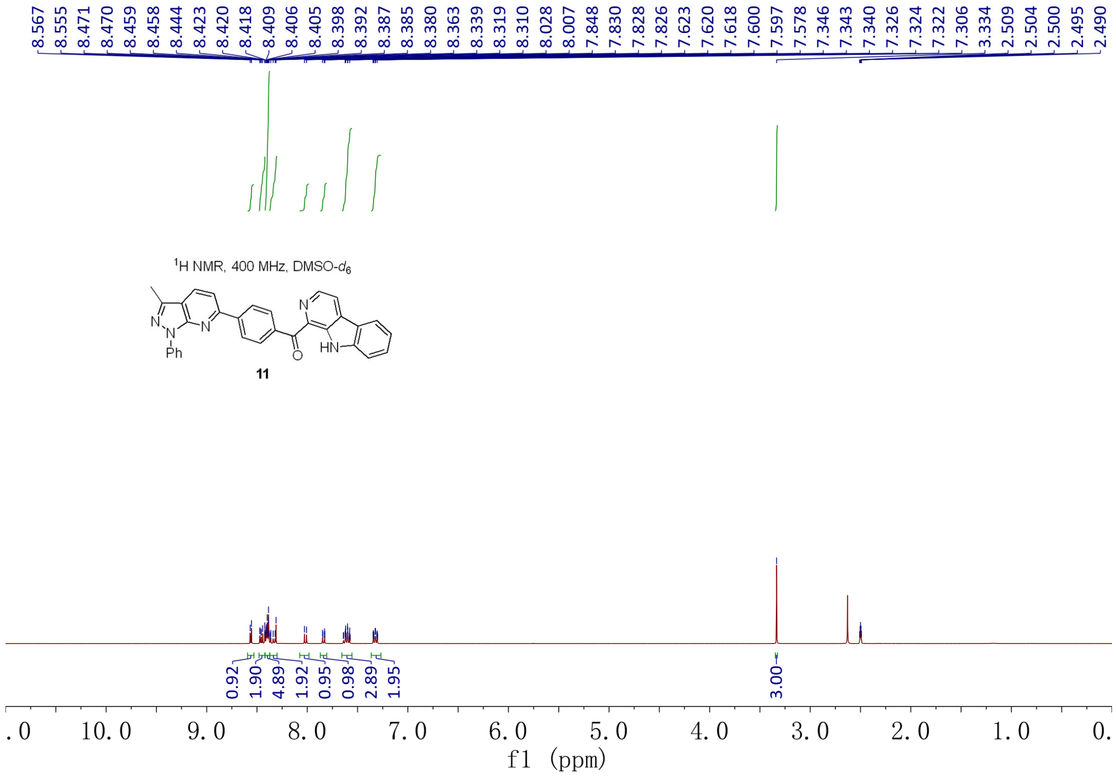
8.567
8.555
8.471
8.470
8.459
8.458
8.444
8.423
8.420
8.418
8.409
8.406
8.405
8.398
8.392
8.387
8.385
8.380
8.363
8.339
8.319
8.310
8.028
8.007
7.848
7.830
7.828
7.826
7.623
7.620
7.618
7.600
7.597
7.578
7.346
7.343
7.340
7.326
7.324
7.322
7.306
3.334
2.509
2.504
2.500
2.495
2.490
1H NMR, 400 MHz, DMSO-d6
N
N
N
Ph
N
O
HN
11
0.92
1.90
4.89
1.92
0.95
0.98
2.89
1.95
3.00
10.0
9.0
8.0
7.0
6.0
5.0
4.0
3.0
2.0
1.0
f1 (ppm)

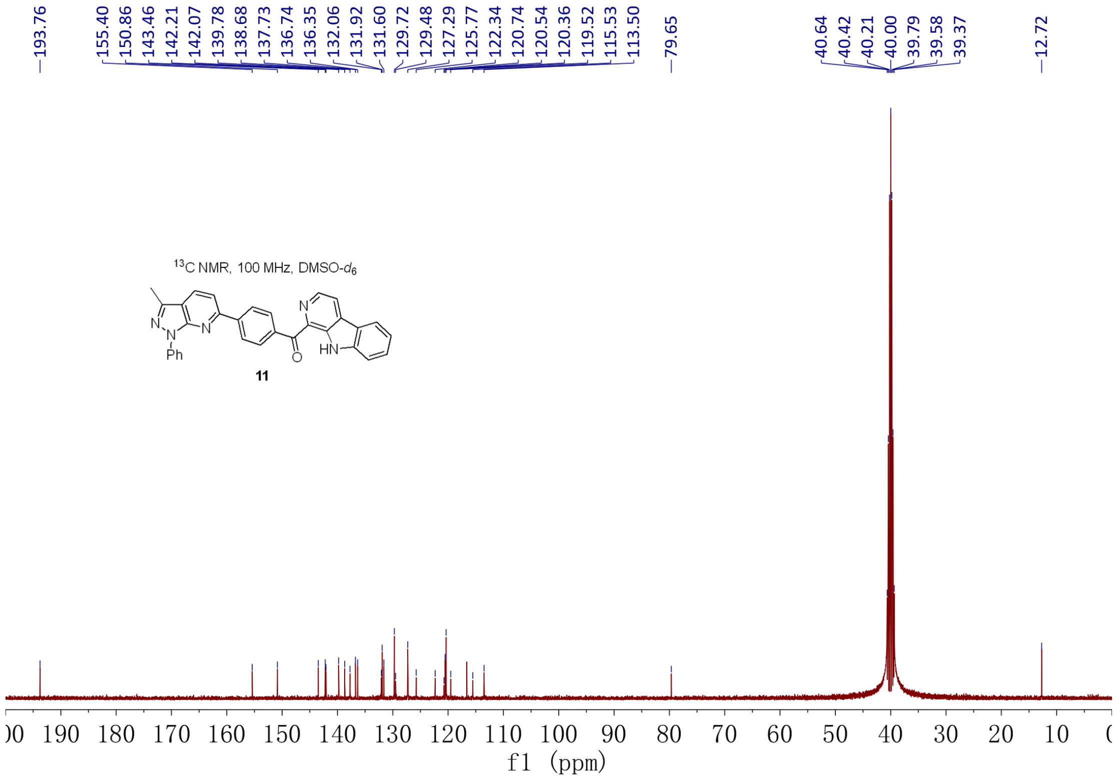
193.76
155.40
150.86
143.46
142.21
142.07
139.78
138.68
137.73
136.74
136.35
132.06
131.92
131.60
129.72
129.48
127.29
125.77
122.34
120.74
120.54
120.36
119.52
115.53
113.50
79.65
40.64
40.42
40.21
40.00
39.79
39.58
39.37
12.72
13C NMR, 100 MHz, DMSO-d6
N
N
N
Ph
N
O
HN
11
190
180
170
160
150
140
130
120
110
100
90
80
70
60
50
40
30
20
10
f1 (ppm)

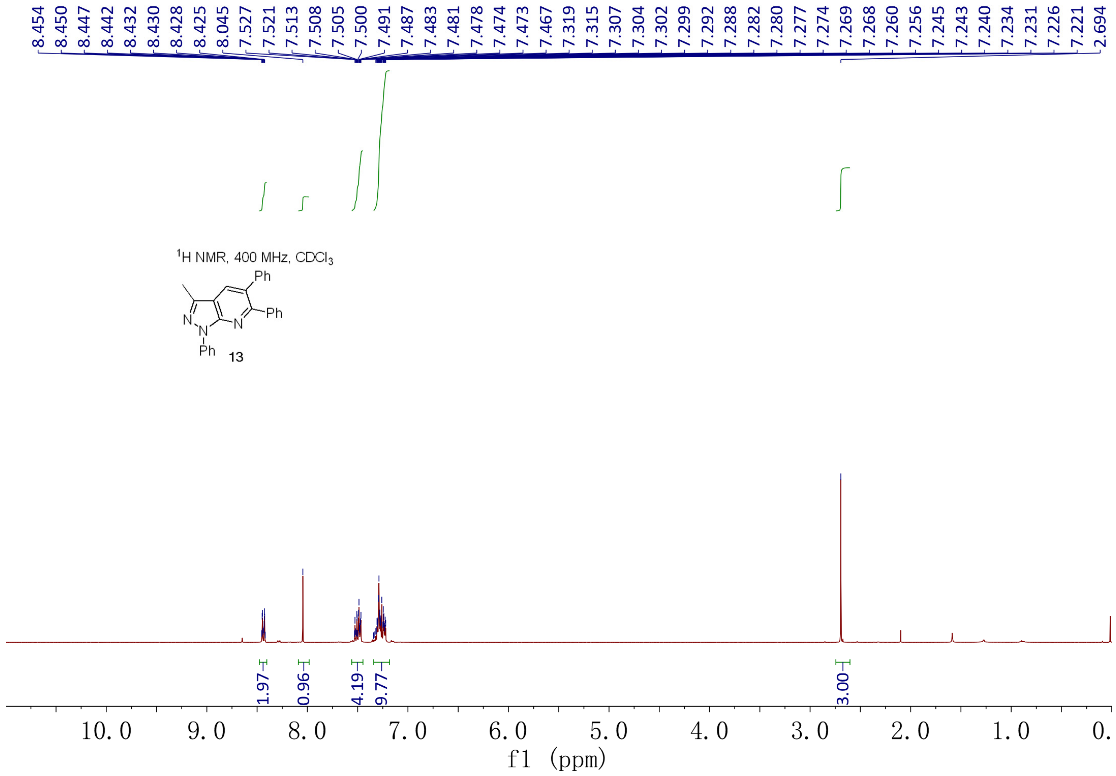
8.454 8.450 8.447 8.442 8.432 8.430 8.428 8.425 8.045 7.527 7.521 7.513 7.508 7.505 7.500 7.491 7.487 7.483 7.481 7.478 7.474 7.473 7.467 7.319 7.315 7.307 7.304 7.302 7.299 7.292 7.288 7.282 7.280 7.277 7.274 7.269 7.268 7.260 7.256 7.245 7.243 7.240 7.234 7.231 7.226 7.221 2.694
^{1}H NMR, 400 MHz, $CDCl_3$
Ph
Ph
N
N
N
Ph
13
1.97
0.96
4.19
9.77
3.00
10.0 9.0 8.0 7.0 6.0 5.0 4.0 3.0 2.0 1.0 0.
f1 (ppm)

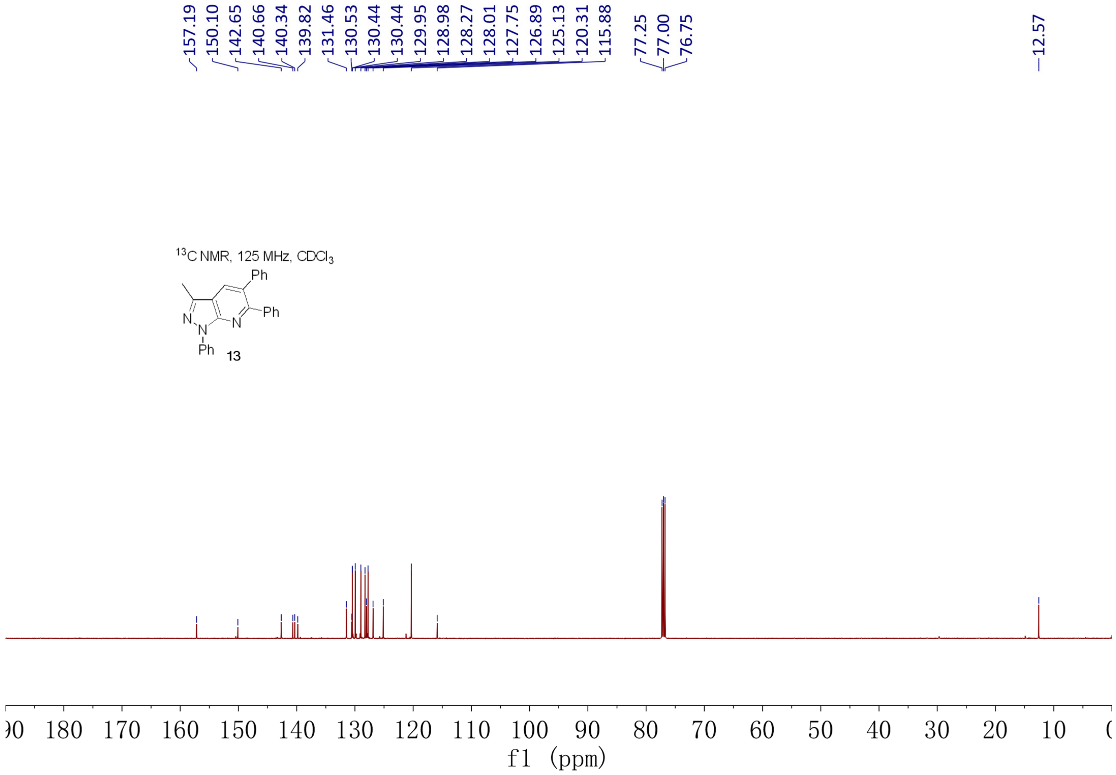
157.19 150.10 142.65 140.66 140.34 139.82 131.46 130.53 130.44 130.44 129.95 128.98 128.27 128.01 127.75 126.89 125.13 120.31 115.88
77.25 77.00 76.75
12.57
^{13}C NMR, 125 MHz, $CDCl_3$
Ph
Ph
N
N
N
Ph
13
90 180 170 160 150 140 130 120 110 100 90 80 70 60 50 40 30 20 10 0
f1 (ppm)

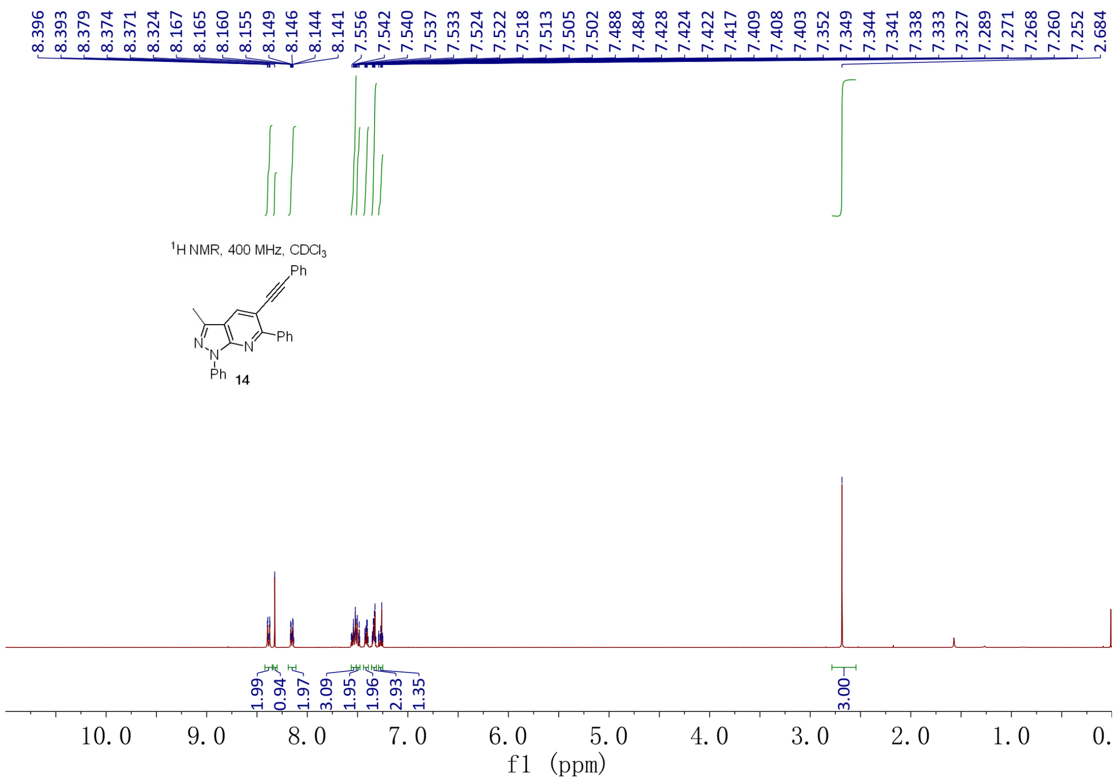
8.396 8.393 8.379 8.374 8.371 8.324 8.167 8.165 8.160 8.155 8.149 8.146 8.144 8.141 7.556 7.542 7.540 7.537 7.533 7.524 7.522 7.518 7.513 7.505 7.502 7.488 7.484 7.428 7.424 7.422 7.417 7.409 7.408 7.403 7.352 7.349 7.344 7.341 7.338 7.333 7.327 7.289 7.271 7.268 7.260 7.252 2.684
1H NMR, 400 MHz, CDCl3
Ph
Ph
N
N
N
Ph
14
1.99 0.94 1.97 3.09 1.95 1.96 2.93 1.35 3.00
10.0 9.0 8.0 7.0 6.0 5.0 4.0 3.0 2.0 1.0 0.
f1 (ppm)

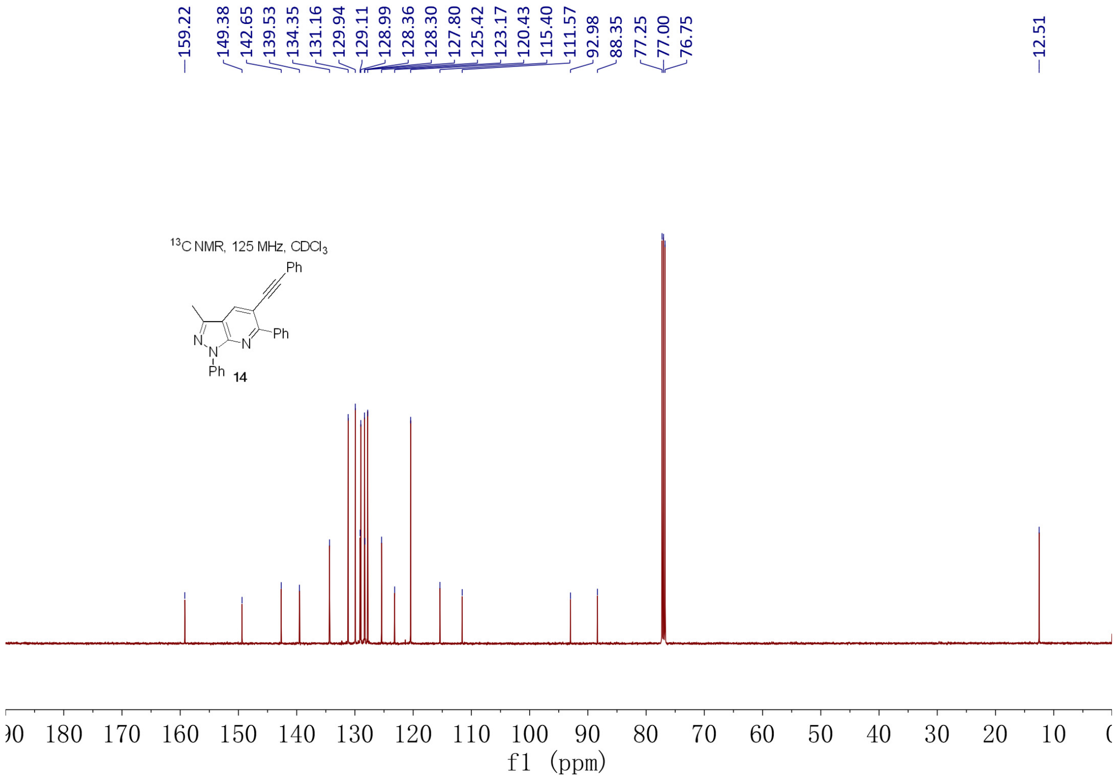
159.22 149.38 142.65 139.53 134.35 131.16 129.94 129.11 128.99 128.36 128.30 127.80 125.42 123.17 120.43 115.40 111.57 92.98 88.35 77.25 77.00 76.75 12.51
13C NMR, 125 MHz, CDCl3
Ph
Ph
N
N
N
Ph
14
180 170 160 150 140 130 120 110 100 90 80 70 60 50 40 30 20 10
f1 (ppm)

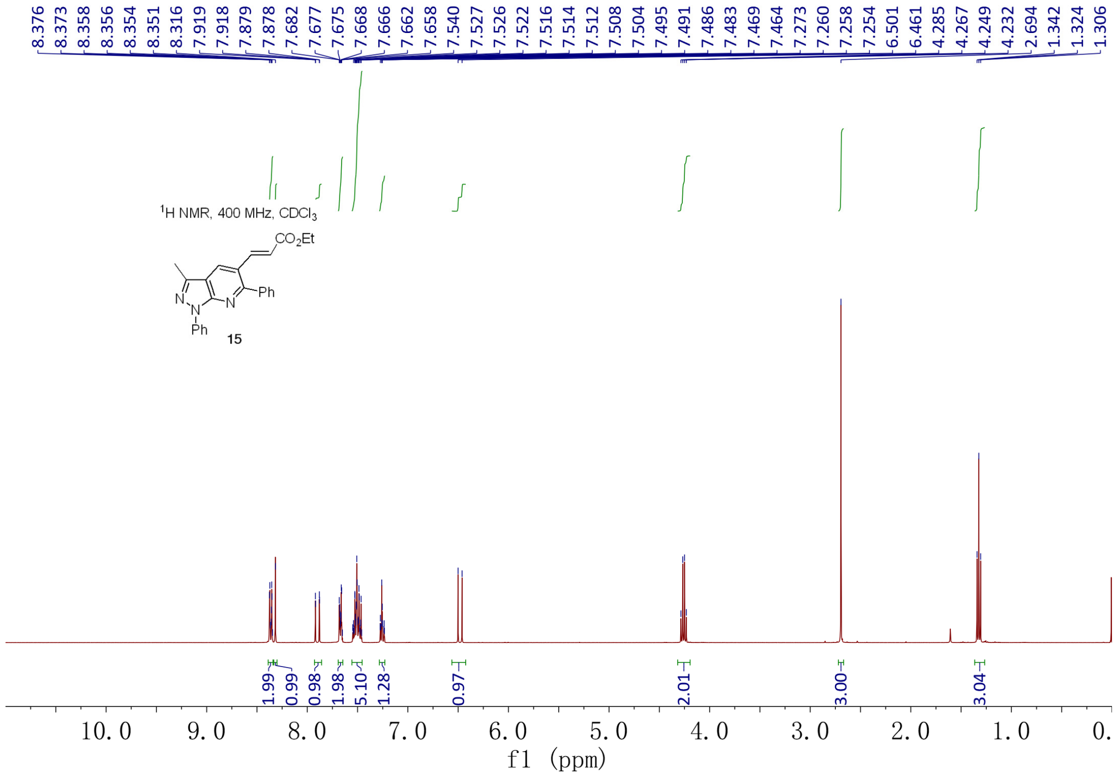

8.376 8.373 8.358 8.356 8.354 8.351 8.316 7.919 7.918 7.879 7.878 7.682 7.677 7.675 7.668 7.666 7.662 7.658 7.540 7.527 7.526 7.522 7.516 7.514 7.512 7.508 7.504 7.495 7.491 7.486 7.483 7.469 7.464 7.273 7.260 7.258 7.254 6.501 6.461 4.285 4.267 4.249 4.232 2.694 1.342 1.324 1.306
1H NMR, 400 MHz, CDCl3
CO2Et
Ph
N
N
N
Ph
15
1.99 0.99 0.98 1.98 5.10 1.28 0.97 2.01 3.00 3.04
10.0 9.0 8.0 7.0 6.0 5.0 4.0 3.0 2.0 1.0 0.
f1 (ppm)

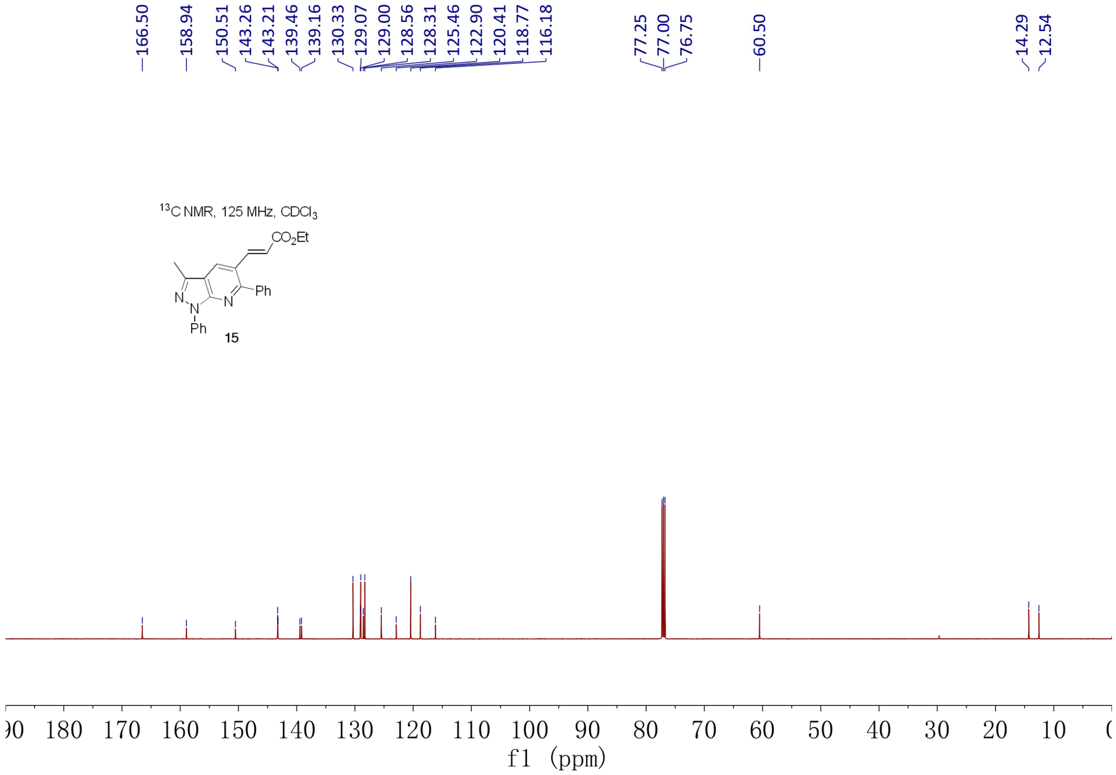

166.50 158.94 150.51 143.26 143.21 139.46 139.16 130.33 129.07 129.00 128.56 128.31 125.46 122.90 120.41 118.77 116.18 77.25 77.00 76.75 60.50 14.29 12.54
13C NMR, 125 MHz, CDCl3
CO2Et
Ph
N
N
N
Ph
15
180 170 160 150 140 130 120 110 100 90 80 70 60 50 40 30 20 10
f1 (ppm)

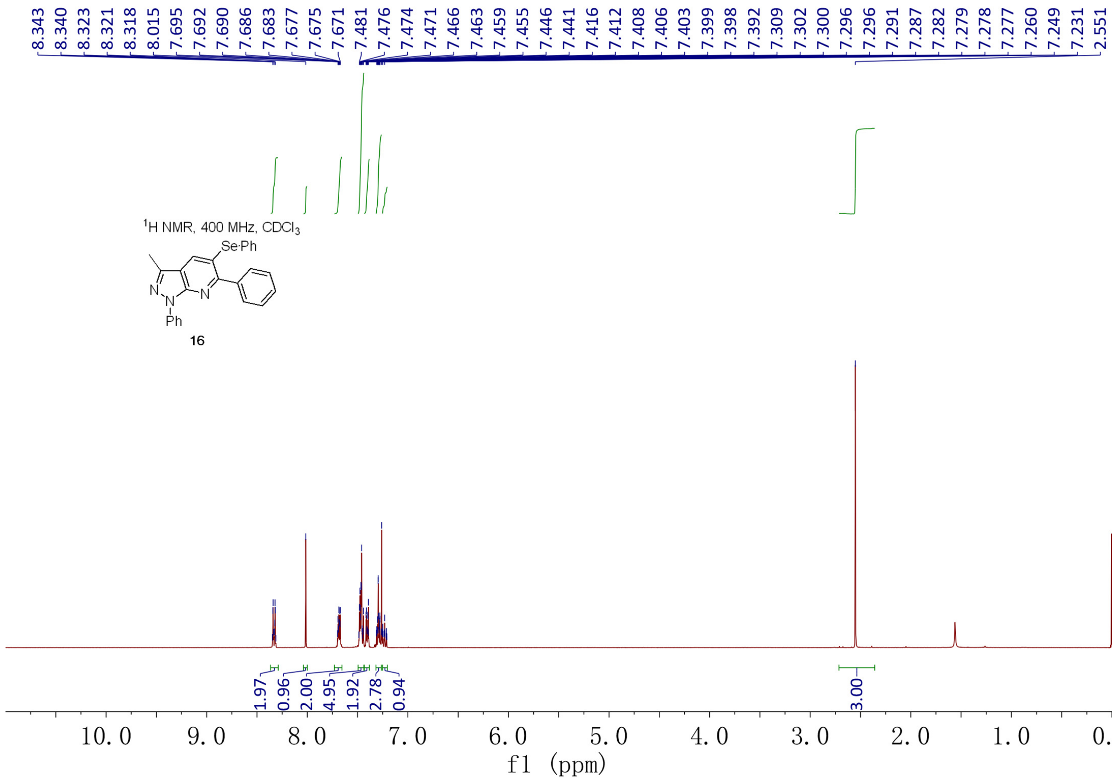
8.343
8.340
8.323
8.321
8.318
8.015
7.695
7.692
7.690
7.686
7.683
7.677
7.675
7.671
7.481
7.476
7.474
7.471
7.466
7.463
7.459
7.455
7.446
7.441
7.416
7.412
7.408
7.406
7.403
7.399
7.398
7.392
7.309
7.302
7.300
7.296
7.296
7.291
7.287
7.282
7.279
7.278
7.277
7.260
7.249
7.231
2.551
1H NMR, 400 MHz, CDCl3
Se-Ph
N
N
N
Ph
16
1.97
0.96
2.00
4.95
1.92
2.78
0.94
3.00
10.0
9.0
8.0
7.0
6.0
5.0
4.0
3.0
2.0
1.0
f1 (ppm)

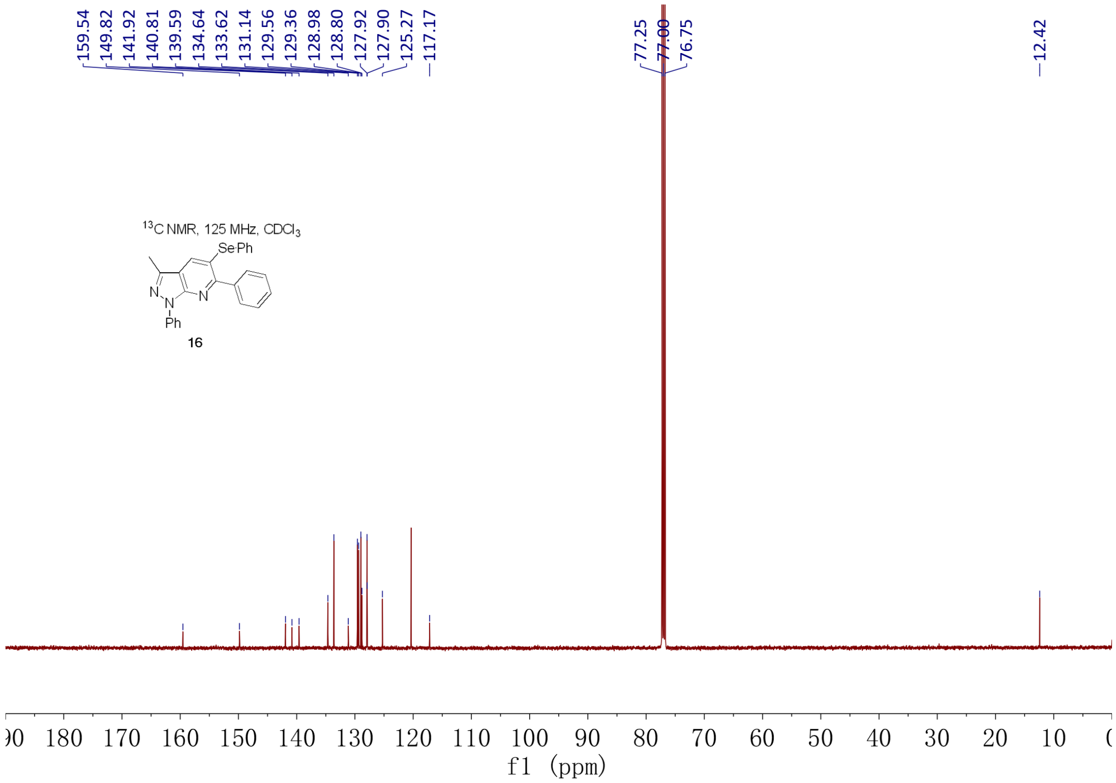
159.54
149.82
141.92
140.81
139.59
134.64
133.62
131.14
129.56
129.36
128.98
128.80
127.92
127.90
125.27
117.17
77.25
77.00
76.75
12.42
13C NMR, 125 MHz, CDCl3
Se-Ph
N
N
N
Ph
16
180
170
160
150
140
130
120
110
100
90
80
70
60
50
40
30
20
10
f1 (ppm)

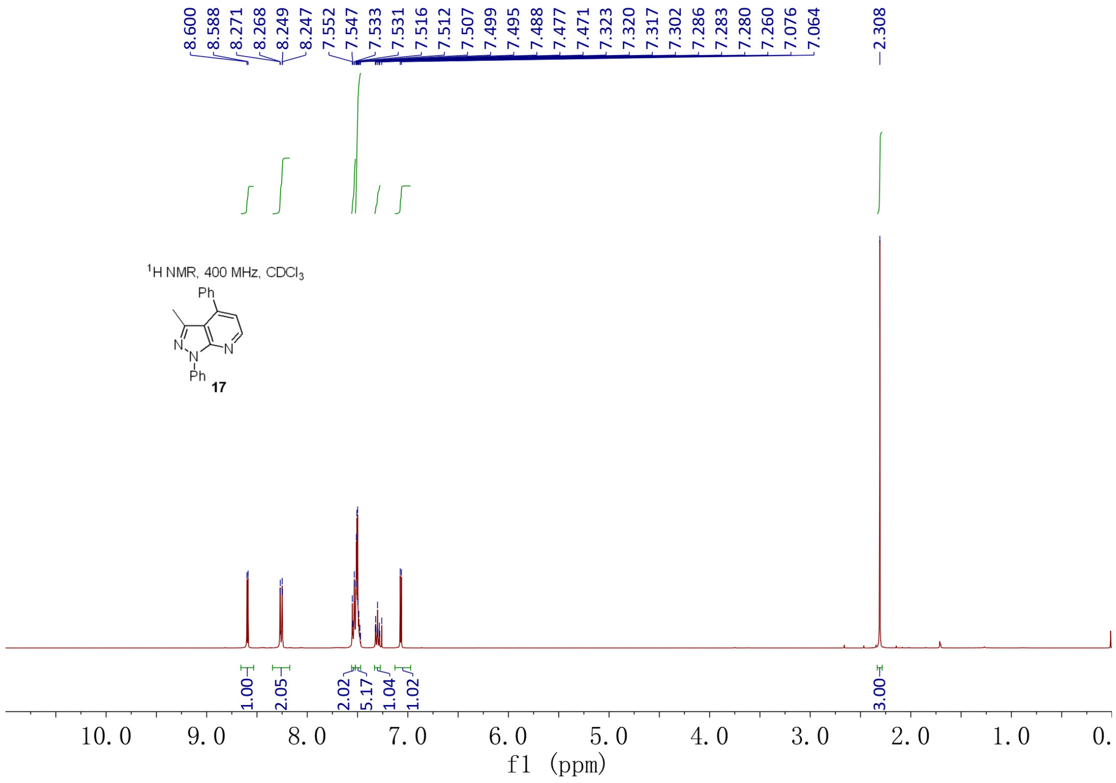
8.600
8.588
8.271
8.268
8.249
8.247
7.552
7.547
7.533
7.531
7.516
7.512
7.507
7.499
7.495
7.488
7.477
7.471
7.323
7.320
7.317
7.302
7.286
7.283
7.280
7.260
7.076
7.064
2.308
1H NMR, 400 MHz, CDCl3
Ph
N
N
N
Ph
17
1.00
2.05
2.02
5.17
1.04
1.02
3.00
10.0
9.0
8.0
7.0
6.0
5.0
4.0
3.0
2.0
1.0
0.
f1 (ppm)

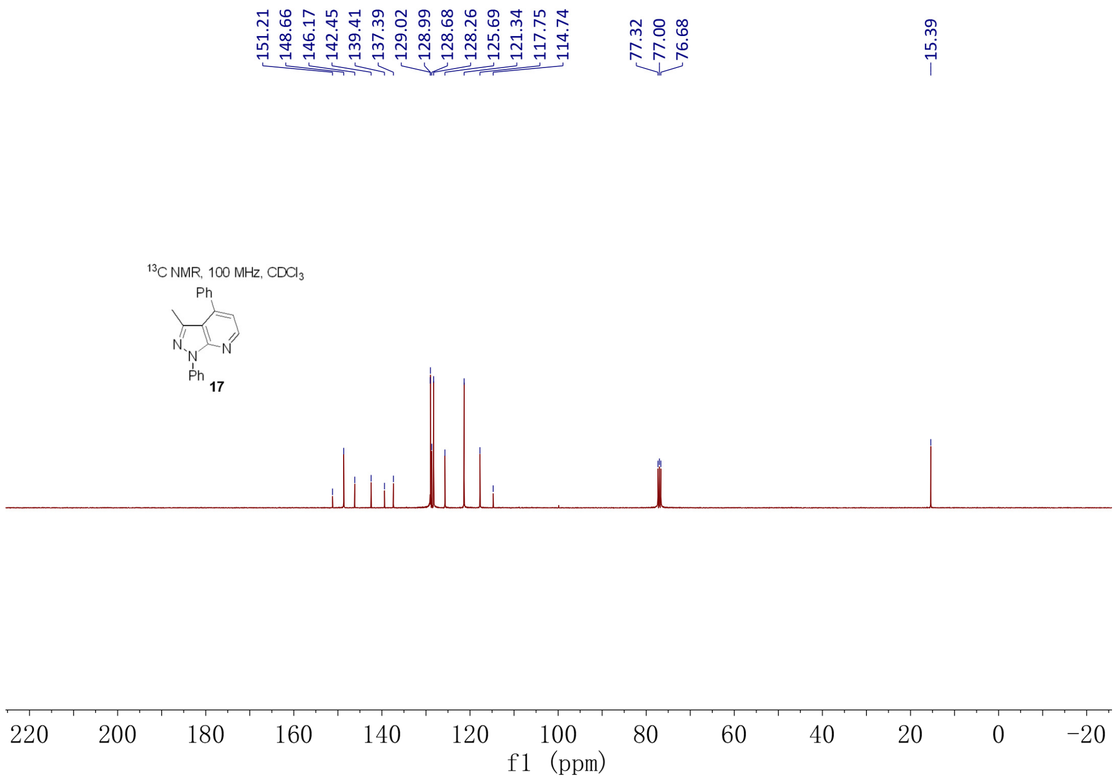
151.21
148.66
146.17
142.45
139.41
137.39
129.02
128.99
128.68
128.26
125.69
121.34
117.75
114.74
77.32
77.00
76.68
15.39
13C NMR, 100 MHz, CDCl3
Ph
N
N
N
Ph
17
220
200
180
160
140
120
100
80
60
40
20
0
−20
f1 (ppm)
